# Anger among Chinese migrants amid COVID-19 discrimination: The role of host news coverage, cultural distance, and national identity

**DOI:** 10.1371/journal.pone.0259866

**Published:** 2021-11-16

**Authors:** Xiaoyuan Li, Alexander S. English, Steve J. Kulich

**Affiliations:** 1 Intercultural Institute, Shanghai International Studies University, Shanghai, China; 2 Department of Psychology and Behavioral Sciences, Zhejiang University, Hangzhou, Zhejiang, China; University of Hradec Kralove: Univerzita Hradec Kralove, CZECH REPUBLIC

## Abstract

As the early COVID-19 outbreak sparked xenophobia against people of Asian and Chinese background, we collected data from Chinese migrants worldwide to test how discrimination at a macro-level was perceived by the Chinese during COVID-19 globally. Specifically, we examined (1) whether/how the Chinese migrants were aware of discrimination against their co-nationals during COVID; (2) if so, whether anger was a predominant reaction of these Chinese towards certain exposure to relevant information; (3) how responses of anger transcend across the group of Chinese migrants. Integrating the ecological approach to media and cultural psychology, as well as the intergroup perspective of social psychology, we conducted a study that explored the impact of traditional media exposure to discrimination on collective anger—a process mediated by national identity among the Chinese migrants. Findings provide some evidence that geographically dispersed mono-cultural groups may share or identify with collective emotions when facing xenophobic threats in a macro context. Further examination of cultural distance (between China and the host country) among the Chinese migrants also revealed a particular interaction between host newspaper coverage and cultural distance on national identity. These findings suggest further research to examine the emotional norms of similar cultures bonded via strong collective identities in times of intergroup threat and the theoretical possibility for diasporic identity processes.

*“It’s divided us, angered us, set us against one another. I know the country’s grown weary of the fight, but we need to remember—we’re at war with the virus, not one another.”*

                                Thanksgiving Speech, U.S. President Joe Biden, Nov. 27, 2020

## Introduction

The COVID-19 pandemic sparked xenophobia and discrimination, particularly against individuals of Asian and Chinese backgrounds [1–3]. As the novel SARS-CoV-2 was first noted and erupted in China in early 2020 and developed into a global pandemic, Chinese living abroad experienced racial stereotyping threats, usually associating them with the deadly pathogen [4, 5]. For example, in the UK, there were more than 260 cases of hate crimes against Chinese people noted between January and March 2020, almost three times that of the previous two years [6]. Recent research has investigated this phenomenon in the ongoing COVID-19 pandemic [7–9]. However, little is known about the affective orientation of those who face discrimination on the collective level.

In the current research, we focus on how Chinese nationals living abroad emotionally responded to group (national) discrimination during early COVID-19. By expanding previous research on media ecology and diasporic identity, we examine the mechanism of information exposure in determining potential emotional contagion in the context of collective threats. Based on data from a Chinese international migrant sample during the early days of the coronavirus in April 2020 and online-sourced host- and ethnic-media coverage noting anti-Chinese discrimination, we examine relevant models with national identity as a mediator and cultural distance as a moderator. Starting with an inductive approach based on self-report responses, we sought to identify (1) the sources of information through which these Chinese expatriates learned about discrimination against co-nationals during COVID-19, (2) the specific type of information exposure leading to anger among these Chinese, and (3) the roles of national identity and cultural distance in this hypothesized group process.

### Media ecology and the cultural migrants

The ecological approach to media and culture was based on the ecological systems theory [10] that proposed a holistic analysis of human development in relation to the surrounding environmental contexts [11, 12]. For media scholars, ecology refers to the complex communication systems as environments [13], in which media and the transactions among messages interact with human thinking and behavior [14, 15]. For cultural psychologists, the ecological perspective offers a lens in which human behaviors are analyzed in both the cultural and situational contexts where individuals are involved in communication practices [12] and intercultural contact [16]. Both media ecology and cultural ecology focus on culture as a macrosystem within “worldwide globalism” [17]. On the one hand, media transmit and represent cultural practices via its products and communication processes [18] based on globalized digital technology [19]. On the other hand, culture is conceptualized as globally variant patterns of psychological functioning that distinguish groups bonded via shared values or beliefs [20], such as individualism and collectivism [21, 22], interdependence and independence [23, 24], and social identity [25]. A clash of patterns and identity is often highlighted among individuals facing challenges of whether or not to sustain the heritage culture when they are in contact with another culture in migration [26–28].

In global migration, the ecological interaction between media and culture is captured in the diasporic media sphere where concepts like the “imagined community” [29], “transnationalized imagined community” [30, 31], and “long-distance nationalism” [32, 33] are used to bracket the “ethnospaces” [34] of culture. Much scholarly attention has been given to the advancement of media technologies and digital communication in the formation and maintenance of transnational diasporic identity [35–38]. For the Chinese diaspora, scholars explore the intricate relationship between Chinese ethnic/national belonging and ethnic media usage [39–41]. For example, Sun [42–45] conducted prolific research on the consumption of Chinese ethnic media (press, films, TV, the Internet, and social media) among the Chinese in constructing a transnational sense of belonging to the home place.

These studies underscore the symbiosis of media consumption and individuals’ search for cultural belonging in an “intermedia dimension” [14, p. 209]. However, as much as “culture” is emphasized, little is known about the specific intercultural factors that undergird or interact in these cultural and media processes in transnational migration, such as mediated intergroup contact via host or ethnic reportage. Currently, academic understanding of host media consumption has not clearly indicated the relationship between host media and immigrant identity, although most literature highlight the importance of acculturation attitudes and socio-cultural context in determining migrants’ media behaviors and identity. Research suggest that higher host media consumption is related to bicultural identity orientation [46, 47], whereas under unique circumstances mediated negative news coverage may consolidate minority identity and reduce assimilation tendencies [48]. Also, a potential ecological factor to consider is the proximity of culture and geography between countries in determining international news coverage and newsworthiness [49, 50], such that culturally close countries may report on each other in ways reflecting a more reciprocal process of interaction that influences sojourners’ cultural awareness and identity.

Traditionally, the term “diaspora” refers to the scattering and migration of a minority group with a shared ancestral homeland [51, p.8]. It is also used as a metaphoric designation for several categories of people away from the homeland, such as expatriates and alien residents [52]. In the case of the Chinese diaspora, it has often included ethnic Chinese immigrants, sojourners, and temporary migrants worldwide irrespective of their migration history and factual citizenship to encompass a complex transnational population [53]. Recently, scholars have also proposed international students as alternative visions of diaspora to maximize the empirical significance of this population in diasporic studies [54]. In the current research, we focus mainly on Chinese international students and expatriates as the population under study. Our research purpose is to understand the macro-level, socio-political context of xenophobia during COVID-19 and some ecologically relevant factors in understanding the psychological processes of national cultural groups under threat in migration. We explore whether these Chinese sojourners residing in different countries could somehow share the “symbolic mediation of experiences” [55, p. 740] via information exposure to anti-Chinese discrimination. We seek to capture their reactions during this naturally occurring pandemic to test how worldwide discrimination towards the Chinese was perceived, and to fully unpack how these cultural individuals emotionally responded to a global health crisis. We examine this proposition based on the assumption that similar exposure to media content and forms may involve collective identity in processes that are expressed by emotions.

### Information exposure and social identity

As is discussed earlier, even though diasporic media studies generally focus on identity as a cultural level construct, the theoretical and methodological approach to issues of collective identity are qualitatively bracketed within the dynamic context of global mobility [39]. As a result, little research has explored a potentially quantifiable relationship between media exposure and the migrants’ cultural/national identity.

Beyond the cultural mediascape, current communication research has extensively examined the effects of media exposure on audience reception [56–60]. This uni-directional approach suggests a socio-cultural conceptualization of media in cultivating the context of real-world happenings and impacting people’s attitudes toward relevant incidents [58]. For example, researchers found that media framing of immigration might impact people’s attitudes towards immigration irrespective of the valence (negativity/positivity) of news stories [61]. In the intergroup context, media scholars proposed the social identity model of media effects [62], on the premise that “when a person is exposed to media content, one or more in-groups will become salient” [63]. In this process, exposure to media content may challenge individuals’ perception of their group vitality based on whether the information is congruent with their social beliefs [64]. If this information is incongruent, such as in the face of negative media stereotyping of one’s ingroup, people would reinforce their group identification for collective changes. The best case-in-point is how Muslim Americans sought responses to identity threats when perceiving negative media representations of their religious ingroup [65], and felt their religious identity was under attack after 9/11 [66]. Other research has explored the possibility for the negative media effect on the Muslim identity to carry over to the Palestinian diaspora in the UK as a result of shared religious belief [67].

The influence of media exposure on collective identification is not restricted to traditional media. It is well-known that social media has the power to galvanize collective agency, as is most recently demonstrated by the Black Lives Matter movement [68]. In diasporic media studies, scholars have proposed the term “digital diaspora” to capture the dynamic nature of digital media consumption served for migrants’ national bonding via shared experiences [69]. For media ecologists, the multi-level, multidimensional process of information dissemination is also viable through interconnected entities in the communication process, such as interpersonal communication, intercommunity communication, and local news diffusion [70, 71]. This diffusion proposition is supported by studies that identified a vicarious group effect on victimization and discrimination. For instance, Gross and Aday [72] found that direct experience with victimization towards oneself or one’s close others had an effect on fear of victimization, whilst local news exposure had an agenda-setting effect. Wofford and colleagues [73] identified a vicarious effect of sharing discriminatory experiences in interpersonal relationships on mental health. Other research also found associations of direct and vicarious racism with negative affect [74, 75], and that vicarious racism occurs from both observation of one’s immediate environment and from media coverage of racist incidents [76].

We postulate that vicarious discrimination has an effect on the psychological experiences of individuals, not merely from an individual’s exposure to media itself, but to the near social environment in which the individual absorbs information as well. Unfortunately, to date, few studies have examined the shared experiences with discrimination among mono-cultural migrants across multiple countries, even if they are not directly connected in real life. It is within this background that we argue for a direct understanding of the current impact of COVID-19 experiences of stigmatized populations like the Chinese, who have faced increased threat and negative stereotyping as a result of similar media consumption of citizens in their social environments [77]. In the current research, we investigate whether the Chinese migrants would respond negatively to collective stigma during COVID-19 via vicarious exposure to discriminatory incidents. Next, we elaborate on this potential process from the perspective of intergroup emotions.

### Intergroup emotions

Emotions are transferred or assumed across groups. “People imagine, estimate, guess, intuit, or simulate what others feel even if direct information about their responses is not available” [78, p. 5]. According to intergroup emotions theory [79], emotions can be experienced collectively as a result of group categorization and group identification. Group-based emotions are susceptible to specific events and are influenced by a salient social identity [80, 81]. It is argued that people represent the emotional responses of those who are psychologically salient by responding to acute stimuli or chronological ingroup membership [78, 82]. For example, previous research has shown that people may be motivated to share group-based emotions to satisfy a sense of belonging, even if these emotions are generally not considered to be pleasant, such as group sadness in the context of the Israeli National Memorial Day [83].

In the context of pandemics, theories about behavioral immune systems have revealed how people demonstrate aggressive emotions and behaviors towards sickly others and initiate strong in-group solidarity in avoiding contagious outgroups [84, 85]. However, not only do those who practice discrimination form or strengthen an ingroup identity seeking to gain existential security, the discriminated outgroup might also bond via stigma-based solidarity for collective efficacy [86]. During COVID-19, shared identity and emotions were shown to be salient when individuals self-categorize as being members of a group experiencing a common threat or emergency [87, 88]. It is possible that as the targets of discrimination, individuals may be preoccupied with group categorizations like nationality and race if such identities are ascribed to them [89].

In discrimination research, one emotional state that stands out as an automatic prompt for out-group prejudice and discrimination is anger, compared to sadness or other more neutral states [90]. It is proposed that threats posed by out-groups, such as competition for resources and contamination, will induce distinct emotions like anger and disgust [91, 92]. Research has validated that group-based anger towards an out-group predicts stronger ingroup identification [93] and identification with the advantaged ingroup also predicts anger towards the disadvantaged out-group [94]. However, group identification and collective anger does not just have an effect on those who discriminate against others–when lower-status group members recognize discrimination, they have been shown to mobilize collective anger and action to counteract status hierarchies [95, p. 29]. Individuals who feel that members of their group are being discriminated against and derogated often respond to and demonstrate group-based anger and collective action, even if this perceived discrimination of the group they identify with (self-stereotyping) only comes from secondary sources [96], which could include word of mouth or media reports.

Accordingly, ingroup members share the same emotional involvement based on their group’s appraisals and their membership in it [97]. One crucial question is, what contextual exposures do individuals need in order to enhance group identity and to get angry, even if they are not physically situated in the conflict? Based on the above theoretical underpinnings, we propose that shared stigma via the social and media environments among a cultural group will encourage its members to identify with each other and negatively react to the same perceived stimulus. In the current study, the perceived stimulus is anti-Chinese discrimination during COVID-19.

### Rationale for the current research

Scholars in media ecology propose that media constitute the means of how we know and understand things in the human environment, aside from the biological and geological environment [98]. Research on media exposure and vicarious emotional arousal have revealed that individuals are capable of locating themselves in media events and automatically respond to the emotional content that they are exposed to [99–102]. Applying this to the COVID-19 pandemic, media may have helped form an adverse emotional climate [103]. Research among the Chinese population found that media exposure has an effect on negative emotions via media vicarious traumatization [104, 105]. Chang and colleagues [106] also revealed how online news served as a hotbed for blaming and negative emotions towards the Chinese during COVID-19. More importantly, beyond typical collective bonding as a result of discrimination, the occurrence of COVID-19 may have galvanized a greater sense of collectivism among the Chinese in coping with the pandemic, as revealed by a social media analysis [107].

If knowing that Chinese abroad experienced discrimination might arouse identification and emotional reactions towards this information, then *where* and *how* such information is acquired becomes a key ecological component to this group process. We base our assumptions on the fact that despite widespread narratives and anecdotes about discrimination against the Chinese during COVID-19, it is improbable for the migrant group at large to be directly and physically inflicted, especially if many were confined by shelter-at-home lock-down measures. Although physical distance to discriminatory events might undoubtedly impact the perceived severity of events, we argue that national identity is a crucial link minimizing the impact that distance normally has on emotional arousal to perceived group threat, so much so that Chinese abroad can still “feel for” each other via exposure to similar information. Following this rationale, we first ask

**RQ1**: Were the Chinese migrants abroad aware of discrimination against co-nationals in other countries during COVID-19? If so, would certain source(s) of information arouse anger among them? Could this process be mediated by national identity?

To address this set of exploratory questions, we propose that in the current study, overseas Chinese could be exposed to information about anti-Chinese discrimination from several sources: traditional news reporting (TV and newspapers [print and digital]) and communication on social network sites (SNS). Furthermore, we made the important distinction between news reports and digital “word of mouth” propagated anecdotes (such as tweets and narratives of discrimination messages) on SNS, as these two types of information have a varied impact on the perceived credibility and severity of social incidents among users [108]. This exploratory analysis intends to understand whether and what sources of information can impact group identity and emotions among the Chinese migrants when they know about discrimination against co-nationals.

Whilst using an exploratory method in understanding the researched phenomenon, we were also aware of the biases that self-report measurement of information exposure might bring to our analysis. Following the previously discussed media effect on social identity, we seek to verify whether host and ethnic Chinese media coverage on anti-Chinese discrimination, as an objective reflection of information exposure in the host environment, would confirm relevant processes indicated by participants’ self-reports. This confirmatory approach may further allow us to achieve a better understanding of media effect by addressing the next question

**RQ2**: Will exposure to host or ethnic media coverage on anti-Chinese discrimination arouse anger among the Chinese migrants by enhancing their national identity?

Following RQ2, we further consider the ecological factor of cultural distance in the proposed effect of information exposure on national identity and anger. As will be revealed in the next section, our study contains a sample of Chinese migrants from 33 world countries in the context of one global event and intergroup phenomenon—COVID-19 and anti-Chinese discrimination. Therefore, it is imperative for the current research to examine the underlying factors embedded in country-level differences. We base our rationale on the fact that different countries have dynamic and variant patterns of international news flow [109]. These patterns are, among others, determined by ideological and cultural factors [110], such as cultural affinity and cultural proximity between countries [111], as well as the West/non-West imbalance in international news flow [112]. For example, previous research has found a dominating role of core countries in international news flow [113], and that cultural proximity may differentiate people’s acceptance of US media products by whether they were in the USA, Asia, or Europe [114]. Furthermore, based on the classic social identity theory that in-group favoritism and out-group hostility enables discrimination [115], it is also possible that beyond cultural distance, another confounding factor in the happenstance or reportage of discrimination incidents targeting the Chinese is a country’s existing attitude toward China as a result of pre-pandemic geopolitical situations, such as the U.S.- China trade war before 2020. Considering these possibilities, we specifically adopted a measurement of cultural distance between different countries and China [116], as well as their pre-pandemic favoritism towards China as a control factor for raising the third question

**RQ3**: Will cultural distance between China and the host country interact with host or ethnic media coverage in affecting national identity among the Chinese migrants?

## Materials and methods

### Participants

The original sample had 345 participants, among which 14 were excluded as non-Chinese citizens (identified by passport) as the research mainly examined Chinese national identity, and 5 more were excluded as outliers using the Mahalanobis Distances method. The final sample consisted of 326 Chinese participants (*N*_female_ = 226, 69.3%) who were residing outside China or had just repatriated during the COVID-19 crisis (dividing repatriates/non-repatriates in the sample did not impact major variables; see S1 Table in S1 File). These participants resided in 33 countries, among whom 66.9% were students (*n* = 218), 14.7% were temporary and permanent residents (*n* = 48), 12.6% were migrant workers (*n* = 41), 5.8% were visitors (*n* = 19). The majority of participants resided in the USA (*n* = 60, 18.4%), UK (*n* = 41, 12.6%), Spain (*n* = 41, 12.6%), Italy (*n* = 25, 7.7%), Germany (*n* = 18, 5.5%), and Japan (*n* = 15, 4.6%). All participants were recruited via online convenience and snowball sampling from April to May 2020. The participants were aged between 18 to 53 (*M* = 27.52, *SD* = 6.087) with an average of 40.40 months of stay in the host country (*SD* = 42.296). S1 Fig in S1 File shows the distribution of participants in each country.

### Measures

Participants completed an online survey on Qualtrics. They were informed that the research was to understand discrimination and sense of belonging during COVID-19, as well as the confidentiality of their personal information. Major scale items were translated into Chinese and back translated into English to guarantee content reliability. The survey included items related to the topics/sub-scales noted below, relevant demographic questions, and two scales of perceived discrimination not adopted for the current study (please refer to S1 File for relevant analysis).

#### Source(s) of information

Participants were asked, “How do you know about discrimination against Chinese abroad during COVID-19?” and selected from a list of the following items: 1. TV and (online) newspaper reports (Traditional Media); 2. News on social networking sites (SNS News); 3. Anecdotes on social networking sites (SNS Anecdotes); 4. Acquaintances; 5. Self-experience; 6. Others; and 7. “I do not know.” Participants were allowed to make multiple choices. Each item was dummy coded into a dichotomous variable (Yes = 1; No = 0). For example, if participants selected Item 1, then the variable is coded as Traditional Media = 1. Supplementary materials include full analyses of all sources of information and correlations (S4 Table in S1 File).

#### National identity

National identity was measured using the Collective Self-Esteem Scale (CSES) by Luhtanen and Crocker [117]. The CSES is a 16-item scale containing the four constructs of Membership, Private Self, Public Self, and Identity. The wording of the items was adapted to pinpoint participants’ Chinese identity since prior validations of the scale suggested that the items could easily be adapted to assess self-esteem concerning one specific group membership or nationality [118]. For example, the item “I am a worthy member of the social groups I belong to” was phrased as “I am a worthy member of my nation.” Participants were prompted to think about their Chinese national membership before scoring the items on a 5-point Likert scale ranging from “strongly disagree” to “strongly agree.” Among the 16 items, items 2, 4, 5, 7, 10, 12, 13, 15 were reverse coded. The overall scale showed sound reliability (Cronbach’s α = .876). Participants scored a mean of 3.95 with a range from 2.44 to 5.00 (*SD* = .504), suggesting strong yet variant degrees of national identity. The Chinese translation of CSES can be found in S12 Table in S1 File.

#### Anger

Participants were asked to rate on a 5-point Likert scale from “Strongly Disagree” to “Strongly Agree” on the statement: “You feel angry when knowing that Chinese are discriminated abroad during COVID-19.” The participants scored a mean of 4.22 (*SD* = .703) on this measurement. We also used other exploratory emotional analyses, showing a basis for why anger was selected as an important outcome variable. Please refer to S2 Fig and S2, S3 Tables in S1 File for support.

#### Host newspaper coverage

As an objective proxy for host media exposure, we measured host newspaper coverage by counting the total number of online news reports on anti-Chinese discrimination from the top 3 newspapers in each country. This approach was based on previous findings that 60% of top stories on news websites covered the same topics as covered by traditional media (newspaper, TV and radio) [119]. To ascertain that we captured newspapers representative of host traditional media, we used print circulation data as a ranking criterion to identify the top 3 newspapers in each country, based on similar rationales applied in traditional media research [120]. Wherever circulation information was unavailable, other ranking criteria (such as website ranking) were adopted from relevant sources (S7 Table in S1 File). We further conducted keyword combination searches for the terms “discrimination”/“stigma”/“racism”+ “Chinese”/“COVID” on the website of each newspaper with the start date set at January 23, 2020, the date of the Wuhan lockdown (after which global travel bans towards Chinese were carried out); the end date was set at the day when the last survey response was submitted by participants in each country. All searches were conducted in the language of the newspaper (e.g., Dutch for *Het Nieuwsblad* in Belgium and Japanese for *Asahi Shimbun* [朝日新聞] in Japan) with the help of Google translation. In all, our sourcing counted for 379 host news reports from 80 online newspaper sites across 33 countries. A Host Newspaper Coverage score was assigned to each participant based on the number of reports we found for each country (*M* = 16.34, *SD* = 9.286). All host newspaper listings, references, data collection, country-level host news reports and hyperlinks, can be found in S7, S8 Tables and S6, S7 Figs in S1 File.

#### Ethnic newspaper coverage

Similarly, as an objective proxy for ethnic Chinese media exposure, we measured the total number of online news reports on anti-Chinese discrimination from leading ethnic Chinese newspapers in each country, with the overall aim of sourcing data from the top 3 ethnic Chinese news outlets by circulation ranking. We accessed ethnic newspaper ranking for countries with considerable Chinese immigrant population (e.g., U.S. and Australia), whereas some countries only had three or fewer ethnic Chinese news portals available online and were hence included in the source data regardless of circulation ranking (e.g., Belgium and Greece). For other countries, no ethnic Chinese news outlet or portal could be identified (e.g., Saudi Arabia & Uruguay) and their ethnic Chinese coverage was coded as zero. Using the same approach, we conducted brief keyword combination searches for the Chinese terms “华人”/“疫情” + “歧视” (“Chinese”/“COVID”+“discrimination”) on these ethnic Chinese news portals in an attempt to delimit search results with further content verification by three Chinese coders. All the articles were dated from January 23, 2020, until the date of the last survey response from each country. In all, our sourcing counted for 548 ethnic Chinese news reports from 73 online newspaper sites. An Ethnic Newspaper Coverage score was assigned to each participant based on the number of ethnic reports we found for each country (*M* = 49.81, *SD* = 53.375). All details of this ethnic Chinese newspaper sourcing can be found in the S9, S10 Tables and S8, S9 Figs in S1 File.

#### Sino distance

In our study, Sino Distance refers to the degree of cultural differences between China and the host country. Similar with a previous study on cultural distance and sojourners’ experiences in China [121], we acquired secondary data from Muthukrishna and colleagues [116] who designed a Chinese scale of measuring cultural distance by incorporating biological index and data from the World Values Survey (WVS) with the Chinese data as a baseline. Adopting this data, we were able to identify the Sino-distance values for 28 countries in our sample. For the five countries (Portugal, Belgium, Angola, Saudi Arabia, and Kenya) not listed in Muthukrishna et al. [116], we took the mean of bordering countries’ values to generate an approximate value of Sino-distance, based on a similar approach adopted by Suanet and Van de Vijver [122] in calculating cultural distance. A Sino Distance score was assigned to each participant in accordance with their reported host country (*M* = .14, *SD* = .027). S13 Table in S1 File shows details of the Sino distance score for each country.

#### Sino favoritism

Sino Favoritism refers to the degree of favorable attitude a country held toward China before COVID-19. To measure this variable, we accessed Pew Research Center’s 2019 Spring Global Attitudes Survey [123] dataset and adopted its global data on attitudes toward China from Item “Q17b. Please tell me if you have a [very favorable, somewhat favorable, somewhat unfavorable or very unfavorable] opinion of China”. Scores for this item were reverse coded, calculated on the country-level, and assigned to each participant in the current study so that a higher score indicates more favorable attitude held by the host country toward China before the pandemic (*M* = 2.24, *SD* = .262). 20 country scores were achieved using this data, countries missing in the 2019 survey were amended for by Pew’s 2017 data (Vietnam, Peru, and Chile) and 2020 data (Belgium). Scores for missing countries (*n* = 9) from all years were calculated using series mean. Given the potential inadequacy of how missing values were amended, we also used World Bank’s 2019 and 2018 data on global trade with China [124] and calculated each country’s export-to-China volume change over year as a proxy for attitude toward China, for which positive export volume change over year would indicate more favoritism [125]. This trade change variable was significantly correlated with Sino Favoritism (*r* = .456, *p* < .001), approving the robustness of our measurement. Details of these data and correlations can be found in S13, S14 Tables in S1 File.

## Results

### Analytical procedure

As has been explained in the previous part, we first adopted an inductive approach based on a self-report multiple-choice question to find out the potential sources of information from which the Chinese migrants became aware of anti-Chinese discrimination during early COVID-19. Items in this question allowed us to generate a set of binary variables that represented each source of information, which we would use as potential predictors to explore the specific type of information exposure that would influence anger and national identity (as a mediator). This exploratory method would indicate the viability of potential model(s) for us to concurrently confirm via online sourced data—host and ethnic Chinese newspaper coverage—as a form of objective media/information exposure. On the basis that our hypothesized model stood via the confirmatory effort, we further introduced a third factor—Sino distance—to test its interaction with the confirmed predictor of anger and national identity. Next, we lay out our analytic process step by step.

### Exploratory analysis

We calculated the means, standard deviations, and correlations among the measures and demographic variables. Gaining information about discrimination from traditional media was significantly correlated with both anger (*ρ* = .122, *p* = .028) and national identity (*ρ* = .199, *p* < 0.001), as was the factor related to SNS news (*ρ*_anger_ = .123, *p* = .026; *ρ*_national identity_ = .166, *p* = .003) (Table 1). Besides traditional media and SNS news, other sources of information were not correlated with either anger or national identity (S4 Table in S1 File), suggesting that anecdotal “word of mouth” or direct experiences with discrimination had minimal vicarious group effects in the context of anti-Chinese discrimination. Similarly, host newspaper coverage was positively correlated with anger (*r* = .115, *p* = .038) and national identity (*r* = .115, *p* = .038), and negatively correlated with Sino Favoritism (*r* = -.327, *p* < .001) to a significant level. Anger and national identity were significantly correlated (*r* = .293, *p* < .001). Ethnic newspaper coverage was neither correlated with anger (*r* = .012, *p* = .827) nor national identity (*r* = .011, *p* = .844); but it was positively correlated with host newspaper coverage (*r* = .286, *p* < .001) and Sino Distance (*r* = .176, *p* = .001), and negatively correlated with Sino Favoritism (*r* = -.384, *p* < .001). These results suggest that the amount of host newspaper coverage and ethnic Chinese newspaper coverage were on par with each other, and countries more favorable toward China had fewer reports on anti-Chinese discrimination, suggesting probably fewer discriminatory incidents. Besides, Sino Distance was correlated with ethnic newspaper coverage (*r* = .176, *p* = .001) and Sino Favoritism (*r* = -.149, *p* < .01), suggesting that countries culturally farther from China had more ethnic Chinese reports and were less favorable toward China. In conclusion, the strong correlations of media-relevant variables (self-reported exposure to traditional media and SNS news, plus host newspaper coverage as a proxy for host media exposure) with anger and national identity, indicated that the causal inference of traditional media exposure as the predictor of anger and national identity could be made and further tested.

**Table 1:** Means, standard deviations and correlations of major variables.

N = 326	1	2	3	4	5	6	7	8	9	10	11	M	SD
1. Gender	-											1.70	0.46
2. Age	-0.14*	-										27.52	6.10
3. Education	-0.06	0.38***	-									6.98	1.49
4. Traditional Media	-0.06	-0.05	-0.02	-								0.56	0.50
5. SNS News^a^	-0.05	-0.01	-0.03	0.14*	-							0.90	0.31
6. Host Newspaper Coverage	0.06	-0.09	0.08	-0.06	0.08	-						16.34	9.29
7. Ethnic Newspaper Coverage^b^	-0.12*	0.12*	0.23***	0.06	-0.02	0.29***	-					49.81	53.37
8. Anger	-0.03	-0.01	-0.04	0.12*	0.12*	0.12*	0.01	-				4.22	0.70
9. National Identity	0.04	0.01	-0.01	0.20***	0.17**	0.12*	0.01	0.29***	-			3.95	0.50
10. Sino Favoritism	0.06	-0.07	-0.18***	0.13*	0.04	-0.33***	-0.38***	0.06	0.19***	-		2.24	0.26
11. Sino Distance	0.03	0.08	0.13*	0.00	0.05	0.04	0.18**	-0.02	-0.09	-0.15**	-	0.14	0.03

***p< = 0.001

**p< = 0.01

*p< = 0.5.

Note: Traditional Media Use and SNS News are binary variables (1 = Yes; 0 = No) and used Spearman’s correlation.

Host/Ethnic Newspaper Coverage refers to the number of news reports on anti-Chinese discrimination in each country by host and ethnic media (Jan.23 - Apr.23, 2020).

### Confirmatory analysis

**Fig 1:**
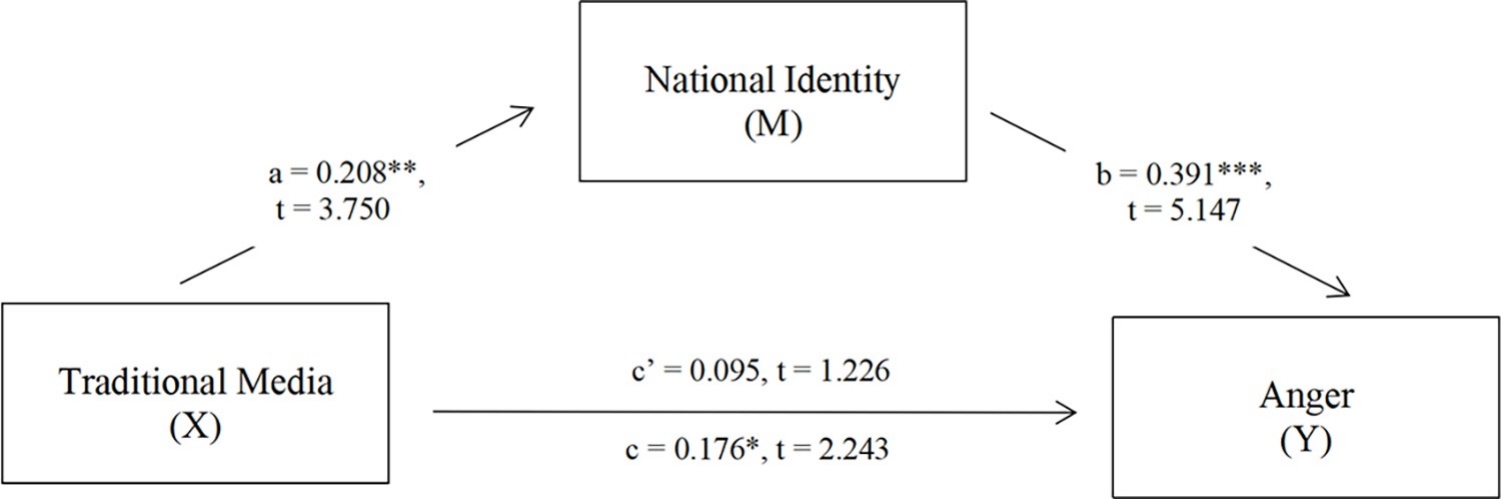
Mediation model. In this model, participants’ self-reported exposure to traditional media as a source of information about anti-Chinese discrimination was positively associated with national identity and national identity was positively associated with anger towards this information. The “effect” of traditional media use on anger was fully mediated by national identity (N = 326). ***p<0.001, **p<0.01, *p<0.5; Traditional Media (1 = Yes; 0 = No).

To test model viability, we first conducted two separate two-step hierarchical regressions to “predict” anger based on the participants’ self-reported exposure to traditional media and SNS news by controlling for gender, age, and education (S5, S6 Tables in S1 File). We found a significant result regressing traditional media on anger (*F*(4,321) = 1.422, *p* = .026, *ΔR*^*2*^ = .015), and a marginally significant regression of SNS news on anger (*F*(4,321) = 1.110, *p* = .053, *ΔR*^*2*^ = .012). To be parsimonious, we then adopted traditional media as a the predictor (*X*) of anger (*Y*) in exploring the potential mediating role of national identity (*M*), as was proposed in RQ 1. We adopted Andrew F. Hayes’ [126] Process Model 4 (version 3.5) in SPSS with 5,000 bootstrapped samples. Gender, age, and education were included as covariates. As shown in Fig 1, the total effect of traditional media (*X*) on anger was significant (c = .176, *p* = .026), whereas its direct effect on anger was non-significant (c’ = .095, *p* = .221) when national identity was included in the model. Therefore, national identity fully mediated the relationship between traditional media and anger and explained 45.5% of the total “effect”.

As much as this statistical attempt appeared to be effective, we consider these results to be illuminating only on a narrative basis, since traditional media was measured via “yes” or “no” responses. In order to confirm that relevant mechanism truly existed, we further utilized host newspaper coverage (*X*) as a proxy for host traditional media in testing its effect on anger (*Y*) with national identity (*M*) as the mediator, as was hypothesized in Q2. Ethnic newspaper coverage was not considered for this analysis because it was not correlated with anger or national identity in the previous results. A three-step hierarchical regression was conducted to predict anger based on host newspaper coverage and national identity after controlling for gender, age, and education (Table 2). We found that newspaper coverage (*F*(4, 321) = 1.365, *p* = .029, *ΔR*^*2*^ = .015) had a significant effect on anger, and when national identity (*F*(5, 320) = 6.789, *p* < 0.001, *ΔR*^*2*^ = .079) was added to the model, the effect of host newspaper coverage on anger became non-significant (*β* = .089, *p* = .101).

**Table 2:** Hierarchical regression of host newspaper coverage on anger and national identity.

N = 326	step 1		step 2		step 3	
DV: Anger	beta	p	beta	p	beta	p
Gender	-.03	.65	-.03	.57	-.04	.49
Age	.00	.96	.02	.76	.01	.87
Education	-.04	.52	-.05	.37	-.05	.44
Host Newspaper Coverage			.12	.03*	.09	.10
National Identity					.28	.00***
**model statistics**						
*F (df)*	.22 (3,322)		1.37 (4, 321)		6.79 (5, 320)	
*R* ^*2*^	.00		.02		.10	
*ΔR* ^*2*^	.00		.02		.08	

**Fig 2:**
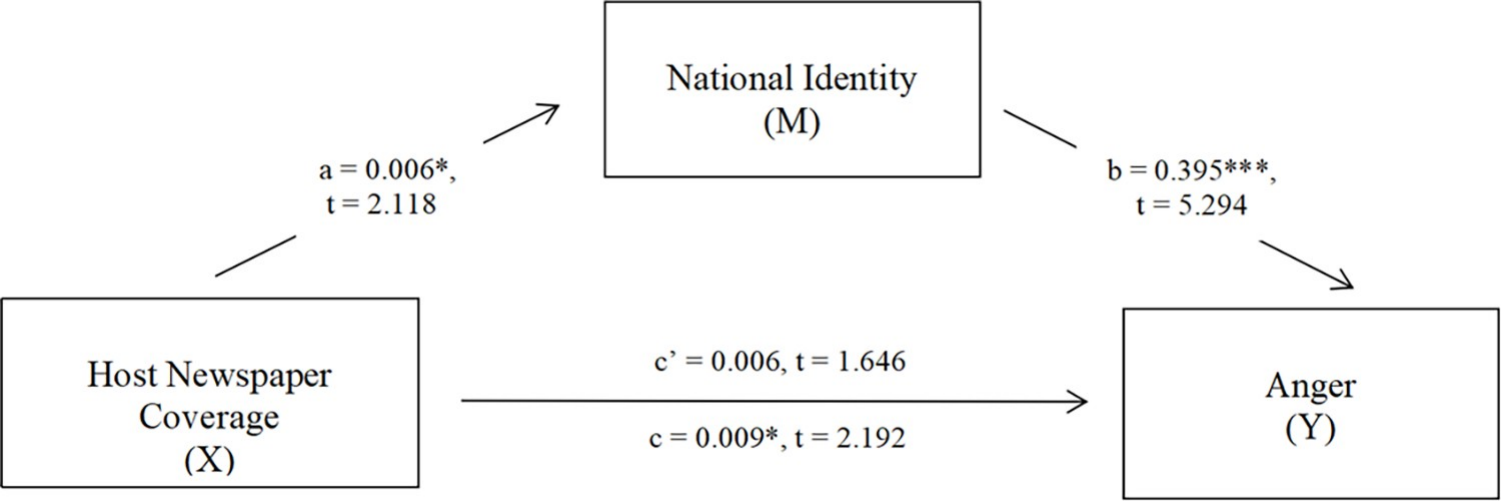
Mediation model. In this model, host newspaper coverage on anti-Chinese discrimination was positively associated with national identity and national identity was positively associated with anger towards this information. The effect of host newspaper coverage on anger was fully mediated by national identity (N = 326). ***p<0.001, **p<0.01, *p<0.5.

Next, we adopted the same mediation model procedure (Process Model 4 with bootstrap 5,000) to test the effect of national identity as a mediator. After controlling gender, age, and education as covariates, we tested the total effect of newspaper coverage (*X*) on anger (*Y*), and the mediation of national identity (*M*). As shown in Fig 2, host newspaper coverage (*X*) significantly predicted national identity (*M*) (a = .006, *t*(321) = 2.118, *p* = .035). The total effect of host newspaper coverage (*X*) on anger was significant (c = .009, *t*(321) = 2.192, *p* = .029), but not its direct effect on anger (c’ = .007, t(320) = 1.646, *p* = .101) when national identity was included in the model. Therefore, national identity fully mediated the relationship between host newspaper coverage and anger and explains 26.88% of the total effect. This mediation effect hence confirmed our assumptions generated earlier when traditional media was used as a predictor. These similar results allowed us confidence that host traditional media was a key source of exposure that induced anger among the Chinese, a process mediated by national identity.

### Model extension

**Fig 3:**
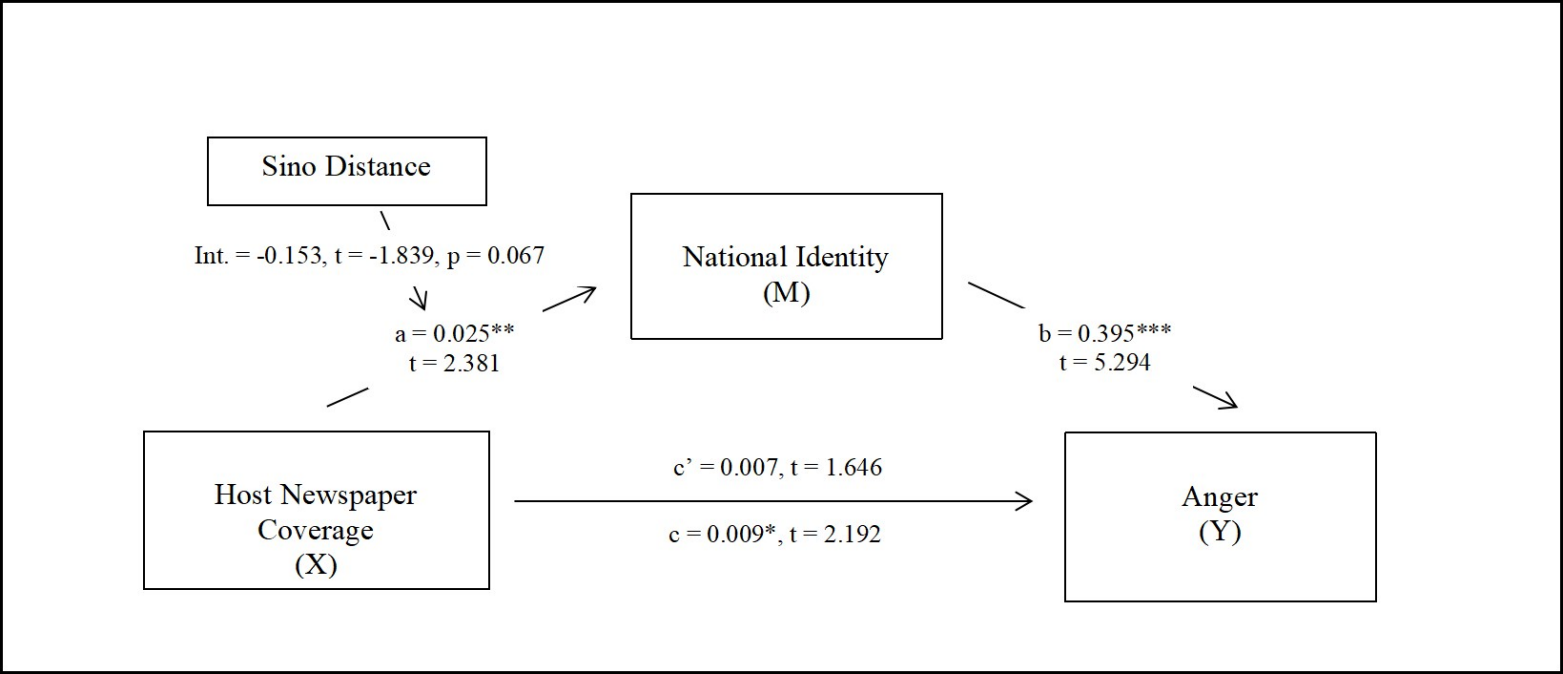
Moderated mediation model. Sino distance interacted with host newspaper coverage on anti-Chinese discrimination to an almost significant level in moderating national identity among the Chinese (N = 326).

**Fig 4:**
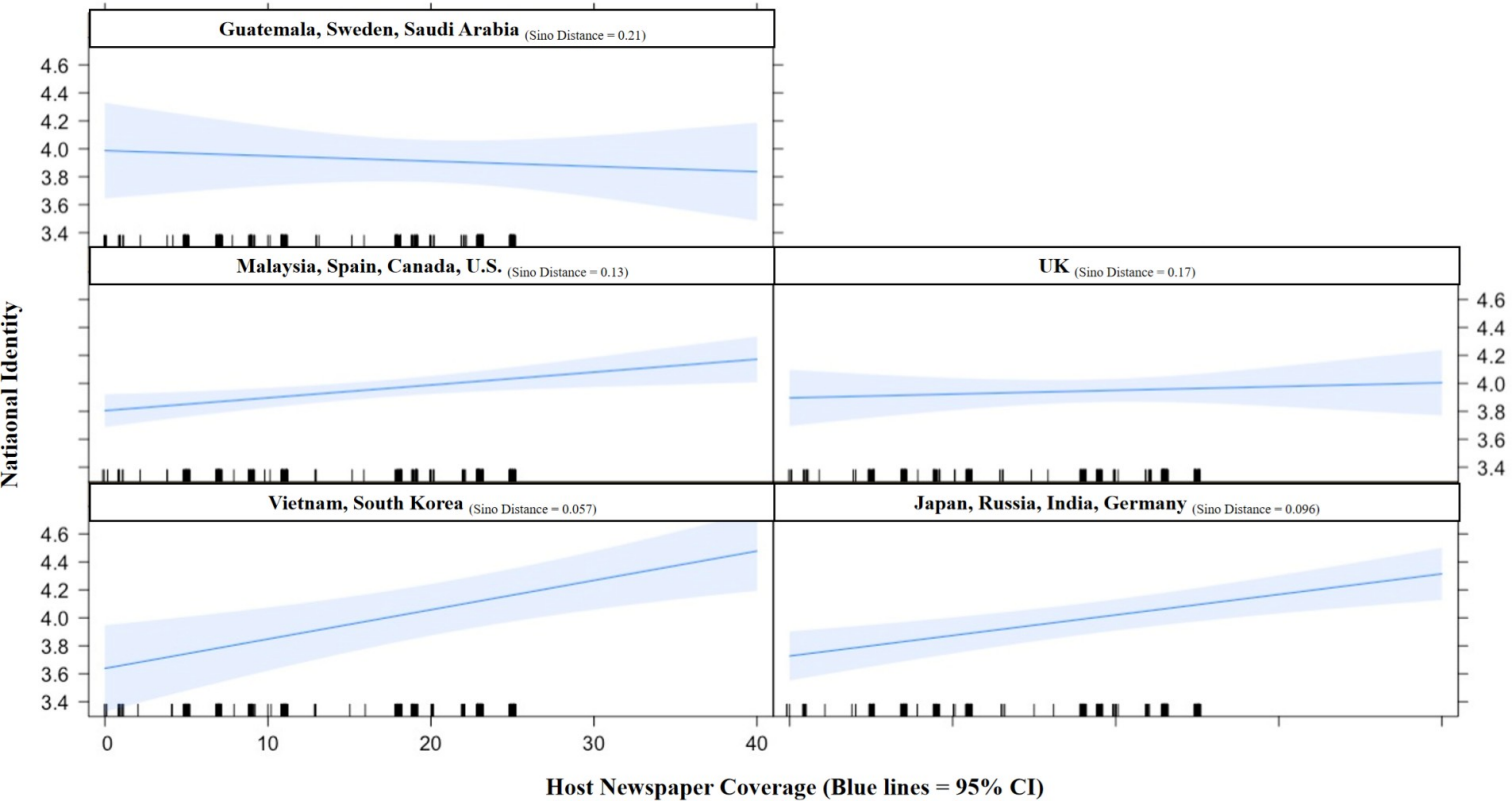
Plotting moderation. When controlling for Sino Favoritism, Sino Distance negatively and significantly interacted with host newspaper coverage in predicting national identity. As Sino Distance and host newspaper coverage both increased, the increase in national identity became moderate; in countries most culturally away from China, an increase in host newspaper coverage was related to a decrease in national identity (N = 326).

For RQ 3, we explored how Sino Distance interacted with host newspaper coverage in moderating the strength of national identity among our Chinese participants in different countries. Accordingly, we adopted Andrew Hayes’ [126] Process Model 7 (version 3.5) in SPSS with 5,000 bootstrapped samples, with host newspaper coverage (*X*) as the predictor, anger (*Y*) as the outcome, and national identity (*M*) as the mediator. As shown in Fig 3, when Sino Distance was in the model, its interaction with newspaper coverage (*X*) approached significance in predicting less national identity (Interaction = -.153, *t* [319] = -1.839, *p =* .067), while the direct effect of newspaper coverage on national identity remained significant (a = .025, *t* [319] = 2.397, *p* = .017) and the mediation model remained robust. Because this interaction effect was not ideal, a post-hoc examination on the interference of pre-pandemic favoritism toward China was performed, given its significant correlations with national identity, host newspaper coverage, and Sino distance (Table 1). We further controlled for Sino Favoritism in the model, which showed a significant interaction (Interaction = -.161, *t* [318] = -1.984, *p* = .048) with valid total effect (*c* = .012, *t* [320] = 2.633, *p* = .009) and indirect effect (Effect = .004, 95% C.I. [.002, .007]) (S16 Fig in S1 File). We then used R to plot the moderation by demonstrating five sections of Sino Distance and their interactions with host newspaper coverage. We marked several representative countries in each Sino Distance section (e.g., Vietnam being the most culturally close and Saudi Arabia the most culturally distant from China). As shown in Fig 4, after controlling for pre-pandemic Sino Favoritism, the interaction of Host Newspaper Coverage and Sino Distance negatively affected national identity. For participants in countries that were culturally closer to China, an increase of host newspaper coverage on anti-Chinese discrimination was associated with higher national identity. However, examining the host countries rated as increasingly culturally distant, higher levels of host newspaper coverage became unrelated to national identity. These results and other findings are discussed below.

## Discussion

Synthesizing results from our analyses in addressing the three research questions, we found that: (1) During COVID-19, overseas Chinese migrants were aware of discrimination against co-nationals from several sources of information (see S3 Fig in S1 File for detail), whereas only the tendency to report learning discrimination messages from traditional media was correlated with anger and national identity. The tendency to report SNS News as a source of information also revealed similar patterns to a marginally significant level (S6 Table, S4 Fig in S1 File). (2) On this narrative basis, by measuring both host and ethnic newspaper coverage in each host country, we found that only host newspaper coverage functioned as a proxy for traditional media exposure to anti-Chinese discrimination in predicting anger among the Chinese, a process fully accounted for by national identity. (3) Our inquiry into the proximal factor of Sino distance by controlling Sino favoritism as a covariate also revealed its nuanced interaction with host newspaper coverage in moderating national identity. As host newspaper coverage on anti-Chinese discrimination increased, the Chinese members displayed an increase in national identity in culturally closer countries, a moderate increase in culturally neutral countries, and a decrease in culturally farther countries. We now discuss the theoretical and empirical implications of these findings.

### Migrants in crisis: The (un)imagined community

This study examined collective identity and emotions among the Chinese migrants (i.e. students, residents, and workers) worldwide within an ecological framework during the early period of the coronavirus outbreak (April, 2020). We regard the global socio-political context of anti-Chinese sentiment and discriminatory incidents towards the Chinese as a macro-level factor in the cultural and media ecology of transnational migrant living. It is possible that during this unprecedented crisis, not only have Chinese been exposed in a global climate of ethnic or race-linked attitudes or conflicts in their day-to-day intercultural contact [127], but host media coverage of discrimination also set an agenda of racial issues that underscored intergroup threats [128]. Our findings offer an opportunity to understand the processes of information exposure, national identity and vicarious sharing of emotions among the Chinese in ways that transcend national boundaries.

Whilst previous research focused on how consumption of home and ethnic media fosters a sense of cultural belonging [39, 129], our study compared host and ethnic media and validated the role of host media in cultivating the Chinese migrants’ collective identity during intergroup conflicts based on their sense of nationhood, echoing previous research that explored how a shift-away from ethnic media may still cultivate ethnic identity [130]. By taking an alternative path from research on diasporic identity as an idealized form of belonging [131, 132, p.2], we replenished diasporic studies by focusing on a rather understudied demographic in this population and approached identity from a social psychological perspective. Specifically, the sudden occurrence of COVID-19 has allowed reexamination of the psychological nature of migrants’ collective identity in a context-dependent, time-specific situation that captured intergroup contact beyond transnational variations. Hence, we argue that during a global crisis, the imagined community of a cultural group in migration is no longer a hypothetical, idealized existence, but actually exists in the minds of its members with realistic psychological consequences.

In that regard, our findings have confirmed theories in intergroup emotions [79], with the implication that shared appraisals of events not only trigger shared emotions within culture [133] but also shared consciousness that a particular emotion is being experienced in prototypical emotional episodes [134]. Our study contributed to similar research on cultures bonded with a strong sense of identity when facing group threats, such as threat perception and out-group attitudes among Israeli Jews [135] and collective reaction of Chinese in the face of extreme events [136].

However, one unanswered question is whether collective anger triggered by discrimination content in the media is due to group-based appraisal of threats to the Chinese collective identity, or whether the expression of anger is endorsed by cultural values and norms [88, 137] in these situations (or both). There are reasons to believe that if the appraisal of group discrimination is powerful enough to trigger anger among the Chinese migrants scattered across the world, cultural value is as likely at play as identity. Our finding that these Chinese abroad experienced anger in relation to their sense of national identity infers that group appraisals are oriented towards home culture identification when intergroup conflict cues are underscored in the host context. This is supported by our post-hoc factor analysis showing anger as an independent emotional component to be correlated with national identity (S3, S4 Tables in S1 File). This finding is consistent with robust past research that minority groups perceived more discrimination directed at their group (collective identity) compared to themselves (self-identities) being personally discriminated [138], explaining in part why our test of individual-level perceived discrimination did not produce clear results during our preliminary research (see S1 File).

There may be other issues that need to be addressed. How much do individual emotions represent group emotions? How do emotions towards the same event differ on the individual level (such as experience of personal discrimination) and on the group level (such as learning about collective discrimination)? Will appraisals of actual events (such as real-life experience of discrimination) and imagined events (such as visualizing discrimination against oneself or one’s group) lead to different emotions? These are questions not adequately addressed in the current study, but are being answered with ongoing research [104, 105, 139]. Exploring culturally fostered emotions from an ecological perspective will benefit future research and tap into the nuances of cultural emotional patterns in a globalized world. For example, future research could examine collective emotions among the Chinese diaspora by situating emotions in both individual and global contexts, not just during a global crisis.

### Media exposure and migrant identity

While previous research investigated both the direct and indirect effect of shared social identity on collective feelings [140], our study demonstrated the indirect effect of national identity in the relationship between host media coverage on inter-group threat events and anger among a sample of geographically separated transnational Chinese migrant members. From a narrative perspective, the enactment of national identity via information exposure to group threats was significant when individuals reported the tendency to receive such information from traditional media and SNS news, but not from the less credible sources of anecdotes and vicarious observations in the online and social environments. Although fake news and human propagated untrue messages on social media are proven to be capable of exacerbating people’s perception [141], we propose that in our research context, there might be a medium effect in the crisis communication [142] of anti-Chinese discrimination. Accordingly, our empirical examination of host newspaper coverage as a predictor of national identity and anger implied the agenda setting role of traditional news media in identity confirmation among cultural migrants in a threat context. This implication echoes recent findings that collective media agenda still has the power to garner people’s attention in a high-choice media environment [143], and that identity and anger mediated the relationship between online alternative news and protest intention, but not social media news and protest intention in a mass movement [144].

Still, our yet unchecked process of social network communication in relation to national identity and anger suggests future research potential. It is possible that interpersonal and intergroup communication on social network offered a chance to reduce intergroup prejudice [145]) and stigma [146], hence alleviating the agenda-setting effect of traditional media on identity and negative emotions towards the outgroup by potentially diversifying collective identities [147]. Further studies could focus on the nuanced pathways of social media content and interactions among users to explore how individuals regulate their identities and emotions by giving personal meanings to group-threat events. Research could explore the structure and content of such messages for their “framing” and “melting” effects on identity and emotions via a multiplicity of research methods [148]. Research could also investigate how media consumption of discrimination content is related to group coping strategies, emotional suppression, and post-traumatic growth, adopting approaches similar to research already studied on the individual level [149, 150]. We also suggest future research grounded in a similar context to focus on the dynamic transactional process of selective media exposure in facilitating social identity processes [151] and the potential “reinforcing spiral” of media’s effect on social identity [152].

Finally, we also failed to identify a relevant variable focused on Chinese home media exposure among the participants in both of our exploratory and confirmatory studies. Due to the impossibility to post-hoc identify the variance among our participants in terms of their exposure to Chinese home media, we could not similarly effectively source this potential component of media exposure, which limited the scope of our findings. Although not initially collecting self-reports for Chinese home media exposure was a disadvantage, we argue that our objective measure of exposure to host and ethnic media still offered a reliable empirical support for our findings. We suggest that future research aim to analyze media exposure comparatively across the host, ethnic, and home trajectories to fully unpack the processes of media and identity during similar global coverage. Future research could also identify the role of migrants’ social networks with home cultural members in disseminating inter-group information and reinforcing in-group identities in a time of global crisis.

### Sino distance: A nuanced understanding

As our study confirmed the role of national identity in mediating the process between traditional media (host newspaper) coverage of anti-Chinese discrimination and anger among transnational Chinese migrants, a further examination of Sino Distance as a moderator revealed a nuanced understanding of how national identity may vary among mono-cultural members residing in different countries. Our findings suggest that for people in culturally close countries, such as Vietnam, Japan, and Germany, the increase of host news coverage on discrimination was related to a rise in national identity. In fact, within this range of Sino Distance (SDis = .057 ~ .118), countries of smaller cultural distance demonstrated a sharper upward trend for national identity among the Chinese as host newspaper coverage increased, suggesting that people in culturally similar countries enhanced their national identity when there were more reports about anti-Chinese discrimination. For participants in countries that are moderately distanced from China, such as Malaysia, Spain, USA, and UK (SDis = .124 ~ .172), an increase in news coverage was mildly related to a rise in national identity. However, for participants in culturally distant countries, such as Guatemala, Sweden, and Saudi Arabia, the increase of news coverage seemingly reduced national identity among the Chinese. Within this range of Sino Distance (SDis = .192 ~ .212), countries of greater cultural distance demonstrated a downward trend for national identity among the Chinese, suggesting that people in the most culturally different countries experienced a decrease in national identity when the number of reports of anti-Chinese discrimination increased in the host country. To help understand these findings given the potentiality of direct causal links, we found that Sino distance did not predict national identity (*β* = —.090, *p* = .110), nor host newspaper coverage (*β* = .032, *p* = .569).

This nuanced interaction could be indicative of country-level differences in news reporting and coverage. For instance, research has found that in climate change coverage, the Filipino press is more likely to represent collectivism instead of individualism than Western journalism [153], and that despite globalized journalistic standards, country-level reporting is also affected by local contexts, such as cultural and institutional settings [154]. It is to be noted that we achieved a significant moderation of Sino Distance by controlling for Sino Favoritism, the host country’s pre-pandemic favorable attitude toward China, which was negatively predicted by Sino distance (*β =* -.129, *p =* .019) and negatively predicted both host newspaper coverage (*β =* -.323, *p* = .000) and ethnic newspaper coverage (*β =* -.358, *p* = .000). Therefore, an important question to consider is whether different countries’ news coverage on a global story concerning a target country was a result of bilateral relations and cultural affinity, such that countries more favorable towards China had fewer discriminatory incidents targeting the Chinese community and hence less coverage, whereas shared cultural values (such as collectivism) in culturally close countries would allow host coverage to have greater impact on the Chinese sojourners’ national identity.

Part of the efficacy of our study is that there seems to be a media agenda-setting role that stigmatized “the Chinese” when the outbreak of the virus was reported in China. Notably, our news sourcing revealed that on January 30–31, 2020, there was a surge/onset of global reports about anti-Chinese sentiments and xenophobia (see S9 Fig and S13 Table in S1 File). In the same time window, WHO announced the coronavirus a global emergency [155] and the U.S. imposed a travel ban on people who had been to Wuhan in the past 14 days [156], followed by Western media reports naming COVID-19 as the “Chinese virus” or “Wuhan virus” [157, 158] even though the WHO and other responsible authorities urged that the virus not be associated with a place or cultural group [159]. Given this unique surge of reports, we then conducted a post-hoc analysis by specifically counting the number of host reports on January 30 and 31, 2020 in each country as very likely a result of the “Chinese virus” effect on anti-Chinese sentiment. Further correlation and regression results showed that Sino distance significantly and negatively predicted host news coverage during January 30 and 31 (*β* = —.019, *p* < .001), meaning that culturally close countries had been more focused on reporting anti-Chinese discrimination than culturally distant countries did when it came to racial stereotyping. This finding could perhaps also explain why an increase of reports enhanced national identity more among participants in culturally closer countries given shared racial identity among culturally close countries.

However, further research is in need to examine why an increase of host news coverage on anti-Chinese discrimination did not enhance national identity among the Chinese in culturally distant countries. Whilst more coverage would mean more discriminatory incidents, they might also counter the negative effect of realistic intergroup conflicts in culturally distant countries in a way that these Chinese minorities feel cared about in the national press.

### Strengths and limitations

This research conducted a cross-sectional design to test how traditional media exposure to discrimination towards co-nationals could enhance national identity and elicit anger among transnational Chinese migrants. We utilized self-reported data as a narrative guidance and objective online data to explore relevant models. The contribution of this research is its ecological approach to consider vicarious identity and emotions among the Chinese during the early days of the Coronavirus outbreak worldwide. As an early study (data collected in April 2020), some significant limitations need to be noted.

The first weakness is that at that time we only relied on correlational cross-sectional data and non-random sampling. This limits our ability to thoroughly analyze and understand causal relationships between individual-level media exposure and anger. Secondly, we did not successfully measure and compare perceived discrimination on both individual and collective levels to shed light on the nature of vicarious discrimination explored in our research. By asking participants about their awareness of anti-Chinese discrimination, it is possible that this articulation had a priming effect on participants, whereas the more suitable method could be to conduct a quasi-experiment with a control group or combined follow-up surveys or interviews, although with the rapid break of the pandemic, developing such designs appeared out of reach, especially for a diasporic sample. Besides, the complex finding of the interaction effect might be due to timing as the pandemic might have impacted these groups of people at different times. Only a longitudinal study would be able to capture the changes in identity and anger due to host or ethnic news coverage. We caution researchers when considering this result. Finally, our sample only captured a fraction of the rather diverse Chinese diaspora. The purpose of their migration (international education or labor) itself during or before COVID-19 suggests this group’s further tendency for repatriation and affiliation with the home country. Our research results might not be generalizable to other demographics of the overseas Chinese population.

Despite these limitations, care has been taken with this design during the early stage of the global COVID-19 pandemic. The data gathered from our diasporic sample and objective sources, along with the integration of media and psychological approaches to collective identity, provide some degree of confidence in the theories and method used, robustness of analysis, and findings. Obviously, the diverse policies of COVID-19 lockdowns and impacts of social distancing in different settings have played a pivotal role in this study such that the results are likely not fully replicable in non-pandemic times. However, the study shows how certain diasporic groups might unite during challenging times, and why or why they may not “fit in” during those periods in host climate that has emphasized integration as probably the best approach to acculturate.

## Conclusion

Findings in this study reveal how traditional media exposure, especially host newspaper coverage on anti-Chinese discrimination in foreign countries tends to broadly arouse anger among Chinese migrant members via national identity. However, this process is moderated complexly by whether the participants were culturally close to, or distant from the host country. Understanding how variant ecological factors work on collective emotions, especially that of anger, through factors that impact ethnic identity offers implications for future research into similar diasporic and cultural psychology research.

## Supporting information

S1 File (ZIP)
