# Supplementary material for: Anger among Chinese migrants amid COVID-19 discrimination: The role of host news coverage, cultural distance, and national identity
Source: PLoS One. 2021 Nov 16;16(11):e0259866. doi: 10.1371/journal.pone.0259866 (PMC8594835; doi:10.1371/journal.pone.0259866)
Supplement: S1 File — (ZIP) [file pone.0259866.s001.zip › S File.docx]

Supplementary Material

# Supplementary data

In our main study, we tested the relationship between traditional media exposure to anti-Chinese discrimination and anger among the Chinese migrants – a process mediated by national identity. In this supplementary document, we provide further analyses to explain the rationale of our study by discussing alternative factors and models not properly addressed in the main study. We also showcase why studying media exposure, group anger, and national identity in the paths examined in our study provides the most conclusive explanation .

# Participant recruitment

In this study, we recruited Chinese participants from 33 countries by posting our survey link on Chinese social media platforms (Weibo and WeChat) primarily through the researchers’ and assistants’ network for a pilot test. After the pilot test, we used snowball sampling by asking participants to share the survey with their Chinese community with a random lottery bonus of around 5-15 RMB per person. Anyone who was studying or living outside China during the COVID-19 outbreak was solicited to take the survey. We did not make country-specific requirements on the participants. After data collection, we screened participants with several demographic indicators. For example, we asked respondents whether they were abroad during the early COVID-19 outbreak, in which country they were, the type of visa they held, and whether they had repatriated to China recently. Respondents who were residing in China but filled out the survey were removed (as they reported “always in China during COVID”). By asking participants what national passports they held, we further took out participants who reported being foreign nationals. This is because keeping them would cause validity concerns when implementing our national identity scale. After coding our two continuous variables, national identity and anger, we removed outliers in the sample using the Mahalanobis Distances method. An initial sample of 345 was collected, 14 were excluded as non-Chinese nationals, another five as outliers. Among 326 of the finalized sample, 57 (17.5%) were recent repatriates by May 10, 2020. Average time abroad was 40.40 months (*SD* = 42.20). **S1** **Fig.** shows where those Chinese expatriates (had) sojourned. We color coded them by West/Non-West as a reference since we also measured Sino distance in our main study. Western countries include those in North America (including Mexico [*n* =1]), Europe, and Australia, non-Western countries include those in South America, Asia, and Africa.

# Repatriates do not confound the study

In order to determine whether having repatriates in our sample would confound major hypotheses tests, we conducted a series of independent samples t-tests by grouping repatriates (*N* = 57, 17.5%)/non-repatriates (*N* = 269, 82.5%) and tested whether these two subgroups scored differently on major variables. As shown in **S1** **Table**, repatriates and non-repatriates did not score differently across the major variables in our exploratory study - their tendencies to select traditional media and SNS news, anger, as well as national identity (*df* = 324, p >.05), thus were put together as a homogenous sample for further analyses. Notably, repatriates and non-repatriates demonstrated variances in their tendency to choose SNS anecdotes and “self-experience” as to how they became aware of anti-Chinese discrimination. In both cases, repatriates (*M*_SNS Anecdotes_ = .68, *p* = .009; *M*_self-experience_ = .39, *p* = .011) scored higher than non-repatriates (*M*_SNS Anecdotes_ = .61, *M*_self-experience_ = .28), suggesting that repatriates self-reported greater exposure to social media propagated anecdotes about discrimination and higher instances of direct discrimination experiences. We caution readers about these results, as repats represent only a small fraction of the sample and we cannot draw any reasonable conclusions about this sub-sample.

# Why anger?

In a separate exploratory analysis, we examined whether anger was the predominant emotion among the Chinese towards hypothetical discrimination in a “being abroad” scenario. Participants were asked to choose how they would feel if they were discriminated during COVID-19 in a foreign country from a list of emotions used in a similar exploratory study on African Americans [1]. Among 11 types of emotions, anger was predominately the most prevalent response in our sample (70.9%), followed by sadness, powerlessness, frustration, and scare (**S2** **Fig.**). By coding the responses as binary variables (Yes =1, No = 0), we retrieved 11 emotion variables and further conducted a principal component analysis based on Eignevalue > 1 and varimax rotation to examine whether these emotions could be reduced into several factors.

As shown in **S2** **Table**, the 11 emotions were reduced to four components, which we accordingly named “Inferiority” (powerless, hopeless, scared, vulnerable, and inferior), “Sadness” (frustrated, sad), “Shame” (humiliated, vengeful), and “Anger.” As a result, Anger stood alone as a unique factor without correlation with any other emotion. **S3** **Table** shows the correlations of these four emotional components with the major variables in our study. Only Anger was positively correlated with national identity (*ρ =* .138*, p =* .012), and only Sadness was positively correlated with Sino distance (*ρ =* .120*, p =* .030) to a significant level. Besides, Anger was negatively correlated with weakness (*ρ = -*.140*, p =* .011) and sadness (*ρ = -*.158*, p =* .007). Shame was also negatively correlated with Weakness (*ρ =* -.478, *p <* .001) and Sadness (*ρ = -*.196*, p <* .001). These results suggest that emotions with negative action tendencies, such as anger and shame, are significantly different from passive emotions - mostly notably sadness that could have been more prevalent among participants in culturally distant countries. A further regression analysis also revealed that national identity predicated anger even in this hypothetical scenario (*β* = .118, *p* = .034). In conclusion, these findings may support the notion that when emotions are “tied to social contexts, the distinction between the individual and the group blurs” [2, p.8]. As our study examines the mechanism of collective emotions, anger is justified as the major outcome variable based on this series of analyses.

# Other sources of information/media exposure

Another important question is which information source activates certain emotions. Here, we extend our exploratory analyses from the main study. As we previously asked, “How do you know about discrimination against Chinese abroad during COVID-19?”, participants selected from a list of the following items: 1. TV and (online) newspaper reports (Traditional Media); 2. News on social networking sites (SNS News); 3. Anecdotes on social networking sites (SNS Anecdotes); 4. Acquaintances; 5. Self-experience; 6. Others; and 7. “I do not know.” Each item is dummy coded into a dichotomous variable (Yes = 1; No = 0). For example, if participants selected “Self-experience”, then the variable is coded as Self-experience = 1. **S4** **Table** shows that other sources of information were not significantly related to anger or national identity. However, reading anecdotes about discrimination on social media was highly correlated with “acquaintances” (i.e. knowing Chinese co-nationals who had been discriminated) (*ρ* = .351, *p* < .001), and mildly correlated with “self-experience” (*ρ* = .114, *p* = .040). Besides, “acquaintances” and “self-experience” were highly correlated (*ρ* = .426, *p* < .001). For descriptive statistics, 48.2% of participants reported personally knowing co-nationals who had been discriminated, and 30.1% reported having been discriminated themselves, while another 2.5% were not aware of discrimination against Chinese (**S3** **Fig.**). This self-reported discrimination seems to match a lot of the news stories that were documenting Asian hate during the early days of the pandemic (**S7** **Table**).

# Regression analyses

As discussed in the main text, before we tested the mediation effect of national identity in PROCESS, we ran a series of hierarchical regressions to examine whether the participants’ tendency to use Traditional Media and SNS News as an information source would impact their anger and national identity. In **S5** **Table** and **S6** **Table**, we regressed Traditional Media Use and SNS News respectively on anger, and found traditional media (step 2, *F*(4,321) = 1.422, *p* = .026, *ΔR^2^* = .015) to be a stronger predictor than SNS News (step 2, *F*(4,321) = 1.110, *p* = .053, *ΔR^2^* = .012). Given that SNS News has a marginal effect on anger (*p* = 0.53), putting it into the mediation model (Andrew Hayes’ Model 4, 2018, version 3.5) with national identity as the hypothesized mediator does not alter its total effect, even though the path from SNS News Use to National Identity (a = 0.29, *p* < .001) and National Identity to Anger (b = 0.40, *p* < .001) were both significant (**S4 Fig.**).

# Host social support as a proximal factor

Research has shown that host social support could alleviate acculturative stress [3] and benefit sojourners’ psychological well-being and adjustment [4, 5]. It is reasonable to believe that although some hosts may exercise racial discrimination, other hosts may offer moral and emotional support. This proposal is based on past findings of the relationships among host social support, ethnic identification, and mental health in the acculturation literature [6, 7]. For example, host social support alleviates the negative effect of perceived discrimination on well-being [8]. Therefore, we adopted host social support as a proximal factor for intercultural contact among our Chinese sample to test its effect on anger and the mediation of national identity.

Host social support was measured by the adapted Multidimensional Scale of Perceived Social Support (MSPSS, [9]). It is a four-item scale measuring the quality of social support received by Chinese participants in their host countries. Participants were asked to rate on a 5-point Likert scale from “strongly disagree” to “strongly agree” concerning the following statements: “When things go bad, I can count on my host friends,” “I have host friends who can share my happiness and sadness,” “I can talk to my host friends about my problems,” and “My host friends try their best to help me.” Higher scores suggest higher social support in the host country. The scale is internally reliable (Cronbach’s α = .884). Participants scored a mean of 3.17 with a range from 2.92 to 3.36, suggesting a moderate host social support level.

Correlation analyses show that host social support is negatively and strongly correlated with both anger (*r* = -.148, *p* = .007) and national identity (*r* = -.190, *p* = .001) (**S4** **Table**). We then ran the same mediation model (Andrew Hayes’ Model 4, 2018, version 3.5) with host social support as the predictor, anger as the outcome, and national identity as the mediator. As shown in **S5** **Fig.**, the total effect of host social support on anger is significant (c = -.118, *p* = .009). When national identity is in the model, its direct effect is not significant (c’ = -.076, *p* = .085). In this model, national identity explains 35.8% of the total effect as a full mediator but still has a positive effect on anger (b = .385, *p* < .001).

# The elephant in the room - perceived discrimination

During data collection, we also implemented two perceived discrimination measurements on our sample. We adopted the Everyday Discrimination Scale (EDS) [10] and its companion measure, the abbreviated Heightened Vigilance Scale (HVS, [11]). EDS is a nine-item scale measuring participants’ everyday experience with discrimination by asking, for example, how frequently they feel being treated with less courtesy than others or called names or insulted. HVS is a four-item scale measuring how vigilant respondents are in daily settings as a behavioral indicator of perceived discrimination. Respondents are asked to rate how often “You try to prepare for possible insults from other people before leaving home”, “Feel that you always have to be very careful about your appearance (to get good service or avoid being harassed)”, “Carefully watch what you say and how you say it”, and “Try to avoid certain social situations and places.” The scales were translated and back translated with expert review.

All items of EDS and HVS are anchored on a 6-point scale ranging from “very frequently” to “never” and reverse coded so that higher scores indicate higher perceived discrimination. Participants were primed to specifically report their experiences during COVID-19. Both EDS (Cronbach’s α = .912, M = 2.10, SD = 0.88) and HVS (Cronbach’s α = .808, M = 3.34, SD = 1.26) demonstrate strong internal reliability. By measuring perceived discrimination, we wanted to check whether this direct measure of perceived discrimination would have effect on national identity and anger, given that perceived discrimination is, of course, the most proximal indicator of discrimination. However, a preliminary correlation analyses revealed that everyday discrimination was not correlated with anger (*r* = -.050, *p* = .371) or national identity (*r* = .012, *p* = .829); neither was heightened vigilance (*r*_anger_ = -.026, *p* = .635; *r*_national identity_ = .020, *p* = .719). An independent samples t test comparing West vs. Non-West also did not show significantly different levels of perceived discrimination in our sample (Everyday Discrimination: *F*(2, 324) = .165, t = .006, *p* = .995; Heightened Vigilance: *F*(2, 324) = 4.822, t = .809, *p* = .419). We contend that due to social distancing and lockdowns during the period of our data collection, perhaps previous measurement of perceived discrimination made little sense to the respondents whey they were primed to think about daily contact with the hosts, thus we were not able to identify meaningful results relevant to perceived discrimination.

# Other alternative models

During our revision of the paper, we were also recommended by colleagues to test alternative models for potential empirical implications. Some readers may find them useful, thus we hereby offer the results of the alternative models we tested and explain why we are confident with our hypothesized models. These tests of alternative models were only relevant to our exploratory analysis of self-reported traditional media use, therefore we caution readers to interpret them with care.

1. Testing Anger as a mediator (Traditional Media → Anger → National Identity)

As shown in **S10 Fig.**, we tested the alternative hypothesis that anger could also mediate the relationship between traditional media exposure and national identity, and compared the results with our main model (**S11** **Table**). As a result, anger was a *partial* mediator in this model, compared to national identity being a full mediator in our main study. We did not report this finding in the main study because it is less relevant to our theoretical underpinning, but we agree that it might shed light on the explanatory power of the theories we adopted concerning intergroup emotions [2], which posits that the relation between anger and group identity could be reciprocal. However, we argue that having national identity as a mediator (Traditional Media → National Identity → Anger), as hypothesized and validated in the main study, is a better model fit based on the traditional mediation effect size measure P*_M_* (the ratio of the indirect effect to the total effect) [12]. Following this approach, we found that national identity as a mediator explains 46.02% of the relationship between traditional media and national identity, compared with anger accounting for 16.34% of the relationship between traditional media and national identity. Thus, we are confident that this comparative advantage serves as an empirical ground for our model in the main study.

1. Testing National Identity as a predictor (A. National Identity → Traditional Media → Anger; B. National Identity → Anger → Traditional Media)

As shown in **S11 Fig.** and **S12** **Fig**., we tested alternative mediation models with national identity as the predictor. As a result, neither one of the alternative models stood since there was no significant relationship between traditional media and anger in the mediation analysis.

1. Testing National Identity as a moderator

As shown in **S13 Fig.**, testing national identity as a moderator in the relationship between traditional media and anger in Study 1, and that between newspaper coverage and anger in Study, did not produce significant results.

1. Testing paths using SEM with National Identity as a mediator

**Direct effect**

Our results (**S14** **Fig.**) reveal that both Host Newspaper Coverage and Sino Favoritism positively predicted national identity (*β* = 0.200, SE = 0.003; *p* <0.001; *β* = 0.489, SE = 0.111; *p* <0.001). Meanwhile, national identity positively predict anger (*β* = 0.294, SE = 0.074; *p* <0.001).

**Indirect effect**

Host Newspaper Coverage was indirectly and positively associated with anger (*β* = 0.059, *p* <0.001, CI 95% [0.024,0.108]).

Sino Favoritism was indirectly and positively linked with anger (*β* = 0.075, *p* < 0.001, CI 95% [0.040 ,0.123]).

1. Testing paths using SEM with Anger as a mediator

As shown in **S15** **Fig.**, RMSWA > 0.10 indicates a poor model fit [13]. Therefore, Anger does not mediate the relationship between Host Newspaper Coverage and National Identity.

# References

1. Mackie DM, Smith ER. Group-based emotion in group processes and intergroup relations. Group Process Intergr Relat. 2017;20: 658-668. [doi: 10.1177/1368430217702725](https://doi.org/10.1177/1368430217702725).
2. Williams DR, John DA, Oyserman D, Sonnega J, Mohammed SA, et al. Research on discrimination and health: An exploratory study of unresolved conceptual and measurement issues. Am J Public Health. 2012;102: 975-978. doi: 10.2105/AJPH.2012.300702.
3. Poyrazli S, Kavanaugh PR, Baker A, Al-Timimi N. Social support and demographic correlates of acculturative stress in international students. J Coll Couns. 2004;7: 73-82. doi: 10.1002/j.2161-1882.2004.tb00261.x.
4. Bender M, van Osch Y, Sleegers W, Ye M. Social support benefits psychological adjustment of international students: Evidence from a meta-analysis. J Cross-Cult Psychol. 2019;50: 827-847. doi: 10.1177/0022022119861151.
5. English AS, Zhang YB, Tong R. Social support and cultural distance: Sojourners’ experience in China. Int J Intercult Relat. 2021;80: 349-358. doi: 10.1016/j.ijintrel.2020.10.006.
6. Berry JW. Contexts of acculturation. In Sam DL, Berry JW, editors. Cambridge handbook of acculturation psychology. New York (NY): Cambridge University Press; 2006. pp. 27-42.
7. Schwartz SJ, Unger JB, Zamboanga BL, Szapocznik J. Rethinking the concept of acculturation: implications for theory and research. Am Psychol. 2010;65: 237-251. doi: 10.1037/a0019330.
8. Jasinskaja-Lahti I, Liebkind K, Jaakkola M, Reuter A. Perceived discrimination, social support networks, and psychological well-being among three immigrant groups. J Cross-Cult Psychol. 2006;37: 293-311. doi: 10.1177/0022022106286925.
9. Tonsing K, Zimet G, Tse S. Assessing social support among South Asians: The multidimensional scale of perceived social support. Asian J Psychiatry 2012;5: 164-168. doi: 10.1016/j.ajp.2012.02.012.
10. Williams DR, Yan Y, Jackson JS, Anderson NB. Racial differences in physical and mental health: Socioeconomic status, stress, and discrimination. J Health Psychol*.* 1997;2: 335-351. doi: [10.1177/135910539700200305](https://doi.org/10.1177/135910539700200305).
11. Williams DR. Measuring discrimination resource. 2016. Available from: <https://scholar.harvard.edu/davidrwilliams/node/32777>
12. Wen Z, Fan X. Monotonicity of effect sizes: Questioning kappa-squared as mediation effect size measure, PubMed. 2015;10: 193-203. doi: 10.1037/met0000029
13. Browne MW, Cudeck R. Alternative ways of assessing model fit. In Bollen KA, Long JS, editors. Testing structural equation models (pp. 136-162). Newsbury Park (CA): Sage; 1993. pp. 136-162.


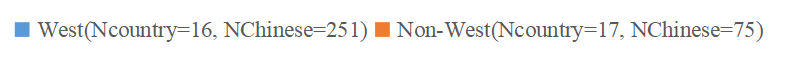


**Figure S1.** We plotted where the Chinese migrants were sojourning by country. As most of them were lone individuals in “non-dominant” countries, dichotomizing them by West/Non-West category allowed a better visualization of the distribution of our sample.

**Figure S2.** We explored participants’ potential emotional responses by having them think about being discriminated in a foreign country during COVID-19. Anger stood out as the predominant emotion.

**Figure S3.** Percentage of different sources of information where participants learned about discrimination against Chinese in other countries during COVID-19.


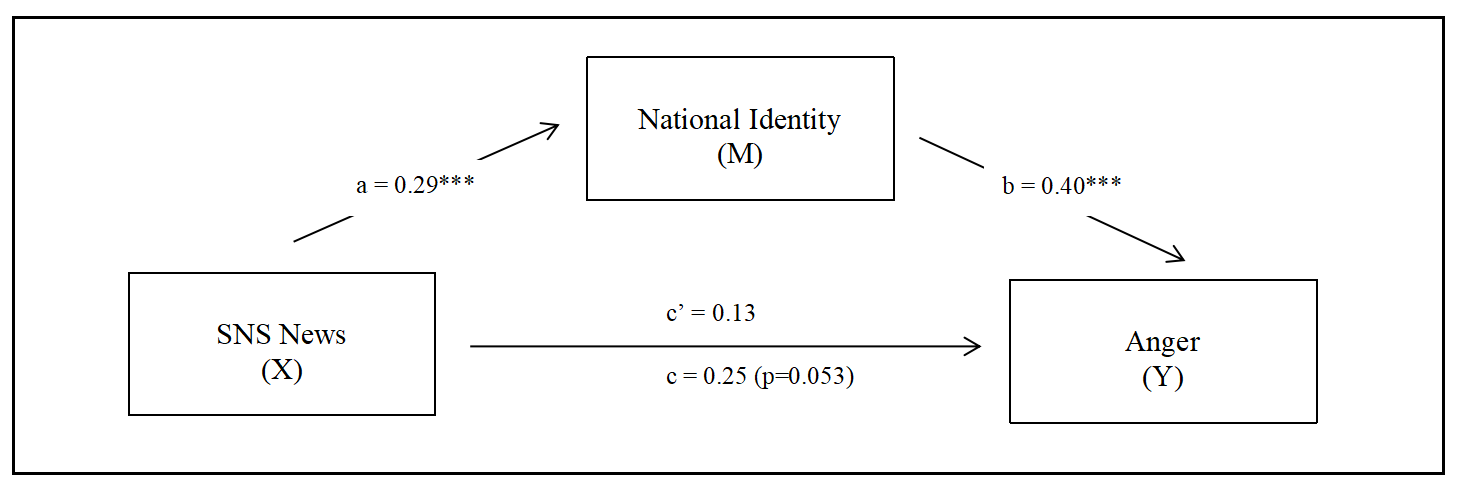


**Figure S4. Mediation Model.** In this model, gaining information about anti-Chinese discrimination from SNS news was positively associated with national identity and national identity was positively associated with anger towards this information. We caution readers to interpret this marginal mediation carefully as the measurement of SNS News as an information source is not empirically reliable. ***p<0.001, **p<0.01, *p<0.5; SNS News Use (1=Yes; 0=No).


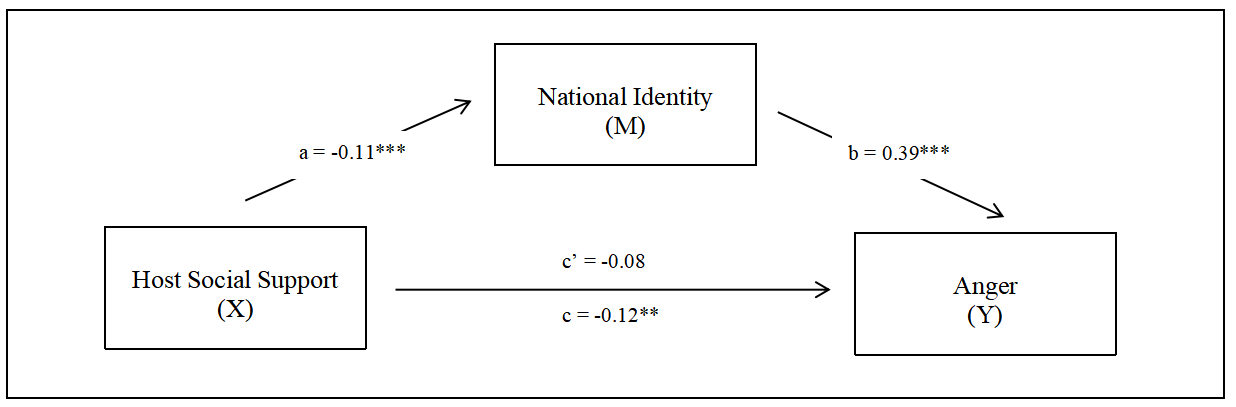


***p<0.001, **p<0.01, *p<0.5.

**Figure S5.** **Mediation Model.** In this model, having host social support was negatively associated with national identity and national identity was positively associated with anger towards group discrimination. Host social support significantly alleviated anger with national identity as a mediator.

**Figure S6.** Country-level host newspaper coverage on discrimination against Chinese during COVID-19.

January 31 was the day when WHO announced the new coronavirus a global emergency and the US closed border to travelers who had been to China in the last 14 days.

**Figure S7. World News Trend.** Global reporting (by country-level host newspaper coverage) on discrimination against Chinese during COVID-19.

**Figure S8.** Country-level ethnic Chinese newspaper coverage on discrimination against Chinese during COVID-19.

**Figure S9.** Country-level stacked host and ethnic Chinese newspaper coverage on discrimination against Chinese during COVID-19.


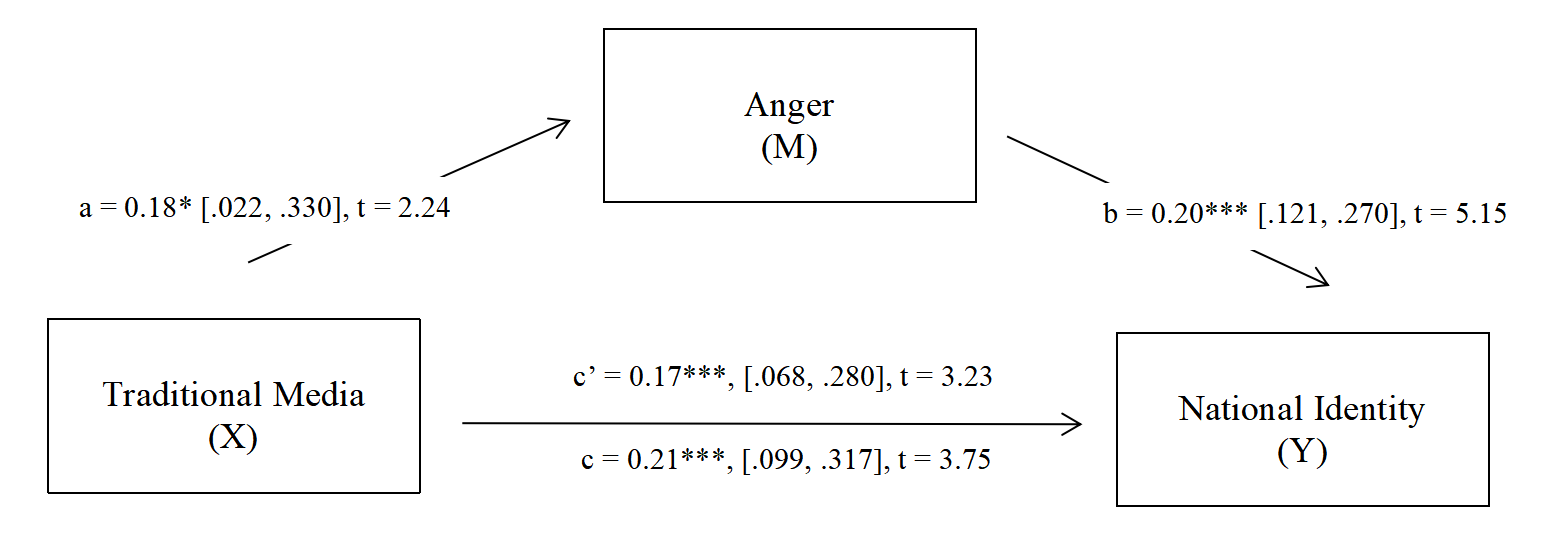


**Figure S10.** Alternative model with anger as the mediator between traditional media and national identity (Study 1).


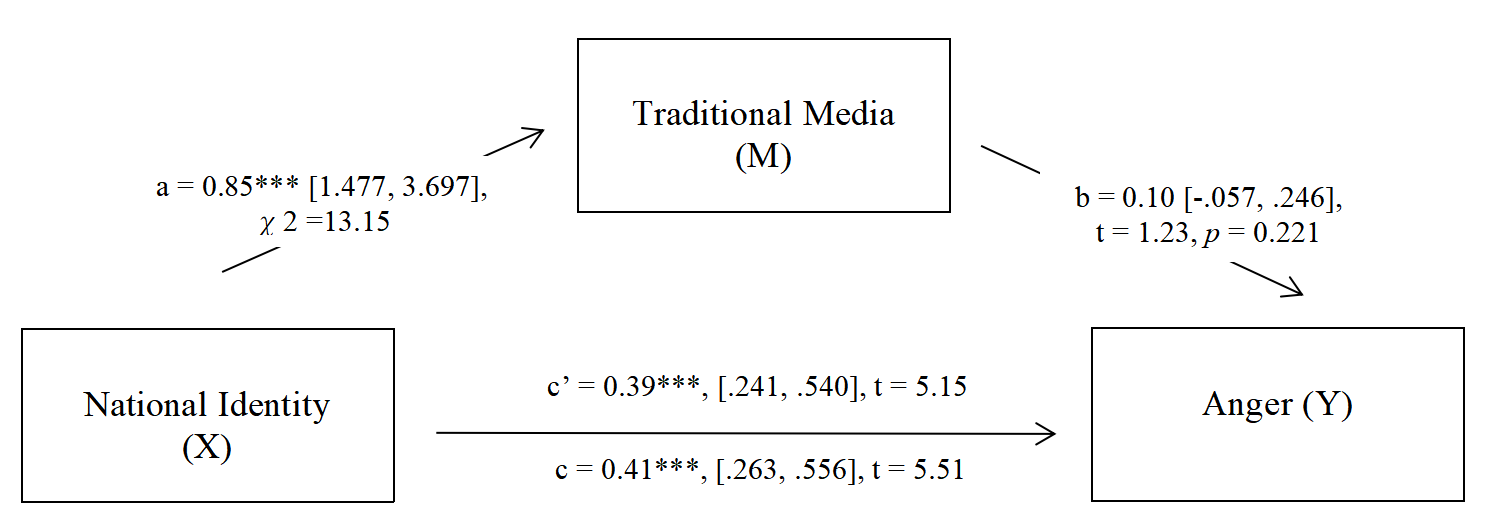


**Figure S11.** Alternative model with national identity as the predictor (National Identity → Traditional Media → Anger).


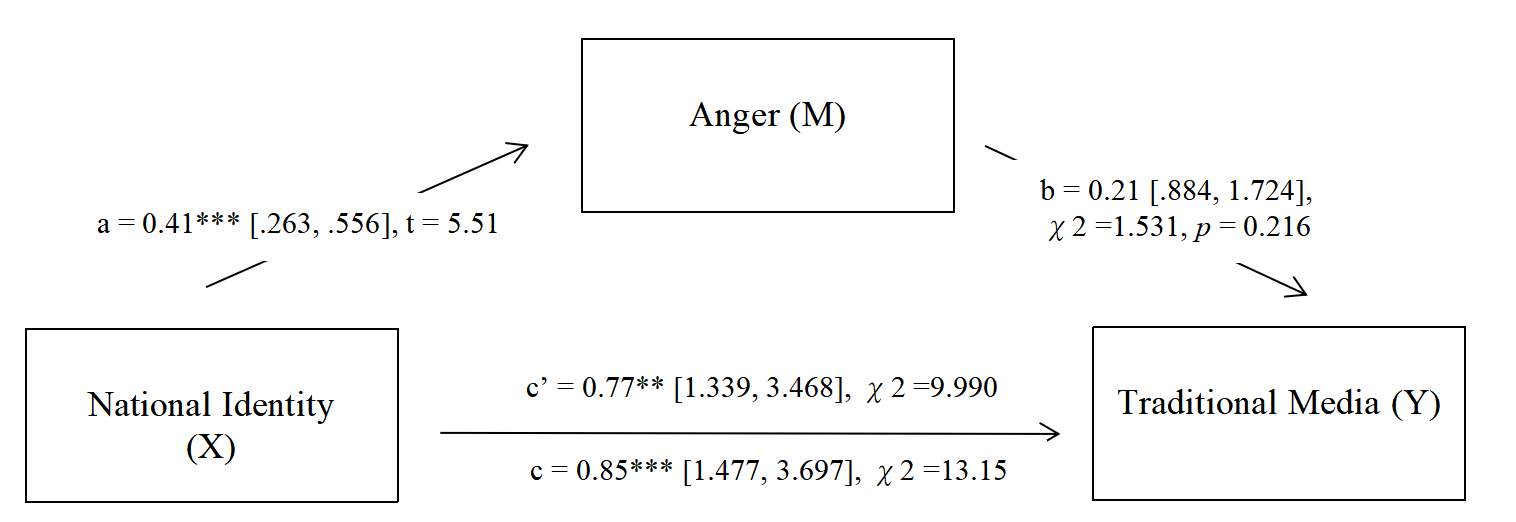


**Figure S12.** Alternative model with national identity as the predictor (National Identity → Anger → Traditional Media).


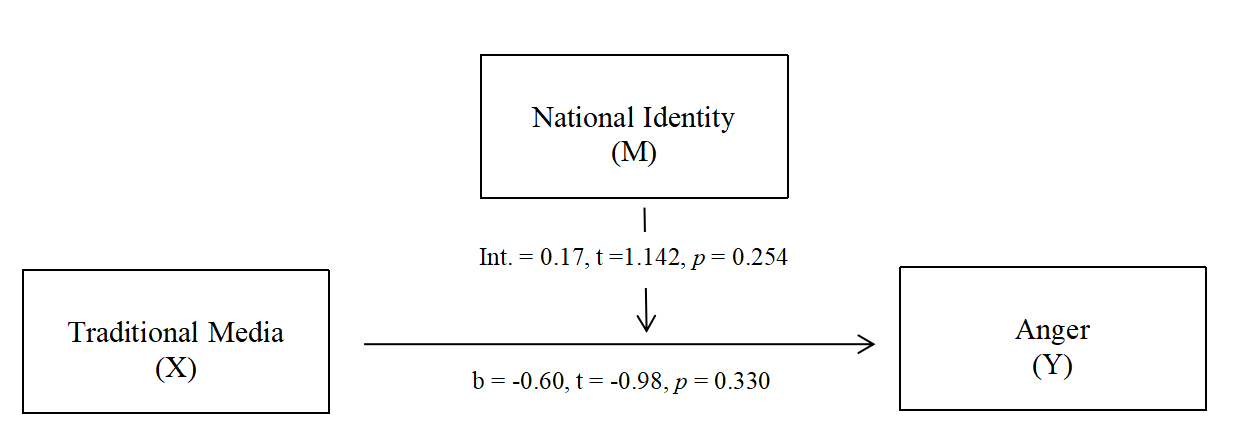


**Figure S13.** Alternative model with national identity as a moderator.


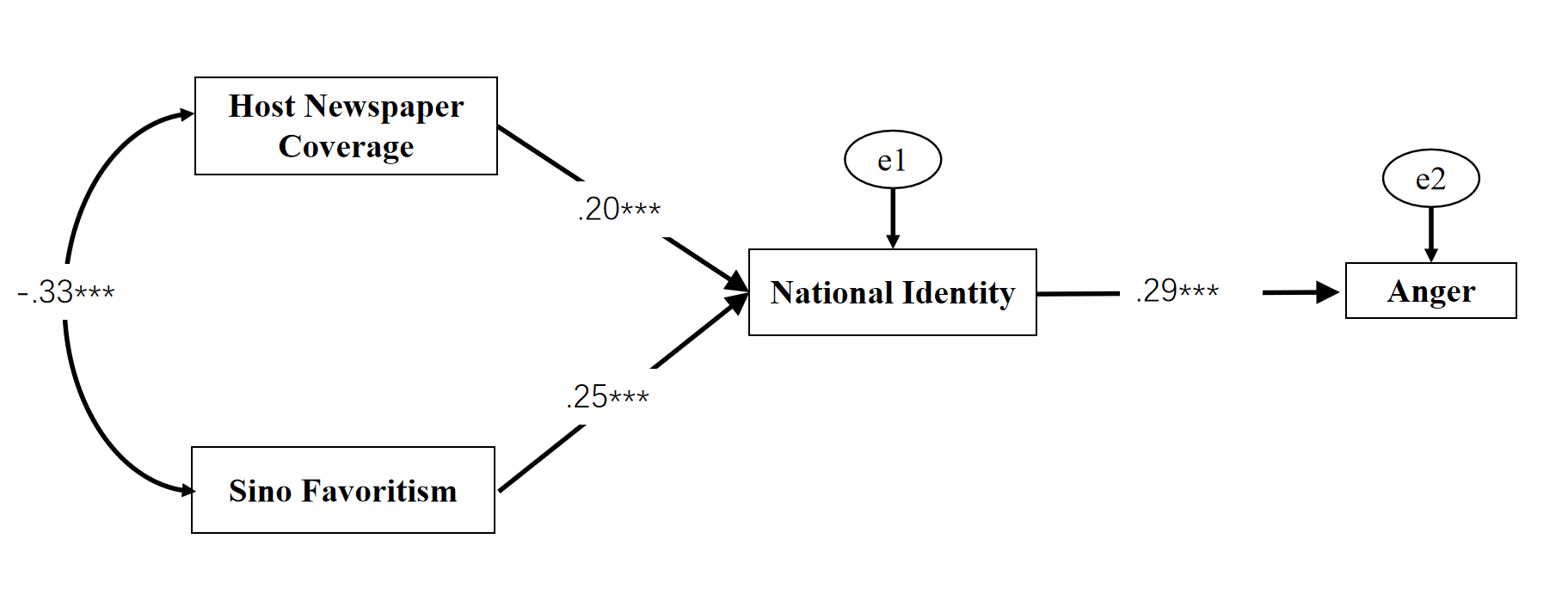


**Figure S14.** National Identity structural model (χ^2^(2) = 3.026, *p* = 0.220, CFI = 0.993; RMSEA = 0.040). Note: All reported estimates are standardized. * *p* < 0.05, ** *p* < 0.01, *** *p* < 0.001. Education, gender and age were controlled. .


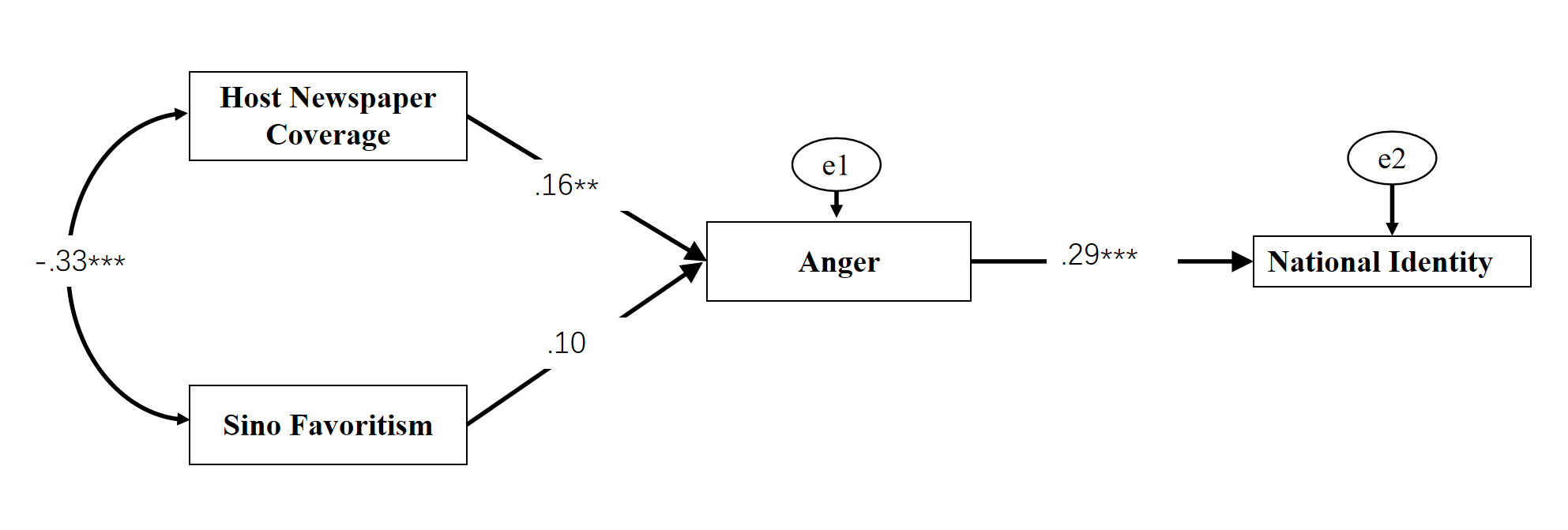


**Figure S15.** Anger structural model (χ^2^(2) = 18.784, *p* = 0.000, CFI = 0.885; RMSEA = 0.161). Note: All reported estimates are standardized. * *p* < 0.05, ** *p* < 0.01, *** *p* < 0.001. Education, gender and age were controlled.


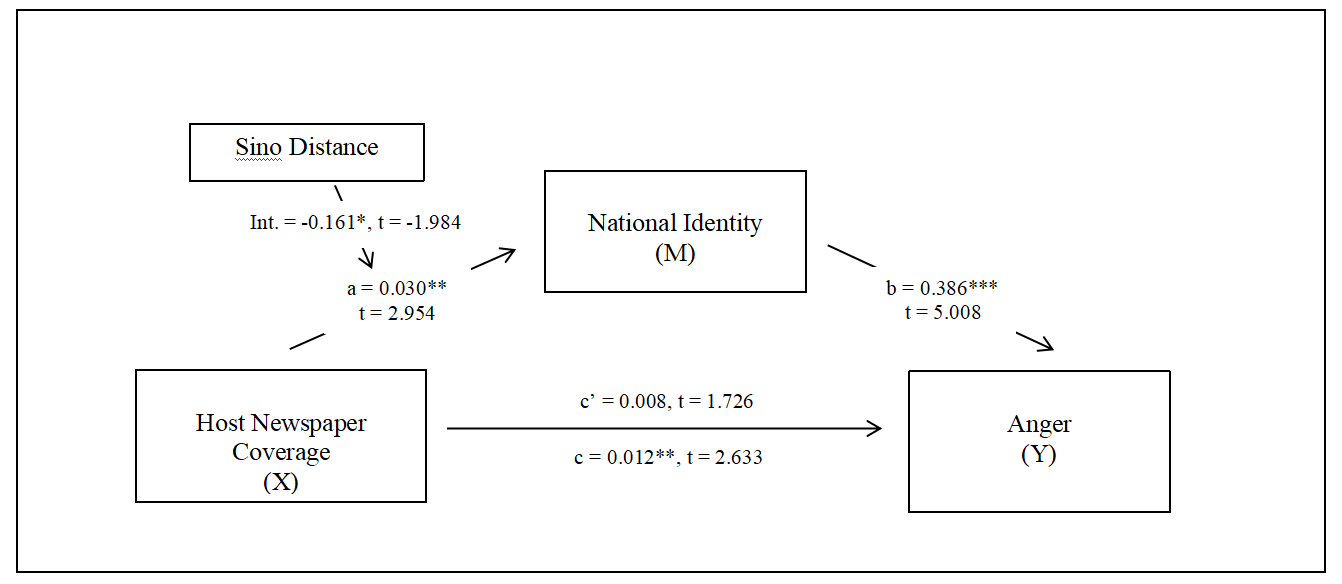


**Figure S16.** **Moderated Mediation.** Interaction effect of Sino Distance x Host Newspaper Coverage on national identity when controlling for Sino Favoritism.

**S1 Table. Independent Samples *t*-tests and Descriptive Statistics by Repatriate/Non-repatriate.**

| Outcome | Group | | | | | 95% CI for Mean Difference |  |  |  |
| --- | --- | --- | --- | --- | --- | --- | --- | --- | --- |
|  | Repatriates (N=57) | |  | Non-Repatriates (N=269) | |  |  |  |  |
|  | *M* | *SD* |  | *M* | *SD* |  | *t* | *df* | *p* |
| Traditional Media | 0.60 | 0.50 |  | 0.55 | 0.50 | -0.09, 0.19 | 0.69 | 324 | 0.09 |
| SNS News | 0.88 | 0.33 |  | 0.90 | 0.30 | -0.11, 0.06 | -0.50 | 324 | 0.32 |
| SNS Anecdotes | 0.68 | 0.47 |  | 0.61 | 0.49 | -0.06, 0.22 | 1.10 | 324 | 0.01 |
| Acquaintances | 0.53 | 0.50 |  | 0.47 | 0.50 | -0.09, 0.20 | 0.74 | 324 | 0.97 |
| Self-experience | 0.39 | 0.49 |  | 0.28 | 0.45 | -0.03, 0.24 | 1.55 | 324 | 0.01 |
| Do Not Know | 0.04 | 0.19 |  | 0.02 | 0.15 | -0.03, 0.06 | 0.57 | 324 | 0.26 |
| Anger | 4.19 | 0.79 |  | 4.23 | 0.69 | -0.24, 0.16 | -0.37 | 324 | 0.14 |
| National Identity | 3.92 | 0.51 |  | 3.96 | 0.50 | -0.18, 0.11 | -0.49 | 324 | 0.89 |
| Host Social Support | 3.17 | 0.88 |  | 3.17 | 0.87 | -0.25, 0.25 | -0.02 | 324 | 0.92 |

**S2 Table.** **Factor Analysis.** Factor reduction using principal component analysis of 11 emotional responses to hypothetical discrimination.

| Rotated Component Matrix | **Inferiority** | **Sadness** | **Shame** | **Anger** |
| --- | --- | --- | --- | --- |
| 1. Angry |  |  |  | 0.762 |
| 1. Frustrated |  | 0.741 |  |  |
| 1. Sad |  | 0.786 |  |  |
| 1. Powerless | 0.413 |  |  |  |
| 1. Hopeless | 0.625 |  |  |  |
| 1. Scared | 0.652 |  |  |  |
| 1. Vulnerable | 0.8 |  |  |  |
| 1. Humiliated |  |  | 0.731 |  |
| 1. Vengeful |  |  | 0.793 |  |
| 1. Inferior | 0.559 |  |  |  |
| 1. Not Surprised Or Resigned |  |  |  |  |

Extraction Method: Principal Component; Rotation Method: Varimax with Kaiser Normalization; Rotation converged in 5 iterations. Anger stood out as a unique factor.

**S3 Table.** **Correlation Analyses.** Correlations of four emotional factors with major variables.

| N = 326 | 1 | 2 | 3 | 4 | 5 | 6 | 7 | 8 | 9 | M | SD |
| --- | --- | --- | --- | --- | --- | --- | --- | --- | --- | --- | --- |
| Gender | - |  |  |  |  |  |  |  |  | 1.70 | 0.46 |
| Age | -0.14* | - |  |  |  |  |  |  |  | 27.52 | 6.10 |
| Education | -0.06 | 0.55*** | - |  |  |  |  |  |  | 6.98 | 1.49 |
| Anger# | 0.08 | 0.01 | 0.05 | - |  |  |  |  |  | - | - |
| Inferiority | 0.15** | -0.09 | -0.05 | -0.14* | - |  |  |  |  | - | - |
| Sadness | -0.03 | -0.02 | -0.03 | -0.15** | -0.11 | - |  |  |  | - | - |
| Shame | -0.17** | 0.09 | 0.08 | 0.05 | -0.48*** | -0.20*** | - |  |  | - | - |
| Sino Distance | 0.03 | 0.09 | 0.10 | 0.06 | 0.09 | 0.12* | -0.07 | - |  | 0.14 | 0.03 |
| National Identity | 0.04 | -0.04 | -0.04 | 0.14* | 0.00 | 0.03 | -0.03 | -0.05 | - | 3.95 | 0.50 |

***p < 0.001, **p < 0.01, *p < 0.5; Anger^#^ here differs from “anger” in main study. Spearman’s correlation was adopted for this analysis.

**S4 Table.** **Correlation Analyses.** Correlations of other sources of information with major variables in the exploratory analysis.

| N = 326 | 1 | 2 | 3 | 4 | 5 | 6 | 7 | 8 | 9 | M | SD |
| --- | --- | --- | --- | --- | --- | --- | --- | --- | --- | --- | --- |
| 1. Gender | - |  |  |  |  |  |  |  |  | 1.70 | 0.46 |
| 1. Age | -0.14* | - |  |  |  |  |  |  |  | 27.52 | 6.10 |
| 1. Education | -0.06 | 0.55*** | - |  |  |  |  |  |  | 6.98 | 1.49 |
| 1. SNS Anecdotes | 0.08 | -0.16** | -0.04 | - |  |  |  |  |  | 0.62 | 0.49 |
| 1. Acquaintances | 0.11 | -0.13* | -0.03 | 0.35*** | - |  |  |  |  | 0.48 | 0.50 |
| 1. Self-experience | 0.16** | -0.15** | -0.07 | 0.11* | 0.43*** | - |  |  |  | 0.30 | 0.46 |
| 1. Do Not Know | -0.02 | 0.05 | -0.01 | -0.12* | -0.11* | -0.06 | - |  |  | 0.02 | 0.16 |
| 1. Anger | -0.03 | -0.01 | -0.04 | 0.05 | 0.01 | -0.04 | -0.03 | - |  | 4.22 | 0.70 |
| 1. National Identity | 0.04 | 0.01 | -0.01 | -0.05 | 0.00 | -0.06 | -0.04 | 0.12* | - | 3.95 | 0.50 |

***p<=0.001, **p<=0.01, *p<=0.5; “Do Not Know” means respondents reported not knowing about anti-Chinese discrimination during COVID. Variables 4, 5, 6, and 7 were binary (1 = Yes, 0 = No) and Spearman’s correlation was adopted.

**S5 Table. Hierarchical Regression.** Testing traditional media as a predictor of anger and national identity as a mediator.

|  | step 1 | | step 2 | | step 3 | |
| --- | --- | --- | --- | --- | --- | --- |
| N = 326 | beta | p | beta | p | beta | p |
| Gender | -.03 | .65 | -.02 | .75 | -.03 | .60 |
| Age | .00 | .96 | .00 | .95 | .00 | .99 |
| Education | -.04 | .52 | -.03 | .60 | -.03 | .60 |
| Traditional Media |  |  | .11 | .03 | .08 | .22 |
| National Identity |  |  |  |  | .28 | .00 |
| **model statistics** |  | |  | |  | |
| *F (df)* | .22 (3,322) | | 1.42 (4, 321) | | 6.53 (5, 320) | |
| *R^2^* | .00 | | .02 | | .09 | |
| *ΔR^2^* | .02 | | .02 | | .08 | |

**S6 Table. Hierarchical Regression.** Testing SNS news as a predictor of anger and national identity as a mediator.

|  | step 1 | | step 2 | | step 3 | |
| --- | --- | --- | --- | --- | --- | --- |
| N = 326 | beta | p | beta | p | beta | p |
| Gender | -.03 | .65 | -.02 | .73 | -.03 | .59 |
| Age | .00 | .96 | .01 | .91 | .00 | .99 |
| Education | -.04 | .52 | -.04 | .51 | -.03 | .55 |
| SNS News |  |  | .11 | .05 | .06 | .29 |
| National Identity |  |  |  |  | .28 | .00 |
| **model statistics** |  | |  | |  | |
| *F (df)* | .22 (3,322) | | 1.11 (4, 321) | | 6.44 (5, 320) | |
| *R^2^* | .00 | | .01 | | .09 | |
| *ΔR^2^* | .00 | | .01 | | .08 | |

**S7 Table. Sourcing Host Media.** List of media outlets sourced for online host newspaper reports of discrimination against Chinese during COVID-19 by country (Jan.23-Apr.23, 2020).

| **Country/ Region** | **Number of Participants** | **Number of Reports** | **End Date of Search** | **Top 3 Newspapers** | **Ranking Reference** |
| --- | --- | --- | --- | --- | --- |
| USA | 60 | 23 | 21/04/2020 | USA Today; Wall Street Journal (WSJ), New York Times (NYT) | https://en.wikipedia.org/wiki/List_of_newspapers_in_the_United_States |
| Spain | 42 | 7 | 20/04/2020 | El Pais; El Mundo; ABC | https://en.wikipedia.org/wiki/List_of_newspapers_in_Spain |
| Great Britain | 42 | 25 | 19/04/2020 | Metro; The Sun; Daily Mail | https://en.wikipedia.org/wiki/List_of_newspapers_in_the_United_Kingdom_by_circulation#2020_to_present |
| Italy | 29 | 18 | 23/04/2020 | Corriere della Sera; La Republica; Il Sole 24 Ore | https://en.wikipedia.org/wiki/List_of_newspapers_in_Italy |
| Germany | 19 | 11 | 16/04/2020 | Süddeutsche Zeitung; Frankfurter Allgemeine Zeitung; Die Welt | https://en.wikipedia.org/wiki/List_of_newspapers_in_Germany |
| Australia | 17 | 19 | 21/04/2020 | ABC News; News.com.au; 7NEWS | https://www.theguardian.com/media/2020/apr/14/guardian-australia-doubles-audience-to-become-fourth-most-popular-news-site-in-the-country |
| Japan | 15 | 4 | 18/04/2020 | 読売新聞 (Yomiuri Shimbun); 朝日新聞 (Asahi Shimbun); 毎日新聞 (Mainichi Shimbun) | https://www.wikiwand.com/en/Newspaper_circulation#:~:text=The%20Japanese%20Yomiuri%20Shimbun%20(%E8%AA%AD%E5%A3%B2,circulated%20newspapers%20in%20the%20world. |
| South Korea | 14 | 42 | 16/04/2020 | 조선일보(Chosun Ilbo); 중앙 일보(Joonhang Illbo); 동아일보(Donga Ilbo) | https://seoulz.com/the-south-korean-media-and-newspapers-a-full-breakdown/ |
| Russia | 13 | 5 | 20/04/2020 | Komsomolskaya Pravda; Russian Newspaper; Novaya Gazeta | https://www.mlg.ru/ratings/media/federal/8058/ |
| Peru | 13 | 9 | 18/04/2020 | El Comercio Peru; La Republica Peru; Peru.com | https://www.allyoucanread.com/peru-newspapers/ |
| France | 9 | 11 | 20/04/2020 | Le Monde; Le Figaro; Le Parisien | https://en.wikipedia.org/wiki/Mass_media_in_France |
| Singapore | 8 | 27 | 17/04/2020 | The Straits Times; 联合早报 (Lianhe Zaobao); 联合晚报 (Lianhe Wanbao) | https://en.wikipedia.org/wiki/List_of_newspapers_in_Singapore |
| Brazil | 6 | 22 | 18/04/2020 | Folha de S. Paulo; O Estabo; O Globo | https://en.wikipedia.org/wiki/List_of_newspapers_in_Brazil |
| Malaysia | 5 | 19 | 19/04/2020 | New Straits Times; The Star; The Sun | https://www.researchgate.net/publication/233357149_Electoral_Authoritarianism_and_the_Print_Media_in_Malaysia_Measuring_Political_Bias_and_Analyzing_Its_Cause/figures?lo=1 |
| Canada | 5 | 20 | 20/04/2020 | The Globe and Mail; Toronto Star; Hamilton Spectator | https://www.agilitypr.com/resources/top-media-outlets/top-10-canadian-print-outlets/ |
| Angola | 4 | 1 | 17/04/2020 | Angola Press Agency; Jornal de Angola; Ver Angola | https://en.wikipedia.org/wiki/Mass_media_in_Angola |
| Belgium | 4 | 9 | 18/04/2020 | Het Laatste Nieuws; Het Nieuwsblad, Le Soir | https://www.4imn.com/be/ |
| Portugal | 3 | 13 | 17/04/2020 | Correio de Manhã; Expresso; Jornal de Noticias | https://en.wikipedia.org/wiki/List_of_newspapers_in_Portugal |
| India | 2 | 10 | 18/04/2020 | The Times of India; The Hindu; Hindustan Times | https://en.wikipedia.org/wiki/List_of_newspapers_in_India_by_circulation |
| Greece | 2 | 0 | 19/04/2020 | Ta Nea; To Vima; Kathimerini | https://www.4imn.com/gr/ |
| Sweden | 2 | 8 | 16/04/2020 | Dagens Nyheter; Göteborgs-Posten; Sydsvenskan | https://medialandscapes.org/country/sweden/media/print |
| Saudi Arabia | 1 | 2 | 16/04/2020 | Arab News; Asharq Al-Awsat; Al Riyadh | https://www.4imn.com/sa/ |
| Netherlands | 1 | 15 | 16/04/2020 | De Telegraaf; Algemeen Dagblad; De Volkskrant | https://en.wikipedia.org/wiki/List_of_newspapers_in_the_Netherlands |
| Switzerland | 1 | 11 | 16/04/2020 | 20 Minuten; 20 minutes; Tages-Anzeiger | https://www.statista.com/statistics/701430/daily-newspapers-highest-circulation-switzerland/ |
| Mexico | 1 | 16 | 16/04/2020 | El Universal; Reforma; La Jornada | https://posora.com/mexico-daily-newspapers/ |
| South Africa | 1 | 1 | 17/04/2020 | Daily Sun; Isolezwe; The Star | http://www.marklives.com/2017/05/abc-analysis-q1-2017-the-biggest-circulating-newspapers-in-sa/ |
| Vietnam | 1 | 9 | 18/04/2020 | Tuoi Tre; Viet Nam News; Tien Phong | https://www.4imn.com/vn/ |
| Kenya | 1 | 4 | 18/04/2020 | Daily Nation; Standard; Faifa Leo | https://businesstoday.co.ke/newspaper-readership-in-kenya-daily-nation-standard/ |
| Guatemala | 1 | 5 | 18/04/2020 | Prensa Libre; El Periodio; La Hora | https://www.4imn.com/gt/ |
| Uraguay | 1 | 4 | 18/04/2020 | El Pais, El Observador; La Republica | https://www.4imn.com/uy/ |
| Chile | 1 | 8 | 18/04/2020 | Las Ultimas Noticias; La Tercera; El Mostrador | https://www.4imn.com/cl/ |
| Kuwait | 1 | 0 | 18/04/2020 | Arab Times; Kuwait Times; Al Rai Al Aam | https://www.4imn.com/kw/ |
| Lebanon | 1 | 1 | 16/04/2020 | Al Balad; The Daily Star; Al Akhbar | https://www.4imn.com/lb/ |

Note: 1) All financial newspapers were excluded even if ranking was high; 2) India’s 1^st^ (Dainik Jagran) and 2^nd^ (Dainik Bhaskar) circulated newspapers are in Hindi and no relevant reports could be found, researchers switched to all English newspaper instead; 3) As Australian newspaper websites required paid readership and limited open access, researchers used the top 3 news websites (no print circulation) as alternatives; 4) Peru.com is not sourced as it is not a newspaper; 5) Cells coded as 0 mean no report was found.

**S8 Table.** **Host Newspaper Coverage.** List of online host newspaper reports of discrimination against Chinese during COVID-19 by country.

| **USA (Jan.23-Apr.21, 2020)** | | |
| --- | --- | --- |
| **Newspaper** | **Date** | **News Title** |
| USA Today | 3/28/2020 | [They look at me and think I’m some kind of virus: What it’s like to be Asian during the coronavirus pandemic](https://www.usatoday.com/story/news/nation/2020/03/28/coronavirus-racism-asian-americans-report-fear-harassment-violence/2903745001/) |
| USA Today | 3/26/2020 | [Asian American lawmakers sound the alarm on coronavirus-related discrimination](https://www.usatoday.com/story/news/politics/2020/03/26/coronavirus-asian-american-lawmakers-fear-rise-discrimination/2890935001/) |
| USA Today | 3/11/2020 | [No, calling the novel coronavirus the Wuhan virus is not racist](https://www.usatoday.com/story/opinion/2020/03/11/sars-cov-2-chinese-coronavirus-isnt-racist-wuhan-covid-19-column/5020771002/) |
| USA Today | 3/2/2020 | [Coronavirus on college campuses: Fight fear and racism along with the outbreak](https://www.usatoday.com/story/opinion/2020/03/01/coronavirus-fight-fear-racism-anti-chinese-sentiment-campus-column/4913481002/) |
| USA Today | 4/15/2020 | [Foreign policy experts call for the end to hate crimes against Asian American community: Readers](https://www.usatoday.com/story/opinion/readers/2020/04/15/asian-american-community-pushes-back-against-hate-crimes-reader-views/2991666001/) |
| USA Today | 1/31/2020 | [Coronavirus is spreading. And so is anti-Chinese sentiment and xenophobia](https://www.usatoday.com/story/news/nation/2020/01/31/coronavirus-chinese-xenophobia-racism-misinformation/2860391001/) |
| USA Today | 3/20/2020 | [Trump uses China as a foil when talking coronavirus, distancing himself from criticism](https://www.usatoday.com/story/news/politics/2020/03/20/coronavirus-trump-blames-china-distancing-himself-fallout/2876983001/) |
| USA Today | 3/10/2020 | [Democratic lawmakers call on Republicans to apologize for “bigoted” coronavirus language](https://www.usatoday.com/story/news/politics/2020/03/10/coronavirus-democrats-slam-bigoted-language-republicans/5016354002/) |
| WSJ | 3/30/2020 | [Harvard’s China Virus](https://www.wsj.com/articles/harvards-china-virus-11585585597?mod=searchresults_pos4&page=1) |
| WSJ | 4/19/2020 | [NYC has logged 248 complaints of coronavirus discrimination](https://www.wsj.com/articles/new-york-city-has-logged-248-complaints-of-coronavirus-discrimination-11587308400?mod=searchresults_pos1&page=1) |
| WSJ | 2/3/2020 | [China is the real sick man of Asia](https://www.wsj.com/articles/china-is-the-real-sick-man-of-asia-11580773677) |
| NYT | 4/12/2020 | [The slur I never expected to hear in 2020](https://www.nytimes.com/2020/04/12/magazine/asian-american-discrimination-coronavirus.html) |
| NYT | 2/13/2020 | [As Coronavirus spreads, so does anti-Chinese sentiment](https://www.nytimes.com/2020/01/30/world/asia/coronavirus-chinese-racism.html?auth=login-google) |
| NYT | 3/29/2020 | [How Asian-American leaders are grappling with xenophobia amid coronavirus](https://www.nytimes.com/2020/03/29/us/politics/coronavirus-asian-americans.html) |
| NYT | 3/23/2020 | [Call it “Coronavirus”](https://www.nytimes.com/2020/03/23/opinion/china-coronavirus-racism.html) |
| NYT | 3/14/2020 | [Coronavirus racism infected my high school](https://www.nytimes.com/video/opinion/100000007028034/racism-coronavirus-asians.html) |
| NYT | 2/12/2020 | [For a Chinese traveler, even paradise comes with prejudice](https://www.nytimes.com/2020/02/12/world/asia/china-coronavirus-korea-discrimination.html) |
| NYT | 3/7/2020 | [I’m Chinese. That doesn’t mean I have coronavirus](https://www.nytimes.com/2020/03/07/opinion/coronavirus-asian-racism.html) |
| NYT | 3/13/2020 | [Politicians’ Use of ‘Wuhan Virus’ Starts a Debate Health Experts Wanted to Avoid](https://www.nytimes.com/2020/03/10/us/politics/wuhan-virus.html) |
| NYT | 3/19/2020 | [Trump Defends Using ‘Chinese Virus’ Label, Ignoring Growing Criticism](https://www.nytimes.com/2020/03/18/us/politics/china-virus.html) |
| NYT | 2/18/2020 | [‘Are You Sick?’ For Asian-Americans, a Sneeze Brings Suspicion](https://www.nytimes.com/2020/02/16/us/coronavirus-american-mood.html) |
| NYT | 1/31/2020 | [An Outbreak of Racist Sentiment as Coronavirus Reaches Australia](https://www.nytimes.com/2020/01/31/world/australia/coronavirus-racism-chinese.html) |
| NYT | 3/3/2020 | [‘The Face of the Coronavirus’: A Hong Kong Student Shunned in Italy](https://www.nytimes.com/2020/03/02/world/asia/coronavirus-italy-racism.html) |
| **Spain (Jan.23-Apr.20, 2020)** | | |
| **Newspaper** | **Date** | **News Title** |
| El Pais | 2/24/2020 | [Psicosis, pánico y racismo](https://elpais.com/sociedad/2020/02/23/actualidad/1582490112_320967.html) [Psychosis, panic and racism: the darker side of the coronavirus] |
| El Pais | 2/19/2020 | [Coronavirus desata 'brote' de xenofobia contra personas con rasgos orientales, ¿pasa en Cali?](https://www.elpais.com.co/cali/coronavirus-desata-brote-de-xenofobia-contra-personas-con-rasgos-orientales-pasa-en.html) [Coronavirus unleashes an ‘outbreak’ of xenophobia against people with oriental features, does it happen in Cali?] |
| El Pais | 3/24/2020 | [Temor en la comunidad asiática en Estados Unidos ante los ataques racistas por el coronavirus](https://elpais.com/internacional/2020-03-23/temor-en-la-comunidad-asiatica-en-estados-unidos-ante-los-ataques-racistas-por-el-coronavirus.html) [Fear in the Asian community in the United States before the racist attacks by the coronavirus] |
| El Mundo | 2/2/2020 | [Brotes racistas en todo el mundo contra chinos y asiáticos tras el brote de coronavirus](https://www.elmundo.es/papel/2020/02/02/5e36b134fdddffd4318b46a2.html) [Bachelet criticizes wave of prejudice against Chinese by coronavirus] |
| ABC | 3/2/2020 | [La maquinaria de injerencias rusa difunde bulos sobre el coronavirus también en español](https://www.abc.es/sociedad/abci-maquinaria-injerencias-rusa-difunde-bulos-sobre-coronavirus-tambien-espanol-202003010225_noticia.html) [The Russian interference machinery spreads hoaxes about the coronavirus also in Spanish] |
| ABC | 2/4/2020 | [La Embajada de China afirma que "el enemigo es el coronavirus, no los chinos"](https://www.abc.es/espana/abci-embajada-china-afirma-enemigo-coronavirus-no-chinos-202002041548_video.html) [The Chinese Embassy affirms that “the enemy is the coronavirus, not the Chinese”] |
| **Great Britain (Jan.23-Apr.19, 2020)** | | |
| **Newspaper** | **Date** | **News Title** |
| Metro | 1/31/2020 | [Coronavirus doesn’t discriminate, so neither should you](https://metro.co.uk/2020/01/31/coronavirus-racism-12159409/) |
| Metro | 3/31/2020 | [Coronavirus could ‘deepen racial divides’ in the UK](https://metro.co.uk/2020/03/31/coronavirus-deepen-racial-divides-uk-12454718/) |
| Metro | 2/6/2020 | [Just because I’m Chinese doesn’t mean I have coronavirus](https://metro.co.uk/2020/02/06/just-im-chinese-doesnt-mean-coronavirus-12194030/) |
| Metro | 2/9/2020 | [Royal Caribbean bans Chinese nationals from its ships amid coronavirus concerns](https://metro.co.uk/2020/02/09/royal-caribbean-bans-chinese-nationals-ships-amid-coronavirus-concerns-12209253/) |
| Metro | 3/19/2020 | [Asian woman racially abused on train because of coronavirus pandemic](https://metro.co.uk/2020/03/19/asian-woman-faces-racist-abuse-train-coronavirus-12424389/) |
| Metro | 2/23/2020 | [Woman knocked out defending Chinese friend in racist ‘coronavirus’ attack](https://metro.co.uk/2020/02/23/woman-knocked-defending-chinese-friend-racist-coronavirus-attack-12286814/) |
| Metro | 3/18/2020 | [Donal Trump says calling coronavirus Chinese Virus not racist](https://metro.co.uk/2020/03/18/donald-trump-says-calling-coronavirus-chinese-virus-not-racist-12419530/) |
| Metro | 2/28/2020 | [Loose Women’s Linda Robson admits husband warned her against ordering Chinese takeaway because of coronavirus](https://metro.co.uk/2020/02/28/loose-womens-linda-robson-admits-husband-told-avoid-ordering-chinese-takeaway-coronavirus-12320581/) |
| Metro | 3/11/2020 | [Italian makeup artist creates coronavirus mask look to send an important message](https://metro.co.uk/2020/03/11/italian-makeup-artist-creates-coronavirus-mask-look-send-important-message-12380750/) |
| Metro | 2/11/2020 | [Student’s airbnb cancelled last minute due to coronavirus](https://metro.co.uk/2020/02/11/students-airbnb-cancelled-last-minute-coronavirus-12222061/) |
| Daily Mail | 2/5/2020 | [How Chinese children in UK are suffering a racist backlash due to coronavirus](https://www.dailymail.co.uk/news/article-7964263/Chinese-children-UK-suffer-racist-backlash-coronavirus.html) |
| Daily Mail | 4/15/2020 | [Coronavirus fuels racist backlash with calls to ‘nuke’ China and shops banning Chinese customers over killer bug](https://www.dailymail.co.uk/news/article-8220873/Chinese-NHS-worker-wins-30-000-racial-discrimination-claim-colleague-Kung-Fu-noises.html) |
| Daily Mail | 3/13/2020 | [Ben Foden’s pregnant wife Jackie is accused of racism after she shares post blaming the Chinese government for spreading coronavirus](https://www.dailymail.co.uk/tvshowbiz/article-8108201/Ben-Fodens-pregnant-wife-Jackie-accused-racism-against-chinese.html) |
| Daily Mail | 3/23/2020 | [Donald Trump promises to ‘protect’ Asian Americans against racist backlash](https://www.dailymail.co.uk/news/article-8144767/Donald-Trump-promises-protect-Asian-Americans-against-racist-backlash-coronavirus.html) |
| Daily Mail | 1/30/2020 | [‘We are not the virus’: Chinese community urge Australian public not to blame them for Coronavirus](https://www.dailymail.co.uk/news/article-7945513/Chinese-community-urge-Australians-not-blame-Corona-outbreak.html) |
| Daily Mail | 4/6/2020 | [Bill De Blasio warns of ‘disgusting’ rise in hate crimes against Asian Americans](https://www.dailymail.co.uk/news/article-8192625/Bill-Blasio-warns-disgusting-rise-hate-crimes-against-Asian-Americans-pandemic.html) |
| Daily Mail | 2/5/2020 | [‘Stop eating bats and snakes’: Beauty blogger, 32, reveals xenophobic and racist abuse she’s received over coronavirus](https://www.dailymail.co.uk/news/article-7967633/Asian-American-beauty-blogger-32-bombarded-xenophobic-racist-abuse-coronavirus.html) |
| Daily Mail | 4/15/2020 | [Chinese Australians say they are the victims of disgusting racist attacks](https://www.dailymail.co.uk/news/article-8220099/Chinese-Australians-claim-victims-racist-attacks-coronavirus-crisis.html) |
| The Sun | 4/5/2020 | [Asian woman left needing stitches after attack by three teen girls and woman who hit her with umbrella on NYC bus](https://www.thesun.co.uk/news/11333726/asian-woman-attacked-umbrella-nyc-bus/) |
| The Sun | 1/30/2020 | [‘NO CHINESE ALLOWED’ Coronavirus fuels racist backlash with calls to ‘nuke’ China and shops banning Chinese customers over killer bug](https://www.thesun.co.uk/news/10852439/coronavirus-racist-backlash-nuke-china-shops-ban-customers/) |
| The Sun | 3/23/2020 | [Fauci says he has to tell White House things FOUR times & he would ‘never’ call coronavirus the ‘Chinese Virus’](https://www.thesun.co.uk/news/11236012/inside-dr-anthony-faucis-battles-and-surprises-when-dealing-with-president-trump-and-vice-president-pence/) |
| The Sun | 3/19/2020 | [Trump-backing Senator says ‘China to blame for coronavirus because they eat bats, snakes and dogs’](https://www.thesun.co.uk/news/11209256/trump-backing-senator-says-china-to-blame-for-coronavirus-because-they-eat-bats-snakes-and-dogs/) |
| The Sun | 3/6/2020 | [Spike in vigilante attacks on Asians over coronavirus, warns cops – with knife gang targeting man walking home](https://www.thesun.co.uk/news/11113392/spike-coronavirus-asians-racist-attacks/) |
| The Sun | 3/3/2020 | [Singapore student, 23, battered in London street by four thugs shouting ‘I don’t want your coronavirus in my country’](https://www.thesun.co.uk/news/11086407/singapore-student-attacked-coronavirus-london/) |
| The Sun | 2/19/2020 | [Cops investigate five racist attacks linked to coronavirus as Asian student told ‘go back to your f****** country’](https://www.thesun.co.uk/news/10987569/cops-investigate-five-racist-attacks-coronavirus-asian-student/) |
| **Italy (Jan.23-Apr.23, 2020)** | | |
| **Newspaper** | **Date** | **News Title** |
| Corriere della Sera | 2/1/2020 | [Bimbi cinesi dormono a scuola](https://corrieredelveneto.corriere.it/veneto/cronaca/20_febbraio_01/bimbi-cinesi-dormono-scuolal-auto-quarantena-una-mamma-b9ca6e92-44bd-11ea-a20b-fbd8986ccd28.shtml) [Chinese children sleep in school. A mom’s self-quarantine] |
| Corriere della Sera | 2/6/2020 | [Io, italiana dagli occhi a mandorla Nel vortice dell’ansia collettiva](https://corrierefiorentino.corriere.it/firenze/notizie/cronaca/20_febbraio_06/io-italiana-occhi-mandorla-vortice-dell-ansia-collettiva-051a4350-48b9-11ea-960b-dc1d5deb3765.shtml) [Me, Italian with almond eyes, in the vortex of collective anxiety] |
| Corriere della Sera | 2/4/2020 | [«Io non sono un virus» e Firenze abbraccia il ragazzo cines](https://video.corrierefiorentino.corriere.it/io-non-sono-virus-firenze-abbraccia-ragazzo-cinese/6f76577a-474c-11ea-9503-d9eb4b836b9b)e [“I'm not a virus” and Florence hugs the Chinese boy] |
| Corriere della Sera | 1/30/2020 | [Coronavirus: studenti orientali sospesi conservatorio santa cecilia](https://roma.corriere.it/notizie/cronaca/20_gennaio_30/coronavirus-studenti-orientali-sospesi-conservatorio-santa-cecilia-6a7bbfe8-4342-11ea-bdc8-faf1f56f19b7.shtml) [Coronavirus: Oriental students blocked from santa cecilia conservatory] |
| Corriere della Sera | 2/21/2020 | [Solto collina, lettere di scuse del sindaco con i cinesi per il post sul virus](https://bergamo.corriere.it/notizie/cronaca/20_febbraio_21/solto-collina-lettere-scuse-sindaco-cinesi-il-post-virus-e3960392-54c3-11ea-9196-da7d305401b7.shtml) [Solto hill, letters of apology from the mayor to the Chinese for the post on the virus] |
| Corriere della Sera | 2/28/2020 | [Zaia: «Grazie all’igiene dei veneti solo 116 positivi, i cinesi? Li abbiamo visti tutti mangiare i topi vivi»](https://video.corriere.it/cronaca/zaia-noi-veneti-ci-facciamo-doccia-cinesi-li-abbiamo-visti-tutti-mangiare-topi-vivi/27549638-5a3d-11ea-afa8-e7dfdde6e2a2) [Zaia on coronavirus: Chinese eat live mice] |
| Corriere della Sera | 3/13/2020 | [Umanita convive sempre le epidemie ecco come proteggersi](https://www.corriere.it/sette/attualita/20_marzo_13/umanita-convive-sempre-le-epidemie-ecco-come-proteggersi-553ef5c2-61e8-11ea-9897-5c6f48cf812d.shtml) [Humanity has always lived with epidemics. Here’s how to protect] |
| Corriere della Sera | 3/3/2020 | [Essere Giusti, adesso, verso gli altri](https://www.corriere.it/cultura/20_marzo_03/essere-giusti-adesso-gli-altri-0f2fe6c0-5d7b-11ea-ad92-9d72350309c8.shtml) [Coronavirus, being righteous now towards others] |
| Corriere della Sera | 2/12/2020 | [Torino, due cinesi aggrediti in Borgo Vittoria: «Siete il virus, andate via»](https://torino.corriere.it/cronaca/20_febbraio_12/cinesi-aggrediti-due-italiani-portate-coronavirus-andate-via-63f15f82-4d71-11ea-a2de-b4f1441c3f82.shtml) [Turin, two Chinese attacked in Borgo Vittoria: “You are the virus, go away”] |
| Corriere della Sera | 2/20/2020 | [«Morissero solo i cinesi per il coronavirus»: Esti, sindaco di Solto Collina, scivola in rete](https://bergamo.corriere.it/notizie/cronaca/20_febbraio_05/morissero-solo-cinesi-il-coronavirus-esti-sindaco-solto-collina-scivola-rete-c3ab2f52-4781-11ea-bec1-6ac729c309c6.shtml) [“Only the Chinese would die from the coronavirus”: Esti, mayor of Solto Collina, slips on the net] |
| Corriere della Sera | 2/6/2020 | [«Teppismo razzista su un bimbo cinese». La denuncia di Merola.](https://corrieredibologna.corriere.it/bologna/cronaca/20_febbraio_06/teppismo-razzista-un-bimbo-cinese-denuncia-merola-d03ad23c-4910-11ea-960b-dc1d5deb3765.shtml) [“Racist hooliganism on a Chinese child.”] |
| Corriere della Sera | 2/18/2020 | [Coronavirus Italia, la prof cinese derissa in treno e il tweet virale](https://milano.corriere.it/notizie/cronaca/20_febbraio_18/coronavirus-italia-professoressa-cinese-lala-hu-derisa-treno-frecciarossa-tweet-virale-45a09b94-5279-11ea-ac26-d47429c3b2e0.shtml) [Coronavirus Italy, the Chinese prof comes by train and the viral tweet] |
| La Repubblica | 1/31/2020 | [Coronavirus: "Non entrate": il cartello in cinese in bar a Fontana di Trevi. Palumbo: "Ignobile"](https://roma.repubblica.it/cronaca/2020/01/31/news/coronavirus_spunta_cartello_in_cinese_a_fontana_di_trevi_non_entrate_-247246234/) [Coronavirus, “Do not enter”: the sign in Chinese in a bar at the Trevi Fountain. Palumbo: “Ignoble”] |
| La Repubblica | 4/10/2020 | [Razzismo e coronavirus: dall'Asia, all'Europa, agli Stati Uniti, la giostra di "scherzi linguistici" e battute ispirati dalla discriminazione](https://www.repubblica.it/solidarieta/diritti-umani/2020/04/10/news/razzismo-253645309/) [Racism and coronavirus: from Asia, to Europe, to the United States, the carousel of “jokes” and jokes inspired by discrimination] |
| La Repubblica | 1/26/2020 | [Turisti cinesi insultati e presi a sputi da banda di ragazzini a Venezia](https://www.repubblica.it/cronaca/2020/01/26/news/turisti_cinesi_insultati_e_presi_a_sputi_da_banda_di_ragazzini_a_venezia-246747810/) [Chinese tourists insulted and spit on by a gang of kids in Venice] |
| La Repubblica | 4/22/2020 | [La ragazza coreana che desiderava l'ottimismo](https://ricerca.repubblica.it/repubblica/archivio/repubblica/2020/04/22/la-ragazza-coreana-che-desiderava-lottimismoNapoli11.html?ref=search) [The Korean girl who wanted optimism] |
| La Repubblica | 4/20/2020 | [Più made in Italy e meno cinese: il delivery ai tempi del lockdown](https://www.repubblica.it/economia/rapporti/osserva-italia/osservacibo/2020/04/20/news/piu_made_in_italy_e_meno_cinese_il_delivery_ai_tempi_del_lockdown-254529160/?ref=search) [More made in Italy and less Chinese: delivery at the time of the lockdown] |
| La Repubblica | 2/4/2020 | ["Coronavirus, noi italiani di origini cinesi vittime di razzismo e pregiudizi"](https://torino.repubblica.it/cronaca/2020/02/04/news/_coronavirus_noi_italiani_di_origini_cinesi_vittime_di_razzismo_e_pregiudizi_-247584510/) [“Coronavirus, we Italians of Chinese origin are victims of racism and prejudice”] |
| **Germany (Jan.23-Apr.16, 2020)** | | |
| **Newspaper** | **Date** | **News Title** |
| Süddeutsche Zeitung | 4/10/2020 | [UN: Viren könnten als Terrorwaffe eingesetzt werden](vhttps:/www.sueddeutsche.de/gesundheit/krankheiten-un-viren-koennten-als-terrorwaffe-eingesetzt-werden-dpa.urn-newsml-dpa-com-20090101-200410-99-659366) [UN: Viruses could be used as a weapon of terrorism] |
| Süddeutsche Zeitung | 4/8/2020 | [Unbekannter greift Mundschutz-Träger mit Schlagring an](https://www.sueddeutsche.de/panorama/kriminalitaet-dresden-unbekannter-greift-mundschutz-traeger-mit-schlagring-an-dpa.urn-newsml-dpa-com-20090101-200408-99-636727) [Stranger attacks face mask wearers with brass knuckles] |
| Süddeutsche Zeitung | 3/23/2020 | [Mann sticht 53-Jährigen ohne erkennbaren Grund nieder](https://www.sueddeutsche.de/panorama/kriminalitaet-muenchen-mann-sticht-53-jaehrigen-ohne-erkennbaren-grund-nieder-dpa.urn-newsml-dpa-com-20090101-200323-99-437939) [Man stabs 53-year-olds for no apparent reason] |
| Süddeutsche Zeitung | 3/11/2020 | [Mann attackiert Chinesin mit Desinfektionsmittel](https://www.sueddeutsche.de/panorama/kriminalitaet-muenchen-mann-attackiert-chinesin-mit-desinfektionsmittel-dpa.urn-newsml-dpa-com-20090101-200311-99-287042) [Man attacks Chinese woman with disinfectant] |
| Süddeutsche Zeitung | 3/8/2020 | [Das Virus bringt das Verdrängte zurück](https://www.sueddeutsche.de/kultur/corona-angst-china-kolonialismus-1.4834324) [The virus brings back what has been repressed] |
| Süddeutsche Zeitung | 3/5/2020 | [Verein: Diskriminierung asiatischstämmiger Menschen nimmt zu](https://www.sueddeutsche.de/gesundheit/krankheiten-tuebingen-verein-diskriminierung-asiatischstaemmiger-menschen-nimmt-zu-dpa.urn-newsml-dpa-com-20090101-200305-99-203529) [Association: Discrimination against people of Asian origin is increasing] |
| Süddeutsche Zeitung | 3/2/2020 | [Angst vor Coronavirus: Leipzig-Ordner schicken Japaner aus dem Stadion](https://www.sueddeutsche.de/sport/rb-leipzig-angst-vor-coronavirus-leipzig-ordner-schicken-japaner-aus-dem-stadion-1.4828919) [Fear of corona virus: Leipzig folders send Japanese out of the stadium] |
| Süddeutsche Zeitung | 2/12/2020 | [Spahn: Gut gerüstet gegen das Virus](https://www.sueddeutsche.de/politik/corona-spahn-gut-geruestet-gegen-das-virus-1.4795510) [Spahn: Well prepared against the virus] |
| Frankfurter Allgemeine Zeitung | 2/14/2020 | [Maas warnt vor Rassismus wegen neuem Virus](https://www.faz.net/aktuell/gesellschaft/gesundheit/coronavirus/heiko-maas-warnt-vor-rassismus-wegen-neuem-coronavirus-16633610.html) [Mass warns of racism because of new virus] |
| Frankfurter Allgemeine Zeitung | 2/3/2020 | [Wie Rassisten das Coronavirus für sich nutzen](https://www.faz.net/aktuell/gesellschaft/gesundheit/coronavirus/coronavirus-in-china-sinophobie-und-rassismus-im-netz-16614102.html) [How racists use the coronavirus for themselves] |
| Welt | 2/3/2020 | [Die „gelbe Gefahr“ ist zurück](https://www.welt.de/debatte/kommentare/article205543151/Rassismus-Die-gelbe-Gefahr-ist-zurueck.html) [The “yellow danger” is back] |
| **Australia (Jan.23-Apr.21, 2020)** | | |
| **News Site** | **Date** | **News Title** |
| ABC | 4/3/2020 | [Australians urged to ‘show kindness’ amid reports of COVID-19 racial discrimination complaints](https://www.abc.net.au/news/2020-04-03/racism-covid-19-coronavirus-outbreak-commissioner-discrimination/12117738) |
| ABC | 2/1/2020 | [Coronavirus has sparked racist attacks on Asians in Australia — including me](https://www.abc.net.au/news/2020-02-01/coronavirus-has-sparked-racist-attacks-on-asian-australians/11918962) |
| ABC | 4/9/2020 | [As coronavirus sparks anti-Chinese racism, xenophobia rises in China itself](https://www.abc.net.au/news/2020-04-09/coronavirus-intensifies-anti-foreigner-sentiment-in-china/12128224) |
| ABC | 4/15/2020 | [Coronavirus fears prompting racially motivated offences against Queensland's Chinese community, police say](https://www.abc.net.au/news/2020-04-15/coronavirus-queensland-racially-motivated-offence-rise-chinese/12148476) |
| ABC | 4/21/2020 | [Racist coronavirus graffiti sprayed on Chinese-Australian family’s home in Melbourne](https://www.abc.net.au/news/2020-04-22/racist-coronavirus-graffiti-sprayed-on-family-home-in-melbourne/12170162) |
| ABC | 2/24/2020 | [Fake news, censorship, coronavirus and racism: the testy relationship between China and Australia fires up Q+A](https://www.abc.net.au/news/2020-02-25/wang-xining-china-accusations-denials-and-racism-on-q+a/11996474) |
| ABC | 2/20/2020 | [How Sinophobia goes viral: Building resilience against Australia’s latest anti-Chinese contagion](https://www.abc.net.au/religion/andrew-jakubowicz-sinophobia-goes-viral/11983714) |
| ABC | 2/2/2020 | [My story of racism and discrimination in Australia](https://www.abc.net.au/everyday/the-count-and-the-challenges-of-being-in-the-minority/11897466) |
| ABC | 4/8/2020 | [South Australian councillor spat at in racist coronavirus attack](https://www.abc.net.au/news/2020-04-08/salisbury-councillor-targeted-in-racist-coronavirus-attack/12133078) |
| ABC | 4/13/2020 | [COVID-19-branded racist vandalism appears as second Nazi flag pops up in regional Victoria](https://www.abc.net.au/news/2020-04-13/coronavirus-racism-blamed-nazi-flag-appears-in-regional-victoria/12145166) |
| ABC | 2/4/2020 | [Coronavirus travel ban sees Chinese students miss start of university, Australian tertiary education sector scrambling](https://www.abc.net.au/news/2020-02-04/coronavirus-scare-sees-chinese-students-miss-uniniversity/11929948) |
| ABC | 2/6/2020 | [Racist coronavirus event at Melbourne nightclub Pawn & Co cancelled after backlash](https://www.abc.net.au/news/2020-02-06/racist-coronavirus-event-mr-chans-pawn-and-co-melbourne/11934426) |
| ABC | 3/20/2020 | [Hong Kong student accused of having coronavirus was ‘punched for wearing a face mask’](https://www.abc.net.au/news/2020-03-20/coronavirus-hong-kong-student-assaulted-for-wearing-face-mask/12075470) |
| News.com | 3/3/2020 | [‘Disgusting and sickening’: Virus challenge Australia is failing](https://www.news.com.au/national/politics/disgusting-and-sickening-virus-challenge-australia-is-failing/news-story/f340fac9937c4117da579d9c56248789) |
| 7NEWS | 3/10/2020 | [Man filmed ‘intimidating’ Asian people at Brisbane Airport in apparent coronavirus-fueled racism](https://7news.com.au/lifestyle/health-wellbeing/man-filmed-intimidating-asian-people-at-brisbane-airport-in-apparent-coronavirus-fueled-racism-c-737966) |
| 7NEWS | 4/21/2020 | [Scott Morrison condemns ‘anti-Asian’ behaviour in coronavirus crisis](v) |
| 7NEWS | 2/11/2020 | [Coronavirus ‘racism’ won’t be tolerated, say Scott Morrison and Anthony Albanese](https://7news.com.au/travel/coronavirus-racism-wont-be-tolerated-say-scott-morrison-and-anthony-albanese--c-691859) |
| 7NEWS | 2/6/2020 | [Racist graffiti left on driveway of Perth family home in wake of coronavirus scare](https://7news.com.au/travel/coronavirus/racist-graffiti-left-on-driveway-of-perth-family-home-in-wake-of-coronavirus-scare-c-686592) |
| 7NEWS | 4/17/2020 | [Coronavirus: University of Melbourne international students assaulted in unprovoked racist attack](https://7news.com.au/lifestyle/health-wellbeing/coronavirus-university-of-melbourne-international-students-assaulted-in-unprovoked-racist-attack-c-983675) |
| **Japan (Jan.23-Apr.18, 2020)** | | |
| **Newspaper** | **Date** | **News Title** |
| 読売新聞 (Yomiuri Shimbun) | --- | --- |
| 朝日新聞 (Asahi Shimbun) | 2/10/2020 | [新型コロナウイルスの流行で露わになった「世界の人種差別」](https://globe.asahi.com/article/13108689) (“Racism in the world” revealed by the epidemic of the new coronavirus) |
| 朝日新聞 (Asahi Shimbun) | 1/30/2020 | [「中国人ウイルスとみなさないで」隔離求める署名が波紋](https://www.asahi.com/articles/ASN1Z42K2N1ZUHBI015.html) (“Don’t consider it a Chinese virus” signatures calling for quarantine ripple) |
| 朝日新聞 (Asahi Shimbun) | 3/9/2020 | [新型コロナ、病名の背景に「地名はダメ」のルールと事情](https://www.asahi.com/articles/ASN367HMKN36UBQU007.html?iref=pc_ss_date_article) (New corona, rules and circumstances of “place name is not good” in the background of disease name) |
| 朝日新聞 (Asahi Shimbun) | 2/20/2020 | [「コロナ消え失せろ」　パリ郊外の日本料理店に落書き](https://www.asahi.com/sp/articles/ASN2N34K1N2NUHBI007.html?iref=sp_ss_date_article)(“Disappear Corona” doodle at a Japanese restaurant on the outskirts of Paris) |
| 毎日新聞 (Mainichi Shimbun) | --- | --- |
| **South Korea (Jan.23-Apr.16, 2020)** | | |
| **Newspaper** | **Date** | **News Title** |
| 조선일보 (Chosun Ilbo) | 2/9/2020 | [美 샌프란서 亞계 노인 대상 ‘묻지마’ 공격 빈발…’펜데믹 中혐오’ 추정](https://biz.chosun.com/site/data/html_dir/2021/02/09/2021020902263.html%20) (Frequent “don't ask” attacks on senior citizens of San Francisco in the United States... ‘Pandemic Chinese hate’ presumption) |
| 조선일보 (Chosun Ilbo) | 1/29/2020 | [택시들 中관광객 거부… 배민노조는 "중국인 많은 지역 배달 중단"](https://www.chosun.com/site/data/html_dir/2020/01/29/2020012900214.html) (Taxi refusal of Chinese tourists... Baemin’s Union “Stops Delivery to Many Chinese People”) |
| 조선일보 (Chosun Ilbo) | 1/30/2020 | [아시아인들 "난 바이러스가 아니다" 해시태그 운동](https://www.chosun.com/nsearch/?query=%EC%A4%91%EA%B5%AD%EC%9D%B8%EC%97%90%20%EB%8C%80%ED%95%9C%20%EC%B0%A8%EB%B3%84&siteid=&sort=1&date_period=all&writer=&field=&emd_word=&expt_word=&opt_chk=false&app_check=0) (Asians “I’m not a virus” hashtag movement) |
| 조선일보 (Chosun Ilbo) | 1/31/2020 | [황교익, 설현 소환하며 中혐오 문제 비판 "한국인도 박쥐 먹어왔다"](https://www.chosun.com/site/data/html_dir/2020/01/31/2020013101127.html) (Hwang Gyo-ik summons Seol-hyun and criticizes China’s hate issue “Koreans have also eaten bats”) |
| 조선일보 (Chosun Ilbo) | 1/31/2020 | [우한폐렴 이후 세계서 들끓는 ‘동양인 차별’… “욕하고 침뱉고”](https://www.chosun.com/site/data/html_dir/2020/01/31/2020013101797.html) (“Asian discrimination” in the world after Wuhan pneumonia... “Curse and spit”) |
| 조선일보 (Chosun Ilbo) | 1/31/2020 | [[종합]황교익, 설현 박쥐 먹방 언급→극우 언론·정치인 비판…"혐오 정서 퍼뜨리지말길" (전문)](https://www.chosun.com/site/data/html_dir/2020/01/31/2020013102105.html) (Criticism of far-right media and politicians “Do not spread hateful emotions”) |
| 조선일보 (Chosun Ilbo) | 1/31/2020 | [[SC이슈] "中무분별한 혐오NO!"..황교익, 뜬금없는 설현 소환](https://www.chosun.com/site/data/html_dir/2020/01/31/2020013102155.html) ([SC Issue] “Chinese Indiscriminate Hate NO!”.. Hwang Gyo-ik summons Seolhyun) |
| 조선일보 (Chosun Ilbo) | 1/31/2020 | [[종합] "설현 소환→정치 음모 제기"...황교익, '中혐오' 날선 비판에 설전ing](https://www.chosun.com/site/data/html_dir/2020/01/31/2020013102497.html) ([General] “Summon Seolhyun → Political Conspiracy Raised”...) |
| 조선일보 (Chosun Ilbo) | 1/31/2020 | [황교익 "극우 언론, '박쥐 먹는 중국인'으로 혐오 정서 유발…한국 정부 혐오까지 부추겨"[전문]](https://www.chosun.com/site/data/html_dir/2020/01/31/2020013102409.html) (Hwang Gyo-ik “The far-right media,‘bat-eating Chinese’ triggered hate sentiment... even the South Korean government incited hatred”) |
| 조선일보 (Chosun Ilbo) | 1/31/2020 | ["설현 박쥐 먹방은 왜.." 황교익, 언급 논란→'中혐오 중단' 주장ing [종합]](https://www.chosun.com/site/data/html_dir/2020/01/31/2020013102828.html) (“Why is Seolhyun bat eating...”) |
| 조선일보 (Chosun Ilbo) | 1/31/2020 | ["中 혐오 멈춰야" 황교익, '설현 박쥐 먹방'만? 신종 코로나언급 이유 (전문)[종합]](https://www.chosun.com/site/data/html_dir/2020/01/31/2020013103121.html) (“Stop hatred of China” Hwang Gyo-ik, only ‘Seolhyun Bat Meokbang’? Reasons for mentioning new coronavirus) |
| 조선일보 (Chosun Ilbo) | 2/1/2020 | [한국인 등 동양인 수업 참석 금지… 우한 폐렴 빌미로 인종차별 확산](https://www.chosun.com/site/data/html_dir/2020/02/01/2020020100161.html) (Prohibition of attending classes for Asians such as Koreans… Racial discrimination spread under the guise of Wuhan pneumonia) |
| 조선일보 (Chosun Ilbo) | 2/5/2020 | [인권위원장 “신종코로나 확산, 혐오 아닌 인류애·연대로 대처해야”](https://www.chosun.com/site/data/html_dir/2020/02/05/2020020501633.html) (Chairman of Human Rights Committee “We need to deal with the spread of new coronas and humanity and solidarity rather than hate”) |
| 조선일보 (Chosun Ilbo) | 2/5/2020 | [中여성 독일서 욕설·폭행 당해… 韓대사관 "안전 주의" 긴급공지](https://www.chosun.com/site/data/html_dir/2020/02/05/2020020501640.html) (Chinese woman was abused and assaulted in Germany... Emergency Notice of “Safety Caution” by the Korean Embassy) |
| 조선일보 (Chosun Ilbo) | 2/10/2020 | [독일 유명배우, 중국인 세입자 내쫓아… 전염되는 ‘중국인 혐오’](https://www.chosun.com/site/data/html_dir/2020/02/13/2020021300581.html) (Famous German actor kicks out Chinese tenants... Contagious “Chinese hate”) |
| 조선일보 (Chosun Ilbo) | 2/12/2020 | [[기자의 시각] '오사카 폐렴'이었다면](https://www.chosun.com/site/data/html_dir/2020/02/11/2020021103979.html) (Reporter's Perspective] If it was ‘Osaka pneumonia’) |
| 조선일보 (Chosun Ilbo) | 2/13/2020 | [승차 거부·욕설·폭행… 유럽의 日常이 된 동양인 차별](https://www.chosun.com/site/data/html_dir/2020/02/10/2020021000207.html) (Refusal to ride, profanity, and assault... Discrimination of Asians, which has become the Japanese leader in Europe) |
| 조선일보 (Chosun Ilbo) | 3/18/2020 | [트럼프 "코로나는 중국 바이러스", 中 양제츠 "중국에 먹칠하면 반격"](https://www.chosun.com/site/data/html_dir/2020/03/18/2020031800259.html) (Trump “Corona is a Chinese virus”, Chinese Yangzetsu “fight against China”) |
| 조선일보 (Chosun Ilbo) | 3/19/2020 | [美 공화당 중진 "코로나 사태 책임은 뱀·박쥐·개 먹는 중국에" 발언 논란](https://www.chosun.com/site/data/html_dir/2020/03/19/2020031906305.html) (Controversy about remarks by US Republican leaders “China is responsible for eating snakes, bats and dogs”) |
| 조선일보 (Chosun Ilbo) | 3/21/2020 | [‘마스크 착용’ 제레미 린, CBA 복귀 위해 베이징 입성](https://www.chosun.com/site/data/html_dir/2020/03/21/2020032101205.html) (Jeremy Lin “wearing a mask” enters Beijing to return to CBA) |
| 조선일보 (Chosun Ilbo) | 3/22/2020 | [우레이의 코로나19 확진→아시아 선수 차별 분위기 '스멀스멀'](https://www.chosun.com/site/data/html_dir/2020/03/22/2020032200055.html) (Corona 19 confirmed by Wooray → Asian players being discriminated against) |
| 조선일보 (Chosun Ilbo) | 3/24/2020 | [트럼프 "코로나 확산 아시아계 미국인 잘못 아냐"](https://www.chosun.com/site/data/html_dir/2020/03/24/2020032400744.html) (Trump “It’s not wrong to spread the corona Asian Americans”) |
| 조선일보 (Chosun Ilbo) | 3/24/2020 | [존 조대니얼 대 킴, 코로나19 동양인 차별 비판.."멍청이들"[종합]](https://www.chosun.com/site/data/html_dir/2020/03/24/2020032405397.html) (John Jo Daniel vs. Kim, Criticizes Discrimination of Asians for Corona 19..) |
| 조선일보 (Chosun Ilbo) | 3/24/2020 | [[종합]한국계 존 조·대니얼 대 킴, 코로나19 확산→인종차별에 맹비난 "멍청이들"](https://www.chosun.com/site/data/html_dir/2020/03/24/2020032405588.html) ([General] Korean-born John Jo and Daniel vs. Kim, spread of Corona 19 → “dumbs” criticized for racial discrimination) |
| 조선일보 (Chosun Ilbo) | 4/7/2020 | [트럼프의 '차이나 바이러스'에 린 이어 콴도 반발..."인종 차별 심해져"](https://www.chosun.com/site/data/html_dir/2020/04/07/2020040701264.html) (Trump’s ‘China Virus’ followed by Lin and Kwan’s backlash...“Racial discrimination intensifies”) |
| 중앙 일보 (Joonhang Illbo) | 2/2/2020 | [[송준석 칼럼] 코로나바이러스와 인종차별](http://m.ch.koreadaily.com/news/read.asp?art_id=8179045) ([Song Joon-seok’s Column] Coronavirus and Racism) |
| 중앙 일보 (Joonhang Illbo) | 3/27/2020 | [[아름다운 우리말] 김칫국 주례사](http://m.ny.koreadaily.com/news/read.asp?art_id=6360509&category=opinion) ([Beautiful Korean] Kim Chit-guk Juryesa) |
| 중앙 일보 (Joonhang Illbo) | 2/21/2020 | [욕시 인종차별 승차거부 택시 신고 캠페인 시작](http://m.sf.koreadaily.com/news/read.asp?art_id=8041327) (Started a campaign to report racist racism refused to ride a taxi) |
| 동아일보 (Donga Ilbo) | 1/28/2020 | [세계를 뒤덮은 ‘우한 폐렴’ 공포… 중국인 여행자 봉변도](https://www.donga.com/news/article/all/20200128/99415778/1) (The fear of “Wuhan pneumonia” covering the world… Chinese tourist Bongbyondo) |
| 동아일보 (Donga Ilbo) | 1/30/2020 | [“중국인으로 오해받아 기침만 해도 눈총”](https://www.donga.com/news/article/all/20200130/99453157/1) (“Because of being misunderstood as a Chinese, even coughing is glorious”) |
| 동아일보 (Donga Ilbo) | 1/31/2020 | [[e글e글] 황교익, ‘박쥐 먹방’ 설현 ‘강제 소환’…후폭풍](https://www.donga.com/news/article/all/20200131/99481249/2) (Hwang Gyoik, “Bat Meokbang” Seolhyun “Forced Summoning”… After storm) |
| 동아일보 (Donga Ilbo) | 1/31/2020 | [中 관광객에 침 세례…유럽서 ‘동양인 혐오’ 확산](https://www.donga.com/news/article/all/20200131/99482688/1) (Baptism of Chinese tourists... 'Oriental hatred' spread in Europe) |
| 동아일보 (Donga Ilbo) | 2/3/2020 | [혐오의 칼날[내가 만난 名문장]](https://www.donga.com/news/article/all/20200203/99504926/1) (Blade of hate [name sentence I met]) |
| 동아일보 (Donga Ilbo) | 2/3/2020 | [‘메이드 인 차이나’…獨 언론 신종 코로나 표지에 中 항의](https://www.donga.com/news/article/all/20200203/99516610/2) (“Made in China”… China protests against the cover of the new coronavirus in the media) |
| 동아일보 (Donga Ilbo) | 2/4/2020 | [‘反아시아 바이러스’[횡설수설/김영식]](https://www.donga.com/news/article/all/20200204/99522290/1) (“Anti-Asian Virus” [Gibbe/Kim Young-sik]) |
| 동아일보 (Donga Ilbo) | 2/4/2020 | [중국 유학생 “지하철서 말하면 눈치…기숙사 같이 살기 싫다고 해”](https://www.donga.com/news/article/all/20200204/99534962/1) (Chinese student “When I talk about the subway, I notice… They say they don’t want to live like a dorm.”) |
| 동아일보 (Donga Ilbo) | 2/5/2020 | [신종 코로나에 아시아 혐오 기승…유엔도 나서 ‘차별 철폐’](https://www.donga.com/news/article/all/20200205/99555531/1) (Asian hate rises in the new corona... The United Nations also stepped up to “Abolish Discrimination”) |
| 동아일보 (Donga Ilbo) | 2/10/2020 | [“박쥐나 먹으니 바이러스에 걸리지”…유럽의 도넘은 中혐오](https://www.donga.com/news/article/all/20200210/99617637/1) (“Eating bats makes you get a virus”… European hatred of China) |
| 동아일보 (Donga Ilbo) | 2/17/2020 | [세계적 골키퍼 부폰, 中 기자에게 코로나 농담했다가 뭇매](https://www.donga.com/news/article/all/20200217/99736875/1) (World-class goalkeeper Buffon made a joke about Corona to a Chinese reporter) |
| 동아일보 (Donga Ilbo) | 2/21/2020 | 유럽 여행에서 겪은 일[카버의 한국 블로그] (What happened on a trip to Europe [Carver's Korean Blog]) |
| 동아일보 (Donga Ilbo) | 3/4/2020 | 中매체 “러시아 내 중국인들 강제 추방 등 부당한 차별 받아” (Chinese “received unfair discrimination such as forced deportation of Chinese people in Russia”) |
| 동아일보 (Donga Ilbo) | 4/9/2020 | “동양인 쓸어버릴 것” SNS ‘총격 암시글’…美 혐오 ‘위험수위’ (“I will wipe out the Asians” SNS “Suggestions for shooting”… US hate ‘dangerous level’) |
| **Russia (Jan.23-Apr.20, 2020)** | | |
| **Newspaper** | **Date** | **News Title** |
| Komsomolskaya Pravda | --- | --- |
| Russian Newspaper | 3/16/2020 | [Свобода слова - не повод для расизма](https://rg.ru/2020/03/16/svoboda-slova-ne-povod-dlia-rasizma.html) (Freedom of speech is not a reason for racism) |
| Russian Newspaper | 2/25/2020 | [В Корее из-за коронавируса появились рестораны "только для корейцев"](https://rg.ru/2020/02/25/v-koree-iz-za-koronavirusa-poiavilis-restorany-tolko-dlia-korejcev.html) (“Korean only” restaurants open in Korea due to coronavirus) |
| Russian Newspaper | 4/6/2020 | [Американцы китайского происхождения столкнулись с расизмом из-за COVID-2019](https://rg.ru/2020/04/06/amerikancy-kitajskogo-proishozhdeniia-stolknulis-s-rasizmom-iz-za-covid-2019.html) (Chinese Americans face racism over COVID-2019) |
| Russian Newspaper | 3/18/2020 | [Эксперты осудили использование выражения "китайский вирус"](https://rg.ru/2020/03/18/eksperty-osudili-ispolzovanie-vyrazheniia-kitajskij-virus.html) (Experts condemn the use of the expression “Chinese virus”) |
| Novaya Gazeta | 2/1/2020 | [«В лифте едем не дыша»](https://novayagazeta.ru/articles/2020/02/01/83730-v-lifte-edem-ne-dysha) (“We are not breathing in the elevator”) |
| **Peru (Jan.23-Apr.18, 2020)** | | |
| **Newspaper** | **Date** | **News Title** |
| El Comercio | 3/4/2020 | [Anna, de padres chinos, llevó comida para compartir con sus compañeros, nadie la aceptó por miedo al covid-19](https://www.elcomercio.com/tendencias/discriminacion-nina-china-argentina-covid19.html) (Anna, of Chinese parents, brought food to share with her colleagues, no one accepted it for fear of covid-19) |
| El Comercio | 2/14/2020 | [El FC Barcelona muestra su solidaridad con China por la crisis del coronavirus](https://www.elcomercio.com/deportes/futbol-barcelona-solidaridad-china-coronavirus.html) (FC Barcelona shows its solidarity with China due to the coronavirus crisis) |
| El Comercio | 3/4/2020 | [Coronavirus en Argentina: Bullying contra hija de chinos por ofrecer budín en escuela primaria](https://elcomercio.pe/mundo/latinoamerica/coronavirus-en-argentina-anna-l-hija-de-comerciantes-chinos-es-victima-de-cruel-bullying-por-ofrecer-budin-a-sus-companeros-en-escuela-primaria-chubut-trelew-noticia/) (Coronavirus in Argentina: Bullying against daughter of Chinese for offering pudding in elementary school) |
| El Comercio | 3/27/2020 | [El discurso de odio contra China creció de manera alarmante en Twitter por el coronavirus](https://www.elcomercio.com/actualidad/discurso-odio-china-covid19-twitter.html) (Hate speech against China grew alarmingly on Twitter due to coronavirus) |
| La Republica Peru | 3/23/2020 | [“Llévate tu coronavirus”: sujeto insulta a joven china y golpea a la amiga que intentó defenderla](https://larepublica.pe/mundo/2020/02/23/inglaterra-llevate-tu-coronavirus-sujeto-insulta-a-joven-y-golpea-a-la-amiga-que-intento-defenderla-ataque-agresion-coronavirus-violencia-rddr/) (“Llévate tu coronavirus”: sujeto insulta a joven china y golpea a la amiga que intentó defenderla) |
| La Republica Peru | 3/6/2020 | [El coronavirus y la xenofobia, una peligrosa combinación](https://larepublica.pe/mundo/2020/03/07/coronavirus-consecuencias-oms-el-coronavirus-y-la-xenofobia-una-peligrosa-combinacion-xenofobia-onu-atmp/) (The coronavirus and xenophobia, a dangerous combination) |
| La Republica Peru | 3/25/2020 | [Carmen Salinas se disculpa por comentarios racistas: “Nunca tuve la intención de dañar”](https://larepublica.pe/espectaculos/2020/03/25/carmen-salinas-se-disculpa-por-comentarios-racistas-contra-china-nchs/) (Carmen Salinas apologizes for racist comments: “I never intended to harm”) |
| La Republica Peru | 3/30/2020 | [El discurso de odio contra China creciun 900% en Twitter a causa de la pandemia](https://www.larepublica.co/internet-economy/el-discurso-de-odio-contra-china-crecio-un-900-en-twitter-a-causa-de-la-pandemia-2985336) (Hate speech against China grew 900% on Twitter due to the pandemic) |
| La Republica Peru | 3/11/2020 | [Joven de origen asiático queda postrado en hospital tras sufrir ataque racista por el coronavirus](https://larepublica.pe/mundo/2020/03/11/coronavirus-joven-asiatico-queda-postrado-en-el-hospital-tras-sufrir-ataque-racista-por-el-covid-19-espana-thomas-siu-discriminacion-rddr/) (Young man of Asian origin is bedridden in hospital after suffering a racist attack by the coronavirus) |
| **France (Jan.23-Apr.20, 2020)** | | |
| **Newspaper** | **Date** | **News Title** |
| Le Monde | 4/9/2020 | [Coronavirus: aux Etats-Unis, les Asiatiques, victimes de discrimination, contre-attaquent](https://www.lemonde.fr/international/article/2020/04/09/coronavirus-aux-etats-unis-victimes-de-discrimination-les-asiatiques-contre-attaquent_6036105_3210.html) (Coronavirus: In the United States, Asians, victims of discrimination, fight back) |
| Le Monde | 1/30/2020 | [« Garde ton virus, sale Chinoise ! » : avec le coronavirus, le racisme antiasiatique se propage en France](https://www.lemonde.fr/planete/article/2020/01/29/coronavirus-le-racisme-antiasiatique-se-propage-en-france_6027701_3244.html) (“Keep your virus, dirty Chinese!”: With the coronavirus, anti-Asian racism is spreading in France) |
| Le Figaro | 1/30/2020 | [«Rentre chez toi, garde ta maladie!»: quand le coronavirus sert d’excuse au racisme antiasiatique](https://www.lefigaro.fr/actualite-france/rentre-chez-toi-garde-ta-maladie-quand-le-coronavirus-sert-d-excuse-au-racisme-antiasiatique-20200130) (“Go home, keep your illness!”: When the coronavirus is used as an excuse for anti-Asian racism) |
| Le Figaro | 2/6/2020 | [Coronavirus : un restaurant vietnamien à Prague refuse l'entrée aux clients chinois](https://www.lefigaro.fr/flash-actu/un-restaurant-vietnamien-a-prague-refuse-l-entree-aux-clients-chinois-a-cause-de-coronavirus-20200206) (Coronavirus: Vietnamese restaurant in Prague denies entry to Chinese customers) |
| Le Figaro | 2/8/2020 | [La crise du coronavirus ressuscite les clichés autour de la nourriture chinoise](https://www.lefigaro.fr/conso/la-crise-du-coronavirus-ressuscite-les-cliches-autour-de-la-nourriture-chinoise-20200208) (Coronavirus crisis resuscitates clichés around Chinese food) |
| Le Figaro | 2/3/2020 | [EN DIRECT - Coronavirus: «Pas d'épidémie en France», rassure le directeur de la Santé: Asiatiques stigmatisés: un phénomène récurrent de boucs émissaires liés aux épidémies](https://www.lefigaro.fr/sciences/2020/02/03/01008-20200203LIVWWW00001-en-direct-coronavirus-chine-epidemie-.php) (LIVE - Coronavirus: “No epidemic in France”, reassures the Director of Health: Stigmatized Asians: a recurring scapegoat phenomenon linked to epidemics) |
| Le Figaro | 2/19/2020 | [EN DIRECT - Coronavirus : «Quelques dizaines» de Français rapatriés de Wuhan en fin de semaine](https://www.lefigaro.fr/sciences/2020/02/19/01008-20200219LIVWWW00001-en-direct-coronavirus-chine-epidemie-diamond-princess-oms-.php) (LIVE - Coronavirus: “A few dozen” French repatriated from Wuhan at the end of the week) |
| Le Parisien | 1/28/2020 | [«Ils vont nous contaminer» : quand le coronavirus ravive la stigmatisation des Asiatiques en France](v) (“They will infect us”: when the coronavirus revives the stigmatization of Asians in France) |
| Le Parisien | 2/24/2020 | [Coronavirus : SOS Racisme lance une campagne contre les préjugés anti-asiatique](v) (Coronavirus: SOS Racism launches a campaign against anti-Asian prejudices) |
| Le Parisien | 1/31/2020 | [Coronavirus : le racisme antichinois jusque dans les cours de récré](https://www.leparisien.fr/societe/coronavirus-le-racisme-antichinois-jusque-dans-les-cours-de-recre-31-01-2020-8249106.php) (Coronavirus: anti-Chinese racism even in the playgrounds) |
| Le Parisien | 2/2/2020 | [Coronavirus : de jeunes Français d’origine asiatique pris à partie en Seine-et-Marne](https://www.leparisien.fr/seine-et-marne-77/coronavirus-de-jeunes-francais-d-origine-asiatique-pris-a-partie-en-seine-et-marne-02-02-2020-8250627.php) (Coronavirus: young French of Asian origin taken to task in Seine-et-Marne) |
| **Singapore (Jan.23-Apr.17, 2020)** | | |
| **Newspaper** | **Date** | **News Title** |
| The Straits Times | 2/7/2020 | [Discrimination against Chinese a virus: China Daily, Asia …](https://www.straitstimes.com/asia/discrimination-against-chinese-a-virus-china-daily) |
| The Straits Times | 3/30/2020 | [Sharon Au told to ‘go back to China’ in racist encounter in France](https://www.straitstimes.com/lifestyle/in-france-sharon-au-told-to-go-back-to-china) |
| The Straits Times | 3/28/2020 | [Coronavirus scare prompts racist attacks on ‘Chinese-looking’ Indians](https://www.straitstimes.com/asia/south-asia/coronavirus-scare-prompts-racist-attacks-on-chinese-looking-indians) |
| The Straits Times | 2/7/2020 | [Coronavirus: Chinese community in Australia complain of racism as MPs call for calm](https://www.straitstimes.com/asia/australianz/coronavirus-chinese-community-in-australia-complain-of-racism-as-mps-call-for-calm) |
| The Straits Times | 2/14/2020 | [Attacked for wearing face mask: Anti-Asian prejudice bubbles up in California over coronavirus fears](https://www.straitstimes.com/world/united-states/fake-flyers-and-face-mask-fear-california-fights-coronavirus-discrimination) |
| The Straits Times | 3/6/2020 | [London police arrest 2 teenagers linked to Covid-19 racist assault on Singaporean student](https://www.straitstimes.com/singapore/london-police-arrest-2-teenagers-linked-to-covid-19-racist-assault-on-singaporean-student) |
| The Straits Times | 4/1/2020 | [Asian Americans tell harrowing stories of abuse amid of abuse amid coronavirus outbreak](https://www.straitstimes.com/world/united-states/asian-americans-tell-harrowing-stories-of-abuse-amid-coronavirus-outbreak-in-the) |
| The Straits Times | 3/29/2020 | [Mistaken as Chinese, Indian woman forced into quarantine over coronavirus fears](https://www.straitstimes.com/asia/south-asia/mistaken-as-chinese-indian-woman-forced-into-quarantine-over-virus-fears) |
| The Straits Times | 3/7/2020 | [More shock than anger: Singaporean student opens up about Covid-19 racist attack in London](https://www.straitstimes.com/singapore/singaporean-student-in-london-seeks-eyewitnesses-after-coronavirus-related-taunt-and) |
| The Straits Times | 3/9/2020 | [Train rider in US sprays Asian passenger with air freshener amid Covid-19 fears](https://www.straitstimes.com/search?searchkey=discrimination%20Chinese) |
| The Straits Times | 2/5/2020 | [German and other European media fan coronavirus fears and Sinophobia](https://www.straitstimes.com/world/europe/german-and-other-european-media-fan-coronavirus-fears-and-sinophobia) |
| 联合早报 (Lianhe Zaobao) | 3/3/2020 | [疑因冠病疫情受歧视 新加坡学生伦敦街头被打成重伤](https://www.zaobao.com/realtime/singapore/story20200303-1033834) (Singaporean student in London got discriminated and beat up due to COVID) |
| 联合早报 (Lianhe Zaobao) | 2/12/2020 | [“COVID-19”传染病命名之乱](https://www.zaobao.com/znews/singapore/story20200212-1028424) (The messy business of naming “COVID-19”) |
| 联合早报 (Lianhe Zaobao) | 3/7/2020 | [张思浓：冠病疫情与德媒眼中的中国](https://www.zaobao.com/zopinions/views/story20200307-1035022) (Sinong Zhang: Coronavirus and China in German media) |
| 联合早报 (Lianhe Zaobao) | 3/26/2020 | [日副首相踢爆意代表曾呛：黄种人才会得冠病](https://www.zaobao.com/znews/others/story20200326-1040346) (Japan’s vice minister rebukes Italian remark: “only Asians are infected”) |
| 联合早报 (Lianhe Zaobao) | 3/29/2020 | [欧菁仙因疫情遭歧视](https://www.zaobao.com.sg/zentertainment/celebs/story20200329-1041064) (Jingxian Ou was discriminated due to COVID) |
| 联合早报 (Lianhe Zaobao) | 3/13/2020 | [交流站：对抗疫情不应戴上种族有色眼镜](https://www.zaobao.com.sg/forum/views/talk/story20200313-1036677) (Racism is not the cure to COVID) |
| 联合早报 (Lianhe Zaobao) | 3/4/2020 | [新加坡学生在伦敦街头疑因疫情被打成重伤 维文面簿留言慰问](https://www.zaobao.com.sg/news/singapore/story20200304-1033972) (Singaporean student victimized by racist attacks: warm messages on social media) |
| 联合早报 (Lianhe Zaobao) | 1/30/2020 | [新移民家长担心孩子被歧视 希望学校重视心理教育](https://www.zaobao.com.sg/news/singapore/story20200130-1024738) (Singaporean new immigrant parents worry about racism at school) |
| 联合早报 (Lianhe Zaobao) | 3/19/2020 | [特朗普：“中国病毒”说法没错中国未更早通报疫情](https://www.zaobao.com.sg/realtime/world/story20200319-1038360https:/www.zaobao.com.sg/realtime/world/story20200319-1038360) (Trump: “Chinese virus” is not wrong because China hid the virus) |
| 联合早报 (Lianhe Zaobao) | 3/24/2020 | [陆克文：冠病疫情压倒民族主义](https://www.zaobao.com.sg/zopinions/views/story20200324-1039636) (When COVID spread beats nationalism) |
| 联合早报 (Lianhe Zaobao) | 2/11/2020 | [郑永年：美国（西方）外交中的种族主义情结](https://www.zaobao.com.sg/forum/expert/zheng-yong-nian/story20200211-1027971) (Racist mentality in American [Western] diplomacy) |
| 联合早报 (Lianhe Zaobao) | 3/20/2020 | [欧菁仙：巴黎“封人怨”](https://www.zaobao.com.sg/zlifestyle/columns/story20200320-1038523) (Jingxian Ou Commentary: Hatred under lockdown in Paris) |
| 联合早报 (Lianhe Zaobao) | 3/18/2020 | [特朗普称冠病“中国病毒” 中方抗议双方摩擦激化](https://www.zaobao.com.sg/znews/greater-china/story20200318-1037946) (Trump’s “Chinese virus”, China’s anger and worsened diplomatic relations) |
| 联合早报 (Lianhe Zaobao) | 4/4/2020 | [刀口上的口罩封锁与口罩外交](https://www.zaobao.com.sg/wencui/politic/story20200404-1042805) (Mask boycott and mask diplomacy) |
| 联合早报 (Lianhe Zaobao) | 4/13/2020 | [周雁冰：明天会更好](https://www.zaobao.com.sg/zlifestyle/columns/story20200413-1045056) (Tomorrow will be a better day) |
| 联合早报 (Lianhe Zaobao) | 2/13/2020 | [世卫：避免引发歧视病毒正名COVID-19](https://www.zaobao.com.sg/znews/international/story20200213-1028500) (WHO: Coronavirus called COVID-19 to curb racism) |
| 联合晚报 (Lianhe Wanbao) | --- | --- |
| **Brazil (Jan.23-Apr.22, 2020)** | | |
| **Newspaper** | **Date** | **News Title** |
| Folha De S.Paulo | 2/3/2020 | [Em meio a surto de coronavírus, orientais no Brasil relatam preconceito e desconforto](https://www1.folha.uol.com.br/equilibrioesaude/2020/02/em-meio-a-surto-de-coronavirus-orientais-no-brasil-relatam-preconceito-e-desconforto.shtml) (Anti-China feeling spreads with the spread of coronavirus) |
| Folha De S.Paulo | 1/30/2020 | [Asiaticos-pelo-mundo-ja-sofrem-discriminacao-diante-do-temor-do-coronavirus](https://www1.folha.uol.com.br/equilibrioesaude/2020/01/asiaticos-pelo-mundo-ja-sofrem-discriminacao-diante-do-temor-do-coronavirus.shtml) (Asians around the world already suffer discrimination in the face of the fear of coronavirus) |
| O Estado de S. Paulo | 3/19/2020 | [Na contramao de Eduardo Bolsonaro, Mandetta pediu respeito a chineses ao tratar do coronavirus](https://politica.estadao.com.br/noticias/geral,na-contramao-de-eduardo-bolsonaro-mandetta-pediu-respeito-a-chineses-ao-tratar-do-coronavirus,70003239474) (Against Eduardo Bolsonaro, Mandetta asked to respect Chinese when dealing with the coronavirus) |
| O Estado de S. Paulo | 2/14/2020 | [Rumores e panico podem aasustar mais que a epidemia', diz embaixador da China sobre coronavirus](https://saude.estadao.com.br/noticias/geral,rumores-e-panico-podem-assustar-mais-que-a-epidemia-diz-embaixador-da-china-sobre-coronavirus,70003196973) (Rumor and panic can scare more than the epidemic, says Chinese ambassador on coronavirus) |
| O Estado de S. Paulo | 3/19/2020 | [Trump defende uso do termo 'virus chines' para o coronavirus: Nao e racista](https://internacional.estadao.com.br/noticias/geral,trump-defende-uso-do-termo-virus-chines-para-o-coronavirus-nao-e-racista,70003239355) (Trump defends use of the term ‘Chinese virus’ for coronavirus: ‘Not racist’) |
| O Estado de S. Paulo | 4/7/2020 | [A contraditoria retorica anti-China](https://politica.estadao.com.br/blogs/fausto-macedo/a-contraditoria-retorica-anti-china/) (The contradictory anti-China rhetoric) |
| O Estado de S. Paulo | 3/2/2020 | [Weintraub e a sensatez do CNE (Weintraub and the snsibility of the CNE)](https://opiniao.estadao.com.br/noticias/notas-e-informacoes,weintraub-e-a-sensatez-do-cne,70003289874) |
| O Estado de S. Paulo | 3/18/2020 | [O SUS e o direito a saude em tempos de coronavirus](https://politica.estadao.com.br/blogs/fausto-macedo/o-sus-e-o-direito-a-saude-em-tempos-de-coronavirus/) (SUS and the right to health in the times of coronavirus) |
| O Estado de S. Paulo | 3/31/2020 | [Inquisicao, coronavirus e corrupcao](https://politica.estadao.com.br/blogs/fausto-macedo/inquisicao-coronavirus-e-corrupcao/) (Inquisition, coronavirus, and corruption) |
| O Estado de S. Paulo | 1/31/2020 | [Sentimento anti-china se espalha com a propagacao do coronavirus](https://saude.estadao.com.br/noticias/geral,sentimento-anti-china-se-espalha-com-a-propagacao-do-coronavirus,70003180318) (Anti-china feeling spreads with the spread of coronavirus) |
| O Globo | 2/5/2020 | [Instituto Brasil-China critica discriminação e diz que ajudará a identificar crimes contra asiáticos](https://oglobo.globo.com/sociedade/coronavirus/instituto-brasil-china-critica-discriminacao-diz-que-ajudara-identificar-crimes-contra-asiaticos-1-24230737) (Brazil-China Institute criticizes discrimination and says it will help to identify crimes against Asians) |
| O Globo | 1/30/2020 | [Banidos de hotéis, excluídos de escolas, vítimas de comentários racistas: coronavírus provoca histeria mundial contra chineses](https://g1.globo.com/mundo/noticia/2020/01/30/banidos-de-hoteis-excluidos-de-escolas-vitimas-de-comentarios-racistas-coronavirus-provoca-histeria-mundial-contra-chineses.ghtml) (Banned from hotels, excluded from schools, victims of racist comments: coronavirus causes global hysteria against Chinese) |
| O Globo | 3/3/2020 | [OMS lança guia contra o estigma social em torno do coronavírus](https://oglobo.globo.com/sociedade/coronavirus/oms-lanca-guia-contra-estigma-social-em-torno-do-coronavirus-24283587) (WHO launches guide against social stigma around coronavirus) |
| O Globo | 2/4/2020 | [Estudante diz ter sido chamada de 'chinesa porca' por idosa no metrô do Rio e registra queixa na polícia](https://g1.globo.com/rj/rio-de-janeiro/noticia/2020/02/04/estudante-diz-ter-sido-chamada-de-chinesa-porca-por-idosa-no-metro-do-rio-e-registra-ocorrencia.ghtml) (Student says she was called a ‘Chinese pig’ by an elderly woman on the Rio subway and files a police complaint) |
| O Globo | 3/23/2020 | [Artigo: Preconceito político e teoria da conspiração](https://oglobo.globo.com/opiniao/artigo-preconceito-politico-teoria-da-conspiracao-24322035) (Political prejudice and conspiracy theory) |
| O Globo | 3/23/2020 | [Cuspe, gritos e ataques: em tempos de coronavírus, chineses americanos temem por sua segurança](https://oglobo.globo.com/mundo/cuspe-gritos-ataques-em-tempos-de-coronavirus-chineses-americanos-temem-por-sua-seguranca-24322546) (Spit, screams and attacks: in times of coronavirus, Chinese Americans fear for their safety) |
| O Globo | 3/31/2020 | [Chineses de cidade italiana antecipam quarentena e ficam sem casos de coronavírus](https://oglobo.globo.com/mundo/chineses-de-cidade-italiana-antecipam-quarentena-ficam-sem-casos-de-coronavirus-1-24342426) (Chinese in Italian city anticipate quarantine and run out of coronavirus cases) |
| O Globo | 2/11/2020 | [Racismo e xenofobia on-line se banalizam, e número de denúncias no Brasil diminui](https://oglobo.globo.com/sociedade/racismo-xenofobia-on-line-se-banalizam-numero-de-denuncias-no-brasil-diminui-24242017) (Online racism and xenophobia become commonplace, and the number of complaints in Brazil decreases) |
| O Globo | 2/5/2020 | [Coronavírus: condomínio em SP tentou segregar chineses como 'medida de prevenção'](https://g1.globo.com/sp/sao-paulo/noticia/2020/02/05/coronavirus-condominio-em-sp-tentou-segregar-chineses-como-medida-de-prevencao.ghtml) (Coronavirus: condominium in SP tried to segregate Chinese as a ‘preventive measure’) |
| O Globo | 4/4/2020 | [Artigo: Valorize as relações China-Brasil, deputado Eduardo](https://oglobo.globo.com/opiniao/artigo-valorizeas-relacoes-china-brasildeputado-eduardo-24350358) (Article: Value China-Brazil relations, Deputy Eduardo) |
| O Globo | 4/8/2020 | [Respondendo a Trump, OMS nega centrar-se na China e diz que pior da pandemia ainda está pela frente](https://oglobo.globo.com/mundo/respondendo-trump-oms-nega-centrar-se-na-china-diz-que-pior-da-pandemia-ainda-esta-pela-frente-24358815) (Responding to Trump, WHO denies focusing on China and says the worst of the pandemic is still ahead) |
| O Globo | 2/6/2020 | [Como o coronavírus vai ganhar um novo nome e por que isso é importante](https://g1.globo.com/ciencia-e-saude/noticia/2020/02/06/como-o-coronavirus-vai-ganhar-um-novo-nome-e-por-que-isso-e-importante.ghtml) (How the coronavirus will get a new name and why this is important) |
| **Malaysia (Jan.23-Apr.19, 2020)** | | |
| **Newspaper** | **Date** | **News Title** |
| New Straits Times | 3/28/2020 | [Coronavirus scare prompts racist attacks on 'Chinese-looking' Indians](https://www.straitstimes.com/asia/south-asia/coronavirus-scare-prompts-racist-attacks-on-chinese-looking-indians) |
| New Straits Times | 2/21/2020 | [Covid-19-Wary Singaporeans Shunning Chinese Citizens, Businesses on Island](https://www.nst.com.my/world/world/2020/02/567585/covid-19-wary-singaporeans-shunning-chinese-citizens-businesses-island) |
| New Straits Times | 2/17/2020 | [Covid-19 outbreak causing people to spread fake news, stirring up xenophobia](https://www.nst.com.my/news/nation/2020/02/566613/covid-19-outbreak-causing-people-spread-fake-news-stirring-xenophobia) |
| New Straits Times | 2/15/2020 | [Anti-Asian bigotry spreading faster than Covid-19 in California](https://www.nst.com.my/world/world/2020/02/565691/anti-asian-bigotry-spreading-faster-covid-19-california) |
| New Straits Times | 2/15/2020 | [Following the French in dealing with fear](https://www.nst.com.my/opinion/columnists/2020/02/565673/following-french-dealing-fear) |
| New Straits Times | 2/12/2020 | [Dutch DJ apologises for racist Covid-19 song](https://www.nst.com.my/world/world/2020/02/564773/dutch-dj-apologises-racist-covid-19-song) |
| New Straits Times | 2/21/2020 | [After Covid-19 quarantine, Americans face stigma](https://www.nst.com.my/world/world/2020/02/567523/after-covid-19-quarantine-americans-face-stigma) |
| New Straits Times | 2/8/2020 | [Don't add undue pressure on China](https://www.nst.com.my/opinion/columnists/2020/02/563566/dont-add-undue-pressure-china) |
| New Straits Times | 2/19/2020 | [Covid-19 sinophobia likely due to rise in fallacies](https://www.nst.com.my/news/nation/2020/02/566925/covid-19-sinophobia-likely-due-rise-fallacies) |
| New Straits Times | 2/17/2020 | [Banning travellers from China only creates false sense of security](https://www.nst.com.my/news/nation/2020/02/566447/banning-travellers-china-only-creates-false-sense-security) |
| New Straits Times | 2/14/2020 | [NST leader: Sinophobia](https://www.nst.com.my/opinion/leaders/2020/02/565365/nst-leader-sinophobia) |
| New Straits Times | 1/30/2020 | [Distasteful stereotyping of a health concern](https://www.nst.com.my/opinion/letters/2020/01/560843/distasteful-stereotyping-health-concern) |
| New Straits Times | 3/30/2020 | [Covid-19 knows no national boundaries, has no prejudices](https://www.nst.com.my/opinion/letters/2020/03/579348/covid-19-knows-no-national-boundaries-has-no-prejudices) |
| The Star | 3/23/2020 | [Trump under fire for calling Covid-19 Chinese virus](https://www.thestar.com.my/news/regional/2020/03/23/trump-under-fire-for-calling-covid-19-chinese-virus) |
| The Star | April 5, 2020 | [Stigma in the time of coronavirus](https://www.thestar.com.my/news/focus/2020/04/05/stigma-in-the-time-of-coronavirus) |
| The Star | 3/30/2020 | [Covid-19: Huge surge of hate speech toward Chinese on Twitter](https://www.thestar.com.my/tech/tech-news/2020/03/30/covid-19-huge-surge-of-hate-speech-toward-chinese-on-twitter) |
| The Star | 2/20/2020 | [Italian-Chinese man fights back against Covid-19 coronavirus racism in viral video](https://www.thestar.com.my/news/nation/2020/02/14/italian-chinese-man-fights-back-against-coronavirus-racism-in-viral-video) |
| The Star | 3/17/2020 | [Covid-19: Stop the stigma and stereotyping of coronavirus sufferers](https://www.thestar.com.my/lifestyle/health/2020/03/17/fighting-against-the-stigma-of-covid-19) |
| The Star | 2/6/2020 | [Fear of the unknown](https://www.thestar.com.my/news/nation/2020/02/06/fear-of-the-unknown) |
| The Sun | --- | --- |
| **Canada (Jan.23-Apr.20, 2020)** | | |
| **Newspaper** | **Date** | **News Title** |
| Toronto Star | 4/16/2020 | [What it means to be Asian during COVID-19](https://www.thestar.com/opinion/contributors/2020/04/16/what-it-means-to-be-asian-during-covid-19.html) |
| Toronto Star | 4/2/2020 | [Worries grow that discrimination against Chinese Canadians](https://www.thestar.com/politics/federal/2020/04/02/worries-grow-that-discrimination-against-chinese-canadians-is-getting-worse-as-pandemic-continues.html) |
| Toronto Star | 4/13/2020 | [The virus of anti-Asian prejudice](https://www.thestar.com/opinion/contributors/2020/04/13/the-virus-of-anti-asian-prejudice.html) |
| Toronto Star | 1/28/2020 | [I experienced anti-Chinese racism during SARS. But with](https://www.thestar.com/life/opinion/2020/01/28/i-experienced-anti-chinese-racism-during-sars-but-with-coronavirus-scare-social-media-makes-it-so-much-worse.html) |
| Toronto Star | 3/3/2020 | [Chinese organization launches 'stop the spread' of racism](https://www.thestar.com/news/gta/2020/03/03/chinese-organization-launches-stop-the-spread-of-racism-campaign-amid-coronavirus-outbreak.html) |
| Toronto Star | 1/29/2020 | [Coronavirus fallout triggers worry in Toronto's Chinatown](https://www.thestar.com/news/gta/2020/01/29/coronavirus-fallout-triggers-worry-in-torontos-chinatown.html) |
| Toronto Star | 4/15/2020 | [Why it's time for Canadians to be bolder against anti-Asian racism in the age of COVID-19](https://www.thestar.com/opinion/contributors/2020/04/16/why-its-time-for-canadians-to-be-bolder-against-anti-asian-racism-in-the-age-of-covid-19.html) |
| Toronto Star | 3/24/2020 | [“You and your virus go back where you came from”, Canadian actor Russell Yuen repels racist-attack](https://www.thestar.com/opinion/2020/03/24/you-and-your-virus-go-back-where-you-came-from-canadian-actor-russell-yuen-repels-racist-attack.html) |
| Toronto Star | 2/2/2020 | [Toronto flower shop owner left ‘shaken’ after experiencing coronavirus racism](https://www.thestar.com/news/gta/2020/02/02/toronto-flower-shop-owner-left-shaken-after-experiencing-coronavirus-racism.html) |
| Toronto Star | 3/17/2020 | [Covid-19: Stop the stigma and stereotyping of coronavirus sufferers](https://www.thestar.com.my/lifestyle/health/2020/03/17/fighting-against-the-stigma-of-covid-19) |
| The Globe and Mail | 3/19/2020 | [Coronavirus triggers xenophobia in some African countries](https://www.theglobeandmail.com/world/article-coronavirus-triggers-xenophobia-in-some-african-countries/) |
| The Globe and Mail | 3/4/2020 | [Montreal police investigating recent vandalism at Buddhist](v) |
| The Globe and Mail | 2/3/2020 | [Unfortunately xenophobia has existed in this country since the first settlers](https://www.theglobeandmail.com/canada/article-racism-canada-coronavirus-fears-chinese-canadians-xenophobia/) |
| The Globe and Mail | 1/31/2020 | [From the archives: For Chinese-Canadians like me, coronavirus is just the latest strain of infectious fear we’ve faced](https://www.theglobeandmail.com/opinion/article-the-line-between-us-for-chinese-canadians-like-me-coronavirus-is/) |
| The Globe and Mail | 1/31/2020 | [‘I know some people are going to get racist about this virus with Chinese-Canadians.’ Readers react to racism fears over the coronavirus](https://www.theglobeandmail.com/canada/article-racism-fears-coronavirus-chinese-canadians-i-know-some-people-racist/) |
| The Globe and Mail | 1/27/2020 | [Fear over coronavirus prompts school board in Ontario to warn parents about racism against Chinese community](https://www.theglobeandmail.com/canada/article-panic-over-coronavirus-prompts-school-board-in-ontario-to-warn-parents/) |
| The Globe and Mail | 3/29/2020 | [Cultures clash over wearing masks amid virus](https://www.theglobeandmail.com/canada/british-columbia/article-cultures-clash-over-wearing-masks-amid-virus/) |
| The Globe and Mail | 2/11/2020 | [Chinese restaurants in GTA see large drop in sales in weeks after coronavirus outbreak](https://www.theglobeandmail.com/canada/toronto/article-chinese-restaurants-in-gta-see-large-drop-in-sales-in-weeks-after/) |
| Hamilton Spectator | 1/31/2020 | [Business down at Wuhan Noodle restaurant in Markham amid racism, coronavirus fear](https://www.thespec.com/news/ontario/2020/01/31/business-down-at-wuhan-noodle-restaurant-in-markham-amid-racism-coronavirus-fear.html) |
| Hamilton Spectator | 1/29/2020 | [Editorial: Bigotry is the virus we should worry most about](https://www.thespec.com/opinion/editorials/2020/01/29/editorial-bigotry-is-the-virus-we-should-worry-most-about.html) |
| **Angola (Jan. 23-Apr.17, 2020)** | | |
| **Newspaper** | **Date** | **News Title** |
| Angola Press Agency | --- | --- |
| Jornal de Angola | --- | --- |
| Ver Angola | 4/6/2020 | [Covid-19: “You can’t pretend” that Angola-China relations are not being affected](https://www.verangola.net/va/en/042020/Trade/19359/Covid-19-You-can'-t-pretend-that-Angola-China-relations-are-not-being-affected.htm) |
| **Belgium (Jan.23-Apr.18, 2020)** | | |
| **Newspaper** | **Date** | **News Title** |
| Het Laatste Nieuws | 3/4/2020 | [Aziatische man (23) slachtoffer van ‘coronaracisme’ op straat in Londen](https://www.hln.be/buitenland/aziatische-man-23-slachtoffer-van-coronaracisme-op-straat-in-londen~aa772180/) (Asian man (23) victim of “corona racism” on the street in London) |
| Het Laatste Nieuws | 2/9/2020 | [Schokkend opschrift “sterf Chinees” in Nederlands studentenhuis, vloer besmeurd met uitwerpselen](https://www.hln.be/buitenland/schokkend-opschrift-sterf-chinees-in-nederlands-studentenhuis-vloer-besmeurd-met-uitwerpselen~a2655d93/) ( Shocking inscription “die Chinese” in Dutch student house, floor smeared with excrement) |
| Het Laatste Nieuws | 1/31/2020 | [Chinezen worden gemeden, gepest en gediscrimineerd vanwege coronavirus](https://www.hln.be/buitenland/chinezen-worden-gemeden-gepest-en-gediscrimineerd-vanwege-coronavirus~af94bd15/) (Chinese are shunned, bullied and discriminated against because of the coronavirus) |
| Het Nieuwsblad | 3/3/2020 | [Deze Chinese vrouw is het gezicht van het coronavirus: “Ik ben nog nooit zo vernederd”](https://www.nieuwsblad.be/cnt/dmf20200302_04872778) (This Chinese woman is the face of the coronavirus: “I have never been so humiliated”) |
| Het Nieuwsblad | 1/31/2020 | [Gerepatrieerde Belgen uit Wuhan moeten 14 dagen in quarantaine, Aziatische Vlamingen merken angst](https://www.nieuwsblad.be/cnt/dmf20200131_04829310) (Repatriated Belgians from Wuhan have to be quarantined for 14 days, Asian Flemish people notice fear) |
| Het Nieuwsblad | 1/31/2020 | [Chinezen in Vlaanderen voelen zich geviseerd sinds uitbraak: “‘Daar komt hij met het coronavirus’, hoor je dan”](https://www.nieuwsblad.be/cnt/dmf20200131_04828563) (Chinese in Flanders feel targeted since the outbreak: “Here he comes with the coronavirus”, you hear) |
| Le Soir | 3/4/2020 | [Coronavirus : la communauté chinoise à Bruxelles «en a marre»](https://plus.lesoir.be/284690/article/2020-03-04/coronavirus-la-communaute-chinoise-bruxelles-en-marre) (Coronavirus: the Chinese community in Brussels “is fed up”) |
| Le Soir | 3/17/2020 | [Coronavirus: l’épidémie des riches après celles propagées par les pauvres](https://plus.lesoir.be/288010/article/2020-03-17/coronavirus-lepidemie-des-riches-apres-celles-propagees-par-les-pauvres) (Coronavirus: the epidemic of the rich after those spread by the poor) |
| Le Soir | 2/17/2020 | [Coronavirus: y a-t-il une recrudescence de racisme asiaphobe](https://www.lesoir.be/280934/article/2020-02-17/coronavirus-y-t-il-une-recrudescence-de-racisme-asiaphobe) (Coronavirus: is there an upsurge in Asian racism) |
| **Portugal (Jan.23-Apr.17, 2020)** | | |
| **Newspaper** | **Date** | **News Title** |
| Correio da Manhã | 2/5/2020 | [Prostitutas chinesas obrigadas a cobrar metade do preço por falta de clientes devido ao coronavírus](https://www.cmjornal.pt/mundo/detalhe/prostitutas-chinesas-obrigadas-a-cobrar-metade-do-preco-por-falta-de-clientes-devido-ao-coronavirus) (Chinese prostitutes forced to charge half the price for lack of customers due to coronavirus) |
| Correio da Manhã | 3/10/2020 | [Lojas e restaurantes chineses fecham para férias após quebra nos negócios devido ao coronavírus](https://www.cmjornal.pt/sociedade/detalhe/lojas-e-restaurantes-chineses-fecham-para-ferias-apos-quebra-nos-negocios-devido-a-coronavirus) (Chinese shops and restaurants close for holidays after business breakdown due to coronavirus) |
| Correio da Manhã | 2/3/2020 | [Em meio a surto de coronavírus, orientais no Brasil relatam preconceito e desconforto](https://www.jornalcorreiodamanha.com.br/brasil/1023-em-meio-a-surto-de-coronavirus-orientais-no-brasil-relatam-preconceito-e-desconforto) (Amid an outbreak of coronavirus, Easterners in Brazil report prejudice and discomfort) |
| Expresso | 4/17/2020 | [Covid-19. Liga de Chineses de Portugal queixa-se de juiz por causa da expressão “vírus chinês”](https://expresso.pt/coronavirus/2020-04-17-Covid-19.-Liga-de-Chineses-de-Portugal-queixa-se-de-juiz-por-causa-da-expressao-virus-chines) (Covid-19. Liga de Chineses de Portugal complains about a judge because of the expression “Chinese virus”) |
| Expresso | 3/20/2020 | [Governos devem proteger direitos humanos na resposta à pandemia, alerta HRW](https://expresso.pt/coronavirus/2020-03-19-Governos-devem-proteger-direitos-humanos-na-resposta-a-pandemia-alerta-HRW) (Governments must protect human rights in response to the pandemic, warns HRW) |
| Jornal de Noticias | 3/4/2020 | ["Parem de comer gatos venenosos". Xenofobia contra orientais cresce no Brasil](https://www.jn.pt/mundo/parem-de-comer-gatos-venenosos-xenofobia-contra-orientais-cresce-no-brasil-11887564.html) (“Stop eating poisonous cats”. Xenophobia against Orientals grows in Brazil) |
| Jornal de Noticias | 4/14/2020 | [O vírus racista](https://www.jn.pt/opiniao/manuel-molinos/o-virus-racista-11778189.html) (The racist virus) |
| Jornal de Noticias | 4/17/2020 | [Juiz fala em "vírus chinês" e deixa Liga dos Chineses ofendida](https://www.jn.pt/justica/juiz-fala-em-virus-chines-e-deixa-liga-dos-chineses-ofendida-12085664.html) (Judge speaks of “Chinese virus”  and leaves League of Chinese offended) |
| Jornal de Noticias | 2/3/2020 | [Vila do conde cria grupo de trabalho para reagir a casos de coronavirus](https://www.jn.pt/nacional/vila-do-conde-cria-grupo-de-trabalho-para-reagir-a-casos-de-coronavirus-11781173.html) (Village of the count creates group of work to react to coronavirus cases) |
| Jornal de Noticias | 3/4/2020 | [Nao quero o teu coronavirus no meu pais jovem asiatico agredido em londres](https://www.jn.pt/mundo/nao-quero-o-teu-coronavirus-no-meu-pais-jovem-asiatico-agredido-em-londres-11887077.html) (Don't want your coronavirus in my parents: Young asian battered in London) |
| Jornal de Noticias | 2/5/2020 | [Comunidade chinesa revoltada com programa da rfm que satirizou mortes](https://www.jn.pt/nacional/comunidade-chinesa-revoltada-com-programa-da-rfm-que-satirizou-mortes-11788241.html) (Chinese community revolted with program of rfm that satirized deaths) |
| Jornal de Noticias | 4/15/2020 | [Pgr do brasil pede inquerito a ministro por racismo contra chineses](https://www.jn.pt/mundo/pgr-do-brasil-pede-inquerito-a-ministro-por-racismo-contra-chineses-12072045.html) (Pgr do brasil asks inquiry the minister for racism against Chinese) |
| Jornal de Noticias | 3/8/2020 | [Organizacoes de direitos humanos denunciam problemas na resposta da china ao coronavirus](https://www.jn.pt/mundo/organizacoes-de-direitos-humanos-denunciam-problemas-na-resposta-da-china-ao-coronavirus-11793879.html) (Human rights organizations report problems in China response to coronavirus) |
| **India (Jan.23-Apr.18, 2020)** | | |
| **Newspaper** | **Date** | **News Title** |
| Times of India | 2/19/2020 | [How Coronavirus Is fuelling racial tensions](https://timesofindia.indiatimes.com/india/slurs-boycott-persecution-how-coronavirus-ignorance-and-fear-is-fuelling-social-tensions/articleshow/74174571.cms) |
| The Hindu | 4/13/2020 | [COVID-19 and the crumbling world order](https://www.thehindu.com/opinion/op-ed/covid-19-and-the-crumbling-world-order/article31324259.ece) |
| The Hindu | 3/31/2020 | [People from Northeast face racist slurs amidst coronavirus lockdown](https://www.thehindu.com/news/cities/mumbai/people-from-northeast-face-racist-slurs-coronavirus-lockdown/article31212978.ece) |
| The Hindu | 2/6/2020 | [A time for solidarity, not stigma](https://www.thehindu.com/opinion/op-ed/a-time-for-solidarity-not-stigma/article30744945.ece) |
| Hindustan Times | 3/24/2020 | [Meiyang Chang called coronavirus by two men on bike, asks 'how do you punish people for stupidity, ignorance?'](https://www.hindustantimes.com/tv/meiyang-chang-called-coronavirus-by-two-men-on-bike-asks-how-do-you-punish-people-for-stupidity-ignorance/story-qiDgehmNkMCaZzHbjT6I6I.html) |
| Hindustan Times | 4/11/2020 | [US, China trade blame at UNSC's first Covid-19 meet](https://www.hindustantimes.com/world-news/us-china-trade-blame-at-unsc-s-first-covid-19-meet/story-msuDWe6FHzildlheUjlfjJ.html) |
| Hindustan Times | 2/17/2020 | [N-E students allege discrimination at TISS amid coronavirus scare](https://www.hindustantimes.com/india-news/n-e-students-allege-discrimination-at-tiss-amid-coronavirus-scare/story-5gI6GZgPw8PT2LmelDTV2K.html) |
| Hindustan Times | 2/18/2020 | [Coronavirus scare: North-east students at TISS allege racial discrimination](https://www.hindustantimes.com/mumbai-news/coronavirus-scare-north-east-students-at-tiss-allege-racial-discrimination/story-PHw5NG9IfsP0P8g6DVmgHJ.html) |
| Hindustan Times | 3/7/2020 | ‘[Do not discriminate against foreigners': Tourism ministry tells states](https://www.hindustantimes.com/india-news/do-not-discriminate-against-foreigners-says-director-general-of-tourism/story-oHj0JJ15fWe2OS1a8VrWAL.html) |
| Hindustan Times | 2/6/2020 | [Coronavirus scare fuels racism claims among Uber drivers](https://www.hindustantimes.com/world-news/coronavirus-scare-fuels-racism-claims-among-uber-drivers-riders/story-NaSZiIM9RNwFtmtzpY5BCO.html) |
| **Greece (Jan.23-Apr.23, 2020)** | | |
| **Newspaper** | **Date** | **News Title** |
| Ta Nea | --- | --- |
| To Vima | --- | --- |
| Kathimerini | --- | --- |
| **Sweden (Jan.23-Apr.23, 2020)** | | |
| **Newspaper** | **Date** | **News Title** |
| Dagens Nyheter | 2/7/2020 | [Asiater utanför Kina vittnar om rasism efter virusutbrottet](https://www.dn.se/nyheter/varlden/asiater-utanfor-kina-vittnar-om-rasism-efter-virusutbrottet/) (Asians outside China testify about racism after the virus outbreak) |
| Dagens Nyheter | 2/6/2020 | [”Rasism uppstår i nya coronavirusets spår”](https://www.dn.se/asikt/rasism-uppstar-i-nya-coronavirusets-spar/) (Racism arises in the wake of the new coronavirus) |
| Dagens Nyheter | 2/8/2020 | [Ryktena om coronaviruset används i politiska syften](https://www.dn.se/nyheter/varlden/ryktena-om-coronaviruset-anvands-i-politiska-syften/) (Rumors of the coronavirus are being used for political purposes) |
| Göteborgs-Posten | 2/6/2020 | [Ökad rasism mot asiater efter nya coronaviruset](https://www.gp.se/nyheter/g%C3%B6teborg/%C3%B6kad-rasism-mot-asiater-efter-nya-coronaviruset-1.23578915) (Increased racism against Asians after the new coronavirus) |
| Göteborgs-Posten | 2/7/2020 | [Mamman: De kallade min son för smittspridare](https://www.gp.se/nyheter/sverige/mamman-de-kallade-min-son-f%C3%B6r-smittspridare-1.23679204) (Mother: They called my son a spreader) |
| Göteborgs-Posten | 2/7/2020 | [Ambassadör: Kränkningar mot kineser är rasism](https://www.gp.se/nyheter/sverige/ambassad%C3%B6r-kr%C3%A4nkningar-mot-kineser-%C3%A4r-rasism-1.23691885) (Ambassador: Violations against the Chinese are racism) |
| Expressen | 2/8/2020 | [Kinas ord: Svenskar corona-kränker oss](https://www.expressen.se/nyheter/kinas-ord-svenskar-corona-kranker-oss/) (Swedish Chinese restaurants lose half of their customers) |
| Expressen | 2/23/2020 | [Svenska Kinarestauranger tappar hälften av kunderna](https://www.expressen.se/nyheter/svenska-kinarestauranger-tappar-halften-av-kunderna/) (Swedish Chinese restaurants lose half of their customers) |
| **Saudi Arabia (Jan.23-Apr.16, 2020)** | | |
| **Newspaper** | **Date** | **News Title** |
| Arab News | 4/5/2020 | [The blame game begins: Racism on the rise amid coronavirus pandemic](https://www.arabnews.com/node/1653231/saudi-arabia) |
| Asharq Al-Awsat | --- | --- |
| Al Riyadh | 3/25/2020 | كورو[نا.. «الديموقراطي» المنفتح على الآخر!](https://www.alriyadh.com/1812344) (Corona .. the “democrat” who is open to the other!) |
| **The Netherlands (Jan.23-Apr.16, 2020)** | | |
| **Newspaper** | **Date** | **News Title** |
| De Telegraaf | 2/12/2020 | [Chinees virus: grapje, plagerij of racisme?](https://www.telegraaf.nl/nieuws/1488586819/chinees-virus-grapje-plagerij-of-racisme) (Chinese virus: Joking, teasing or racism?) |
| De Telegraaf | 3/4/2020 | [Student in elkaar geslagen om 'corona'](https://www.telegraaf.nl/nieuws/2072278307/student-in-elkaar-geslagen-om-corona) (Student beaten up for 'corona') |
| Algemeen Dagblad | 1/31/2020 | [Chinezen worden gemeden, gepest en gediscrimineerd vanwege coronavirus](https://www.ad.nl/buitenland/chinezen-worden-gemeden-gepest-en-gediscrimineerd-vanwege-coronavirus~af94bd15/?referrer=https%3A%2F%2Fwww.google.com.hk%2F) (Chinese are shunned, bullied and discriminated against because of coronavirus) |
| Algemeen Dagblad | 2/20/2020 | [Doodsverwensing voor Chinese studenten, vloer besmeurd met poep en urine](https://www.ad.nl/binnenland/doodsverwensing-voor-chinese-studenten-vloer-besmeurd-met-poep-en-urine~a2655d93/) (Death wish for Chinese students, floor smeared with poo and urine) |
| Algemeen Dagblad | 1/31/2020 | [Gediscrimineerd vanwege corona: ‘Moet ik wéér gaan uitleggen dat ik geen honden en katten eet’](https://www.ad.nl/binnenland/gediscrimineerd-vanwege-corona-moet-ik-weer-gaan-uitleggen-dat-ik-geen-honden-en-katten-eet~a3c73d27/) (Discriminated because of corona: 'Do I have to explain again that I don't eat dogs and cats') |
| Algemeen Dagblad | 2/10/2020 | [Aangifte tegen dj om discriminerend coronalied 'Voorkomen is beter dan Chinezen’](https://www.ad.nl/binnenland/aangifte-tegen-dj-om-discriminerend-coronalied-voorkomen-is-beter-dan-chinezen~a421146c/) (Declaration against DJ for discriminating corona song 'Prevention is better than the Chinese') |
| Algemeen Dagblad | 3/16/2020 | [Protest van Jiye (29) tegen coronaracisme gaat niet door wegens... coronarisico](https://www.ad.nl/den-haag/protest-van-jiye-29-tegen-coronaracisme-gaat-niet-door-wegens-coronarisico-br~ae390425/) (Jiye (29) protest against corona racism is canceled due to corona risk) |
| Algemeen Dagblad | 2/14/2020 | [Openbaar Ministerie start onderzoek naar discriminerend coronalied](https://www.ad.nl/binnenland/openbaar-ministerie-start-onderzoek-naar-discriminerend-coronalied~a315b727/) (Public Prosecution Service starts investigation into discriminatory corona song) |
| Algemeen Dagblad | 2/10/2020 | [Gordon door het stof in DWDD om foute grap: ‘Ik wil mijn diepe, diepe excuses aanbieden’](https://www.ad.nl/show/gordon-door-het-stof-in-dwdd-om-foute-grap-ik-wil-mijn-diepe-diepe-excuses-aanbieden~a53d54e3/) (Gordon through the dust in DWDD for wrong joke: 'I want to offer my deep, deep apologies') |
| Algemeen Dagblad | 2/17/2020 | [Smakeloos is iets anders dan kwetsend. Wat dat betreft is het net Chinees eten](https://www.ad.nl/den-haag/smakeloos-is-iets-anders-dan-kwetsend-wat-dat-betreft-is-het-net-chinees-eten~a60da706/) (Tasteless is different from hurtful. It's like Chinese food in that regard) |
| De Volkskrant | 3/11/2020 | [uitgescholden-en-bedreigd-want-alle-chinezen-hebben-corona](https://www.volkskrant.nl/nieuws-achtergrond/uitgescholden-en-bedreigd-want-alle-chinezen-hebben-corona~b563944c/) (Called names and "all Chinese have corona") |
| De Volkskrant | 2/11/2020 | [discriminatie-van-chinezen-in-nederland-heeft-een-lange-geschiedenis](https://www.volkskrant.nl/columns-opinie/discriminatie-van-chinezen-in-nederland-heeft-een-lange-geschiedenis~bdfc2868/) (discrimination-of-chinese-in-the-netherlands-has-a-long-history) |
| De Volkskrant | 2/10/2020 | [we-moeten-nederland-ons-tweede-vaderland-beschermen-zonder-paniek-te-zaaien](https://www.volkskrant.nl/nieuws-achtergrond/we-moeten-nederland-ons-tweede-vaderland-beschermen-zonder-paniek-te-zaaien~be74aec1/) (we-must-protect-the netherlands-our-second-homeland-without-sowing-panic) |
| De Volkskrant | 2/10/2020 | [aangifte-tegen-dj-en-radio-10-om-corona-carnavalslied-voorkomen-is-beter-dan-chinezen](https://www.volkskrant.nl/nieuws-achtergrond/aangifte-tegen-dj-en-radio-10-om-corona-carnavalslied-voorkomen-is-beter-dan-chinezen~bbd3668b/) (declaration-against-dj-and-radio-10-om-corona-carnivalsong-prevention-is-better-than-chinese) |
| De Volkskrant | 3/15/2020 | [het-coronavirus-wakkert-nationalisme-in-europese-landen-aan](https://www.volkskrant.nl/nieuws-achtergrond/het-coronavirus-wakkert-nationalisme-in-europese-landen-aan~bf193e09/) (the-coronavirus-fuels-nationalism-in-european-countries) |
| **Switzerland (Jan.23-Apr.16, 2020)** | | |
| **Newspaper** | **Date** | **News Title** |
| 20 Minuten | 1/31/2020 | [Asien-Schweizer leiden unter Corona-Rassismus](https://www.20min.ch/story/asien-schweizer-leiden-unter-corona-rassismus-852383926676) (Asian-Swiss suffer from corona racism) |
| 20 Minuten | 3/1/2020 | [Roan (23) wurde Opfer von Corona-Rassismus](https://www.20min.ch/story/roan-23-wurde-opfer-von-corona-rassismus-660153492180) (Roan (23) became a victim of Corona racism) |
| 20 minutes | 1/30/2020 | [Coronavirus et racisme antiasiatique : « J’étais tellement choquée de la violence des mots et de cette humiliation »](https://www.20minutes.fr/sante/2706243-20200129-coronavirus-racisme-anti-asiatique-tellement-choquee-violence-mots-humiliation) (Coronavirus and anti-Asian racism: "I was so shocked at the violence of the words and this humiliation") |
| 20 minutes | 2/6/2020 | [Coronavirus : Le racisme antiasiatique se répand dans le monde entier](https://www.20minutes.fr/monde/2710991-20200205-coronavirus-racisme-antiasiatique-repand-monde-entier) (Coronavirus: Anti-Asian racism is spreading around the world) |
| 20 minutes | 1/27/2020 | [Coronavirus : « La référence au "péril jaune" joue sur les fantasmes, les peurs » selon le chercheur Vincent Geisser](https://www.20minutes.fr/arts-stars/medias/2704775-20200127-coronavirus-reference-peril-jaune-joue-fantasmes-peurs-selon-chercheur-vincent-geisser) (Coronavirus: "The reference to the" yellow peril "plays on fantasies, fears" according to researcher Vincent Geisser) |
| 20 minutes | 1/29/2020 | [Coronavirus : Des Français d’origine asiatique dénoncent le racisme ambiant avec le hashtag #JeNeSuisPasUnVirus](https://www.20minutes.fr/societe/2705295-20200128-coronavirus-francais-origine-asiatique-denoncent-racisme-ambiant-hashtag-jenesuispasunvirus) (Coronavirus: French people of Asian origin denounce the prevailing racism with the hashtag #JeNeSuisPasUnVirus) |
| 20 minutes | 3/27/2020 | [Coronavirus : Des associations dénoncent des cas de contrôles policiers « abusifs » et « violents »](https://www.20minutes.fr/societe/2749411-20200327-coronavirus-associations-denoncent-cas-controles-policiers-abusifs-violents) (Coronavirus: Associations denounce cases of "abusive" and "violent" police checks) |
| 20 minutes | 1/27/2020 | [Le « Courrier Picard » s'excuse après sa une jugée raciste](https://www.20minutes.fr/arts-stars/culture/2704551-20200127-courrier-picard-excuse-apres-jugee-raciste) (The "Courrier Picard" apologizes after its a racist judgment) |
| 20 minutes | 3/1/2020 | [Coronavirus : Une lycéenne bretonne d’origine asiatique agressée](https://www.20minutes.fr/sante/2730099-20200301-coronavirus-lyceenne-bretonne-origine-asiatique-agressee) (Coronavirus: A Breton high school student of Asian origin attacked) |
| Tages-Anzeiger | 2/29/2020 | [«Das Virus ergreift uns, bevor es uns befällt»](https://www.tagesanzeiger.ch/wissen/medizin-und-psychologie/panik-ist-angst-ohne-denken/story/22127300) (The virus seizes us before it hits us) |
| Tages-Anzeiger | 2/2/2020 | [Mit dem Mundschutz aufs Jungfraujoch](https://www.tagesanzeiger.ch/schweiz/standard/mit-dem-mundschutz-aufs-jungfraujoch/story/21191818) (With the face mask on the Jungfraujoch) |
| **Mexico (Jan.23-Apr.16, 2020)** | | |
| **Newspaper** | **Date** | **News Title** |
| El Universal | 3/3/2020 | [Cuando China discriminó a mexicanos por influenza](https://www.eluniversal.com.mx/ciencia-y-salud/salud/el-dia-en-que-china-discrimino-mexicanos-por-influenza-h1n1) (When China discriminated against Mexicans for influenza) |
| El Universal | 2/6/2020 | [El hashtag contra la discriminación que se ha vuelto viral](https://www.eluniversal.com.mx/mundo/coronavirus-nosoyunvirus-el-hashtag-contra-la-discriminacion-que-se-ha-vuelto-viral) (The anti-discrimination hashtag that has gone viral) |
| El Universal | 3/25/2020 | [Carmen Salinas se disculpa](https://www.eluniversal.com.mx/espectaculos/farandula/carmen-salinas-se-disculpa-por-comentario-racista-sobre-chinos) (Carmen Salinas apologizes) |
| El Universal | 3/25/2020 | [Laura Bozzo llama a chinos "genocidas"](https://www.eluniversal.com.mx/espectaculos/farandula/laura-bozzo-apoya-carmen-salinas-y-llama-chinos-genocidas) (Laura Bozzo calls Chinese "genocidal") |
| El Universal | 3/17/2020 | ['Travieso' Arce exhibe su xenofobia con China por el COVID-19](https://www.eluniversal.com.mx/universal-deportes/box/coronavirus-travieso-arce-exhibe-su-xenofobia-con-china-por-el-covid-19) ('Naughty' Arce exhibits his xenophobia with China due to COVID-19) |
| El Universal | 3/28/2020 | [Covid-19: estereotipos y etiquetas](https://www.eluniversal.com.mx/opinion/mauricio-meschoulam/covid-19-estereotipos-y-etiquetas) (Covid-19: stereotypes and labels) |
| Reforma | 4/4/2020 | [Retrata 'Corona' xenofobia por Covid-19](https://www.reforma.com/aplicacioneslibre/preacceso/articulo/default.aspx?__rval=1&urlredirect=https:/www.reforma.com/retrata-corona-xenofobia-por-covid-19/ar1912055?referer=--7d616165662f3a3a6262623b727a7a7279703b767a783b7d7e3a--) ('Corona' portrays xenophobia by covid-19) |
| Reforma | 4/16/2020 | [Impulsa China recuperación y desarrollo](https://www.reforma.com/libre/acceso/accesofb.htm?urlredirect=/impulsa-china-recuperacion-y-desarrollo/ar1921403) (Promote China recovery and development) |
| La Jornada | 2/3/2020 | [Ciudadanos chinos sufren racismo y discriminación](https://www.jornada.com.mx/2020/02/03/mundo/020n2mun) (Chinese citizens suffer racism and discrimination) |
| La Jornada | 3/25/2020 | [Conapred llama a erradicar discriminación contra ciudadanos chinos](https://www.jornada.com.mx/ultimas/politica/2020/03/25/conapred-llama-a-erradicar-discriminacion-contra-ciudadanos-chinos-7203.html) (Conapred calls to eradicate discrimination against Chinese citizens) |
| La Jornada | 2/13/2020 | [En el barrio chino ni temen ni se espantan por el Covid-19](https://www.jornada.com.mx/2020/02/13/capital/029n2cap) (In Chinatown they are neither afraid nor frightened by Covid-19) |
| La Jornada | 2/3/2020 | [El racismo, la peor epidemia](https://www.jornada.com.mx/2020/02/03/edito) (Racism, the worst epidemic) |
| La Jornada | 2/19/2020 | [Xenofobia, otro peligro de la epidemia](https://www.jornada.com.mx/2020/02/19/edito) (Xenophobia, another danger of the epidemic) |
| La Jornada | 4/11/2020 | [Trump aprovecha el Covid-19 para impulsar xenofobia en EU](https://www.jornada.com.mx/ultimas/mundo/2020/04/11/trump-aprovecha-el-covid-19-para-impulsar-xenofobia-en-eu-2722.html) (Trump takes advantage of Covid-19 to promote xenophobia in the US) |
| La Jornada | 4/9/2020 | [Racismo, otro rostro de la pandemia](https://www.jornada.com.mx/2020/04/09/opinion/021a1pol) (Racism, another face of the pandemic) |
| La Jornada | 3/10/2020 | [Italia: Covid-19 y xenofobia](https://www.jornada.com.mx/2020/03/10/opinion/028a1mun) (Italy: Covid-19 and xenophobia) |
| **South Africa (Jan.23-Apr.17, 2020)** | | |
| **Newspaper** | **Date** | **News Title** |
| Daily Sun | 3/30/2020 | [Masechaba Ndlovu bashed for sharing 'offensive' coronavirus video](https://www.dailysun.co.za/Celebs/masechaba-ndlovu-bashed-for-sharing-offensive-coronavirus-video-20200330) |
| Isolezwe | **---** | **---** |
| The Star | **---** | **---** |
| **Vietnam (Jan.23-Apr.18, 2020)** | | |
| **Newspaper** | **Date** | **News Title** |
| Tuoi Tre | 4/1/2020 | [Cộng đồng người Mỹ gốc Á trước rủi ro sức khỏe trong mùa dịch COVID-19](https://tuoitre.vn/cong-dong-nguoi-my-goc-a-truoc-rui-ro-suc-khoe-trong-mua-dich-covid-19-20200331173418189.htm) (The Asian American community faces health risks during the COVID-19 pandemic) |
| Tuoi Tre | 3/17/2020 | [Ông Trump gọi 'virus Trung Quốc', báo Trung Quốc phản bác ngay](https://tuoitre.vn/ong-trump-goi-virus-trung-quoc-bao-trung-quoc-phan-bac-ngay-20200317125034159.htm) (Mr. Trump called the 'Chinese virus', the Chinese newspaper immediately denied it) |
| Tuoi Tre | 1/28/2020 | ['Người phụ nữ trẻ ăn dơi' không phải người Vũ Hán](https://tuoitre.vn/nguoi-phu-nu-tre-an-doi-khong-phai-nguoi-vu-han-20200128093118414.htm) ('Young woman eating bats' is not Wuhan) |
| Tuoi Tre | 2/26/2020 | [Bộ Ngoại giao Trung Quốc: 'Báo Wall Street Journal đã nhận sai'](https://tuoitre.vn/bo-ngoai-giao-trung-quoc-bao-wall-street-ournal-da-nhan-sai-20200226151841392.htm) (China's Foreign Ministry: 'Wall Street Journal got it wrong') |
| Tuoi Tre | 2/2/2020 | [Thủ tướng Canada cảnh báo tình trạng kỳ thị người Trung Quốc do virus corona](https://tuoitre.vn/thu-tuong-canada-canh-bao-tinh-trang-ky-thi-nguoi-trung-quoc-do-virus-corona-20200202085731242.htm) (Canadian Prime Minister warns of discrimination against Chinese people due to corona virus) |
| Tuoi Tre | 3/13/2020 | [Khẩu trang đắt hàng ở châu Á nhưng thất thế ở Mỹ](https://tuoitre.vn/khau-trang-dat-hang-o-chau-a-nhung-that-the-o-my-20200313012849859.htm) (Masks are expensive in Asia but lost in the US) |
| Tuoi Tre | 4/6/2020 | [Sinh viên quốc tế lâm cảnh chông chênh](https://tuoitre.vn/sinh-vien-quoc-te-lam-canh-chong-chenh-20200406112219462.htm) (International students are in a precarious situation) |
| Viet Nam News | --- | **---** |
| Tien Phong | 3/3/2020 | [Trong "tâm bão" virus corona, nạn phân biệt chủng tộc bùng nổ nhắm vào người châu Á](https://hoahoctro.tienphong.vn/trong-tam-bao-virus-corona-nan-phan-biet-chung-toc-bung-no-nham-vao-nguoi-chau-a-post1218678.tpo) (In the "heart of the storm" of the corona virus, racism broke out targeting Asians) |
| Tien Phong | 3/6/2020 | [Sinh viên châu Á nghi bị tấn công ở London vì phân biệt chủng tộc dịch Covid-19](https://tienphong.vn/sinh-vien-chau-a-nghi-bi-tan-cong-o-london-vi-phan-biet-chung-toc-dich-covid-19-post1173592.tpo) (Asian students suspected of being attacked in London because of racism during the Covid-19 epidemic) |
| **Kenya (Jan.23-Apr.18, 2020)** | | |
| **Newspaper** | **Date** | **News Title** |
| Daily Nation | --- | --- |
| Standard | 3/4/2020 | [Coronavirus is not China virus, just as Ebola is not Africa virus — Chinese Ambassador Wu Peng](https://www.standardmedia.co.ke/ureport/article/2001362823/coronavirus-is-not-china-virus-just-as-ebola-is-not-africa-virus-chinese-ambassador-wu-peng) |
| Standard | 3/5/2020 | [Cases of racist attacks in wake of coronavirus](https://www.standardmedia.co.ke/health/article/2001363029/cases-of-racist-attacks-in-wake-of-coronavirus) |
| Standard | 3/16/2020 | [It is wrong to stigmatise victims of coronavirus](https://www.standardmedia.co.ke/letters/article/2001364471/it-is-wrong-to-stigmatise-victims-of-coronavirus) |
| Standard | 3/5/2020 | [Harassing Chinese nationals over coronavirus wrong](https://www.standardmedia.co.ke/editorial/article/2001362949/harassing-chinese-nationals-over-coronavirus-wrong) |
| Taifa Leo | --- | --- |
| **Guatemala (Jan.23-Apr.18, 2020)** | | |
| **Newspaper** | **Date** | **News Title** |
| Prensa Libre | 2/16/2020 | [Taxistas en Nueva York se niegan a llevar pasajeros chinos por temor al coronavirus](https://www.prensalibre.com/internacional/taxistas-en-nueva-york-se-niegan-a-llevar-pasajeros-chinos-por-temor-al-coronavirus/) (New York taxi drivers refuse to take Chinese passengers for fear of coronavirus) |
| El Periodio | 2/7/2020 | [Las redes se movilizan contra la discriminación a la población china por el coronavirus](https://www.elperiodico.com/es/extra/20200205/redes-discriminacion-poblacion-china-coronavirus-7836985) (Networks mobilize against discrimination against the Chinese population by the coronavirus) |
| El Periodio | 2/17/2020 | [Familias de menores chinos denuncian discriminaciones por el coronavirus](https://www.elperiodico.com/es/sociedad/20200217/familias-menores-chinos-denuncian-discriminacion-coronavirus-7852711) (Families of Chinese minors denounce discrimination due to coronavirus) |
| El Periodio | 3/17/2020 | [El coronavirus crea nuevas tensiones entre EEUU y China](https://elperiodico.com.gt/noticias/internacionales/2020/03/17/el-coronavirus-crea-nuevas-tensiones-entre-eeuu-y-china/) (The coronavirus creates new tensions between the US and China) |
| El Periodio | 3/25/2020 | [Trump elimina de sus discursos la palabra "virus chino" para referirse al COVID-19](https://www.elperiodico.com/es/internacional/20200325/trump-elimina-palabra-virus-chino-covid-19-7903832) (Trump removes the word "Chinese virus" from his speeches to refer to COVID-19) |
| La Hora | --- | --- |
| **Uruguay (Jan.23-Apr.18, 2020)** | | |
| **Newspaper** | **Date** | **News Title** |
| El Pais | 3/24/2020 | [Declaración de la Embajada de China en Uruguay](https://www.elpais.com.uy/mundo/declaracion-embajada-china-uruguay.html?__cf_chl_jschl_tk__=5dbfe46c1aa0e3dd22d2fe215f6f34cf8a0d9572-1623734412-0-ARMl5R7rlWDwoKIWR6j5CJEEt4_D_AWfp7fesjqTuIrhQ7GurslMc20QR9GcyIXumJ4fEtlX0jiUcibHpmPKHQaO8UTbyzcMJXH-tw_VvlyrVe) (Statement from the Chinese Embassy in Uruguay) |
| El Pais | 4/3/2020 | [Coronavirus: qué esconde la "sopa de murciélago" a la que culpan por la pandemia](https://www.elpais.com.uy/informacion/sociedad/coronavirus-esconde-sopa-murcielago-culpan-pandemia.html) (Coronavirus: what hides the "bat soup" blamed for the pandemic) |
| El Observador | 2/21/2020 | [Coronavirus: qué es la sinofobia y cómo la epidemia destapa el miedo y odio a China](https://www.elobservador.com.uy/nota/coronavirus-que-es-la-sinofobia-y-como-la-epidemia-destapa-el-miedo-y-odio-a-china-202022143930) (Coronavirus: what is Sinophobia and how the epidemic uncovers the fear and hatred of China) |
| El Observador | 1/31/2020 | [Coronavirus: aerolíneas de todo el mundo suspenden sus vuelos a China y EE.UU. emite una alerta de viaje por la enfermedad de Wuhan](https://www.elobservador.com.uy/nota/coronavirus-aerolineas-de-todo-el-mundo-suspenden-sus-vuelos-a-china-y-ee-uu-emite-una-alerta-de-viaje-por-la-enfermedad-de-wuhan-202013116338) (Coronavirus: Airlines around the world suspend their flights to China and the US issues a travel alert for Wuhan disease) |
| La Republica | --- | --- |
| **Chile (Jan.23-Apr.18, 2020)** | | |
| **Newspaper** | **Date** | **News Title** |
| Las Ultimas Noticias | --- | --- |
| La Tercera | 3/22/2020 | [Xenofobia en tiempos de crisis:“Me escupieron porque pensaban que tenía coronavirus”](https://www.latercera.com/la-tercera-domingo/noticia/xenofobia-en-tiempos-de-crisis-me-escupieron-porque-pensaban-que-tenia-coronavirus/2XBVYQUBT5AIFLSAIFEGKXNUBI/) (Xenophobia in times of crisis: "They spat on me because they thought I had coronavirus") |
| La Tercera | 1/31/2020 | [Insultos y prohibicin de ingreso: Coronavirus gatilla ola de racismo contra chinos en el mundo](https://www.latercera.com/la-tercera-pm/noticia/insultos-y-prohibicion-de-ingreso-coronavirus-gatilla-ola-de-racismo-contra-chinos-en-el-mundo/994244/) (Insults and entry ban: Coronavirus triggers wave of racism against Chinese in the world) |
| La Tercera | 3/27/2020 | [Discursos de odio contra China aumentaron en un 900% debido al coronavirus](https://www.latercera.com/mouse/discursos-odio-china-coronavirus/) (Hate speeches against China increased by 900% due to coronavirus) |
| La Tercera | 3/25/2020 | [La xenofobia del coronavirus. Además, ¿en qué está la búsqueda de una vacuna?](https://www.latercera.com/podcast/noticia/la-xenofobia-del-coronavirus-ademas-en-que-esta-la-busqueda-de-una-vacuna/NXHTQJQT3VAZBFY53LD3F5S5NI/) (The xenophobia of the coronavirous.) |
| La Tercera | 3/6/2020 | [coronavirus-bachelet-llama-a-combatir-la-estigmatizacion-y-advierte-consecuencias-del-covid-19](https://www.latercera.com/nacional/noticia/coronavirus-bachelet-llama-a-combatir-la-estigmatizacion-y-advierte-consecuencias-del-covid-19/4LYEJ4QPZZEGRA7NJAJPWTKUPU/) (Bachelet-coronavirus-calls-to-fight-stigmatization-and-warns-consequences-of-covid-19) |
| El Mostrador | 4/25/2020 | [Prejuicios y xenofobia en la agenda pública durante el COVID-19](https://www.elmostrador.cl/noticias/opinion/columnas/2020/04/15/prejuicios-y-xenofobia-en-la-agenda-publica-durante-el-covid-19/) (Prejudice and xenophobia on the public agenda during COVID-19) |
| El Mostrador | 2/26/2020 | [Sinofobia](https://www.elmostrador.cl/noticias/opinion/columnas/2020/02/26/sinofobia/) (Sinophobia) |
| El Mostrador | 4/2/2020 | [Pandemia, orientalismo y discriminacion](https://www.elmostrador.cl/noticias/opinion/2020/04/02/pandemia-orientalismo-y-discriminacion/) (Pandemic, orientalism and discrimination) |
| **Kuwait (Jan.23-Apr.18, 2020)** | | |
| **Newspaper** | **Date** | **News Title** |
| Arab Times | --- | --- |
| Kuwait Times | --- | --- |
| Al Rai Al Aam | --- | --- |
| **Lebanon (Jan.23-Apr.16, 2020)** | | |
| **Newspaper** | **Date** | **News Title** |
| Al Balad | --- | --- |
| The Daily Star | --- | --- |
| Al Akhbar | 2/4/2020 | ع[نصريّة لا تعرف حدوداً](https://al-akhbar.com/World/283626) (Racism knows no bounds) |

**S9 Table. Sourcing Ethnic Chinese Media.** List of media outlets sourced for ethnic Chinese newspaper reports of discrimination against Chinese during COVID-19 by country (Jan.23-Apr.23, 2020)

| Country/ Region | Number of Participants | Number of Reports | End Date of Search | Sourced Newspapers | Ranking Reference |
| --- | --- | --- | --- | --- | --- |
| USA | 60 | 156 | 21/04/2020 | 《侨报》; 《世界新闻网》;《星岛日报》 | <https://www.opportunityagenda.org/explore/resources-publications/immigration-coverage-chinese-language-newspapers> |
| Spain | 42 | 62 | 20/04/2020 | 《欧华报》; 《西班牙侨声报》;《华新报》（欧浪网） | https://worldchinesemedia.com/ |
| UK | 42 | 32 | 19/04/2020 | FT中文网; 《英中时报》;《英国侨报》 | https://worldchinesemedia.com/ |
| Italy | 29 | 17 | 23/04/2020 | 《欧华联合时报》;《欧洲时报》;《欧洲华人报》 | http://www.chinanews.com/focus_site/hwlt-4/mtjj-yidali.htm |
| Germany | 19 | 22 | 16/04/2020 | 《德国之声》;《欧洲新桥报》（《新欧洲侨报》;《欧洲时报德国》 | https://worldchinesemedia.com/ |
| Australia | 17 | 39 | 21/04/2020 | 《SBS中文网》;《ABC》中文版; 《澳大利亚人》报 | http://sydpost.com/20713 |
| Japan | 15 | 13 | 18/04/2020 | 《日本新华侨报》;《日本新闻网》;《日本东方新报》 | https://www.hhlink.com/%E5%88%86%E7%B1%BB%E6%8E%92%E8%A1%8C%E6%A6%9C/%E6%97%A5%E6%9C%AC/%E4%B8%AD%E6%96%87%E5%AA%92%E4%BD%93/1 |
| South Korea | 14 | 6 | 16/04/2020 | 《朝鲜日报》;《中央日报》; 人民网韩文版 | https://www.google.com.hk/search?q=%E9%9F%A9%E5%9B%BD%E7%9A%84%E4%B8%AD%E6%96%87%E6%8A%A5%E7%BA%B8&newwindow=1&sxsrf=AOaemvIsIBppRfXYylyQ7_3gZ28Dy5Bf2w%3A1632113062490&ei=phFIYaOvHdPQ-QalmpSADw&oq=%E9%9F%A9%E5%9B%BD%E7%9A%84%E4%B8%AD%E6%96%87%E6%8A%A5%E7%BA%B8&gs_lcp=Cgdnd3Mtd2l6EAMyBAgjECc6BQgAEJECOg4ILhCABBCxAxDHARCjAjoICAAQgAQQsQM6BAgAEEM6BAguEEM6BwgAELEDEEM6CAguEIAEELEDOgsILhCABBCxAxCDAToHCAAQsQMQCjoECAAQCjoECC4QCjoECAAQDToECC4QDToECAAQHjoFCAAQgAQ6BwgAEIAEEAxKBAhBGABQ5NkIWIXwCGCU8QhoAHAAeACAAeICiAGjHJIBCDMuMTguMy4xmAEAoAEBwAEB&sclient=gws-wiz&ved=0ahUKEwijmsr63ozzAhVTaN4KHSUNBfAQ4dUDCA4&uact=5 |
| Russia | 13 | 20 | 20/04/2020 | 《俄罗斯龙报纸》;《俄罗斯侨报》;《透视俄罗斯》 | <https://m.hhlink.com/top-websites/Russia/Chinese-Media/1> |
| Peru | 13 | 0 | 18/04/2020 | 《秘鲁通》; 《秘华商报》;《公言报》 | https://weixin.sogou.com/weixin?type=2&s_from=input&query=%E7%A7%98%E9%B2%81%E5%8D%8E%E4%BA%BA%E5%AA%92%E4%BD%93&ie=utf8&_sug_=y&_sug_type_=&w=01019900&sut=1999&sst0=1631064086898&lkt=1%2C1631064086773%2C1631064086773 |
| France | 9 | 18 | 20/04/2020 | 《华人街》; RFI中文; 《欧洲时报》 | https://worldchinesemedia.com/ |
| Singapore | 8 | 16 | 17/04/2020 | 《联合早报》;《南洋商报》;《我报》 | <https://m.hhlink.com/%E7%BD%91%E7%AB%99%E7%9B%AE%E5%BD%95/Singapore/%E4%B8%AD%E6%96%87%E5%AA%92%E4%BD%93/1> |
| Brazil | 6 | 32 | 18/04/2020 | 《拉美侨声》;《南美侨报》网; 巴西华人网 | https://worldchinesemedia.com/ |
| Malaysia | 5 | 34 | 19/04/2020 | 《星洲日报》;《南洋商报》;《中国报》 | https://zh.wikipedia.org/wiki/%E9%A6%AC%E4%BE%86%E8%A5%BF%E4%BA%9E%E5%A0%B1%E5%88%8A%E5%88%97%E8%A1%A8 |
| Canada | 5 | 9 | 20/04/2020 | 《加拿大和世界报道》;《多伦多新闻网》;《华侨新报》 | https://worldchinesemedia.com/ |
| Angola | 4 | 4 | 17/04/2020 | 《安哥拉华人报》 | https://worldchinesemedia.com/ |
| Belgium | 4 | 6 | 18/04/2020 | 《欧华商报》;《华商时报》 | https://weixin.sogou.com/weixin?type=2&s_from=input&query=%E6%AF%94%E5%88%A9%E6%97%B6%E5%8D%8E%E4%BA%BA%E5%AA%92%E4%BD%93&ie=utf8&_sug_=y&_sug_type_=&w=01019900&sut=1791&sst0=1631064868774&lkt=1%2C1631064868673%2C1631064868673 |
| Portugal | 3 | 3 | 17/04/2020 | 《葡新报》;《葡华报》;《華人PT》 | [https://weixin.sogou.com/weixin?type=2&s_from=input&query=%E8%91%A1%E8%90%84%E7%89%99%E5%8D%8E%E4%BA%BA%E5%AA%92%E4%BD%93&ie=utf8&_sug_=y&_sug_type_=&w=01019900&sut=5922&sst0=1631065131696&lkt=1%2C1631065131565%2C1631065131565](https://weixin.sogou.com/weixin?type=2&s_from=input&query=%E8%91%A1%E8%90%84%E7%89%99%E5%8D%8E%E4%BA%BA%E5%AA%92%E4%BD%93&ie=utf8&_sug_=y&_sug_type_=&w=01019900&sut=5922&sst0=1631065131696&lkt=1,1631065131565,1631065131565) |
| India | 2 | 0 | 18/04/2020 | 印度华人网; 印度中文网 | https://m.hhlink.com/%E7%BD%91%E7%AB%99%E7%9B%AE%E5%BD%95/%E5%8D%B0%E5%BA%A6/%E4%B8%BB%E6%B5%81%E5%AA%92%E4%BD%93/1 |
| Greece | 2 | 11 | 19/04/2020 | 《中希时报》(希中网); 《希华时讯》 | https://weixin.sogou.com/weixin?type=2&query=%E5%B8%8C%E8%85%8A%E5%8D%8E%E4%BA%BA%E5%AA%92%E4%BD%93&ie=utf8&s_from=input&_sug_=y&_sug_type_=&w=01019900&sut=2922&sst0=1631063775521&lkt=1%2C1631063775417%2C1631063775417 |
| Sweden | 2 | 1 | 16/04/2020 | 《北欧国际新闻》; 《北欧华人报》;《北欧时报》 | https://worldchinesemedia.com/ |
| Saudi Arabia | 1 | 0 | 16/04/2020 | 沙特华人网 | http://www.dubaiqqt.com/SaudiArabia/ |
| Netherlands | 1 | 21 | 16/04/2020 | 《中荷商报》; 《华侨新天地》 | [https://weixin.sogou.com/weixin?type=2&s_from=input&query=%E8%8D%B7%E5%85%B0%E5%8D%8E%E4%BA%BA%E5%AA%92%E4%BD%93&ie=utf8&_sug_=y&_sug_type_=&w=01019900&sut=1943&sst0=1631065498227&lkt=1%2C1631065498123%2C1631065498123](https://weixin.sogou.com/weixin?type=2&s_from=input&query=%E8%8D%B7%E5%85%B0%E5%8D%8E%E4%BA%BA%E5%AA%92%E4%BD%93&ie=utf8&_sug_=y&_sug_type_=&w=01019900&sut=1943&sst0=1631065498227&lkt=1,1631065498123,1631065498123) |
| Switzerland | 1 | 0 | 16/04/2020 | 《欧亚时报》 | [https://weixin.sogou.com/weixin?type=2&s_from=input&query=%E7%91%9E%E5%A3%AB%E5%8D%8E%E4%BA%BA%E5%AA%92%E4%BD%93&ie=utf8&_sug_=y&_sug_type_=&w=01019900&sut=1891&sst0=1631065741797&lkt=1%2C1631065741695%2C1631065741695](https://weixin.sogou.com/weixin?type=2&s_from=input&query=%E7%91%9E%E5%A3%AB%E5%8D%8E%E4%BA%BA%E5%AA%92%E4%BD%93&ie=utf8&_sug_=y&_sug_type_=&w=01019900&sut=1891&sst0=1631065741797&lkt=1,1631065741695,1631065741695) |
| Mexico | 1 | 4 | 16/04/2020 | 《华文时报》; 墨西哥华人网; 墨西哥新闻网 | https://www.hhlink.com/hhViewArea.aspx?AreaID=1917 |
| South Africa | 1 | 6 | 17/04/2020 | 《非洲之声》;《非洲侨报》;《中非新闻》 | Consulted Chinese experts in Africa |
| Vietnam | 1 | 0 | 18/04/2020 | —————— | https://www.hhlink.com/%E5%85%A8%E7%90%83%E5%8D%8E%E4%BA%BA/%E8%B6%8A%E5%8D%97 |
| Kenya | 1 | 6 | 18/04/2020 | 《非洲之声》;《非洲侨报》;《中非新闻》 | Consulted Chinese experts in Africa |
| Guatemala | 1 | 0 | 18/04/2020 | 《南美侨报-危地马拉新声》 | http://www.br-cn.com/product/epaper/ |
| Uraguay | 1 | 0 | 18/04/2020 | —————— | No sourcing information. |
| Chile | 1 | 10 | 18/04/2020 | 智利中文网; 《南美侨报-智利之窗》 | http://www.br-cn.com/product/epaper/ |
| Kuwait | 1 | 0 | 18/04/2020 | —————— | No sourcing information. |
| Lebanon | 1 | 0 | 16/04/2020 | —————— | No sourcing information. |

Note: 1) All financial newspapers were excluded even if ranking was high; 2) News circulation ranking was used as a criteria for countries with more than 3 ethnic Chinese news outlets, some countries had only three or two Chinese ethnic media and they were sourced regardless of ranking; 3) Cells coded as 0 mean no report or ethnic Chinese media was found.

**S10 Table.** **Ethnic Chinese Newspaper Coverage.** List of online ethnic Chinese newspaper reports of discrimination against Chinese during COVID-19 by country.

| **USA (Jan.23-Apr.21, 2020)** | | |
| --- | --- | --- |
| **Newspaper** | **Date** | **News Title** |
| 侨报 | 1/23/2020 | [布碌仑辱骂攻击华女 白女律师认罪获轻判]("http://epaper.uschinapress.com/category/6541-1-23-2020.html) |
| 侨报 | 1/24/2020 | [全美华人剧集城市仇恨犯罪率上升 洛城居冠]("http://epaper.uschinapress.com/category/6542-1-24-2020.html) |
| 侨报 | 1/31/2020 | [因排华污点 加拿大伯克利法学院改名]("http://epaper.uschinapress.com/category/6549-1-31-2020.html) |
| 侨报 | 2/1/2020 | [中国外交官：不建议各国撤侨 呼吁国际社会共同抗击疫情 部分国家已现假借病毒的种族歧视事件]("http://epaper.uschinapress.com/category/6585-2-1-2020.html) |
| 侨报 | 2/2/2020 | [从中国返程 华人自愿选择隔离 美学校致信家长勿孤立中国人 李显龙：排华情绪对防疫无益]("http://epaper.uschinapress.com/category/6586-2-2-2020.html) |
| 侨报 | 2/2/2020 | [莫让病毒引发的偏见成“病毒]("http://epaper.uschinapress.com/category/6586-2-2-2020.html)” |
| 侨报 | 2/3/2020 | [来自中国旅客需在指定机场入境]("http://epaper.uschinapress.com/category/6587-2-3-2020.html) |
| 侨报 | 2/3/2020 | [肯尼迪机场防疫升级 去过湖北有无症状都隔离]("http://epaper.uschinapress.com/category/6587-2-3-2020.html) |
| 侨报 | 2/3/2020 | [排华排不了病毒]("http://epaper.uschinapress.com/category/6587-2-3-2020.html) |
| 侨报 | 2/4/2020 | [疑因戴口罩 亚裔女遭袭]("http://epaper.uschinapress.com/category/6588-2-4-2020.html) |
| 侨报 | 2/4/2020 | [《时代》：疫情暴发应警惕种族主义]("http://epaper.uschinapress.com/category/6588-2-4-2020.html) |
| 侨报 | 2/4/2020 | [俄将遣返新型肺炎患者？俄卫生部：将治疗至痊愈-全球邮轮禁止近日赴华旅客登船 英德各现一起因疫情“仇外”事件]("http://epaper.uschinapress.com/category/6588-2-4-2020.html) |
| 侨报 | 2/4/2020 | [美加强筛查访华反国旅客]("http://epaper.uschinapress.com/category/6588-2-4-2020.html) |
| 侨报 | 2/4/2020 | [怕病毒不去中餐馆 市亿元批此举无知 称不要借机歧视或沾污亚裔]("http://epaper.uschinapress.com/category/6588-2-4-2020.html) |
| 侨报 | 2/4/2020 | [加拿大武汉面馆遭遇人情冷暖 被问是否“卖蝙蝠汤和蛇” 加官员亲自前往就餐支持]("http://epaper.uschinapress.com/category/6588-2-4-2020.html) |
| 侨报 | 2/5/2020 | [WHO: 一些发达国家疫情数据落后-联合国秘书长：应警惕疫情带来歧视]("http://epaper.uschinapress.com/category/6589-2-5-2020.html) |
| 侨报 | 2/5/2020 | [停止歧视！华人不是病毒]("http://epaper.uschinapress.com/category/6589-2-5-2020.html) |
| 侨报 | 2/5/2020 | [拍摄非裔攻击亚裔视频 宋薇：冀提高公众意识]("http://epaper.uschinapress.com/category/6589-2-5-2020.html) |
| 侨报 | 2/5/2020 | [社区组织呼吁勿因疫情歧视亚裔]("http://epaper.uschinapress.com/category/6589-2-5-2020.html) |
| 侨报 | 2/5/2020 | [口罩惹祸：遭歧视冲突频生]("http://epaper.uschinapress.com/category/6589-2-5-2020.html) |
| 侨报 | 2/6/2020 | [从湖北来，乘客纽瓦克机场被强制隔离]("http://epaper.uschinapress.com/category/6590-2-6-2020.html) |
| 侨报 | 2/6/2020 | [疫情严重冲击芝加哥中国城生意]("http://epaper.uschinapress.com/category/6590-2-6-2020.html) |
| 侨报 | 2/6/2020 | [亚裔女戴口罩被攻击 市警仇恨犯罪组展开调查]("http://epaper.uschinapress.com/category/6590-2-6-2020.html) |
| 侨报 | 2/6/2020 | [5分局通报辖区警情 口罩女事件无人联系]("http://epaper.uschinapress.com/category/6590-2-6-2020.html) |
| 侨报 | 2/7/2020 | [破除谣言 卫生局长华埠用餐]("http://epaper.uschinapress.com/category/6591-2-7-2020.html) |
| 侨报 | 2/7/2020 | [哥大图书馆惊现中文“武汉疫情隔离区”]("http://epaper.uschinapress.com/category/6591-2-7-2020.html) |
| 侨报 | 2/7/2020 | [参加学区会遭歧视性拦阻 维权团体发动示威]("http://epaper.uschinapress.com/category/6591-2-7-2020.html) |
| 侨报 | 2/7/2020 | [新型肺炎蔓延 新州华裔学生被称“中国病毒”]("http://epaper.uschinapress.com/category/6591-2-7-2020.html) |
| 侨报 | 2/7/2020 | [戴口罩华女遇袭 布碌仑记者会官民公愤]("http://epaper.uschinapress.com/category/6591-2-7-2020.html) |
| 侨报 | 2/7/2020 | [《华尔街日报》讽中国亚洲病夫遭回怼]("http://epaper.uschinapress.com/category/6591-2-7-2020.html) |
| 侨报 | 2/8/2020 | [华尔街日报刊文中国是亚洲真正病夫 华人白宫请愿促道歉]("http://epaper.uschinapress.com/category/6592-2-8-2020.html) |
| 侨报 | 2/8/2020 | [民代和多个医疗组织反对借新型冠状病毒歧视华裔]("http://epaper.uschinapress.com/category/6592-2-8-2020.html) |
| 侨报 | 2/9/2020 | [中国是亚洲病夫标题非作者所起：回应质疑称 自己并不知编辑会这样去标题]("http://epaper.uschinapress.com/category/6593-2-9-2020.html) |
| 侨报 | 2/9/2020 | [我不是病毒 中国女孩讲述背后故事：希望通过这种方式消除当地民众对病毒的误解]("http://epaper.uschinapress.com/category/6593-2-9-2020.html) |
| 侨报 | 2/10/2020 | [专家作证 反对因疫情而歧视华人]("http://epaper.uschinapress.com/category/6594-2-10-2020.html) |
| 侨报 | 2/10/2020 | [抗疫《华尔街日报》辱华要求道歉 40侨团呼吁示威]("http://epaper.uschinapress.com/category/6594-2-10-2020.html) |
| 侨报 | 2/10/2020 | [消除偏见 多国政要力挺中餐馆]("http://epaper.uschinapress.com/category/6594-2-10-2020.html) |
| 侨报 | 2/10/2020 | [新馆肺炎笼罩：费城华埠超市生意冷清]("http://epaper.uschinapress.com/category/6594-2-10-2020.html) |
| 侨报 | 2/11/2020 | [中国是“觉醒的雄狮”而非“亚洲病夫”]("http://epaper.uschinapress.com/category/6596-2-11-2020.html) |
| 侨报 | 2/11/2020 | [春天将近 中餐业暂遇寒冬]("http://epaper.uschinapress.com/category/6596-2-11-2020.html) |
| 侨报 | 2/11/2020 | [意大利56所学校背中餐挺华人：将向760余名学生供应中餐 对歧视亚裔学生说“不”]("http://epaper.uschinapress.com/category/6596-2-11-2020.html) |
| 侨报 | 2/11/2020 | [中方促《华尔街日报》道歉 该报发布种族主义文章攻击中国 华人白宫请愿撤“病夫”文章]("http://epaper.uschinapress.com/category/6596-2-11-2020.html) |
| 侨报 | 2/12/2020 | [疫情是人类公敌 必须破除种族偏见]("http://epaper.uschinapress.com/category/6597-2-12-2020.html) |
| 侨报 | 2/13/2020 | [因疫情遭歧视 人权团体呼吁积极举报]("http://epaper.uschinapress.com/category/6598-2-13-2020.html) |
| 侨报 | 2/14/2020 | [英国亚裔学生因新冠疫情遭歧视]("http://epaper.uschinapress.com/category/6599-2-14-2020.html) |
| 侨报 | 2/14/2020 | [白思豪、张晟呼吁支持亚裔小商家发展 反对任何歧视华裔行为 挺华社 纽约政要法拉盛用餐]("http://epaper.uschinapress.com/category/6599-2-14-2020.html) |
| 侨报 | 2/15/2020 | [曼哈顿区长高步尔华埠用餐 缓解民众对新冠肺炎恐慌 吸引更多游客参观消费]("http://epaper.uschinapress.com/category/6600-2-15-2020.html) |
| 侨报 | 2/15/2020 | [新冠病毒疫情引恐慌 华埠小企业流失顾客]("http://epaper.uschinapress.com/category/6600-2-15-2020.html) |
| 侨报 | 2/17/2020 | [白思豪怒批华裔乘客 称绝不容忍司机种族歧视]("http://epaper.uschinapress.com/category/6602-2-17-2020.html) |
| 侨报 | 2/18/2020 | [殴伤华裔嫌犯保释 家属痛批保释改革纵容犯罪]("http://epaper.uschinapress.com/category/6603-2-18-2020.html) |
| 侨报 | 2/18/2020 | [海外中餐馆生意跌入寒冬 受疫情影响客流量锐减 部分商家停业 多国政要、媒体为中餐正名]("http://epaper.uschinapress.com/category/6603-2-18-2020.html) |
| 侨报 | 2/18/2020 | [勿因恐慌拒绝到唐人街消费 芝城官方：“中国城很安全”]("http://epaper.uschinapress.com/category/6603-2-18-2020.html) |
| 侨报 | 2/19/2020 | [亚洲多国反对种族歧视 呼吁共克时艰]("http://epaper.uschinapress.com/category/6604-2-19-2020.html) |
| 侨报 | 2/19/2020 | [新冠病毒疫情殃及全美华裔商家]("http://epaper.uschinapress.com/category/6604-2-19-2020.html) |
| 侨报 | 2/19/2020 | [卫生官员强调：勿因疫情而歧视或躲开华人]("http://epaper.uschinapress.com/category/6604-2-19-2020.html) |
| 侨报 | 2/19/2020 | [拒载华客 市长怒文批歧视]("http://epaper.uschinapress.com/category/6604-2-19-2020.html) |
| 侨报 | 2/20/2020 | [支持华裔商家 波士顿市长华埠用餐 波士顿要借力社交媒体为华埠“正名"]("http://epaper.uschinapress.com/category/6605-2-20-2020.html) |
| 侨报 | 2/20/2020 | [俄临时禁制部分中国人入境]("http://epaper.uschinapress.com/category/6605-2-20-2020.html) |
| 侨报 | 2/21/2020 | [肺炎谣言与恐惧严重冲击华人社区 纽约市大邀请专家探讨应对策略]("http://epaper.uschinapress.com/category/6606-2-21-2020.html) |
| 侨报 | 2/21/2020 | [一只口罩告诉了我们很多问题]("http://epaper.uschinapress.com/category/6607-2-22-2020.html) |
| 侨报 | 2/22/2020 | [亚太裔民权组织发表声明：对新冠病毒恐惧不代表可以歧视亚裔]("http://epaper.uschinapress.com/category/6607-2-22-2020.html) |
| 侨报 | 2/22/2020 | [举办新冠病毒主题派对 纽约州大学生惹众怒]("http://epaper.uschinapress.com/category/6607-2-22-2020.html) |
| 侨报 | 2/23/2020 | [搜捕华人间谍涉歧视？美国会议员启动调查]("http://epaper.uschinapress.com/category/6608-2-23-2020.html) |
| 侨报 | 2/23/2020 | [澳主持人发起到华人餐馆就餐活动]("http://epaper.uschinapress.com/category/6608-2-23-2020.html) |
| 侨报 | 2/24/2020 | [中国女生在德被骂”病毒“ 警方控制涉事流浪汉]("http://epaper.uschinapress.com/category/6609-2-24-2020.html) |
| 侨报 | 2/25/2020 | [八大道法生路怒攻击事件 华男被痛殴]("http://epaper.uschinapress.com/category/6610-2-25-2020.html) |
| 侨报 | 2/25/2020 | [华尔街日报拒就辱华文章道歉]("http://epaper.uschinapress.com/category/6610-2-25-2020.html) |
| 侨报 | 2/26/2020 | [旧金山宣布疫情紧急状态：百张病床备好 停止仇视亚裔]("http://epaper.uschinapress.com/category/6611-2-26-2020.html) |
| 侨报 | 2/27/2020 | [难敌疫情恐慌 波城华埠餐厅生意依旧惨淡]("http://epaper.uschinapress.com/category/6612-2-27-2020.html) |
| 侨报 | 2/28/2020 | [海外侨胞反击歧视：挑战面前当休戚与共]("http://epaper.uschinapress.com/category/6614-2-29-2020.html) |
| 侨报 | 2/28/2020 | [华裔拾荒老人遭遇种族歧视 超模吉吉法生谴责]("http://epaper.uschinapress.com/category/6614-2-29-2020.html) |
| 侨报 | 2/29/2020 | [司法部递交上诉文件 力挺推翻哈佛歧视案判决]("http://epaper.uschinapress.com/category/6614-2-29-2020.html) |
| 侨报 | 2/29/2020 | [曼哈塞特宫图书馆宣布 馆长未发表种族歧视言论]("http://epaper.uschinapress.com/category/6614-2-29-2020.html) |
| 侨报 | 3/1/2020 | [朝鲜采取特级防控措施 禁所有外国公民入境]("http://epaper.uschinapress.com/category/6644-3-1-2020.html) |
| 侨报 | 3/1/2020 | [“亚洲面孔”引来恶意]("http://epaper.uschinapress.com/category/6644-3-1-2020.html) |
| 侨报 | 3/1/2020 | [中国老人无故遭辱骂殴打 意歌手怒扇施暴者]("http://epaper.uschinapress.com/category/6644-3-1-2020.html) |
| 侨报 | 3/1/2020 | [加华人遇害 警方定性恐怖主义谋杀]("http://epaper.uschinapress.com/category/6644-3-1-2020.html) |
| 侨报 | 3/1/2020 | [疫情下的美国华人]("http://epaper.uschinapress.com/category/6644-3-1-2020.html) |
| 侨报 | 3/3/2020 | [报道新冠用亚裔配图 纽约邮报引各界谴责]("http://epaper.uschinapress.com/category/6646-3-3-2020.html) |
| 侨报 | 3/3/2020 | [多位民代谴责用亚裔照片报道新冠病毒]("http://epaper.uschinapress.com/category/6646-3-3-2020.html) |
| 侨报 | 3/3/2020 | [台湾旅行团被以色列遣返]("http://epaper.uschinapress.com/category/6646-3-3-2020.html) |
| 侨报 | 3/4/2020 | [助理脸书侮辱华裔惹众怒 冯特丝明日下午华社说明（Frontis）]("http://epaper.uschinapress.com/category/6647-3-4-2020.html) |
| 侨报 | 3/4/2020 | [华裔搭乘公交屡屡遭白眼 八达道侨领呼吁勿纵容歧视]("http://epaper.uschinapress.com/category/6647-3-4-2020.html) |
| 侨报 | 3/4/2020 | [新冠肺炎流言四起 华社商业步入寒冬]("http://epaper.uschinapress.com/category/6647-3-4-2020.html) |
| 侨报 | 3/5/2020 | [中方：中国病毒论极不负责任 病毒溯源工作尚无定论，也能够避免地域污名化]("http://epaper.uschinapress.com/category/6648-3-5-2020.html) |
| 侨报 | 3/5/2020 | [脸书散播排斥华人 冯特斯助理遭开除]("http://epaper.uschinapress.com/category/6648-3-5-2020.html) |
| 侨报 | 3/5/2020 | [非裔社区委员脸书辱亚裔 议员促辞职]("http://epaper.uschinapress.com/category/6648-3-5-2020.html) |
| 侨报 | 3/5/2020 | [非裔地铁攻击华男视频疯传 引排华担忧]("http://epaper.uschinapress.com/category/6648-3-5-2020.html) |
| 侨报 | 3/5/2020 | [不容忍借新冠肺炎 歧视亚裔社区行为]("http://epaper.uschinapress.com/category/6648-3-5-2020.html) |
| 侨报 | 3/6/2020 | [纽市新增两确诊病例 病情严重 市府颁令强制政府雇员检测隔离 市议长：绝不许借疫情歧视亚裔]("http://epaper.uschinapress.com/category/6649-3-6-2020.html) |
| 侨报 | 3/6/2020 | [警调查地铁攻击亚裔案 市府鼓励举报新冠歧视]("http://epaper.uschinapress.com/category/6649-3-6-2020.html) |
| 侨报 | 3/6/2020 | [黎巴嫩中国留学生反歧视：中国人不是病毒]("http://epaper.uschinapress.com/category/6649-3-6-2020.html) |
| 侨报 | 3/7/2020 | [团结抗疫 歧视华人要问责]("http://epaper.uschinapress.com/category/6650-3-7-2020.html) |
| 侨报 | 3/7/2020 | [威廉姆斯谴责因新冠疫情歧视和攻击亚裔]("http://epaper.uschinapress.com/category/6650-3-7-2020.html) |
| 侨报 | 3/7/2020 | [80中国人在俄被隔离始末]("http://epaper.uschinapress.com/category/6650-3-7-2020.html) |
| 侨报 | 3/8/2020 | [因疫情歧视攻击亚裔 守护天使华社巡逻护航]("http://epaper.uschinapress.com/category/6651-3-8-2020.html) |
| 侨报 | 3/8/2020 | [抗议福克斯主持人辱华 闽籍侨团要求其道歉]("http://epaper.uschinapress.com/category/6651-3-8-2020.html) |
| 侨报 | 3/10/2020 | [多民选官员批某些主流媒体 假借新冠疫情煽动种族歧视]("http://epaper.uschinapress.com/category/6653-3-10-2020.html) |
| 侨报 | 3/10/2020 | [德国邮轮公司删除歧视中国人内容]("http://epaper.uschinapress.com/category/6653-3-10-2020.html) |
| 侨报 | 3/11/2020 | [亚裔女子未戴口罩也被挑衅遇袭]("http://epaper.uschinapress.com/category/6654-3-11-2020.html) |
| 侨报 | 3/12/2020 | [又有亚裔因新冠遇袭 葛谟表示将进行调查]("http://epaper.uschinapress.com/category/6655-3-12-2020.html) |
| 侨报 | 3/15/2020 | [借疫情歧视殴打亚裔 男嫌终落网]("http://epaper.uschinapress.com/category/6658-3-15-2020.html) |
| 侨报 | 3/15/2020 | [市人权委员会着手调查涉新冠种族主义行为]("http://epaper.uschinapress.com/category/6658-3-15-2020.html) |
| 侨报 | 3/18/2020 | [特朗普辩称不觉得中国病毒时污名化用法]("http://epaper.uschinapress.com/category/6661-3-18-2020.html) |
| 侨报 | 3/18/2020 | [八大道诊所告示用武汉肺炎惹众怒]("http://epaper.uschinapress.com/category/6661-3-18-2020.html) |
| 侨报 | 3/18/2020 | [八大道中国工商银行门店遭打砸 华人担心是针对华裔的仇恨犯罪 实则为吸毒男子时空闹事]("http://epaper.uschinapress.com/category/6661-3-18-2020.html) |
| 侨报 | 3/18/2020 | [布碌仑区长亚当斯批评特朗普中国病毒言论]("http://epaper.uschinapress.com/category/6661-3-18-2020.html) |
| 侨报 | 3/18/2020 | [强烈反对使用“中国病毒”提法]("http://epaper.uschinapress.com/category/6661-3-18-2020.html) |
| 侨报 | 3/18/2020 | [又一起新冠病毒仇恨犯罪？亚裔女子被骂病毒传播罪魁祸首]("http://epaper.uschinapress.com/category/6661-3-18-2020.html) |
| 侨报 | 3/20/2020 | [“中国病毒说”惹议 美各界呼吁勿贴标签]("http://epaper.uschinapress.com/category/6663-3-20-2020.html) |
| 侨报 | 3/20/2020 | [给新冠肺炎贴上中国标签 民代：歧视置亚裔于危险之中]("http://epaper.uschinapress.com/category/6663-3-20-2020.html) |
| 侨报 | 3/21/2020 | [华人演员发起洗掉仇恨活动]("http://epaper.uschinapress.com/category/6664-3-21-2020.html) |
| 侨报 | 3/22/2020 | [美知名乐队主唱：这非是中国病毒]("http://epaper.uschinapress.com/category/6665-3-22-2020.html) |
| 侨报 | 3/23/2020 | [总统先生，中国病毒的说法让美国华人很受伤]("http://epaper.uschinapress.com/category/6666-3-23-2020.html) |
| 侨报 | 3/25/2020 | [要求停止使用中国病毒之类无名]("http://epaper.uschinapress.com/category/6668-3-25-2020.html) |
| 侨报 | 3/25/2020 | [疫情期社区仇华犯罪增多 闽侨团成立相亲自救组织]("http://epaper.uschinapress.com/category/6668-3-25-2020.html) |
| 侨报 | 3/25/2020 | [英华人律师：对歧视行为说不]("http://epaper.uschinapress.com/category/6668-3-25-2020.html) |
| 侨报 | 3/26/2020 | [参院议员汉化蓬佩奥 别再说武汉病毒]("http://epaper.uschinapress.com/category/6669-3-26-2020.html) |
| 侨报 | 3/26/2020 | [辱华裔传毒欲枪击他 亚裔男布碌仑遭威胁]("http://epaper.uschinapress.com/category/6669-3-26-2020.html) |
| 侨报 | 3/27/2020 | [遏制抹黑亚裔言论 孟昭文力推反歧视法案]("http://epaper.uschinapress.com/category/6670-3-27-2020.html) |
| 侨报 | 3/27/2020 | [国会议员叫蓬佩奥别说武汉病毒]("http://epaper.uschinapress.com/category/6670-3-27-2020.html) |
| 侨报 | 3/27/2020 | [非裔西裔组织声明反歧视 呼吁社会各界支持亚裔社区]("http://epaper.uschinapress.com/category/6670-3-27-2020.html) |
| 侨报 | 3/28/2020 | [FBI警告：针对亚裔仇恨犯罪将增加]("http://epaper.uschinapress.com/category/6671-3-28-2020.html) |
| 侨报 | 3/28/2020 | [新冠期间遇歧视就打911]("http://epaper.uschinapress.com/category/6671-3-28-2020.html) |
| 侨报 | 3/29/2020 | [华裔医生因曝医院抗疫不力被开除]("http://epaper.uschinapress.com/category/6672-3-29-2020.html) |
| 侨报 | 3/30/2020 | [不能让种族主义在美国泛滥]("http://epaper.uschinapress.com/category/6673-3-30-2020.html) |
| 侨报 | 3/30/2020 | [疫情期间仇恨亚裔案飙增 AAARI发起联名抵制歧视]("http://epaper.uschinapress.com/category/6673-3-30-2020.html) |
| 侨报 | 3/30/2020 | [又一起涉新冠仇恨犯罪 亚裔女子遭吐痰殴打入院]("http://epaper.uschinapress.com/category/6673-3-30-2020.html) |
| 侨报 | 3/30/2020 | [#WashTheHate 提醒民众注意 疫情中对亚裔美国人的歧视和暴力行为]("http://epaper.uschinapress.com/category/6673-3-30-2020.html) |
| 侨报 | 4/1/2020 | [湾脊街头惊现仇华涂鸦]("http://epaper.uschinapress.com/category/6702-4-1-2020.html) |
| 侨报 | 4/1/2020 | [马州多位议员呼吁抵制歧视亚裔行为]("http://epaper.uschinapress.com/category/6702-4-1-2020.html) |
| 侨报 | 4/2/2020 | [扬言枪杀华埠所有华人社交媒体帖子宣扬仇恨]("http://epaper.uschinapress.com/category/6703-4-2-2020.html) |
| 侨报 | 4/3/2020 | [针对亚裔仇恨犯罪激增 自疫情爆发以来已出现11起]("http://epaper.uschinapress.com/category/6704-4-3-2020.html) |
| 侨报 | 4/6/2020 | [疫情重歧视增 费城亚裔誓言对抗]("http://epaper.uschinapress.com/category/6707-4-6-2020.html) |
| 侨报 | 4/7/2020 | [疫情当前 明州州长挺亚裔反歧视 开通举报热线]("http://epaper.uschinapress.com/category/6708-4-7-2020.html) |
| 侨报 | 4/7/2020 | [亚裔不是病毒，更不是替罪羊]("http://epaper.uschinapress.com/category/6708-4-7-2020.html) |
| 侨报 | 4/7/2020 | [冒着歧视工作 市长：感谢华人；白思豪视察临时医疗防护用品厂 要求打击仇视亚裔犯罪]("http://epaper.uschinapress.com/category/6708-4-7-2020.html) |
| 侨报 | 4/7/2020 | [遭白男化学液体破脸 华女毁容]("http://epaper.uschinapress.com/category/6708-4-7-2020.html) |
| 侨报 | 4/18/2020 | [DNC：针对亚裔仇恨犯罪必须制止]("http://epaper.uschinapress.com/category/6719-4-18-2020.html) |
| 侨报 | 4/18/2020 | [辱骂殴打亚裔女子 新州女孩被控多罪]("http://epaper.uschinapress.com/category/6719-4-18-2020.html) |
| 侨报 | 4/18/2020 | [反抗针对少数族裔仇恨歧视行为 华裔犹太裔社团联手设网站并推立法]("http://epaper.uschinapress.com/category/6719-4-18-2020.html) |
| 侨报 | 4/10/2020 | [曾将病毒与武汉关联《自然》道歉 如果污名化导致亚洲年轻人离开国际校园 将不啻为一个悲剧]("http://epaper.uschinapress.com/category/6711-4-10-2020.html) |
| 侨报 | 4/10/2020 | [疫情愈演愈烈 法拉盛仇华案频发 奶茶店被砸 路人无端遭辱骂]("http://epaper.uschinapress.com/category/6711-4-10-2020.html) |
| 侨报 | 4/11/2020 | [顾雅明遇袭 成功呵退三劫匪 头部遭攻击 一嫌犯在逃]("http://epaper.uschinapress.com/category/6712-4-11-2020.html) |
| 侨报 | 4/11/2020 | [凯兹呼吁勿忍仇恨犯罪 如遇威胁要及时报警]("http://epaper.uschinapress.com/category/6712-4-11-2020.html) |
| 侨报 | 4/12/2020 | [曾将新冠病毒与武汉关联 《自然》连续三天发文致歉]("http://epaper.uschinapress.com/category/6713-4-12-2020.html) |
| 侨报 | 4/12/2020 | [新冠疫情促歧视华裔犯罪增多]("http://epaper.uschinapress.com/category/6713-4-12-2020.html) |
| 侨报 | 4/12/2020 | [疫情期遇歧视 亚裔群体或不保安 亚美联盟推网上举报表格]("http://epaper.uschinapress.com/category/6713-4-12-2020.html) |
| 侨报 | 4/14/2020 | [全美律师协会讨论：新冠引反亚裔趋势是对民主的威胁]("http://epaper.uschinapress.com/category/6715-4-14-2020.html) |
| 侨报 | 4/14/2020 | [疫情期间遇到入室抢劫，华人如何保护自己？]("http://epaper.uschinapress.com/category/6715-4-14-2020.html) |
| 侨报 | 4/15/2020 | [疫情加剧反亚裔势力抬头 拜登公开谴责]("http://epaper.uschinapress.com/category/6716-4-15-2020.html) |
| 侨报 | 4/17/2020 | [疫情引致全美各地歧视案频发 多族裔人士连线分享反歧视举措]("http://epaper.uschinapress.com/category/6718-4-17-2020.html) |
| 侨报 | 4/17/2020 | [16位知名澳华人呼吁停止种族歧视]("http://epaper.uschinapress.com/category/6718-4-17-2020.html) |
| 侨报 | 4/21/2020 | [英华生别样战疫：反歧视]("http://epaper.uschinapress.com/category/6722-4-21-2020.html) |
| 侨报 | 4/21/2020 | [疫情延歧视增 芝城华人不安]("http://epaper.uschinapress.com/category/6722-4-21-2020.html) |
| 世界新闻网 | --- | --- |
| 星岛日报 | --- | --- |
| **Spain (Jan.23-Apr.20, 2020)** | | |
| **Newspaper** | **Date** | **News Title** |
| 欧华报 | 2/9/2020 | [马德里街头华人小哥：“我不是病毒，请拥抱我吧！”](http://www.nx.chinanews.com/hrzx/2020-02-09/doc-ifztkwce5998225.shtml) |
| 欧华报 | 2/18/2020 | [巴塞华人街头行为艺术 呼吁人们消除歧视]("http://www.ouhua.info/2020/0218/28840.html) |
| 欧华报 | 3/24/2020 | [在海外，遇到种族歧视为什么一定要报案？原因在这里！]("http://www.ouhua.info/2020/0324/29275.html) |
| 欧华报 | 2/3/2020 | [【除了隔离，该做些什么】西卫生部呼吁不要歧视华人 中国开始药物临床试验]("http://www.ouhua.info/2020/0203/28683.html) |
| 欧华报 | 2/5/2020 | [西国王和各政府部门力挺中国 声援华人 官员及记者组团为中餐正名]("http://www.ouhua.info/m/2020/0205/28701.html) |
| 欧华报 | 3/2/2020 | [成为歧视的武器 中国游客少了，损失过大，又来哭惨？]("http://www.ouhua.info/2020/0302/29039.html) |
| 欧华报 | 3/25/2020 | [中国留学生因外出购餐戴口罩被殴打 中使馆回应]("http://www.ouhua.info/m/2020/0325/29295.html) |
| 欧华报 | 2/2/2020 | [美国媒体：“亚洲病夫” VS 西班牙人民：“中国加油”]("http://www.ouhua.info/2020/0206/28711.html) |
| 欧华报 | 2/4/2020 | [“中餐厅别怕，我们来了！” “人不是病毒，排外才是”]("http://www.ouhua.info/2020/0204/28692.html) |
| 欧华报 | 3/9/2020 | [西班牙疫情之下，华人何去何从？]("http://www.ouhua.info/2020/0309/29111.html) |
| 欧华报 | 2/28/2020 | [海外侨胞反击歧视：挑战面前当休戚与共]("http://www.ouhua.info/2020/0228/28981.html) |
| 欧华报 | 2/29/2020 | [【意大利】确诊1128例！0号病人不明 中国人成替罪羊！]("http://www.ouhua.info/m/2020/0229/29024.html) |
| 欧华报 | 1/27/2020 | [Zaragoza华人因中国肆虐 “武汉肺炎”遭当地人“冷眼”]("http://www.ouhua.info/m/2020/0127/28614.html) |
| 欧华报 | 2/1/2020 | [【华人发声】华人代表接受西班牙电视台采访 澄清中国疫情谣言]("http://www.ouhua.info/2020/0201/28670.html) |
| 欧华报 | 2/10/2020 | [驻西使馆和马德里市政府官员走访Usera华人社区]("http://www.ouhua.info/2020/0210/28747.html) |
| 欧华报 | 3/8/2020 | [西班牙抗“疫”前途未卜 马德里市长指责政府]("http://www.ouhua.info/m/2020/0308/29104.html) |
| 欧华报 | 2/5/2020 | [姚飞临时代办就新型冠状病毒疫情举行记者会 驻巴塞总领事林楠约见加区政府官员]("http://www.ouhua.info/2020/0205/28697.html) |
| 欧华报 | 3/2/2020 | [旅西华侨代表：电视节目现场提问西班牙卫生部门负责人西蒙]("http://www.ouhua.info/2020/0302/29037.html) |
| 欧华报 | 4/5/2020 | [海外留守记丨孤独是留学生的必修课]("http://www.ouhua.info/2020/0405/29444.html) |
| 欧华报 | 3/13/2020 | [【马德里大区关店】只有书报亭 药店 菜场 超市和食品店可以正常营业]("http://www.ouhua.info/m/2020/0313/29172.html) |
| 欧华报 | 3/26/2020 | [海外侨胞抗疫录\|美国成全球疫情“重灾区” 在那留守的同胞还好吗？]("http://www.ouhua.info/2020/0326/29301.html) |
| 欧华报 | 4/21/2020 | [被收养华裔女孩感染后痊愈：只因亚洲面孔受到种族主义侵害]("http://www.ouhua.info/2020/0421/29674.html) |
| 欧华报 | 2/13/2020 | 受疫情影响 西班牙综艺El Hormiguero华裔嘉宾YIBING也遭歧视 |
| 欧华报 | 3/11/2020 | [美国华裔在马德里Embajadores被暴打！昏迷两天！]("http://www.elmandarin.info/2020/0311/29142.html) |
| 西班牙侨声报 | 2/4/2020 | [中国驻西班牙使馆就纺织因疫情产生歧视中国公民现象做西政府工作](https://weixin.sogou.com/link?url=dn9a_-gY295K0Rci_xozVXfdMkSQTLW6cwJThYulHEtVjXrGTiVgSyYKEvoYXj7uU0gRtS2CrT0MNh4svKnPglqXa8Fplpd9BQu1TMmoys1nyyJHUvLF-PifaUo43RkPVlM3UiUn0NIbi555r2b41R-15RJJBaGdE3Oj-t_HOEvdDT_TQrGhjk6fQ-9MXqN-1ksyG8vWfvTy6-tKnzSzIiT9S8f45Z) |
| 西班牙侨声报 | 2/5/2020 | [女留学生被殴打，华人遭歧视！种族歧视蔓延速度比病毒快多了](https://weixin.sogou.com/link?url=dn9a_-gY295K0Rci_xozVXfdMkSQTLW6cwJThYulHEtVjXrGTiVgSyYKEvoYXj7u5cqWCQeUDGAMNh4svKnPglqXa8Fplpd9BQu1TMmoys1nyyJHUvLF-PifaUo43RkPVlM3UiUn0NIbi555r2b41bd9p_VvyJQ4fGQdpb0Z7Keh85yMqyGU98oP8EsjjhrB304Ajb7BwJi6ombeFGaTAJv5oFpHP8) |
| 西班牙侨声报 | 2/6/2020 | [善意无国界！西班牙小哥怒怼疫情谣言 国内外网友齐点赞](https://weixin.sogou.com/link?url=dn9a_-gY295K0Rci_xozVXfdMkSQTLW6cwJThYulHEtVjXrGTiVgSyYKEvoYXj7uNRy7pnPMqIYMNh4svKnPglqXa8Fplpd9BQu1TMmoys1nyyJHUvLF-PifaUo43RkPVlM3UiUn0NIbi555r2b41RWSftQWGc4ZMxFn5GjKwOkVTvou7dRaS6TLiZsE4rsL69aeuS4n8KzSCBOQ8OiYgCcVRfkSAy) |
| 西班牙侨声报 | 2/7/2020 | [世界观\|疫情面前，“恐华症”死灰复燃](https://weixin.sogou.com/link?url=dn9a_-gY295K0Rci_xozVXfdMkSQTLW6cwJThYulHEtVjXrGTiVgSyYKEvoYXj7u38-XWWcTYWsMNh4svKnPglqXa8Fplpd9BQu1TMmoys1nyyJHUvLF-PifaUo43RkPVlM3UiUn0NIbi555r2b41Z42CnGtCiWuJDLM99L18tYpYLyG8hjlogOv8X4cE78ch0VnyFcGtDKUbDOdgZ3GRxEm8ojb7o) |
| 西班牙侨声报 | 2/9/2020 | [开始骂中国亚洲病夫了？对比美国H1N1，中国这次疫情有些不对劲](https://weixin.sogou.com/link?url=dn9a_-gY295K0Rci_xozVXfdMkSQTLW6cwJThYulHEtVjXrGTiVgSyYKEvoYXj7uHnuKSGaz1zAMNh4svKnPglqXa8Fplpd9BQu1TMmoys1nyyJHUvLF-PifaUo43RkPVlM3UiUn0NIbi555r2b41UvgkijrUo7s7lwqpZGoEL2xfm1pBbZz5b1dbeQuHTymZGAomMjmPh9FhT6PZsLs7grD5lbzpx) |
| 西班牙侨声报 | 2/10/2020 | [疫情下海外中餐业受挫，各方发声为中餐“正名”](https://weixin.sogou.com/link?url=dn9a_-gY295K0Rci_xozVXfdMkSQTLW6cwJThYulHEtVjXrGTiVgSyYKEvoYXj7uuxHyfOHq66YMNh4svKnPglqXa8Fplpd9BQu1TMmoys1nyyJHUvLF-PifaUo43RkPVlM3UiUn0NIbi555r2b41fWO8eBiWYXfeJtXVj_yz1qusVvsm_Qc0pUdxIzuQs3Lkb-lb_eV0cX54XeQL2HQn3FvGH2hFb) |
| 西班牙侨声报 | 2/11/2020 | [驻西班牙使馆临时代办姚飞与马德里市区政府代表一起走访乌塞拉华侨华人聚集区](https://weixin.sogou.com/link?url=dn9a_-gY295K0Rci_xozVXfdMkSQTLW6cwJThYulHEtVjXrGTiVgSyYKEvoYXj7urWKOTKupqCsMNh4svKnPglqXa8Fplpd9BQu1TMmoys1nyyJHUvLF-PifaUo43RkPVlM3UiUn0NIbi555r2b41bUI1mrZj2km3-SRerCY5a24xOmIPxb4coBodcODT_2IrztptxZ5LX-MeZWcczuj-m6dsNLOKo) |
| 西班牙侨声报 | 2/12/2020 | [加泰罗尼亚自治区政府就新型冠状病毒肺炎疫情发表公报](https://weixin.sogou.com/link?url=dn9a_-gY295K0Rci_xozVXfdMkSQTLW6cwJThYulHEtVjXrGTiVgSyYKEvoYXj7uwWw880IOwxYMNh4svKnPglqXa8Fplpd9BQu1TMmoys1nyyJHUvLF-PifaUo43RkPVlM3UiUn0NIbi555r2b41R4MUfB5sKIWsshE4Is5-NV4C94oz-gYJNJ2b5mGby2QAIRt-bAsKOlYABLVmIZm_67-Pk495C) |
| 西班牙侨声报 | 2/14/2020 | [西班牙\|“信心中国”国际友人为中国战胜疫情加油](https://weixin.sogou.com/link?url=dn9a_-gY295K0Rci_xozVXfdMkSQTLW6cwJThYulHEtVjXrGTiVgSyYKEvoYXj7uprd_yvcRfWYMNh4svKnPglqXa8Fplpd9BQu1TMmoys1nyyJHUvLF-PifaUo43RkPVlM3UiUn0NIbi555r2b41exhaGOOD5gY-tsQhLLp19vqS77T8MfOSW2xpg9ZLUFbhh8FXk8k742kXAvXv_yr7GssQaVGZN) |
| 西班牙侨声报 | 2/16/2020 | [在西班牙遇到歧视怎么办？](https://weixin.sogou.com/link?url=dn9a_-gY295K0Rci_xozVXfdMkSQTLW6cwJThYulHEtVjXrGTiVgSyYKEvoYXj7uqUIEvsm3ljQMNh4svKnPglqXa8Fplpd9BQu1TMmoys1nyyJHUvLF-PifaUo43RkPVlM3UiUn0NIbi555r2b41QturwAS2LkrE0eOV7WRacjREraTL6mmezuQMOSCEPz4wrlPp7K-riyxarTcqxhBVAOyi2-c04) |
| 西班牙侨声报 | 2/16/2020 | [驻巴塞罗那总领事林楠出席巴塞罗那足球俱乐部支持中国人民抗击新冠肺炎疫情活动](https://weixin.sogou.com/link?url=dn9a_-gY295K0Rci_xozVXfdMkSQTLW6cwJThYulHEtVjXrGTiVgSyYKEvoYXj7uU6RdRDEgmooMNh4svKnPglqXa8Fplpd9BQu1TMmoys1nyyJHUvLF-PifaUo43RkPVlM3UiUn0NIbi555r2b41ROMI4avQKKbmwzn-LXJG9jv6d-CKBesUa-vRtFFAhWL-Ose8caD6IbtV1nEWKAXe-ic_pGhty) |
| 西班牙侨声报 | 2/28/2020 | 关于疫情，中国驻西班牙使领馆想对你们说 |
| 西班牙侨声报 | 3/7/2020 | [【关注】面对疫情扩散旅西侨胞举步维艰](https://weixin.sogou.com/link?url=dn9a_-gY295K0Rci_xozVXfdMkSQTLW6cwJThYulHEtVjXrGTiVgSyYKEvoYXj7uNFYvhyFNMrEMNh4svKnPglqXa8Fplpd9BQu1TMmoys1nyyJHUvLF-PifaUo43RkPVlM3UiUn0NIbi555r2b41dbKrtptjaSeVPjzd3-eP2JwyqOBme2CRi7xk4O588_mSfsgWk2V-dmr4lwGc62hGw0ILWVlkn) |
| 西班牙侨声报 | 3/10/2020 | [西班牙疫情之下，华人何去何从？](https://weixin.sogou.com/link?url=dn9a_-gY295K0Rci_xozVXfdMkSQTLW6cwJThYulHEtVjXrGTiVgSyYKEvoYXj7ugsm2C8oN0MoMNh4svKnPglqXa8Fplpd9BQu1TMmoys1nyyJHUvLF-PifaUo43RkPVlM3UiUn0NIbi555r2b41XHnTH8nsq4ga9IMNxIAB3ziTSMWHKCgvJYffQi2L4oZQwL6-LZpGCT4UQu1_ltKuLE1hVruAS) |
| 西班牙侨声报 | 3/11/2020 | [疫情之下，如何看待“中国人在海外遭歧视”？](https://weixin.sogou.com/link?url=dn9a_-gY295K0Rci_xozVXfdMkSQTLW6cwJThYulHEtVjXrGTiVgSyYKEvoYXj7uUn3dG11MPfcMNh4svKnPglqXa8Fplpd9BQu1TMmoys1nyyJHUvLF-PifaUo43RkPVlM3UiUn0NIbi555r2b41X_o8hKUH5qblly_4cXOUOR48qLjoveavyZnSobmmfFICUVDWrC_gO0o5aCqL4-twJ5FVBJWk5) |
| 西班牙侨声报 | 4/3/2020 | [全球确诊超50万，在国外被打的华人决定做一件事](https://weixin.sogou.com/link?url=dn9a_-gY295K0Rci_xozVXfdMkSQTLW6cwJThYulHEtVjXrGTiVgSyYKEvoYXj7uRIsgJrlRRMwMNh4svKnPglqXa8Fplpd9BQu1TMmoys1nyyJHUvLF-PifaUo43RkPVlM3UiUn0NIbi555r2b41TPs-q0xnTouczKmgBCC7xWs-3SWuoJAbKK7BOm1V3goVf2sNPR6jjeK742L1YFkI_YK0bbaHU) |
| 西班牙侨声报 | 4/9/2020 | 西班牙女歌手因新冠蔓延歧视中国人：不想在有中国人的地方呼吸 |
| 西班牙侨声报 | 4/9/2020 | 特朗普指责世卫组织以“中国为中心”，谭德塞回应：不要将新冠病毒政治化 |
| 西班牙侨声报 | 4/15/2020 | [疫情大流行下的全球舆论战：破山中贼易，破心中贼难](https://weixin.sogou.com/link?url=dn9a_-gY295K0Rci_xozVXfdMkSQTLW6cwJThYulHEtVjXrGTiVgSyYKEvoYXj7uP_5FngoxgeYMNh4svKnPglqXa8Fplpd9BQu1TMmoys1nyyJHUvLF-PifaUo43RkPVlM3UiUn0NIbi555r2b41cnrChMH1fQinUefP7L4jWJ9VmyB8yflpfu5FEJ2DqBl2GTQKFZZalx-q4NTmq9Y0XY0_4hVV8) |
| 西班牙侨声报 | 4/17/2020 | 疫情中美国歧视和攻击亚裔现象抬头，留学生遭遇令人担忧 |
| 华新报（欧浪网） | 2/1/2020 | [世卫宣布PHEIC但力挺中国：不要限制与中国来往，不要造谣抹黑中国](https://weixin.sogou.com/link?url=dn9a_-gY295K0Rci_xozVXfdMkSQTLW6cwJThYulHEtVjXrGTiVgSyYKEvoYXj7uJ11f5xSQlhAMNh4svKnPglqXa8Fplpd9GHL9geoS6s22X5AA6vxNVrf5wrTBJGgTAkEZ2IEqpw2OGkwYpTWyKLGv6zyFSY2Q9SBbWnoG4nXUydMl7G9PmbjDfF5vIrClDixPcf-zOC3JgcgWECt18cP2l0pv58) |
| 华新报（欧浪网） | 2/4/2020 | [华人太难了！疫情恐惧型种族歧视开始蔓延](https://weixin.sogou.com/link?url=dn9a_-gY295K0Rci_xozVXfdMkSQTLW6cwJThYulHEtVjXrGTiVgSyYKEvoYXj7uQVWhUtLimekMNh4svKnPglqXa8Fplpd9GHL9geoS6s22X5AA6vxNVrf5wrTBJGgTAkEZ2IEqpw2OGkwYpTWyKMdB3JKg3bidc0AfMBweT0ETA7C2uUw4t2Bv7yov3314DFshhmGq35dZRf44Nz4JAH7M7niA0B) |
| 华新报（欧浪网） | 2/5/2020 | [酒吧禁止中国留学生入内 涉嫌歧视面临一个月停业整顿](https://weixin.sogou.com/link?url=dn9a_-gY295K0Rci_xozVXfdMkSQTLW6cwJThYulHEtVjXrGTiVgSyYKEvoYXj7uHJr06bysvTsMNh4svKnPglqXa8Fplpd9GHL9geoS6s22X5AA6vxNVrf5wrTBJGgTAkEZ2IEqpw2OGkwYpTWyKNABMpjKuRS5E2dLZ6EDS3vInnwQT2KZC44jn5HlqF6F3Ll6kfsqNHJW22kQfx4l9pFdk9C3oo) |
| 华新报（欧浪网） | 2/5/2020 | [共同努力战胜疫情，姚飞代办今日举行记者会](https://weixin.sogou.com/link?url=dn9a_-gY295K0Rci_xozVXfdMkSQTLW6cwJThYulHEtVjXrGTiVgSyYKEvoYXj7uF9MvkH_GD4wMNh4svKnPglqXa8Fplpd9GHL9geoS6s22X5AA6vxNVrf5wrTBJGgTAkEZ2IEqpw2OGkwYpTWyKGRU4S9JA7qmF15rIKm6U3VMb0_RCtGceUEU-PC9gfCwP08dai5DcCFMAQ60UdenDGa3SaS9gX) |
| 华新报（欧浪网） | 2/9/2020 | [在美留学生发起签名活动 抗议《华尔街日报》发表“中国是亚洲真正的病夫”文章](https://weixin.sogou.com/link?url=dn9a_-gY295K0Rci_xozVXfdMkSQTLW6cwJThYulHEtVjXrGTiVgSyYKEvoYXj7ufpjhsYiohAUMNh4svKnPglqXa8Fplpd9GHL9geoS6s22X5AA6vxNVrf5wrTBJGgTAkEZ2IEqpw2OGkwYpTWyKOFYuP0SEyLEQGsrqFv6wlIWM715MqkBRU1X76Ke4INyb25G6SBOxQ9uve67YaV-2Qz7F7QE0Y) |
| 华新报（欧浪网） | 2/10/2020 | 抵御武汉病毒，反对种族歧视 |
| 华新报（欧浪网） | 2/11/2020 | [西班牙人为新肺炎大炸锅 抵制中国人入境情绪上升](https://weixin.sogou.com/link?url=dn9a_-gY295K0Rci_xozVXfdMkSQTLW6cwJThYulHEtVjXrGTiVgSyYKEvoYXj7uY9eFpciKxZsMNh4svKnPglqXa8Fplpd9GHL9geoS6s22X5AA6vxNVrf5wrTBJGgTAkEZ2IEqpw2OGkwYpTWyKHHZIGLIoRZglLWQtfc9PXPMYzdACHmHVFKlqIEMPkSdwfpRMDadbvQl5JJWdrH-JgF2gpvaTh) |
| 华新报（欧浪网） | 2/12/2020 | [加泰罗尼亚自治区政府就新型冠状病毒肺炎疫情发表公报](https://weixin.sogou.com/link?url=dn9a_-gY295K0Rci_xozVXfdMkSQTLW6cwJThYulHEtVjXrGTiVgSyYKEvoYXj7uFbwlbc81VLcMNh4svKnPglqXa8Fplpd9GHL9geoS6s22X5AA6vxNVrf5wrTBJGgTAkEZ2IEqpw2OGkwYpTWyKG-nPwy-H0WdKHtOVFmj0ZOMoXUid9pxA1FQf1lsnuhyEw1mw6uJIADOk0EYE-4zeea3F_TwBp) |
| 华新报（欧浪网） | 2/13/2020 | [官宣！旅西球员高雷雷加盟西乙球队 疫情面前心系祖国](https://weixin.sogou.com/link?url=dn9a_-gY295K0Rci_xozVXfdMkSQTLW6cwJThYulHEtVjXrGTiVgSyYKEvoYXj7u51Ffxd-6YDcMNh4svKnPglqXa8Fplpd9GHL9geoS6s22X5AA6vxNVrf5wrTBJGgTAkEZ2IEqpw2OGkwYpTWyKM2SXDe-v-tUrKddnDjR0u8U1iprUze3lp4cnWEs1Cnudd6UeY_lMHF2817gNugSqELQobaaaG) |
| 华新报（欧浪网） | 2/13/2020 | [姚飞临时代办就当前新冠肺炎疫情接受西班牙COPE广播电台电话连线采访](https://weixin.sogou.com/link?url=dn9a_-gY295K0Rci_xozVXfdMkSQTLW6cwJThYulHEtVjXrGTiVgSyYKEvoYXj7uKvO45Qx8OK8MNh4svKnPglqXa8Fplpd9GHL9geoS6s22X5AA6vxNVrf5wrTBJGgTAkEZ2IEqpw2OGkwYpTWyKEc3NID9peootwQB05U8eSO1hUF5iBn3BN-0o0RvtbdvSOC9YMLh0c8YhSijfIVKfDjY-Ghma_) |
| 华新报（欧浪网） | 2/28/2020 | 热爱弗朗哥的华人被拒绝续约 或是意识形态歧视 |
| 华新报（欧浪网） | 3/2/2020 | 西班牙防疫全攻略 |
| 华新报（欧浪网） | 3/18/2020 | [秘鲁作家不当言论惹怒中国 外交部斥责其极端不负责任](https://weixin.sogou.com/link?url=dn9a_-gY295K0Rci_xozVXfdMkSQTLW6cwJThYulHEtVjXrGTiVgSyYKEvoYXj7u3HtljdK2kDYMNh4svKnPglqXa8Fplpd9GHL9geoS6s22X5AA6vxNVrf5wrTBJGgTAkEZ2IEqpw2OGkwYpTWyKKBoCDbp3nBiFjzn2urgs7wWkE5hLiyLrPqYchsOB9eXEJB-kP4qKubXvVCVVGGv6m8g0hCKwL) |
| 华新报（欧浪网） | 3/23/2020 | [围绕病毒来源的说法持续升级 美国总统特朗普将冠状病毒妄称“中国病毒”](https://weixin.sogou.com/link?url=dn9a_-gY295K0Rci_xozVXfdMkSQTLW6cwJThYulHEtVjXrGTiVgSyYKEvoYXj7uGWYHKYb0SKkMNh4svKnPglqXa8Fplpd9GHL9geoS6s22X5AA6vxNVrf5wrTBJGgTAkEZ2IEqpw2OGkwYpTWyKKfUU4wCxCLN-dBaDocMuM6EBQoKI3nmUqqNNCIvBJjcwjHR_zJl6WZQY8GcvP1egKP3yp0HKm) |
| 华新报（欧浪网） | 3/24/2020 | [美律师团要求中国赔偿 醒醒吧！中国已经崛起！](https://weixin.sogou.com/link?url=dn9a_-gY295K0Rci_xozVXfdMkSQTLW6cwJThYulHEtVjXrGTiVgSyYKEvoYXj7usa4LTMNyaqwMNh4svKnPglqXa8Fplpd9GHL9geoS6s22X5AA6vxNVrf5wrTBJGgTAkEZ2IEqpw2OGkwYpTWyKER5UJKhfxA7h3oopgrGSJv4ZVa1t_O3I6AoTW2nUHCIZExpFTHtgHltkEe7pqMCO80hrNGQH-) |
| 华新报（欧浪网） | 3/25/2020 | 一曲“中国病毒”成为周日网络点击量冠军 到底谁是“病毒本身”？ |
| 华新报（欧浪网） | 4/20/2020 | 联合国秘书长谈新冠疫情 全球应拒绝传播错误信息 |
| 华新报（欧浪网） | 4/20/2020 | 海外华人多难：确诊不敢去医院，“靠自身免疫力扛” |
| **UK (Jan.23-Apr.19, 2020)** | | |
| **Newspaper** | **Date** | **News Title** |
| 英中时报 | 3/13/2020 | [警方呼吁华人举报仇恨犯罪](http://epaper.ukchinese.com/flipV5.html#/ISSN2041207X/20200313/123/4) |
| 英中时报 | 3/13/2020 | [害怕种族主义 中国留学生不敢戴口罩](http://epaper.ukchinese.com/flipV5.html#/ISSN2041207X/20200313/123/4) |
| 英中时报 | 3/13/2020 | [华生地铁咳嗽 被斥“滚回中国](http://epaper.ukchinese.com/flipV5.html#/ISSN2041207X/20200313/123/4)” |
| 英中时报 | 3/13/2020 | [仇恨犯罪增加 华人学生被打至下颌脱臼](http://epaper.ukchinese.com/flipV5.html#/ISSN2041207X/20200313/123/4) |
| 英中时报 | 3/13/2020 | [戴不戴口罩？太难了](http://epaper.ukchinese.com/flipV5.html#/ISSN2041207X/20200313/123/4) |
| 英中时报 | 2/7/2020 | [华人不是病毒 歧视才是病毒](http://epaper.ukchinese.com/flipV5.html#/ISSN2041207X/20200207/122/1) |
| 英中时报 | 2/7/2020 | [中国留学生抗议校方突然取消庆祝活动](http://epaper.ukchinese.com/flipV5.html#/ISSN2041207X/20200207/122/1) |
| 英中时报 | 2/7/2020 | [中国驻英国大使：应共同反对任何侮辱性、歧视性的言行](http://epaper.ukchinese.com/flipV5.html#/ISSN2041207X/20200207/122/1) |
| 英中时报 | 2/7/2020 | [英国卫生大臣：反对任何针对华人社区的种族歧视](http://epaper.ukchinese.com/flipV5.html#/ISSN2041207X/20200207/122/1) |
| 英中时报 | 2/7/2020 | [英国华人发声：我们不是病毒](http://epaper.ukchinese.com/flipV5.html#/ISSN2041207X/20200207/122/1) |
| 英中时报 | 1/31/2020 | [警惕“恐疫”导致“排华”](http://epaper.ukchinese.com/flipV5.html#/ISSN2041207X/20200131/121/1) |
| 英中时报 | 1/31/2020 | [英国主持人嘲笑华人“Ching chang chong”](http://epaper.ukchinese.com/flipV5.html#/ISSN2041207X/20200131/121/1) |
| 英中时报 | 4/7/2020 | [律师讲解英国华人遭遇歧视时如何用法律保护自己]("http://ukchinese.com/2020/04/07/%e5%be%8b%e5%b8%88%e8%ae%b2%e8%a7%a3%e8%8b%b1%e5%9b%bd%e5%8d%8e%e4%ba%ba%e9%81%ad%e9%81%87%e6%ad%a7%e8%a7%86%e6%97%b6%e5%a6%82%e4%bd%95%e7%94%a8%e6%b3%95%e5%be%8b%e4%bf%9d%e6%8a%a4%e8%87%aa%e5%b7%b1/) |
| 英中时报 | 2/19/2020 | [疫情当下，在英华人遇到歧视该怎么办？朱小久律师这样告诉我们！]("http://ukchinese.com/2020/02/19/%e7%96%ab%e6%83%85%e5%bd%93%e4%b8%8b%ef%bc%8c%e5%9c%a8%e8%8b%b1%e5%8d%8e%e4%ba%ba%e9%81%87%e5%88%b0%e6%ad%a7%e8%a7%86%e8%af%a5%e6%80%8e%e4%b9%88%e5%8a%9e%ef%bc%9f%e6%9c%b1%e5%b0%8f%e4%b9%85%e5%be%8b/) |
| 英中时报 | 2/12/2020 | [佛罗伦萨56所学校给7000名学生供应中餐,声援华人,消除歧视!]("http://ukchinese.com/2020/02/12/%e4%bd%9b%e7%bd%97%e4%bc%a6%e8%90%a856%e6%89%80%e5%ad%a6%e6%a0%a1%e7%bb%997000%e5%90%8d%e5%ad%a6%e7%94%9f%e4%be%9b%e5%ba%94%e4%b8%ad%e9%a4%90%e5%a3%b0%e6%8f%b4%e5%8d%8e%e4%ba%ba%e6%b6%88%e9%99%a4/) |
| 英中时报 | 4/17/2020 | [歧视和攻击亚裔现象抬头 中国在美留学生遭遇令人担忧]("http://ukchinese.com/2020/04/17/%e6%ad%a7%e8%a7%86%e5%92%8c%e6%94%bb%e5%87%bb%e4%ba%9a%e8%a3%94%e7%8e%b0%e8%b1%a1%e6%8a%ac%e5%a4%b4-%e4%b8%ad%e5%9b%bd%e5%9c%a8%e7%be%8e%e7%95%99%e5%ad%a6%e7%94%9f%e9%81%ad%e9%81%87%e4%bb%a4%e4%ba%ba/) |
| 英中时报 | 4/15/2020 | [美国疫情下的华人直捐团体：救人，以及挽救信任]("http://ukchinese.com/2020/04/15/%e7%be%8e%e5%9b%bd%e7%96%ab%e6%83%85%e4%b8%8b%e7%9a%84%e5%8d%8e%e4%ba%ba%e7%9b%b4%e6%8d%90%e5%9b%a2%e4%bd%93%ef%bc%9a%e6%95%91%e4%ba%ba%ef%bc%8c%e4%bb%a5%e5%8f%8a%e6%8c%bd%e6%95%91%e4%bf%a1%e4%bb%bb/) |
| 英中时报 | 4/13/2020 | [多伦多华人护士当街遭口水喷脸,暴力殴打！]("http://ukchinese.com/2020/04/13/%e5%a4%9a%e4%bc%a6%e5%a4%9a%e5%8d%8e%e4%ba%ba%e6%8a%a4%e5%a3%ab%e5%bd%93%e8%a1%97%e9%81%ad%e5%8f%a3%e6%b0%b4%e5%96%b7%e8%84%b8%e6%9a%b4%e5%8a%9b%e6%ae%b4%e6%89%93%ef%bc%81/) |
| 英中时报 | 3/19/2020 | [种族歧视！印度移民被误当成中国人，在以色列被打成重伤]("http://ukchinese.com/2020/03/19/%e7%a7%8d%e6%97%8f%e6%ad%a7%e8%a7%86%ef%bc%81%e5%8d%b0%e5%ba%a6%e7%a7%bb%e6%b0%91%e8%a2%ab%e8%af%af%e5%bd%93%e6%88%90%e4%b8%ad%e5%9b%bd%e4%ba%ba%ef%bc%8c%e5%9c%a8%e4%bb%a5%e8%89%b2%e5%88%97%e8%a2%ab/) |
| 英中时报 | 3/7/2020 | [躲不开的“病毒”歧视：中国留学生被困公交车，华商收到威胁信]("http://ukchinese.com/2020/03/07/%e8%ba%b2%e4%b8%8d%e5%bc%80%e7%9a%84%e7%97%85%e6%af%92%e6%ad%a7%e8%a7%86%ef%bc%9a%e4%b8%ad%e5%9b%bd%e7%95%99%e5%ad%a6%e7%94%9f%e8%a2%ab%e5%9b%b0%e5%85%ac%e4%ba%a4%e8%bd%a6%ef%bc%8c/) |
| 英中时报 | 2/26/2020 | [华人老汉捡易拉罐被非裔美国人欺凌，一边取笑中文，一边恐吓推搡]("http://ukchinese.com/2020/02/26/%e5%8d%8e%e4%ba%ba%e8%80%81%e6%b1%89%e6%8d%a1%e6%98%93%e6%8b%89%e7%bd%90%e8%a2%ab%e9%9d%9e%e8%a3%94%e7%be%8e%e5%9b%bd%e4%ba%ba%e6%ac%ba%e5%87%8c%ef%bc%8c%e4%b8%80%e8%be%b9%e5%8f%96%e7%ac%91%e4%b8%ad/) |
| 英中时报 | 2/17/2020 | [“疫情歧视”是对人权的无情挑战]("http://ukchinese.com/2020/02/17/%e7%96%ab%e6%83%85%e6%ad%a7%e8%a7%86%e6%98%af%e5%af%b9%e4%ba%ba%e6%9d%83%e7%9a%84%e6%97%a0%e6%83%85%e6%8c%91%e6%88%98/) |
| 英中时报 | 2/10/2020 | [因病毒恐慌 福贾省一学校不让8岁华人小孩回去上学]("http://ukchinese.com/2020/02/10/%e5%9b%a0%e7%97%85%e6%af%92%e6%81%90%e6%85%8c-%e7%a6%8f%e8%b4%be%e7%9c%81%e4%b8%80%e5%ad%a6%e6%a0%a1%e4%b8%8d%e8%ae%a98%e5%b2%81%e5%8d%8e%e4%ba%ba%e5%b0%8f%e5%ad%a9%e5%9b%9e%e5%8e%bb%e4%b8%8a%e5%ad%a6/) |
| FT中文网 | 2/3/2020 | [#我不是病毒#：从“武汉肺炎”到反华情绪](https://www.ftchinese.com/story/001086144) |
| FT中文网 | 2/2/2020 | [新型冠状病毒疫情引发针对中国人的种族歧视](https://www.ftchinese.com/story/001086137) |
| FT中文网 | 3/5/2020 | [口罩背后的中英价值观差异](http://www.ftchinese.com/story/001086626?full=y) |
| FT中文网 | 2/6/2020 | [新冠疫情助长美国社会对亚裔的刻板印象](https://www.ftchinese.com/story/001086212) |
| FT中文网 | 3/31/2020 | [疫情里的中国留学生群像：没有“大新闻”渲染的五个真实口述]("https://www.ftchinese.com/story/001087030?archive) |
| FT中文网 | 2/3/2020 | [新型冠狀病毒疫情引髮針對中國人的種族歧視]("http://big5.ftchinese.com/story/001086137?archive) |
| 英国侨报 | 2/5/2020 | [27箱消毒液，暖心拥抱，他们力挺华人反歧视]("https://www.ukjs.co.uk/post/ershiqixiangxiaoduyenuanxinyongbaotamenlitinghuarenfanqishi) |
| 英国侨报 | 2/20/2020 | [国际政要发声反对种族歧视 呼吁各国并肩战“疫”]("https://www.ukjs.co.uk/post/fightingforvirus) |
| **Italy (Jan.23-Apr.23, 2020)** | | |
| **Newspaper** | **Date** | **News Title** |
| 欧洲时报 | 1/25/2020 | [意大利华裔女孩遭种族歧视 数千网民道歉]("http://www.oushinet.com/static/content/europe/italy/2020-01-25/781903143473512448.html) |
| 欧洲时报 | 1/29/2020 | [从歧视武汉人到歧视亚裔，这才是最需要防范的“病毒”]("http://www.oushinet.com/static/content/qj/qjnews/2020-01-29/782926788337598464.html) |
| 欧洲时报 | 2/21/2020 | [意大利一超市收银员发布歧视华人广播]("http://www.oushinet.com/static/content/europe/italy/2020-02-21/781903344393256960.html) |
| 欧洲时报 | 2/28/2020 | [华人老人受歧视被打 意大利歌手仗义出手]("http://www.oushinet.com/static/content/europe/italy/2020-02-28/781903414559768576.html) |
| 欧洲时报 | 3/26/2020 | [仇恨犯罪频发，美亚裔拒当哑裔]("http://paper.oushinet.com/?jid=695266574784790528&iid=825031617725595648&ino=2021-03-26) |
| 欧洲时报 | 3/5/2020 | [美反亚裔仇恨犯罪事件上升]("http://paper.oushinet.com/?jid=695266574784790528&iid=817359602852036608&ino=2021-03-05) |
| 欧洲时报 | 4/9/2020 | [欧洲时报：华人社区防控成效显著 提供抗疫新标准]("http://www.chinaqw.com/hqhr/2020/04-09/252827.shtml) |
| 欧洲时报 | 2/5/2020 | [华人青年意大利街头求拥抱 面对歧视不再沉默！]("http://www.chinaqw.com/hqhr/2020/02-05/244916.shtml) |
| 欧华联合时报 | 3/26/2020 | [罗马最有名的中餐馆关门了，老板娘是浙江农村走出的Gucci女郎](http://www.ouhuatimes.cn/m/view.php?aid=5580) |
| 新华联合时报 | 2/13/2020 | [意大利锡耶纳市中国女子餐馆用餐遭歧视 市长表歉意]("http://www.chinaqw.com/hqhr/2020/02-13/245641.shtml) |
| 新华联合时报 | 3/10/2020 | [意大利一华人餐厅遭盗窃 歹徒凌晨破门而入]("http://www.chinaqw.com/hqhr/2020/03-10/248582.shtml) |
| 新华联合时报 | 1/28/2020 | 13岁华人少年米蓝球场上被诅咒得“武汉肺炎” |
| 新华联合时报 | 2/4/2020 | [“你是中国人，赶紧下车”意大利再现歧视现象]("http://www.newsduan.com/newsyun/HuaMeiJingXuan/OuZhou/ydlxhsb/20200204/140594.html) |
| 新华联合时报 | 1/25/2020 | 米兰华人父子当中被羞辱，华人父亲控诉歧视者用法律保护自己 |
| 新华联合时报 | 1/29/2020 | 拒绝歧视 告诉我们的孩子它叫：CORONA VIRUS |
| 新华联合时报 | 2/1/2020 | 罗马酒吧挂牌禁止中国人入内，罗马音乐学校对中国学生停课 |
| 新华联合时报 | 2/2/2020 | 正能量！意大利公众人物、政府官员以实际行动力挺在意华人，号召意大利人前往华人餐馆何商店 |
| **Germany (Jan.23-Apr.16, 2020)** | | |
| **Newspaper** | **Date** | **News Title** |
| 德国之声中文网 | 2/21/2020 | [多国出现新冠恐慌 一名中国人在俄罗斯遇害]("https://www.dw.com/zh/%E5%A4%9A%E5%9B%BD%E5%87%BA%E7%8E%B0%E6%96%B0%E5%86%A0%E6%81%90%E6%85%8C-%E4%B8%80%E5%90%8D%E4%B8%AD%E5%9B%BD%E4%BA%BA%E5%9C%A8%E4%BF%84%E7%BD%97%E6%96%AF%E9%81%87%E5%AE%B3/a-52459384) |
| 德国之声中文网 | 3/6/2020 | [街访：“武漢肺炎”这种说法是不是歧視？]("https://www.dw.com/zh/%E5%8F%B0%E5%8C%97%E8%A1%97%E8%AE%BF-%E6%AD%A6%E6%BC%A2%E8%82%BA%E7%82%8E%E8%BF%99%E7%A7%8D%E8%AF%B4%E6%B3%95%E6%98%AF%E4%B8%8D%E6%98%AF%E6%AD%A7%E8%A6%96/av-52659507) |
| 德国之声中文网 | 2/20/2020 | [中国驱逐《华尔街日报》记者 美批做法「不成熟」]("https://www.dw.com/zh/%E4%B8%AD%E5%9B%BD%E9%A9%B1%E9%80%90%E5%8D%8E%E5%B0%94%E8%A1%97%E6%97%A5%E6%8A%A5%E8%AE%B0%E8%80%85-%E7%BE%8E%E6%89%B9%E5%81%9A%E6%B3%95%E4%B8%8D%E6%88%90%E7%86%9F/a-52431906) |
| 德国之声中文网 | 1/30/2020 | [德语媒体：“我不是病毒！”]("https://www.dw.com/zh/%E5%BE%B7%E8%AF%AD%E5%AA%92%E4%BD%93%E6%88%91%E4%B8%8D%E6%98%AF%E7%97%85%E6%AF%92/a-52219050) |
| 德国之声中文网 | 1/30/2020 | [“病毒”国旗——丹麦报纸一幅漫画惹怒中国人]("https://www.dw.com/zh/%E7%97%85%E6%AF%92%E5%9B%BD%E6%97%97%E4%B8%B9%E9%BA%A6%E6%8A%A5%E7%BA%B8%E4%B8%80%E5%B9%85%E6%BC%AB%E7%94%BB%E6%83%B9%E6%80%92%E4%B8%AD%E5%9B%BD%E4%BA%BA/a-52203802) |
| 欧洲时报德国 | 2/5/2020 | [疫情当头 德国人不再光顾中餐馆？]("https://www.dw.com/zh/%E7%96%AB%E6%83%85%E5%BD%93%E5%A4%B4-%E5%BE%B7%E5%9B%BD%E4%BA%BA%E4%B8%8D%E5%86%8D%E5%85%89%E9%A1%BE%E4%B8%AD%E9%A4%90%E9%A6%86/a-52267089) |
| 欧洲时报德国 | 2/5/2020 | [德国网民发声支持“我不是病毒”运动：抗击病毒靠洗手 不靠歧视]("http://www.oushinet.com/static/content/europe/GER/2020-02-05/781891095377215488.html) |
| 欧洲时报德国 | 2/13/2020 | [德媒借疫情发表种族主义言论]("http://paper.oushinet.com/?jid=695266014320918528&iid=791235447878582272&ino=2020-02-07) |
| 欧洲时报德国 | 2/13/2020 | [驻德国使馆就德国个别媒体发表种族主义言论表明立场]("http://paper.oushinet.com/?jid=695266014320918528&iid=791235447878582272&ino=2020-02-07) |
| 欧洲时报德国 | 2/13/2020 | [敌人是病毒，不是中国人]("http://paper.oushinet.com/?jid=695266014320918528&iid=791235447878582272&ino=2020-02-07) |
| 欧洲时报德国 | 2/20/2020 | [奥：谴责因疫情歧视华人行为]("http://paper.oushinet.com/?jid=695266014320918528&iid=791236429207306240&ino=2020-02-14) |
| 欧洲时报德国 | 2/20/2020 | [种族歧视在荷兰不得人心]("http://paper.oushinet.com/?jid=695266014320918528&iid=791236429207306240&ino=2020-02-14) |
| 欧洲时报德国 | 2/20/2020 | [荷兰电台就涉华歧视再次道歉]("http://paper.oushinet.com/?jid=695266014320918528&iid=791236429207306240&ino=2020-02-14) |
| 德国华商报 | 3/5/2020 | [辱骂中国女孩醉汉被控制]("http://paper.oushinet.com/?jid=695266014320918528&iid=791236902358351872&ino=2020-02-28) |
| 德国华商报 | 3/12/2020 | [美男子欺凌华裔拾荒者，被控四项罪名]("http://paper.oushinet.com/?jid=695266014320918528&iid=791247704259821568&ino=2020-03-06) |
| 德国华商报 | 2/1/2020 | [德国有人借新冠病毒歧视中国人 这背后的社会现象是什么？](http://epaper.huashangbao.com/flipV5.html#/huashang/20200203/39/40) |
| 德国华商报 | 2/1/2020 | [新冠病毒的报道在德国引发恐惧](http://epaper.huashangbao.com/flipV5.html#/huashang/20200203/39/40) |
| 德国华商报 | 2/1/2020 | [多起华人被歧视事件与《明镜周刊》封面无关](http://epaper.huashangbao.com/flipV5.html#/huashang/20200203/39/40) |
| 德国华商报 | 2/1/2020 | [危难中的德国华人团结起来 怒对以新馆肺炎为名的歧视](http://epaper.huashangbao.com/flipV5.html#/huashang/20200203/39/40) |
| 德国华商报 | 2/1/2020 | [新冠病毒吓到德国人 你最近回中国了吗？](http://epaper.huashangbao.com/flipV5.html#/huashang/20200203/39/40) |
| 德国华商报 | 2/1/2020 | [新冠肺炎严重打击德国华人生计 中餐和亚超面临困境 专家辟谣](http://epaper.huashangbao.com/flipV5.html#/huashang/20200203/39/40) |
| 德国华商报 | 2/15/2020 | [女影星将中国女留学生赶出家门，是歧视还是恐惧？](http://epaper.huashangbao.com/flipV5.html#/huashang/20200218/38/1) |
| **Australia (Jan.23-Apr.21, 2020)** | | |
| **News Site** | **Date** | **News Title** |
| SBS中文 | 4/21/2020 | [在疫情中遭遇种族歧视了吗？中文举报方式已开通]("https://www.sbs.com.au/chinese/mandarin/zh-hans/covid-19-a-new-chinese-tool-has-been-launched-to-report-anti-asian-racism) |
| SBS中文 | 4/3/2020 | [调查：疫情期间你在澳大利亚遭遇到种族歧视吗？]("https://www.sbs.com.au/chinese/mandarin/zh-hans/people-in-australia-experiencing-anti-asian-racism-during-coronavirus-pandemic-urged-to-speak-out) |
| SBS中文 | 4/13/2020 | [16名亚裔澳洲人请愿：停止疫情中激化的种族主义](https://www.sbs.com.au/language/mandarin/zh-hans/unityoverfear-high-profile-asian-australians-start-petition-to-fight-coronavirus-fuelled-racism) |
| SBS中文 | 2/16/2020 | [SBS Language \| 【心理热线】怕歧视？怎么教孩子“怼”回去](https://www.sbs.com.au/chinese/mandarin/zh-hans/audio/corona-virus-how-to-go-against-bullying-and-discrimination) |
| SBS中文 | 4/19/2020 | [墨大两亚裔学生街头遭殴打被喊“回中国去”](https://www.sbs.com.au/language/mandarin/zh-hans/two-chinese-students-assaulted-and-robbed-during-apparent-racist-attack-in-melbourne) |
| SBS中文 | 4/28/2020 | [新冠疫情：墨尔本医院“亚裔长相”员工遇种族歧视](https://www.sbs.com.au/language/mandarin/zh-hans/coronavirus-melbourne-health-workers-of-asian-appearance-report-racial-abuse) |
| SBS中文 | 1/31/2020 | [新冠病毒爆发，针对澳洲华人的种族歧视增多](https://www.sbs.com.au/language/mandarin/zh-hans/this-is-racism-chinese-australians-say-they-ve-faced-increased-hostility-since-the-coronavirus-outbreak-began) |
| SBS中文 | 2/13/2020 | [工党领袖谴责针对澳洲华人的种族主义](https://www.sbs.com.au/language/mandarin/zh-hans/coronavirus-outbreak-federal-opposition-leader-made-a-statement-about-the-racism-against-chinese-australians) |
| SBS中文 | 2/14/2020 | [万人向国会请愿：要求澳媒“停止歧视中国”](https://www.sbs.com.au/language/mandarin/zh-hans/thousands-petition-to-parliament-urging-stop-discrimination-against-china-on-media) |
| SBS中文 | 2/13/2020 | [新冠恐慌：马来西亚华裔女生返澳后被房东拒之门外](https://www.sbs.com.au/language/mandarin/zh-hans/coronavirus-outbreak-a-malaysian-student-was-evicted-by-landlord) |
| SBS中文 | 4/13/2020 | [新冠疫情种族主义：维州手机通讯塔上悬挂纳粹旗和中国国旗](https://www.sbs.com.au/language/mandarin/zh-hans/coronavirus-racist-attack-chinese-and-nazi-flags-hung-atop-phone-tower-in-victoria) |
| SBS中文 | 1/29/2020 | [新冠是“中国病毒”？万人呼吁两家澳媒道歉](https://www.sbs.com.au/chinese/mandarin/zh-hans/chinese-community-require-the-apologies-from-sydney-herald-sun-and-telegraph) |
| SBS中文 | 2/28/2020 | [联合国人权官员：疫情掀起针对亚裔种族的偏见](https://www.sbs.com.au/language/mandarin/zh-hans/virus-discrimination) |
| SBS中文 | 2/8/2020 | [SBS Language \| 华人公共场所戴口罩招人反感？](https://www.sbs.com.au/chinese/mandarin/zh-hans/audio/mask-phobia-vox-pop) |
| SBS中文 | 4/17/2020 | [SBS Language \| 【新冠病毒】本地亚裔歧视激增：被骂「滚回 ...](https://teststar.in/1c009a-paris-roubaix-cancelled-due-rising-coronavirus-cases_language/cantonese/zh-hans/victims-of-coronavirus-fuelled-racism-in-australia-are-speaking-out-about-its-impact) |
| SBS中文 | 4/20/2020 | [“回中国去”：疫情中针对亚裔的种族歧视激增](https://www.sbs.com.au/language/mandarin/zh-hans/victims-of-coronavirus-fuelled-racism-in-australia-are-speaking-out-about-its-impact) |
| ABC中文 | 4/21/2020 | [澳洲华人连遭攻击歧视与反歧视是否被政治化](https://www.abc.net.au/chinese/2020-04-22/racism-and-anti-racism-in-australia/12165804) |
| ABC中文 | 4/3/2020 | [澳洲与新冠病毒相关的种族歧视增多遇到该如何投诉？](https://www.abc.net.au/chinese/2020-04-03/racism-covid-19-coronavirus-outbreak-commissioner-discrimination/12119832) |
| ABC中文 | 2/3/2020 | [新冠状病毒爆发后我在澳洲遇到的歧视](https://www.abc.net.au/chinese/2020-02-03/coronavirus-outbreak-sees-rising-racism/11919044) |
| ABC中文 | 4/15/2020 | [反华与排外：疫情引发的种族歧视问题](https://www.abc.net.au/chinese/2020-04-15/coronavirus-intensifies-anti-foreigner-sentiment-in-china/12147984) |
| ABC中文 | 2/11/2020 | [澳卫生部长坚决谴责对华人社区的歧视](https://www.abc.net.au/chinese/2020-02-11/health-minister-absolutely-denounce-and-reject-discrimination/11954948) |
| ABC中文 | 2/21/2020 | [疫情下媒体报道与华人请愿书带来的思考](https://www.abc.net.au/chinese/2020-02-21/community-petition-against-media-headlines-of-chinese-virus/11989662) |
| ABC中文 | 3/5/2020 | [陈振良：别让疫情下的恐惧煽动种族歧视](https://www.abc.net.au/chinese/2020-03-05/chin-tan-on-racism-and-coronavirus/12029552) |
| ABC中文 | 4/20/2020 | [维州华裔医生遭种族主义谩骂呼吁疫情下要团结](https://www.abc.net.au/chinese/2020-04-20/geelong-doctor-calls-for-unity-after-racist-attack/12165882) |
| ABC中文 | 2/14/2020 | [澳洲人怎么看新冠病毒和其引发的种歧？](https://www.abc.net.au/chinese/2020-02-14/how-do-australians-think-of-coronavirus-and-racism/11967864) |
| ABC中文 | 2/12/2020 | [被指歧视皇家加勒比邮轮取消中国乘客登船禁令](https://www.abc.net.au/chinese/2020-02-12/royal-caribbean-cruises-reverses-ban-on-chinese-nationals/11957866) |
| ABC中文 | 2/5/2020 | [佛罗伦萨市长发起“拥抱一名华人”推特话题引热议](https://www.abc.net.au/chinese/2020-02-06/weibo-reactions-florence-mayors-twitter-on-hugging-a-chinese/11932742) |
| ABC中文 | 3/24/2020 | [塔州香港留学生因戴口罩被打](https://www.abc.net.au/chinese/2020-03-24/hong-kong-student-punched-for-wearing-face-mask/12085460) |
| 澳大利亚人报 | --- | --- |
| **Japan (Jan.23-Apr.18, 2020)** | | |
| **Newspaper** | **Date** | **News Title** |
| 日本新华侨报 | 3/30/3030 | [阻击新冠肺炎疫情战役中的“日本作业”]("http://www.jnocnews.co.jp/news/show.aspx?id=104009) |
| 日本新华侨报 | 1/28/2020 | [日本教授呼吁 国内应加强宣传不能歧视外国人]("http://www.jnocnews.co.jp/news/show.aspx?id=103648) |
| 日本新华侨报 | 2/22/2020 | [疫情威胁下 日本出现歧视中国人事例]("http://www.jnocnews.co.jp/news/show.aspx?id=103826) |
| 日本新华侨报 | 3/5/2020 | [疫情蔓延 横滨中华街老字号遭人投递排华歧视信函]("http://www.jnocnews.co.jp/news/show.aspx?id=103896) |
| 日本东方新报 | 4/13/2020 | [东京涉华凶案是日本人因疫情泄愤？驻日领馆：普通刑事案件](http://www.livejapan.cn/news/news_chn/20200413/26713.html) |
| 日本东方新报 | 3/23/2020 | [在美华人驳“中国病毒”论：别有用心 华人需积极应对]("http://www.livejapan.cn/news/news_ch/20200323/26229.html) |
| 日本东方新报 | 3/20/2020 | [特朗普炒作“中国病毒” 希拉里：用种族主义言论掩盖失职]("http://www.livejapan.cn/news/news_ch/20200320/26204.html) |
| 日本东方新报 | 3/20/2020 | [声音：解“政治病毒”，需走出“心魔”迷航]("http://www.livejapan.cn/news/news_ch/20200320/26190.html) |
| 日本东方新报 | 3/19/2020 | [声音：在文明偏见中“自我膨胀”队战胜病毒毫无助益]("http://www.livejapan.cn/news/news_ch/20200319/26158.html) |
| 日本东方新报 | 2/10/2020 | [海外华侨华人遭不公正对待 华文媒体：歧视抑制不了疫情]("http://www.livejapan.cn/news/news_ch/20200210/25383.html) |
| 日本东方新报 | 2/5/2020 | [澳大利亚多位政要呼吁不应因疫情歧视华人而应伸出援手]("http://www.livejapan.cn/news/news_ch/20200205/25308.html) |
| 日本东方新报 | 2/4/2020 | [日本政府：勿使从武汉归国者成“谣言受害者”]("http://www.livejapan.cn/home/home_headLines/20200204/25286.html) |
| 日本东方新报 | 2/3/2020 | [环球同此凉热，莫让病毒引发的偏见成“病毒”]("http://www.livejapan.cn/news/news_ch/20200203/25242.html) |
| 日本新闻网 | --- | --- |
| **South Korea (Jan.23-Apr.16, 2020)** | | |
| **Newspaper** | **Date** | **News Title** |
| 中央日报 | 1/23/2020 | [武汉肺炎引发歧视？日本商店“禁止中国人出入”引争议]("http://chinese.joins.com/news/articleView.html?idxno=96869) |
| 中央日报 | 1/30/2020 | [武汉肺炎引发恐惧韩国商家“进退两难”]("http://chinese.joins.com/news/articleView.html?idxno=96894) |
| 中央日报 | 2/12/2020 | [世卫组织将新馆肺炎正式命名为“COVID-19”]("http://chinese.joins.com/news/articleView.html?idxno=96991) |
| 朝鲜日报 | 2/10/2020 | [武汉肺炎让欧洲的东方人受到日常化歧视]("https://cnnews.chosun.com/client/news/viw.asp?cate=C01&mcate=M1003&nNewsNumb=20200253121&nidx=53122) |
| 朝鲜日报 | 3/18/2020 | [特朗普称新冠位中国病毒遭中国反驳]("https://cnnews.chosun.com/client/news/viw.asp?cate=C01&mcate=M1003&nNewsNumb=20200353319&nidx=53320) |
| 人民网韩文版 | 3/22/2020 | [韩裔美籍演员：“我是在美国感染新冠病毒的，而不是在中国”](http://korea.people.com.cn/n1/2020/0320/c407864-31641969.html) |
| **Russia (Jan.23-Apr.20, 2020)** | | |
| **Newspaper** | **Date** | **News Title** |
| 龙报 | 2/12/2020 | [俄酒店和参观拒绝中国人将被罚 俄消费者联盟：客人享受的权利与国籍无关]("http://paper.dragonnewsru.com/?noId=136) |
| 龙报 | 3/4/2020 | [中国公民在莫斯科隔离遭虐待？中国驻俄大使馆回应：传言并不属实]("http://paper.dragonnewsru.com/?noId=142) |
| 龙报 | 3/24/2020 | [中国小伙自驾穿越欧亚 “遭遇”俄式隔离](http://www.dragonnewsru.com/home/headlines_home/20200324/107038.html) |
| 龙报 | 3/23/2020 | [美国为何提出“中国病毒”？俄媒：美政府无力应对疫情](http://www.dragonnewsru.com/news/rc_news/20200323/107012.html) |
| 龙报 | 3/20/2020 | [中新时评：海内外皆同胞 同理心可化“干戈”](http://www.dragonnewsru.com/news/ch_news/20200320/106954.html) |
| 龙报 | 3/20/2020 | [解“政治病毒” 美国政客须走出“心魔”迷航](http://www.dragonnewsru.com/news/ch_news/20200320/106942.html) |
| 龙报 | 3/5/2020 | [中国外交部：坚决制止针对中国公民的歧视性事件](http://www.dragonnewsru.com/news/ch_news/20200305/106098.html) |
| 龙报 | 2/27/2020 | [海外侨胞反击歧视：挑战面前当休戚与共](http://www.dragonnewsru.com/news/glo_news/20200227/105762.html) |
| 龙报 | 2/24/2020 | ["歧视视频"走红比利时 欧盟“反歧视”表态助中国抗疫](http://www.dragonnewsru.com/news/glo_news/20200224/105548.html) |
| 龙报 | 2/14/2020 | [因疫情被歧视 美国旧金山16岁亚裔男孩遭霸凌](http://www.dragonnewsru.com/news/glo_news/20200214/105082.html) |
| 龙报 | 2/14/2020 | [欧盟委员会委员：对歧视中国人和其他亚洲人零容忍](http://www.dragonnewsru.com/news/glo_news/20200214/105080.html) |
| 龙报 | 2/14/2020 | [海外华侨华人反对泼脏水和歧视 呼吁尊重和平等](http://www.dragonnewsru.com/news/glo_news/20200214/105078.html) |
| 龙报 | 2/10/2020 | [国际社会批驳对疫情的过激反应和极端言论](http://www.dragonnewsru.com/news/wl_news/20200210/104762.html) |
| 龙报 | 2/10/2020 | [澳大利亚政界呼吁反对因疫情歧视华人](http://www.dragonnewsru.com/news/glo_news/20200210/104730.html) |
| 龙报 | 2/10/2020 | [荷兰政府公开阐明反对种族歧视的坚定立场](http://www.dragonnewsru.com/news/glo_news/20200210/104728.html) |
| 龙报 | 2/10/2020 | [多国民众反歧视、反谣言：没有人是孤岛](http://www.dragonnewsru.com/news/glo_news/20200210/104726.html) |
| 龙报 | 2/7/2020 | [俄罗斯中餐馆：客流减少 但员工没有悲观情绪](http://www.dragonnewsru.com/news/rc_news/20200207/104658.html) |
| 龙报 | 2/6/2020 | ["我不是病毒" 全球连线 海外华人积极响应](http://www.dragonnewsru.com/news/glo_news/20200206/104588.html) |
| 龙报 | 2/5/2020 | [专家：某些国际媒体以“地名”称呼病毒非常不公平](http://www.dragonnewsru.com/news/ch_news/20200205/104474.html) |
| 龙报 | 2/3/2020 | [多国政要反对排华情绪 吁各国携手应对疫情](http://www.dragonnewsru.com/news/wl_news/20200203/104352.html) |
| 俄罗斯侨报 | --- | --- |
| 透视俄罗斯 | --- | --- |
| **Peru (Jan.23-Apr.18, 2020)** | | |
| **Newspaper** | **Date** | **News Title** |
| 秘鲁通 | --- | --- |
| 秘华商报 | --- | --- |
| 公言报 | --- | --- |
| **France (Jan.23-Apr.20, 2020)** | | |
| **Newspaper** | **Date** | **News Title** |
| 法国和世界新闻网 | 2/8/2020 | [意大利总统突访学校 确保中国人不被歧视]("https://eunewsnet.com/2020/02/08/4518/) |
| 法国和世界新闻网 | 2/2/2020 | [新冠病毒疫情扩大 德国杂志封面辱华遭谴责]("https://eunewsnet.com/2020/02/02/4504/) |
| RFI中文网 | 2/14/2020 | [新冠肺炎 独有花木兰不戴口罩 陷种族歧视口水]("https://www.rfi.fr/cn/%E4%B8%AD%E5%9B%BD/20200214-%E6%96%B0%E5%86%A0%E8%82%BA%E7%82%8E-%E7%8B%AC%E6%9C%89%E8%8A%B1%E6%9C%A8%E5%85%B0%E4%B8%8D%E6%88%B4%E5%8F%A3%E7%BD%A9-%E9%99%B7%E7%A7%8D%E6%97%8F%E6%AD%A7%E8%A7%86%E5%8F%A3%E6%B0%B4) |
| RFI中文网 | 2/5/2020 | [【视频】可怕的中国病毒中国人？巴黎华人区有话说]("https://www.rfi.fr/cn/%E4%B8%AD%E5%9B%BD/20200205-%E8%A7%86%E9%A2%91%E5%8F%AF%E6%80%95%E7%9A%84%E4%B8%AD%E5%9B%BD%E7%97%85%E6%AF%92%E4%B8%AD%E5%9B%BD%E4%BA%BA%E5%B7%B4%E9%BB%8E%E5%8D%8E%E4%BA%BA%E5%8C%BA%E6%9C%89%E8%AF%9D%E8%AF%B4) |
| RFI中文网 | 3/13/2020 | [林书豪为中国肺炎名称打抱不平批种族歧视]("https://www.rfi.fr/cn/%E4%B8%AD%E5%9B%BD/20200313-%E6%9E%97%E4%B9%A6%E8%B1%AA%E4%B8%BA%E4%B8%AD%E5%9B%BD%E8%82%BA%E7%82%8E%E5%90%8D%E7%A7%B0%E6%89%93%E6%8A%B1%E4%B8%8D%E5%B9%B3%E6%89%B9%E7%A7%8D%E6%97%8F%E6%AD%A7%E8%A7%86) |
| RFI中文网 | 1/28/2020 | [法国亚裔抗议新冠病毒引发的歧视]("https://www.rfi.fr/cn/%E4%B8%AD%E5%9B%BD/20200128-%E6%B3%95%E5%9B%BD%E4%BA%9A%E8%A3%94%E6%8A%97%E8%AE%AE%E6%96%B0%E5%86%A0%E7%97%85%E6%AF%92%E5%BC%95%E5%8F%91%E7%9A%84%E6%AD%A7%E8%A7%86) |
| RFI中文网 | 3/4/2020 | [新冠病毒肆虐助长意大利反华情绪？]("https://www.rfi.fr/cn/%E6%AC%A7%E6%B4%B2/20200304-%E6%96%B0%E5%86%A0%E7%97%85%E6%AF%92%E8%82%86%E8%99%90%E5%8A%A9%E9%95%BF%E6%84%8F%E5%A4%A7%E5%88%A9%E5%8F%8D%E5%8D%8E%E6%83%85%E7%BB%AA) |
| RFI中文网 | 2/5/2020 | [新冠病毒：法卫生部门与华人协会呼吁停止歧视亚裔族群](https://www.rfi.fr/cn/%E6%B3%95%E5%9B%BD/20200205-%E6%96%B0%E5%86%A0%E7%97%85%E6%AF%92%E6%B3%95%E5%9B%BD%E5%8D%AB%E7%94%9F%E9%83%A8%E9%97%A8%E4%B8%8E%E6%B3%95%E5%9B%BD%E4%B8%AD%E5%9B%BD%E4%BA%BA%E5%8D%8F%E4%BC%9A%E5%91%BC%E5%90%81%E6%AD%A7%E8%A7%86%E4%BA%9) |
| RFI中文网 | 1/30/2020 | [法国亚裔社区因新型冠状病毒受到责难]("https://www.rfi.fr/cn/%E6%B3%95%E5%9B%BD/20200130-%E6%B3%95%E5%9B%BD%E7%9A%84%E4%BA%9A%E6%B4%B2%E7%A4%BE%E5%8C%BA%E5%9B%A0%E6%96%B0%E5%9E%8B%E5%86%A0%E7%8A%B6%E7%97%85%E6%AF%92%E8%80%8C%E5%8F%97%E5%88%B0%E8%B4%A3%E9%9A%BE) |
| 欧洲时报 | 4/7/2020 | [风险群体罗姆人：面临歧视和疫情双重冲击]("http://paper.oushinet.com/?jid=694878770519080960&iid=696976132070703104&ino=2020-04-07) |
| 欧洲时报 | 2/21/2020 | [奥媒：谴责因疫情歧视华人行为]("http://www.oushinet.com/static/content/china/chinanews/2020-02-21/782952779030200320.html) |
| 欧洲时报 | 2/3/2020 | [驻西使馆就防止因疫情歧视中国公民现象做西方政府工作]("http://www.oushinet.com/static/content/qj/qjnews/2020-02-03/782927018802020352.html) |
| 欧洲时报 | 4/9/2020 | [中国驻法使馆就侨领及留学生被扣押事件表态]("http://paper.oushinet.com/?jid=694878770519080960&iid=697708200551514112&ino=2020-04-09) |
| 欧洲时报 | 4/10/2020 | [悲惨世界的中国制造容不得污点]("http://paper.oushinet.com/?jid=694878770519080960&iid=698074375986872320&ino=2020-04-10) |
| 欧洲时报 | 4/14/2020 | [马德里11家药房遭武装抢劫 华人店铺遭法籍男子“拿了火腿就跑”]("http://paper.oushinet.com/?jid=694878770519080960&iid=699496440870731776&ino=2020-04-14) |
| 欧洲时报 | 4/16/2020 | [英国出现针对华人歧视言行甚至暴力行为 驻英使馆表态]("http://www.oushinet.com/static/content/qj/qjnews/2020-04-16/782929718683893760.html) |
| 欧洲时报 | 2/28/2020 | [新冠疫情引起针对亚裔种族歧视 对蔓延的仇恨犯罪说不]("http://www.oushinet.com/static/content/qj/qjnews/2020-02-28/782927807717048320.html) |
| 欧洲时报 | 2/5/2020 | [德国友好人士联合发声：“比病毒更糟糕的是歧视和仇恨”]("http://www.oushinet.com/static/content/qj/qjnews/2020-02-05/782927140134846464.html) |
| **Singapore (Jan.23-Apr.17, 2020)** | | |
| **Newspaper** | **Date** | **News Title** |
| 联合早报 | 3/3/2020 | [疑因冠病疫情受歧视 新加坡学生伦敦街头被打成重伤](https://www.zaobao.com/realtime/singapore/story20200303-1033834) (Singaporean student in London got discriminated and beat up due to COVID) |
| 联合早报 | 2/12/2020 | [“COVID-19”传染病命名之乱](https://www.zaobao.com/znews/singapore/story20200212-1028424) (The messy business of naming “COVID-19”) |
| 联合早报 | 3/7/2020 | [张思浓：冠病疫情与德媒眼中的中国](https://www.zaobao.com/zopinions/views/story20200307-1035022) (Sinong Zhang: Coronavirus and China in German media) |
| 联合早报 | 3/26/2020 | [日副首相踢爆意代表曾呛：黄种人才会得冠病](https://www.zaobao.com/znews/others/story20200326-1040346) (Japan’s vice minister rebukes Italian remark: “only Asians are infected”) |
| 联合早报 | 3/29/2020 | [欧菁仙因疫情遭歧视](https://www.zaobao.com.sg/zentertainment/celebs/story20200329-1041064) (Jingxian Ou was discriminated due to COVID) |
| 联合早报 | 3/13/2020 | [交流站：对抗疫情不应戴上种族有色眼镜](https://www.zaobao.com.sg/forum/views/talk/story20200313-1036677) (Racism is not the cure to COVID) |
| 联合早报 | 3/4/2020 | [新加坡学生在伦敦街头疑因疫情被打成重伤 维文面簿留言慰问](https://www.zaobao.com.sg/news/singapore/story20200304-1033972) (Singaporean student victimized by racist attacks: warm messages on social media) |
| 联合早报 | 1/30/2020 | [新移民家长担心孩子被歧视 希望学校重视心理教育](https://www.zaobao.com.sg/news/singapore/story20200130-1024738) (Singaporean new immigrant parents worry about racism at school) |
| 联合早报 | 3/19/2020 | [特朗普：“中国病毒”说法没错中国未更早通报疫情](https://www.zaobao.com.sg/realtime/world/story20200319-1038360https:/www.zaobao.com.sg/realtime/world/story20200319-1038360) (Trump: “Chinese virus” is not wrong because China hid the virus) |
| 联合早报 | 3/24/2020 | [陆克文：冠病疫情压倒民族主义](https://www.zaobao.com.sg/zopinions/views/story20200324-1039636) (When COVID spread beats nationalism) |
| 联合早报 | 2/11/2020 | [郑永年：美国（西方）外交中的种族主义情结](https://www.zaobao.com.sg/forum/expert/zheng-yong-nian/story20200211-1027971) (Racist mentality in American [Western] diplomacy) |
| 联合早报 | 3/20/2020 | [欧菁仙：巴黎“封人怨”](https://www.zaobao.com.sg/zlifestyle/columns/story20200320-1038523) (Jingxian Ou Commentary: Hatred under lockdown in Paris) |
| 联合早报 | 3/18/2020 | [特朗普称冠病“中国病毒” 中方抗议双方摩擦激化](https://www.zaobao.com.sg/znews/greater-china/story20200318-1037946) (Trump’s “Chinese virus”, China’s anger and worsened diplomatic relations) |
| 联合早报 | 4/4/2020 | [刀口上的口罩封锁与口罩外交](https://www.zaobao.com.sg/wencui/politic/story20200404-1042805) (Mask boycott and mask diplomacy) |
| 联合早报 | 4/13/2020 | [周雁冰：明天会更好](https://www.zaobao.com.sg/zlifestyle/columns/story20200413-1045056) (Tomorrow will be a better day) |
| 联合早报 | 2/13/2020 | [世卫：避免引发歧视病毒正名COVID-19](https://www.zaobao.com.sg/znews/international/story20200213-1028500) (WHO: Coronavirus called COVID-19 to curb racism) |
| 南洋商报 | --- | --- |
| 我报 | --- | --- |
| **Brazil (Jan.23-Apr.22, 2020)** | | |
| **Newspaper** | **Date** | **News Title** |
| 巴西华人网 | 2/5/2020 | [歧视和偏见！疫情之下，海外华侨华人遭遇了什么？又该如何应对？]("https://www.brasilcn.com/article/article_40976.html) |
| 巴西华人网 | 4/5/2020 | [全球确诊超110万，美国超27万！联合国大会：合作抗疫不应歧视排外]("https://www.brasilcn.com/article/article_47378.html) |
| 巴西华人网 | 3/24/2020 | [在海外，遇到种族歧视为什么一定要报案？原因在这里！]("https://www.brasilcn.com/article/article_45883.html) |
| 巴西华人网 | 3/15/2020 | [智利冠状病毒感染升到61例 戴上口罩吓跑顾客与受到歧视怎么办？]("https://www.brasilcn.com/article/article_44814.html) |
| 巴西华人网 | 3/12/2020 | [纽约华人患者推特求救，既没有药还被歧视，请求立刻转院！]("https://www.brasilcn.com/article/article_44467.html) |
| 巴西华人网 | 3/6/2020 | [疫情歧视：华人子女好心为同学带零食 不料食物被扔垃圾桶]("https://www.brasilcn.com/article/article_43733.html) |
| 巴西华人网 | 3/1/2020 | [海外侨胞反击歧视：挑战面前当休戚与共]("https://www.brasilcn.com/article/article_43119.html) |
| 巴西华人网 | 2/4/2020 | [冠状病毒蔓延之际，在巴西的东方人遭遇歧视]("https://www.brasilcn.com/article/article_40895.html) |
| 南美侨报 | 2/23/2020 | [即便“谗人罔极”，亦难“交乱四国”](http://www.br-cn.com/news/gj_news/20200223/143302.html) |
| 南美侨报 | 2/4/2020 | [澳大利亚多位政要吁不应因疫情歧视华人而应伸出援手]("http://www.br-cn.com/news/gj_news/20200204/142393.html) |
| 南美侨报 | 4/18/2020 | 英法等西方诸国近期就疫情问题围攻中国 中方：人命关天 救人要紧 |
| 南美侨报 | 2/8/2020 | [外媒：保持理智停止恐慌 病毒不应成为歧视的接口](http://epaper.br-cn.com/#/issue/2605/1) |
| 拉美侨声 | 1/31/2020 | [仇中？华人遭“病毒化” 孩子活动取消 戴口罩也受辱](http://epaper.br-cn.com/#/issue/2586/2) |
| 拉美侨声 | 2/1/2020 | [新加坡将禁曾访中国旅客入境 无论国籍](http://epaper.br-cn.com/#/issue/2588/2) |
| 拉美侨声 | 2/4/2020 | [中国女生戴口罩上街被袭 中国驻英国使馆提醒](http://epaper.br-cn.com/#/issue/2594/2) |
| 拉美侨声 | 2/5/2020 | [各国政要呼吁民众勿要山东反华情绪](http://epaper.br-cn.com/#/issue/2596/2) |
| 拉美侨声 | 2/6/2020 | [中国人在国外为何无法融入主流社会？](http://epaper.br-cn.com/#/issue/2599/4) |
| 拉美侨声 | 2/11/2020 | [《华尔街日报》刊文称中国是“真正的亚洲病夫”中方回应](http://epaper.br-cn.com/#/issue/2610/1) |
| 拉美侨声 | 2/11/2020 | [疫情阴影下的海外华人](http://epaper.br-cn.com/#/issue/2610/7) |
| 拉美侨声 | 1/20/2020 | [亚洲多国反对种族歧视 呼吁共克时艰](http://epaper.br-cn.com/#/issue/2628/9) |
| 拉美侨声 | 3/3/2020 | [海外侨胞反击歧视：挑战面前当休戚与共](http://epaper.br-cn.com/#/issue/2648/7) |
| 拉美侨声 | 3/6/2020 | [中方：中国病毒论极不负责任 病毒溯源工作尚无定论 应避免地域污名化](http://epaper.br-cn.com/#/issue/2660/9) |
| 拉美侨声 | 3/11/2020 | [消除偏见 多国政要力挺中餐馆 当地官员赴唐人街吃中餐 法媒发文辟谣“蝙蝠汤非武汉菜”](http://epaper.br-cn.com/#/issue/2611/6) |
| 拉美侨声 | 3/12/2020 | [亚裔频遭“疫情歧视” 中方呼吁理性维权](http://epaper.br-cn.com/#/issue/2683/2) |
| 拉美侨声 | 3/17/2020 | [美亚裔纷买枪自保](http://epaper.br-cn.com/#/issue/2694/2) |
| 拉美侨声 | 3/17/2020 | [意大利青田华人的纠结——当地人认为只有中国人会感染](http://epaper.br-cn.com/#/issue/2694/7) |
| 拉美侨声 | 3/19/2020 | [回应特朗普将新冠病毒称“中国病毒” 中国外交部：反对污名化 促美纠正](http://epaper.br-cn.com/#/issue/2699/9) |
| 拉美侨声 | 3/20/2020 | [川普屡称”中国病毒“英美指意在卸责](http://epaper.br-cn.com/#/issue/2701/1) |
| 拉美侨声 | 3/21/2020 | [美华埠九旬报贩疑因歧视受袭](http://epaper.br-cn.com/#/issue/2703/2) |
| 拉美侨声 | 3/25/2020 | [国际主流媒体反对借疫情污名化中国](http://epaper.br-cn.com/#/issue/2711/1) |
| 拉美侨声 | 3/26/2020 | [卸责未得逞 特朗普弃用“中国病毒”](http://epaper.br-cn.com/#/issue/2713/2) |
| 拉美侨声 | 4/11/2020 | [将病毒与武汉关联 《自然》道歉](http://epaper.br-cn.com/#/issue/2745/9) |
| **Malaysia (Jan.23-Apr.19, 2020)** | | |
| **Newspaper** | **Date** | **News Title** |
| 星洲日报 | 3/25/2020 | [陈冠希怒喊“闭嘴和停止歧视”网民酸舔共]("https://www.sinchew.com.my/content/content_2240760.html) |
| 星洲日报 | 2/13/2020 | [“澳房东拒让我进屋”·马华裔留学生控诉种族歧视](https://www.sinchew.com.my/content/content_2216756.html) |
| 星洲日报 | 3/18/2020 | [特朗普称冠病“中国病毒”‧陈法拉怒轰种族歧视](https://www.sinchew.com.my/content/content_2236108.html) |
| 星洲日报 | 3/20/2020 | [林路迪加入“洗掉仇恨”活动·促消除对亚裔歧视](https://www.sinchew.com.my/content/content_2237905.html) |
| 星洲日报 | 3/22/2020 | [不满华人被歧视·向太爆粗诅咒特朗普染疫快点死](https://www.sinchew.com.my/content/content_2238800.html) |
| 星洲日报 | 3/24/2020 | [中国病毒惹议 特朗普改口要保护亚裔美国人]("https://www.sinchew.com.my/content/content_2239972.html) |
| 星洲日报 | 3/20/2020 | [疑因疫情歧视亚裔 美国4人被袭受重创毁容]("https://www.sinchew.com.my/content/content_2239972.html) |
| 星洲日报 | 3/6/2020 | [伦敦疫情歧视袭击案 殴狮城男生 2嫌犯落网]("https://www.sinchew.com.my/content/content_2229376.html) |
| 星洲日报 | 2/15/2020 | [许俊杰#我不是病毒]("https://www.sinchew.com.my/content/content_2217251.html) |
| 南洋商报 | 2/5/2020 | [新冠肺炎蔓延美国纽约市华人疑遭歧视](https://www.enanyang.my/%E5%9B%BD%E9%99%85/%E6%96%B0%E5%86%A0%E8%82%BA%E7%82%8E%E8%94%93%E5%BB%B6-%E7%BE%8E%E5%9B%BD%E7%BA%BD%E7%BA%A6%E5%B8%82%E5%8D%8E%E4%BA%BA%E7%96%91%E9%81%AD%E6%AD%A7%E8%A7%86) |
| 南洋商报 | 2/29/2020 | [疫情折射中国与世界关系复杂“恐华症”死灰复燃](http://www.enanyang.my/%E9%BE%99%E8%85%BE%E4%B8%9C%E7%9B%9F/%E7%96%AB%E6%83%85%E6%8A%98%E5%B0%84%E4%B8%AD%E5%9B%BD%E4%B8%8E%E4%B8%96%E7%95%8C%E5%85%B3%E7%B3%BB%E5%A4%8D%E6%9D%82-%E2%80%9C%E6%81%90%E5%8D%8E%E7%97%87%E2%80%9D%E6%AD%BB%E7%81%B0%E5%A4%8D%E7%87%83) |
| 南洋商报 | 3/22/2020 | [全球大流行 疑因疫情歧视亚裔美4人被袭毁容](https://www.chinapress.com.my/20200322/%E2%97%A4%E5%85%A8%E7%90%83%E5%A4%A7%E6%B5%81%E8%A1%8C%E2%97%A2-%E7%96%91%E5%9B%A0%E7%96%AB%E6%83%85%E6%AD%A7%E8%A7%86%E4%BA%9A%E8%A3%94-%E7%BE%8E4%E4%BA%BA%E8%A2%AB%E8%A2%AD%E6%AF%81%E5%AE%B9/) |
| 中国报 | 2/15/2020 | [【武汉肺炎】疫情助长歧视 美国亚裔遭殴打]("https://www.chinapress.com.my/20200215/%E2%97%A4%E6%AD%A6%E6%B1%89%E8%82%BA%E7%82%8E%E2%97%A2%E7%96%AB%E6%83%85%E5%8A%A9%E9%95%BF%E6%AD%A7%E8%A7%86-%E7%BE%8E%E5%9B%BD%E4%BA%9A%E8%A3%94%E9%81%AD%E6%AE%B4%E6%89%93/) |
| 中国报 | 2/25/2020 | [【武汉肺炎】疫情引发种族歧视袭击 意国华女被两人野蛮殴打]("https://www.chinapress.com.my/20200225/%E2%97%A4%E6%AD%A6%E6%B1%89%E8%82%BA%E7%82%8E%E2%97%A2-%E7%96%AB%E6%83%85%E5%BC%95%E5%8F%91%E7%A7%8D%E6%97%8F%E6%AD%A7%E8%A7%86%E8%A2%AD%E5%87%BB-%E6%84%8F%E5%9B%BD%E5%8D%8E%E5%A5%B3%E8%A2%AB%E4%B8%A4/) |
| 中国报 | 3/3/2020 | [【武汉肺炎】疑因疫情受种族歧视 新国留学生伦敦被痛殴]("https://www.chinapress.com.my/20200303/%E2%97%A4%E6%AD%A6%E6%B1%89%E8%82%BA%E7%82%8E%E2%97%A2-%E5%8F%88%E6%98%AF%E6%AD%A7%E8%A7%86%EF%BC%81-%E7%8B%AE%E5%9F%8E%E5%AD%A6%E7%94%9F-%E4%BC%A6%E6%95%A6%E8%A2%AB%E6%89%93-%E9%87%8D%E4%BC%A4/) |
| 中国报 | 3/25/2020 | [新冠不是中国肺炎，陈冠希发声斥责歧视]("https://www.chinapress.com.my/20200325/%E6%96%B0%E5%86%A0%E4%B8%8D%E6%98%AF%E4%B8%AD%E5%9B%BD%E8%82%BA%E7%82%8E-%E9%99%88%E5%86%A0%E5%B8%8C%E5%8F%91%E5%A3%B0%E6%96%A5%E6%AD%A7%E8%A7%86/) |
| 中国报 | 3/6/2020 | [反华情绪愈趋严重 翁虹吁国际放下歧视]("https://www.chinapress.com.my/20200306/%E5%8F%8D%E5%8D%8E%E6%83%85%E7%BB%AA%E6%84%88%E8%B6%8B%E4%B8%A5%E9%87%8D%E3%80%80%E7%BF%81%E8%99%B9%E5%90%81%E5%9B%BD%E9%99%85%E6%94%BE%E4%B8%8B%E6%AD%A7%E8%A7%86/) |
| 中国报 | 4/3/2020 | [【搜电影】首部新冠肺炎电影登场 中国女子惨遭老外歧视]("https://www.chinapress.com.my/20200403/%E3%80%90%E6%90%9C%E7%94%B5%E5%BD%B1%E3%80%91%E9%A6%96%E9%83%A8%E6%96%B0%E5%86%A0%E8%82%BA%E7%82%8E%E7%94%B5%E5%BD%B1%E7%99%BB%E5%9C%BA-%E4%B8%AD%E5%9B%BD%E5%A5%B3%E5%AD%90%E6%83%A8%E9%81%AD%E8%80%81/) |
| 中国报 | 3/18/2020 | [称新冠肺炎”中国病毒“陈法拉轰特朗普种族歧视]("https://www.chinapress.com.my/20200318/%E7%A7%B0%E6%96%B0%E5%86%A0%E8%82%BA%E7%82%8E%E4%B8%AD%E5%9B%BD%E7%97%85%E6%AF%92-%E9%99%88%E6%B3%95%E6%8B%89%E8%BD%B0%E7%89%B9%E6%9C%97%E6%99%AE%E7%A7%8D%E6%97%8F%E6%AD%A7%E8%A7%86/) |
| 中国报 | 1/23/2020 | [【武汉肺炎】”不需歧视中国游客“旅游部长：顾好健康即可]("https://www.chinapress.com.my/20200123/%E2%97%A4%E6%AD%A6%E6%B1%89%E8%82%BA%E7%82%8E%E2%97%A2-%E4%B8%8D%E9%9C%80%E6%AD%A7%E8%A7%86%E4%B8%AD%E5%9B%BD%E6%B8%B8%E5%AE%A2-%E6%97%85%E6%B8%B8%E9%83%A8%E9%95%BF%EF%BC%9A%E9%A1%BE/) |
| 中国报 | 3/26/2020 | [刘彦运：新冠病毒不会”种族歧视“]("https://www.chinapress.com.my/20200326/%E5%88%98%E5%BD%A6%E8%BF%90%EF%BC%9A%E6%96%B0%E5%86%A0%E7%97%85%E6%AF%92%E4%B8%8D%E4%BC%9A%E7%A7%8D%E6%97%8F%E6%AD%A7%E8%A7%86/) |
| 中国报 | 2/28/2020 | [武汉肺炎 拒绝中国人入住柬埔寨酒店被查]("https://www.chinapress.com.my/20200228/%E6%AD%A6%E6%B1%89%E8%82%BA%E7%82%8E-%E6%8B%92%E7%BB%9D%E4%B8%AD%E5%9B%BD%E4%BA%BA%E5%85%A5%E4%BD%8F-%E6%9F%AC%E5%9F%94%E5%AF%A8%E9%85%92%E5%BA%97%E8%A2%AB%E6%9F%A5/) |
| 中国报 | 2/18/2020 | [武汉肺炎 拒绝中国人入住柬埔寨酒店被查]("https://www.chinapress.com.my/20200218/%E2%97%A4%E6%AD%A6%E6%B1%89%E8%82%BA%E7%82%8E%E2%97%A2-%E6%B3%B0%E5%9B%BD%E4%BA%BA%E5%A4%AA%E5%83%8F%E5%8D%8E%E4%BA%BA%E3%80%80%E8%A2%AB%E9%AA%82%E7%97%85%E6%AF%92%E6%8A%A2%E8%80%B3%E6%9C%BA/) |
| 中国报 | 2/2/2020 | [加拿大总理特鲁多 华人社区拜年 强调反对歧视]("https://www.chinapress.com.my/20200202/%E5%8A%A0%E6%8B%BF%E5%A4%A7%E6%80%BB%E7%90%86%E7%89%B9%E9%B2%81%E5%A4%9A-%E5%8D%8E%E4%BA%BA%E7%A4%BE%E5%8C%BA%E6%8B%9C%E5%B9%B4-%E5%BC%BA%E8%B0%83%E5%8F%8D%E5%AF%B9%E6%AD%A7%E8%A7%86/) |
| 中国报 | 1/31/2020 | [【武汉肺炎】疫情迅速扩散引恐慌 纽时：全球反中情绪蔓延]("https://www.chinapress.com.my/20200131/%E2%97%A4%E6%AD%A6%E6%B1%89%E8%82%BA%E7%82%8E%E2%97%A2-%E7%96%AB%E6%83%85%E8%BF%85%E9%80%9F%E6%89%A9%E6%95%A3%E5%BC%95%E6%81%90%E6%85%8C-%E7%BA%BD%E6%97%B6%EF%BC%9A%E5%85%A8%E7%90%83%E5%8F%8D/) |
| 中国报 | 3/5/2020 | [对种族歧视感无言 MC Jin吁亚裔人士小心]("https://www.chinapress.com.my/20200305/%E5%AF%B9%E7%A7%8D%E6%97%8F%E6%AD%A7%E8%A7%86%E6%84%9F%E6%97%A0%E8%A8%80-mc-jin%E5%90%81%E4%BA%9A%E8%A3%94%E4%BA%BA%E5%A3%AB%E5%B0%8F%E5%BF%83/) |
| 中国报 | 3/24/2020 | [美国华人屡屡被殴 开始买枪自组武装]("https://www.chinapress.com.my/20200324/%E7%BE%8E%E5%9B%BD%E5%8D%8E%E4%BA%BA%E5%B1%A1%E5%B1%A1%E8%A2%AB%E6%AE%B4-%E5%BC%80%E5%A7%8B%E4%B9%B0%E6%9E%AA%E8%87%AA%E7%BB%84%E6%AD%A6%E8%A3%85/) |
| 中国报 | 2/19/2020 | [【武汉肺炎】专家：疫情或引起”恐华症“]("https://www.chinapress.com.my/20200219/%E2%97%A4%E6%AD%A6%E6%B1%89%E8%82%BA%E7%82%8E%E2%97%A2%E4%B8%93%E5%AE%B6%EF%BC%9A%E7%96%AB%E6%83%85%E6%88%96%E5%BC%95%E8%B5%B7%E6%81%90%E5%8D%8E%E7%97%87/) |
| 中国报 | 2/4/2020 | [甄子曰专栏：一起来歧视]("https://www.chinapress.com.my/20200204/%E7%94%84%E5%AD%90%E6%9B%B0%E4%B8%93%E6%A0%8F%EF%BC%9A%E4%B8%80%E8%B5%B7%E6%9D%A5%E6%AD%A7%E8%A7%86/) |
| 中国报 | 3/22/2020 | [【全球大流行】疫情来自中国？并无任何证据！]("https://www.chinapress.com.my/20200322/%E2%97%A4%E5%85%A8%E7%90%83%E5%A4%A7%E6%B5%81%E8%A1%8C%E2%97%A2-%E7%96%AB%E6%83%85%E6%9D%A5%E8%87%AA%E4%B8%AD%E5%9B%BD%EF%BC%9F%E5%B9%B6%E6%97%A0%E4%BB%BB%E4%BD%95%E8%AF%81%E6%8D%AE%EF%BC%81/) |
| 中国报 | 1/30/2020 | [潘有文：暂禁中国人入境时抗毒非歧视]("https://www.chinapress.com.my/20200130/%E6%BD%98%E6%9C%89%E6%96%87%EF%BC%9A%E6%9A%82%E7%A6%81%E4%B8%AD%E5%9B%BD%E4%BA%BA%E5%85%A5%E5%A2%83%E6%98%AF%E6%8A%97%E6%AF%92%E9%9D%9E%E6%AD%A7%E8%A7%86/) |
| 中国报 | 4/10/2020 | [【全球大流行】澳女店面大骂店员 亚裔人被当成箭靶]("https://www.chinapress.com.my/20200410/%E2%97%A4%E5%85%A8%E7%90%83%E5%A4%A7%E6%B5%81%E8%A1%8C%E2%97%A2-%E6%BE%B3%E5%A5%B3%E5%BA%97%E9%9D%A2%E5%A4%A7%E9%AA%82%E5%BA%97%E5%91%98-%E4%BA%9A%E8%A3%94%E4%BA%BA%E8%A2%AB%E5%BD%93%E6%88%90%E7%AE%AD/) |
| 中国报 | 3/9/2020 | [不满称武汉肺炎 中国网红爆粗口 钟明轩霸气反击]("https://www.chinapress.com.my/20200309/%E4%B8%8D%E6%BB%A1%E7%A7%B0%E6%AD%A6%E6%B1%89%E8%82%BA%E7%82%8E-%E4%B8%AD%E5%9B%BD%E7%BD%91%E7%BA%A2%E7%88%86%E7%B2%97%E5%8F%A3-%E9%92%9F%E6%98%8E%E8%BD%A9%E9%9C%B8%E6%B0%94%E5%8F%8D%E5%87%BB/) |
| 中国报 | 3/2/2020 | [【武汉肺炎】巫启贤捐赠物资 女儿曝美国排华现象]("https://www.chinapress.com.my/20200302/%E2%97%A4%E6%AD%A6%E6%B1%89%E8%82%BA%E7%82%8E%E2%97%A2-%E5%B7%AB%E5%90%AF%E8%B4%A4%E6%8D%90%E8%B5%A0%E7%89%A9%E8%B5%84-%E5%A5%B3%E5%84%BF%E6%9B%9D%E7%BE%8E%E5%9B%BD%E6%8E%92%E5%8D%8E%E7%8E%B0%E8%B1%A1/) |
| **Canada (Jan.23-Apr.20, 2020)** | | |
| **Newspaper** | **Date** | **News Title** |
| 多伦多新闻网 | 3/9/2020 | [加拿大唐人街等地多座寺庙的雕像被砸坏涂鸦]("http://www.torontonewsnet.com/2020/03/09/9824/) |
| 加拿大和世界报道 | 1/28/2020 | [安省约克区教育局警告近期对冠状病毒疫情的担忧会转向对华人的种族歧视]("https://canadanewsreport.com/2020/01/28/6543/) |
| 加拿大和世界报道 | 2/2/2020 | [加拿大总理特鲁多强调：要避免病毒错误信息传播而引发的歧视]("https://canadanewsreport.com/2020/02/02/6587/) |
| 加拿大国际广播 | 4/8/2020 | [遭遇种族歧视怎么办？请联络我们：人权活动人士Fo Niemi呼吁](https://www.rcinet.ca/zh/2020/04/08/%e9%81%ad%e9%81%87%e7%a7%8d%e6%97%8f%e6%ad%a7%e8%a7%86%e6%80%8e%e4%b9%88%e5%8a%9e%ef%bc%9f%e8%af%b7%e8%81%94%e7%bb%9c%e6%88%91%e4%bb%ac%ef%bc%9a%e4%ba%ba%e6%9d%83%e6%b4%bb%e5%8a%a8%e4%ba%ba%e5%a3%abfo/) |
| 加拿大国际广播 | 3/12/2020 | [新冠病毒时期的种族歧视，活动人士吴婷婷：华裔应该意识到无人可以幸免](https://www.rcinet.ca/zh/2020/03/12/%e6%96%b0%e5%86%a0%e7%97%85%e6%af%92%e6%97%b6%e6%9c%9f%e7%9a%84%e7%a7%8d%e6%97%8f%e6%ad%a7%e8%a7%86%ef%bc%8c%e6%b4%bb%e5%8a%a8%e4%ba%ba%e5%a3%ab%e5%90%b4%e5%a9%b7%e5%a9%b7%ef%bc%9a%e5%8d%8e%e8%a3%94/) |
| 加拿大国际广播 | 3/5/2020 | [专访黎岚辉：新冠病毒引发歧视与仇恨？蒙特利尔连续发生破坏佛教雕塑事件](https://www.rcinet.ca/zh/2020/03/05/%e4%b8%93%e8%ae%bf%e9%bb%8e%e5%b2%9a%e8%be%89%ef%bc%9a%e6%96%b0%e5%86%a0%e7%97%85%e6%af%92%e5%bc%95%e5%8f%91%e6%ad%a7%e8%a7%86%e4%b8%8e%e4%bb%87%e6%81%a8%ef%bc%9f%e8%92%99%e7%89%b9%e5%88%a9%e5%b0%94/) |
| 加拿大国际广播 | 2/3/2020 | [特鲁多：加拿大人不能因肺炎疫情歧视华人](https://www.rcinet.ca/zh/2020/02/03/%e7%89%b9%e9%b2%81%e5%a4%9a%ef%bc%9a%e5%8a%a0%e6%8b%bf%e5%a4%a7%e4%ba%ba%e4%b8%8d%e8%83%bd%e5%9b%a0%e8%82%ba%e7%82%8e%e7%96%ab%e6%83%85%e6%ad%a7%e8%a7%86%e5%8d%8e%e4%ba%ba/) |
| 加拿大国际广播 | 1/30/2020 | [冠状病毒带来的不仅是武汉肺炎，还有种族歧视](https://www.rcinet.ca/zh/2020/01/30/%e5%86%a0%e7%8a%b6%e7%97%85%e6%af%92%e5%b8%a6%e6%9d%a5%e7%9a%84%e4%b8%8d%e4%bb%85%e6%98%af%e6%ad%a6%e6%b1%89%e8%82%ba%e7%82%8e%ef%bc%8c%e8%bf%98%e6%9c%89%e7%a7%8d%e6%97%8f%e6%ad%a7%e8%a7%86/) |
| 加拿大国际广播 | 1/29/2020 | [华人不应因武汉肺炎被歧视和污名化：加拿大华人社团领袖和多伦多官员共同呼吁](https://www.rcinet.ca/zh/2020/01/29/%e5%8d%8e%e4%ba%ba%e4%b8%8d%e5%ba%94%e5%9b%a0%e6%ad%a6%e6%b1%89%e8%82%ba%e7%82%8e%e8%a2%ab%e6%ad%a7%e8%a7%86%e5%92%8c%e6%b1%a1%e5%90%8d%e5%8c%96%ef%bc%9a%e5%8a%a0%e6%8b%bf%e5%a4%a7%e5%8d%8e%e4%ba%ba/) |
| **Angola (Jan. 23-Apr.17, 2020)** | | |
| **Newspaper** | **Date** | **News Title** |
| 安哥拉华人报 | 3/10/2020 | [疫情之下：如何看待“中国人在海外遭歧视”？]("https://mp.weixin.qq.com/s/AnNK32WBqY3s6gP6vrizQA) |
| 安哥拉华人报 | 4/15/2020 | [疫情下的安哥拉，中国人是最安全的群体]("https://mp.weixin.qq.com/s?src=11&timestamp=1617511101&ver=2987&signature=gaUygj-aYdhnRuNzu3f7cgkXkUeVALqsV499YkFN3ssQcgwc25LccQNoblF2Dqbu1vh7Y5ta*sMrqoXiXcrEtKIMyC5EBZhwaioN5v*h1*ynouRqKBLRtBAURaNXWOR8&new=1) |
| 安哥拉华人报 | 3/16/2020 | [侨胞如何应对海外疫情，收好这份防护与求助指南]("https://mp.weixin.qq.com/s?src=11&timestamp=1617511133&ver=2987&signature=gaUygj-aYdhnRuNzu3f7cgkXkUeVALqsV499YkFN3sv7R0QjBOPgiw3SjhMGNb1bbqckOiMZJtgh0u4Wr22HYanwXA8Sboabdmpi5XJrLPDoaxNUl4cFmFdJ9DLD4-8T&new=1) |
| 安哥拉华人报 | 3/5/2020 | [疫情之下，海外中国公民遇到的这些问题，官方回应来了！]("https://mp.weixin.qq.com/s?src=11&timestamp=1617511181&ver=2987&signature=gaUygj-aYdhnRuNzu3f7cgkXkUeVALqsV499YkFN3sv6aDle1hfKl-GX9cqgqEmjHpGclOfzdw9i48YXkwPW3tsVmJvbsyzBPZJpExeFX6Hg2MG2lyTG4Uk*nJ9lDbmF&new=1) |
| **Belgium (Jan.23-Apr.18, 2020)** | | |
| **Newspaper** | **Date** | **News Title** |
| 华商时报 | 3/10/2020 | [疫情之下，如何看待“中国人在海外遭歧视”？]("http://huashang.be/5039/) |
| 华商时报 | 2/3/2020 | [女儿在校遭受歧视 中山学校临时停课——华裔议员接受比媒体专访]("http://huashang.be/4705/) |
| 华商时报 | 2/25/2020 | [阿尔斯特狂欢节出现辱华一幕 华人社团向市长发抗议信]("http://huashang.be/4946/) |
| 欧华商报 | 2/20/2020 | 中国驻欧盟使团团长张明：疫情对中国经济和中欧交流影响有限 |
| 欧华商报 | 3/5/2020 | [气愤！华人在布鲁塞尔受到骚扰！](https://weixin.sogou.com/link?url=dn9a_-gY295K0Rci_xozVXfdMkSQTLW6cwJThYulHEtVjXrGTiVgSyYKEvoYXj7uSSko5d8xX_kMNh4svKnPglqXa8Fplpd9jJHDzjXep-LtXvsmHUDLt0mMuUFC63bJulmsfB9RuoH6Hxu4MSSdX5qLbZoWE3dNbiHJJMKjG15x2JbZA3pWNA2HCST8ORrkPiRZdqF4bQxXz1HW_mJMNrR3_txfvf) |
| 欧华商报 | 3/12/2020 | [驻比使馆心系侨胞，高度重视在比华人的生命健康和安全](https://weixin.sogou.com/link?url=dn9a_-gY295K0Rci_xozVXfdMkSQTLW6cwJThYulHEtVjXrGTiVgSyYKEvoYXj7uzeEMamuLehcMNh4svKnPglqXa8Fplpd9jJHDzjXep-LtXvsmHUDLt0mMuUFC63bJulmsfB9RuoH6Hxu4MSSdXykw9qUSHAcZFmYLasyCftw_KYF7qmlvcqnQFZkvp8Xb1sKURCXUlT9H1G75gVNnRnLkb2Kub7) |
| **Portugal (Jan.23-Apr.17, 2020)** | | |
| **Newspaper** | **Date** | **News Title** |
| 葡华报 | 4/17/2020 | [葡萄牙《快报》刊发中国驻葡萄牙大使蔡润的署名文章《同舟共济 共克时艰》]("http://www.puhuabao.pt/2020/04/17/%e8%91%a1%e8%90%84%e7%89%99%e3%80%8a%e5%bf%ab%e6%8a%a5%e3%80%8b%e5%88%8a%e5%8f%91%e4%b8%ad%e5%9b%bd%e9%a9%bb%e8%91%a1%e8%90%84%e7%89%99%e5%a4%a7%e4%bd%bf%e8%94%a1%e6%b6%a6%e7%9a%84%e7%bd%b2%e5%90%8d/) |
| 葡华报 | 3/25/2020 | [葡萄牙《新闻日报》刊发中国驻葡萄牙大使蔡润的署名文章《患难见真情——中葡携手合作抗击疫情》]("http://www.puhuabao.pt/2020/03/25/%e8%91%a1%e8%90%84%e7%89%99%e3%80%8a%e6%96%b0%e9%97%bb%e6%97%a5%e6%8a%a5%e3%80%8b%e5%88%8a%e5%8f%91%e4%b8%ad%e5%9b%bd%e9%a9%bb%e8%91%a1%e8%90%84%e7%89%99%e5%a4%a7%e4%bd%bf%e8%94%a1%e6%b6%a6%e7%9a%84/) |
| 葡华报 | 2/7/2020 | [RFM电台行政总裁就“中国新冠病毒肺炎疫情”不当言论向在葡华人社群致歉]("http://www.puhuabao.pt/2020/02/07/rfm%e7%94%b5%e5%8f%b0%e8%a1%8c%e6%94%bf%e6%80%bb%e8%a3%81%e5%b0%b1%e4%b8%ad%e5%9b%bd%e6%96%b0%e5%86%a0%e7%97%85%e6%af%92%e8%82%ba%e7%82%8e%e7%96%ab%e6%83%85%e4%b8%8d%e5%bd%93/) |
| 葡新报 | --- | --- |
| 華人PT | --- | --- |
| **India (Jan.23-Apr.18, 2020)** | | |
| **Newspaper** | **Date** | **News Title** |
| 印度华人网 | --- | --- |
| 印度中文网 | --- | --- |
| **Greece (Jan.23-Apr.23, 2020)** | | |
| **Newspaper** | **Date** | **News Title** |
| 中希时报/希中网 | 2/3/2020 | “我是中国人，但我不是病毒！”疫情之下，海外华人遭遇了什么 |
| 中希时报/希中网 | 2/6/2020 | [多国出现歧视华人言论，希腊学者硬核发文：“我们都是中国人！”](https://weixin.sogou.com/link?url=dn9a_-gY295K0Rci_xozVXfdMkSQTLW6cwJThYulHEtVjXrGTiVgSyYKEvoYXj7uiTMEgnEO5mkMNh4svKnPglqXa8Fplpd9u9lzBBlCHhvuD0Qdt9bds6qM9J51jh-RKeryqia6IRpqrAYTxqTtc3wR_pMnikSGrAv87FY9B5vLry37s_Ehy90JOuzkw92YaP9MCbzA5gK7HSjD0CuseZ3jFSypYS) |
| 中希时报/希中网 | 2/9/2020 | [特殊时刻敢歧视中国人？这群希腊人可不答应！](https://weixin.sogou.com/link?url=dn9a_-gY295K0Rci_xozVXfdMkSQTLW6cwJThYulHEtVjXrGTiVgSyYKEvoYXj7utMd8KBQebSoMNh4svKnPglqXa8Fplpd9u9lzBBlCHhvuD0Qdt9bds6qM9J51jh-RKeryqia6IRpqrAYTxqTtcxlqUyRTO2vg3WxK0BC3iay9PuPW0TqznQMSgiSK0gl1TcLih6APmzuSEUMZPuHC81SkAjJCcG) |
| 中希时报/希中网 | 2/19/2020 | [面对疫情，希腊部长力挺中国：两国关系空前友好，我们坚定站在一起！](https://weixin.sogou.com/link?url=dn9a_-gY295K0Rci_xozVXfdMkSQTLW6cwJThYulHEtVjXrGTiVgSyYKEvoYXj7u9CePlNp2DJQMNh4svKnPglqXa8Fplpd9u9lzBBlCHhvuD0Qdt9bds6qM9J51jh-RKeryqia6IRpqrAYTxqTtc-QKus23kFw0C63V1R4ooah4CkLrI_DXERsbFqX1M1TyfLFmvPN07rJjccIRfhbeEnHLudWm63) |
| 中希时报/希中网 | 3/20/2020 | [疫情日趋严重，希腊国际学校作何反应？](https://weixin.sogou.com/link?url=dn9a_-gY295K0Rci_xozVXfdMkSQTLW6cwJThYulHEtVjXrGTiVgSyYKEvoYXj7uKOqhApN8OA8MNh4svKnPglqXa8Fplpd9u9lzBBlCHhvuD0Qdt9bds6qM9J51jh-RKeryqia6IRpqrAYTxqTtc1bqwYgNwEq1iJwsVzDunPrkPun1CgjW8R1oZE7bTL1C3SyUthBf9Q1hSjSgs1LUvm0VghQM4k) |
| 中希时报/希中网 | 3/11/2020 | [除了意大利，西班牙也快撑不住了！疫情之下，华人何去何从？](https://weixin.sogou.com/link?url=dn9a_-gY295K0Rci_xozVXfdMkSQTLW6cwJThYulHEtVjXrGTiVgSyYKEvoYXj7ujhnJOy_Cpq0MNh4svKnPglqXa8Fplpd9u9lzBBlCHhvuD0Qdt9bds6qM9J51jh-RKeryqia6IRpqrAYTxqTtczgihuNGky-B1OWLbM4o5PwR8_tIE5pvagzk-KThDW-wuZpKI4t-EteEHrmTuHC1mw-7b7KEUY) |
| 中希时报/希中网 | 3/25/2020 | 中国驻希腊大使章启月：挚友之情，感同身受！ |
| 希华时讯 | 1/21/2020 | [国外歧视华人事件增多？比疫情更可怕的是人心](https://weixin.sogou.com/link?url=dn9a_-gY295K0Rci_xozVXfdMkSQTLW6cwJThYulHEtVjXrGTiVgSyYKEvoYXj7ux795qFO9r6oMNh4svKnPglqXa8Fplpd9LFXrOpY2QrAp94KXZQjSaGn8ad4eN6Bmld-lE-WxgkUkHNU87pcyHEchEgImsEyQBdOjtbPZAiN2xihTYi2v6CVN85OdxaMnkoRilnro9I2tsI5wzX9X6qd4rhLc3p) |
| 希华时讯 | 2/8/2020 | [雅典孔院希腊学生录制视频为中国加油](https://weixin.sogou.com/link?url=dn9a_-gY295K0Rci_xozVXfdMkSQTLW6cwJThYulHEtVjXrGTiVgSyYKEvoYXj7u0aXQY6Z2lQQMNh4svKnPglqXa8Fplpd9LFXrOpY2QrAp94KXZQjSaGn8ad4eN6Bmld-lE-WxgkUkHNU87pcyHGjIlurWN7r87IvY6b0QLk2mvX26dC-FQJ2RiV9G0atCd25u2DobeMFVi9-mzVOzM2F5mGOuzT) |
| 希华时讯 | 2/9/2020 | [疫情阴影，海外中餐馆生意受到重创！他们站出来力挺中餐](https://weixin.sogou.com/link?url=dn9a_-gY295K0Rci_xozVXfdMkSQTLW6cwJThYulHEtVjXrGTiVgSyYKEvoYXj7u2TqGZ-TsGDEMNh4svKnPglqXa8Fplpd9LFXrOpY2QrAp94KXZQjSaGn8ad4eN6Bmld-lE-WxgkUkHNU87pcyHAIrOJiQB3tNej-WSvZZGub3Gd0NDMhnwvBtKUTUQoqLv-3UHwlS2uxl02g0MnIZzf54EmW9BK) |
| 希华时讯 | 2/20/2020 | [欧洲旅游委员会：尽量不取消中国旅游者的订单](https://weixin.sogou.com/link?url=dn9a_-gY295K0Rci_xozVXfdMkSQTLW6cwJThYulHEtVjXrGTiVgSyYKEvoYXj7ulZT6N95dO5cMNh4svKnPglqXa8Fplpd9LFXrOpY2QrAp94KXZQjSaGn8ad4eN6Bmld-lE-WxgkUkHNU87pcyHEalTM8DTDnJcxq36mQTpcPd16iTYBvH6QfPmsOJEE4f2XZE3Ok4JYq-g8zeawwZNxkqosgXsg) |
| **Sweden (Jan.23-Apr.23, 2020)** | | |
| **Newspaper** | **Date** | **News Title** |
| 北欧时报 | 3/12/2020 | 中国金桥抵达瑞典，东方巨龙享誉北欧 |
| 北欧华人报 | --- | --- |
| 北欧国际新闻 | --- | --- |
| **Saudi Arabia (Jan.23-Apr.16, 2020)** | | |
| **Newspaper** | **Date** | **News Title** |
| 沙特华人网 | --- | --- |
| **The Netherlands (Jan.23-Apr.16, 2020)** | | |
| **Newspaper** | **Date** | **News Title** |
| 中荷商报 | 2/4/2020 | [种族歧视再度肆虐！荷兰医疗专家公然发表这种言论，新冠成了种族歧视者的武器](https://weixin.sogou.com/link?url=dn9a_-gY295K0Rci_xozVXfdMkSQTLW6cwJThYulHEtVjXrGTiVgSyYKEvoYXj7uPtWJvnJoziUMNh4svKnPglqXa8Fplpd97Je-vd-2LlFn-YtfR4Mpk-FD5LZUMo4UOLciBA64oH5NUJ2MsENwYCLzXbvCVpsA6FHRYorWtdXaQxeDtmilLrDUUmGYDJ5TRNEMNsBd8lfQAsLdE1V1WU_hNwooAe) |
| 中荷商报 | 2/5/2020 | [库肯霍夫要被封？荷兰部长发话了：所有人！种族歧视不可接受](https://weixin.sogou.com/link?url=dn9a_-gY295K0Rci_xozVXfdMkSQTLW6cwJThYulHEtVjXrGTiVgSyYKEvoYXj7uIWaKvLNEwRsMNh4svKnPglqXa8Fplpd97Je-vd-2LlFn-YtfR4Mpk-FD5LZUMo4UOLciBA64oH5NUJ2MsENwYOCuAFO3IBxW2ct32M1ZoSJ_89M1-rliX7m2v6GkjvgXOKZVGx0dOGKU8lROJLmwkmUS6bX-Lx) |
| 中荷商报 | 2/10/2020 | [荷兰政府公开阐明反对种族歧视的坚定立场](https://weixin.sogou.com/link?url=dn9a_-gY295K0Rci_xozVXfdMkSQTLW6cwJThYulHEtVjXrGTiVgSyYKEvoYXj7uw98zrAq7jzEMNh4svKnPglqXa8Fplpd97Je-vd-2LlFn-YtfR4Mpk-FD5LZUMo4UOLciBA64oH5NUJ2MsENwYLPnbI1N3SjcmYq2LgpmZbnucqOAQ10njhlCcbcvDKIiay-Leo4dWyhwAXFGuoqXjx38TSOvTz) |
| 中荷商报 | 2/13/2020 | [疫情之下，为何中国人歧视起了中国人？一篇文章看懂](https://weixin.sogou.com/link?url=dn9a_-gY295K0Rci_xozVXfdMkSQTLW6cwJThYulHEtVjXrGTiVgSyYKEvoYXj7uCES6_Lp5OZsMNh4svKnPglqXa8Fplpd97Je-vd-2LlFn-YtfR4Mpk-FD5LZUMo4UOLciBA64oH5NUJ2MsENwYN_SjnBw4CnLo0ymYjMIrppR9NVva2ps_E11ZFPAX6EIMLPHaVLfcHe5efF2GY4LObnxUsC1AJ) |
| 中荷商报 | 2/16/2020 | [#ikchinees运动已正式启动！树立积极而多元的华人与中餐形象，反歧视，撑华人](https://weixin.sogou.com/link?url=dn9a_-gY295K0Rci_xozVXfdMkSQTLW6cwJThYulHEtVjXrGTiVgSyYKEvoYXj7uRvewyK9kIwwMNh4svKnPglqXa8Fplpd97Je-vd-2LlFn-YtfR4Mpk-FD5LZUMo4UOLciBA64oH5NUJ2MsENwYKXoaTUHcKIgGx0dld_aFAFjp9Thp3TI9IS4xPI1FlxIJa4Ds0bSUvCN1In6Stvpx3Cjst0zEB) |
| 中荷商报 | 2/24/2020 | 24岁蒂尔堡女生劝阻“新冠辱华歌” 被荷兰男子仇恨袭击 |
| 华侨新天地 | 2/4/2020 | 徐宏大使就新型冠状病毒感染肺炎疫情接受荷兰《人民报》记者采访 |
| 华侨新天地 | 2/5/2020 | [比病毒更可怕的是这些！荷兰华人对歧视这样做](https://weixin.sogou.com/link?url=dn9a_-gY295K0Rci_xozVXfdMkSQTLW6cwJThYulHEtVjXrGTiVgSyYKEvoYXj7uFQkIn2AIEeUMNh4svKnPglqXa8Fplpd9aMqCX_Fc1K_1LnNcP2FKkOG1B3ax0j5yepJX5DIZKE5VMO-yJzLDKsT7fywTquyWoHlGaha2aHukwFdtCT6UtUL9ekWRtG-C4KBhTv0MrdoNUJ7lSZ1AoWh6aKEymh) |
| 华侨新天地 | 2/9/2020 | [驻荷兰使馆发言人：种族歧视在荷兰不得人心](https://weixin.sogou.com/link?url=dn9a_-gY295K0Rci_xozVXfdMkSQTLW6cwJThYulHEtVjXrGTiVgSyYKEvoYXj7uPk_YkT81FvcMNh4svKnPglqXa8Fplpd9aMqCX_Fc1K_1LnNcP2FKkOG1B3ax0j5yepJX5DIZKE5VMO-yJzLDKsT7fywTquyWoHlGaha2aHukwFdtCT6UtW0OzsU5zQpMBEtDScOqvX78BZQhd8rctGCZkLvtcA) |
| 华侨新天地 | 2/10/2020 | [几万中国人签名请愿荷兰政府反歧视，轰动荷媒体！](https://weixin.sogou.com/link?url=dn9a_-gY295K0Rci_xozVXfdMkSQTLW6cwJThYulHEtVjXrGTiVgSyYKEvoYXj7upCCoD5ixKcYMNh4svKnPglqXa8Fplpd9aMqCX_Fc1K_1LnNcP2FKkOG1B3ax0j5yepJX5DIZKE5VMO-yJzLDKgZQg1pP_oiJpJipBJ-zBZ7zTV25obxmGG8oIhmf52bBlLMl6A5AfCEgQZn36Cbz9s-1N9OvXY) |
| 华侨新天地 | 2/10/2020 | [荷兰辱华事件升级！中国学生公寓内国旗被撕，电梯被泼粪便](https://weixin.sogou.com/link?url=dn9a_-gY295K0Rci_xozVXfdMkSQTLW6cwJThYulHEtVjXrGTiVgSyYKEvoYXj7u3JTtKPFTKBEMNh4svKnPglqXa8Fplpd9aMqCX_Fc1K_1LnNcP2FKkOG1B3ax0j5yepJX5DIZKE5VMO-yJzLDKvqfs_0_zr1Jdmi7vwYY2woQkf5b_GolGAmxgwsT6RyBjxItFp22BaOCnHV2uGhncE1yCmxejr) |
| 华侨新天地 | 2/11/2020 | [辱华升级为暴力！两华裔被群殴，荷媒报道为抢劫案！](https://weixin.sogou.com/link?url=dn9a_-gY295K0Rci_xozVXfdMkSQTLW6cwJThYulHEtVjXrGTiVgSyYKEvoYXj7u8E2S0hZDHU8MNh4svKnPglqXa8Fplpd9aMqCX_Fc1K_1LnNcP2FKkOG1B3ax0j5yepJX5DIZKE5VMO-yJzLDKgIP-inRmylS2_GFPetj-LS9QqKDwGDx2zx7LGhwrmWgwg4K0wDdcK3YHd0RV-S1HDK9jER3B8) |
| 华侨新天地 | 2/11/2020 | [荷兰电视直播+辱华DJ道歉+五万签名，但辱华事件没完](https://weixin.sogou.com/link?url=dn9a_-gY295K0Rci_xozVXfdMkSQTLW6cwJThYulHEtVjXrGTiVgSyYKEvoYXj7uiVfwsBgjoAEMNh4svKnPglqXa8Fplpd9aMqCX_Fc1K_1LnNcP2FKkOG1B3ax0j5yepJX5DIZKE5VMO-yJzLDKm0JaKty7PdyCzEZ5O5yjcp-4FHT7pIOypInl4kiSW8C3x1w86tcqTIhiz-S0Fgd3Gx-uumyi-) |
| 华侨新天地 | 2/14/2020 | [5万请愿签名后，荷兰惊现狂欢节“病毒防护服”！反歧视路漫漫](https://weixin.sogou.com/link?url=dn9a_-gY295K0Rci_xozVXfdMkSQTLW6cwJThYulHEtVjXrGTiVgSyYKEvoYXj7uJI2O6-HS3c0MNh4svKnPglqXa8Fplpd9aMqCX_Fc1K_1LnNcP2FKkOG1B3ax0j5yepJX5DIZKE5VMO-yJzLDKiheYDFKjeU0CvlrZvJ-uffuVGOlJqVivfm-fVXS1JY7ok36N0bwDdyfPlPXJdimW1yybw9goW) |
| 华侨新天地 | 2/17/2020 | [中国人在家自行隔离，荷兰老人却突然找上门？居然是为了……](https://weixin.sogou.com/link?url=dn9a_-gY295K0Rci_xozVXfdMkSQTLW6cwJThYulHEtVjXrGTiVgSyYKEvoYXj7usmwXYB7e1YEMNh4svKnPglqXa8Fplpd9aMqCX_Fc1K_1LnNcP2FKkOG1B3ax0j5yepJX5DIZKE5VMO-yJzLDKjdgtL58YP3eb4DFsEb8ePS_2h7Gtt7sfDNAkcul0jRMH-wOqIx9p1MXpR8qgNcbWkvYpUPBt1) |
| 华侨新天地 | 2/18/2020 | [华人在荷兰戴口罩上街引来检疫员还上电视？专家唱反调](https://weixin.sogou.com/link?url=dn9a_-gY295K0Rci_xozVXfdMkSQTLW6cwJThYulHEtVjXrGTiVgSyYKEvoYXj7usmwXYB7e1YEMNh4svKnPglqXa8Fplpd9aMqCX_Fc1K_1LnNcP2FKkOG1B3ax0j5yepJX5DIZKE5VMO-yJzLDKjdgtL58YP3eb4DFsEb8ePS_2h7Gtt7sfDNAkcul0jRMH-wOqIx9p1MXpR8qgNcbWkvYpUPBt1) |
| 华侨新天地 | 2/19/2020 | [荷兰邮轮被确诊感染，GGD才急寻回荷的乘客！华人却自行隔离](https://weixin.sogou.com/link?url=dn9a_-gY295K0Rci_xozVXfdMkSQTLW6cwJThYulHEtVjXrGTiVgSyYKEvoYXj7u2rCD_lmONyUMNh4svKnPglqXa8Fplpd9aMqCX_Fc1K_1LnNcP2FKkOG1B3ax0j5yepJX5DIZKE5VMO-yJzLDKifgLG6ql4iNnL1kDdTPNLiq_zbGi5WC7Rm8pST20rsFfTtYfuWym74QS4lBfyTIhSeLr8nFj5) |
| 华侨新天地 | 3/1/2020 | [徐宏大使在新冠肺炎疫情吹风会上的问答实录](https://weixin.sogou.com/link?url=dn9a_-gY295K0Rci_xozVXfdMkSQTLW6cwJThYulHEtVjXrGTiVgSyYKEvoYXj7uu01jV2lYhLMMNh4svKnPglqXa8Fplpd9aMqCX_Fc1K_1LnNcP2FKkOG1B3ax0j5yepJX5DIZKE5VMO-yJzLDKs6ANXd3oCO26VM32eATCq5JXdaaE1jNQayBdkKMsMR3GUaiZY11WMZ10ZekTqFqeBiyt3SZI4) |
| 华侨新天地 | 3/26/2020 | 海外侨胞抗疫情录 查尔斯王储都感染了 疫情下的英国华侨华人、留学生怎么样了 |
| 华侨新天地 | 4/11/2020 | 在疫情中反复遭受煎熬的欧洲华人：老板让我摘下口罩！ |
| 华侨新天地 | 4/16/2020 | 傲慢与偏见才是阻挡全球抗疫斗争的最大原罪 致荷兰《人民报》编辑部的公开信 |
| **Switzerland (Jan.23-Apr.16, 2020)** | | |
| **Newspaper** | **Date** | **News Title** |
| 欧亚时报 | --- | --- |
| **Mexico (Jan.23-Apr.16, 2020)** | | |
| **Newspaper** | **Date** | **News Title** |
| 华文时报 | 2/5/2020 | [分享与判断：不在新冠疫区究竟要不要戴口罩](https://weixin.sogou.com/link?url=dn9a_-gY295K0Rci_xozVXfdMkSQTLW6cwJThYulHEtVjXrGTiVgSyYKEvoYXj7uHeOJ2zFxMuoMNh4svKnPglqXa8Fplpd9RVkirRS4ZFTnzyKGsb-4NS1htMfMZ9Ko18bpMDag9bUu5qSJaZJopK0Hh7-B9V_0TeHU3FqmLHAJ6shGqj3ZB9jc8LK63mdzz4ATujvXTA5VgNoIcBpSeCCteAPuj6) |
| 华文时报 | 2/6/2020 | [认时务：相比新冠病毒，种族主义和排外主义才是真正的病毒](https://weixin.sogou.com/link?url=dn9a_-gY295K0Rci_xozVXfdMkSQTLW6cwJThYulHEtVjXrGTiVgSyYKEvoYXj7uWn4xa-m4F6AMNh4svKnPglqXa8Fplpd9RVkirRS4ZFTnzyKGsb-4NS1htMfMZ9Ko18bpMDag9bUu5qSJaZJopERU-baihGaoR0l5sPxzxvVPSoPww_ij5Rq3Otb_CUPj6bzYE1zUkogzdAEQNfV6wWT20QEzOw) |
| 华文时报 | 3/24/2020 | [中国驻墨西哥使馆发言人就墨演员Carmen Salinas歧视性言论发表声明](https://weixin.sogou.com/link?url=dn9a_-gY295K0Rci_xozVXfdMkSQTLW6cwJThYulHEtVjXrGTiVgSyYKEvoYXj7uk_lMJlyPYZ8MNh4svKnPglqXa8Fplpd9RVkirRS4ZFTnzyKGsb-4NS1htMfMZ9Ko18bpMDag9bUu5qSJaZJopMifQxzcnsa3P4KBno__qAl2mOZlhje9Hoi4leA5kErvbVC42-3cv8Yemu-dwLRwL1JoV5m96Q) |
| 华文时报 | 4/10/2020 | [驻墨西哥使领馆举行旅墨侨团新冠肺炎疫情防控工作专题视频会](https://weixin.sogou.com/link?url=dn9a_-gY295K0Rci_xozVXfdMkSQTLW6cwJThYulHEtVjXrGTiVgSyYKEvoYXj7uTSOKXfRcN0gMNh4svKnPglqXa8Fplpd9RVkirRS4ZFTnzyKGsb-4NS1htMfMZ9Ko18bpMDag9bUu5qSJaZJopPlL8ndcetnq-s7_7syi6AujnWmj2rGByq7pATG180uI4FREU9PQvVNBwxuXY1nhuhZKiGAO-z) |
| 墨西哥华人网 | --- | --- |
| 墨西哥新闻网 | --- | --- |
| **South Africa (Jan.23-Apr.17, 2020)** | | |
| **Newspaper** | **Date** | **News Title** |
| 非洲之声 | 2/2/2020 | 《中国是真正的“亚洲病夫”》种族歧视色彩耸人听闻 |
| 非洲之声 | 2/20/2020 | 《联合报》发表题为《瘟疫引发的丑恶歧视》 |
| 非洲之声 | 3/7/2020 | 坚决回击歧视中国公民 制造恐慌情绪 |
| 非洲之声 | 3/20/2020 | 刻意使用带有歧视性意涵的字眼 |
| 非洲之声 | 3/21/2020 | 赤裸裸的种族歧视行为 用以是转移美国国内对其领导的政府应对疫情不力 |
| 非洲之声 | 3/29/2020 | 美国政客煽动歧视 造谣生事 |
| 非洲侨报 | --- | --- |
| 中非新闻 | --- | --- |
| **Vietnam (Jan.23-Apr.18, 2020)** | | |
| **Newspaper** | **Date** | **News Title** |
| --- | --- | --- |
| **Kenya (Jan.23-Apr.18, 2020)** | | |
| **Newspaper** | **Date** | **News Title** |
| 非洲之声 | 2/2/2020 | 《中国是真正的“亚洲病夫”》种族歧视色彩耸人听闻 |
| 非洲之声 | 2/20/2020 | 《联合报》发表题为《瘟疫引发的丑恶歧视》 |
| 非洲之声 | 3/7/2020 | 坚决回击歧视中国公民 制造恐慌情绪 |
| 非洲之声 | 3/20/2020 | 刻意使用带有歧视性意涵的字眼 |
| 非洲之声 | 3/21/2020 | 赤裸裸的种族歧视行为 用以是转移美国国内对其领导的政府应对疫情不力 |
| 非洲之声 | 3/29/2020 | 美国政客煽动歧视 造谣生事 |
| 非洲侨报 | --- | --- |
| 中非新闻 | --- | --- |
| **Guatemala (Jan.23-Apr.18, 2020)** | | |
| **Newspaper** | **Date** | **News Title** |
| 南美侨报-危地马拉心声 | --- | --- |
| **Uruguay (Jan.23-Apr.18, 2020)** | | |
| **Newspaper** | **Date** | **News Title** |
| --- | --- | --- |
| **Chile (Jan.23-Apr.18, 2020)** | | |
| **Newspaper** | **Date** | **News Title** |
| 智利中文网 | 2/7/2020 | [智利华助中心主任王何兴谴责对来自中国包裹也带来病毒的言论](https://weixin.sogou.com/link?url=dn9a_-gY295K0Rci_xozVXfdMkSQTLW6cwJThYulHEtVjXrGTiVgSyYKEvoYXj7ucar4mfqq910MNh4svKnPglqXa8Fplpd9Nxp4MP_3njf8JLl3lEB8jI2vS53gcFWMAhIz6IhiVi6dek363Jx2HFLT4-fDHYuQTBWjyn9LhlgVKZwTriwKlFD3Hjnj82FGA3m2DVNNnL_oddsJRpoqJ04FtETln6) |
| 智利中文网 | 2/9/2020 | [智利华助中心温馨提醒侨胞](https://weixin.sogou.com/link?url=dn9a_-gY295K0Rci_xozVXfdMkSQTLW6cwJThYulHEtVjXrGTiVgSyYKEvoYXj7uEd2SBVvIgpoMNh4svKnPglqXa8Fplpd9Nxp4MP_3njf8JLl3lEB8jI2vS53gcFWMAhIz6IhiVi6dek363Jx2HFYV0CJFJtrnpxnQb77fstuod-YYTghpvFFPnMlkwUznAkZ7ECVRJJIsn7FQXkA9Ttw6EbUpX6) |
| 智利中文网 | 2/15/2020 | [美国华裔演员吐槽：病毒让一些人变蠢 谣言比病毒更可怕](https://weixin.sogou.com/link?url=dn9a_-gY295K0Rci_xozVXfdMkSQTLW6cwJThYulHEtVjXrGTiVgSyYKEvoYXj7uighDdm5IUj8MNh4svKnPglqXa8Fplpd9Nxp4MP_3njf8JLl3lEB8jI2vS53gcFWMAhIz6IhiVi6dek363Jx2HMF7fyJKbuiUCLQphPFiDPaS0J7tdOTeO9K3f3J02EDNXZ261v2fu8JeYjMSzS8cS_nIqRD6bp) |
| 智利中文网 | 2/29/2020 | [海外侨胞反击歧视：挑战面前当休戚与共](https://weixin.sogou.com/link?url=dn9a_-gY295K0Rci_xozVXfdMkSQTLW6cwJThYulHEtVjXrGTiVgSyYKEvoYXj7uOmQ49GrALL4MNh4svKnPglqXa8Fplpd9Nxp4MP_3njf8JLl3lEB8jI2vS53gcFWMAhIz6IhiVi6dek363Jx2HOX8KvTiqKZU7dpSXM2bAyd-79xz9Q6haXU-fCRCgCjLUhjk8pk6YnMyziTNg7w-58IW3IPF7A) |
| 智利中文网 | 3/1/2020 | [疫情之下，华人的这一举动让意大利人反感](https://weixin.sogou.com/link?url=dn9a_-gY295K0Rci_xozVXfdMkSQTLW6cwJThYulHEtVjXrGTiVgSyYKEvoYXj7u7IUaQwuJTlsMNh4svKnPglqXa8Fplpd9Nxp4MP_3njf8JLl3lEB8jI2vS53gcFWMAhIz6IhiVi6dek363Jx2HCExS0sXk6ZUWVPSnr5aK_HmAQWQXINq1AY1-rlfMg4FCJiLKnp8-EQADsGBsisgtoKEbybeeP) |
| 智利中文网 | 3/15/2020 | [智利冠状病毒感染升到61例 戴上口罩吓跑顾客与受到歧视怎么办？](https://weixin.sogou.com/link?url=dn9a_-gY295K0Rci_xozVXfdMkSQTLW6cwJThYulHEtVjXrGTiVgSyYKEvoYXj7uZPVcVuk2IqIMNh4svKnPglqXa8Fplpd9Nxp4MP_3njf8JLl3lEB8jI2vS53gcFWMAhIz6IhiVi6dek363Jx2HEwV4zendT7dFE4jWePWxLIcb6RxORp8jdSGUKvtZ48p7xpe9DhFwr7eWGCXbcDz-pUr1EUsUR) |
| 智利中文网 | 3/16/2020 | [借着疫情妖魔化中国，西方媒体用了这三招](https://weixin.sogou.com/link?url=dn9a_-gY295K0Rci_xozVXfdMkSQTLW6cwJThYulHEtVjXrGTiVgSyYKEvoYXj7u_yAn4yhwhgoMNh4svKnPglqXa8Fplpd9Nxp4MP_3njf8JLl3lEB8jI2vS53gcFWMAhIz6IhiVi6dek363Jx2HHzEnI6dhvuUAUsk2AIVSZHZvsdtoBLs3kwtbj2w2Q7IkM_6_e6kSdFkMoKZEDddGEjC9AVaiZ) |
| 智利中文网 | 3/16/2020 | [智利冠状病毒感染升到75例 每一位侨胞都是防疫专家！顾好自己顾好员工！](https://weixin.sogou.com/link?url=dn9a_-gY295K0Rci_xozVXfdMkSQTLW6cwJThYulHEtVjXrGTiVgSyYKEvoYXj7uYxrxVnzOxWsMNh4svKnPglqXa8Fplpd9Nxp4MP_3njf8JLl3lEB8jI2vS53gcFWMAhIz6IhiVi6dek363Jx2HGo749jh7MuK_WyAPdjGUfUZpdm5rLkfphvb3nKCf-qXj_T44xQaUCGAzDTnMb_Zwdo_dNyECS) |
| 智利中文网 | 3/23/2020 | [中国外交部：同胞一切安好，祖国便是晴天 致敬疫情之下坚守海外的侨胞们](https://weixin.sogou.com/link?url=dn9a_-gY295K0Rci_xozVXfdMkSQTLW6cwJThYulHEtVjXrGTiVgSyYKEvoYXj7uLmo0i5EXWQQMNh4svKnPglqXa8Fplpd9Nxp4MP_3njf8JLl3lEB8jI2vS53gcFWMAhIz6IhiVi6dek363Jx2HHXCq5dCDT8rXHkLEKQbyd6KJ2PwOt0RgNZbODeYTcFH6YsZwwsp4MjyYTAVy1-Crl10J_M4E0) |
| 智利中文网 | 4/19/2020 | 确诊新冠病例9730例！智利华人如何应对“中国病毒”的反华攻击？ |
| 南美侨报-智利之窗 | --- | --- |
| **Kuwait (Jan.23-Apr.18, 2020)** | | |
| **Newspaper** | **Date** | **News Title** |
| 中阿网 | --- | --- |
| **Lebanon (Jan.23-Apr.16, 2020)** | | |
| **Newspaper** | **Date** | **News Title** |
| 中阿网 | --- | --- |

**S11 Table. Alternative Mediators.**

| Exploratory Study | Mediator: National Identity, DV: Anger | | |  |
| --- | --- | --- | --- | --- |
| Predictor:  Traditional Media Use | Direct effect | Indirect effect | Total effect | P*_M_* |
|  | .095* [-.057, .246]  *t* = 1.226 | .081*** [.034, .139] | .176* [ .022, .330]  *t* = 2.243 | 46.02% |
|  | Mediator: Anger, DV: National Identity | | |  |
|  | Direct effect | Indirect effect | Total effect | P*_M_* |
|  | .174** [.068, .280]  t = 3.227 | .034** [.003, .071] | .208*** [.099, .317]  t = 3.750 | 16.34% |

***p<=0.001, **p<=0.01, *p<=0.5; P*_M_* refers to the ratio of the indirect effect to the total effect (Wen & Fan, 2015).

**S12 Table. Chinese National Identity.** The adapted Collective Self-Esteem Scale (Chinese translation).

我们都是不同社会群体或社会类别中的成员。个人所属的一个社会类别便是**自己的国家**。在本问卷中，我们希望您思考自己在**中国**这一团体中的身份，从个人感受出发回答下列问题。答案没有对错，我们希望获得您真实的想法。请仔细阅读每个问题并作出回答：

| I am a worthy member of the nation I belong to.  我是我的国家有价值的一员。 |
| --- |
| I often regret that I belong to this nation. (Reversed)  我时常后悔自己属于这个国家。 |
| Overall, my nation is considered good by others.  总体而言，我的国家在别人看来是好的。 |
| Overall, my nation has very little to do with how I feel about myself. (Reversed)  总体而言，我的国家与我对自己的感受没有多大关系。 |
| I feel I don’t have much to offer to the nation I belong to. (Reversed)  我感到对于自己所属的国家，我没有什么可贡献的。 |
| In general, I’m glad to be a member of the nation I belong to.  总体而言，我很高兴自己是这个国家的一员。 |
| Most people consider my nation to be more ineffective than other nations. (Reversed)  大多数人觉得我的国家比其他国家效率要低。 |
| The nation I belong to is an important reflection of who I am.  我所属的国家很大程度上决定了我是谁。 |
| I am a cooperative participant of the nation I belong to.  我是国家当中积极合作的参与者。 |
| Overall, I often feel that the nation of which I am a member is not worthwhile. (Reversed)  总体而言，我常感到我的国家没有价值。 |
| In general, others respect the nation that I am a member of.  总体而言，他人尊重我的国家。 |
| The nation I belong to is unimportant to my sense of what kind of a person I am. (Reversed)  我的国家对于我觉得自己是怎样一个人来说并不重要。 |
| I often feel I’m a useless member of my nation. (Reversed)  我常觉得自己是国家当中没有用的一员。 |
| I feel good about the nation I belong to.  我对我的国家感觉良好。 |
| In general, others think that the nation I am a member of is unworthy. (Reversed)  总体而言，他人认为我所属的国家没有价值。 |
| In general, belonging to this nation is an important part of my self-image  总体而言，属于这个国家是我个人形象重要的一部分。 |

**S13 Table. Sino Distance and Sino Favoritism.** Host country’s cultural distance from China, favorable attitude toward China, and trade export change before COVID-19.

| **Country** | **Sino Distance** | **Sino Favoritism**  **(Pew 2019)** | **2019 Export to China (billion USD)** | **2018 Export to China (billion USD)** | **2019-2018 Export Change (billion USD)** | **Num. of Reports Jan.30-31, 2020** |
| --- | --- | --- | --- | --- | --- | --- |
| **USA** | 0.150 | 2.01 | 123.236 | 156.004 | -32.768 | 2 |
| **Spain** | 0.137 | 2.36 | 8.609 | 8.793 | -0.184 | 0 |
| **UK** | 0.172 | 2.23 | 23.828 | 23.893 | -0.065 | 3 |
| **Italy** | 0.163 | 2.21 | 21.421 | 21.154 | 0.267 | 2 |
| **Germany** | 0.114 | 2.26 | 105.037 | 106.257 | -1.22 | 0 |
| **Australia** | 0.131 | 2.20 | 119.608 | 105.083 | 14.525 | 0 |
| **Japan** | 0.118 | 1.77 | 171.523 | 180.402 | -8.879 | 0 |
| **South Korea** | 0.073 | 2.17 | 0.216 | 0.213 | 0.003 | 12 |
| **Russia** | 0.089 | 2.95 | 60.257 | 58.887 | 1.37 | 0 |
| **Peru** | 0.142 | 2.87 | 15.212 | 15.213 | -0.001 | 0 |
| **France** | 0.181 | 2.16 | 32.581 | 32.330 | 0.251 | 3 |
| **Singapore** | 0.124 | 2.24 (mean) | 35.230 | 33.638 | 1.592 | 1 |
| **Brazil** | 0.159 | 2.66 | 79.204 | 77.142 | 2.062 | 3 |
| **Malaysia** | 0.156 | 2.24 (mean) | 71.630 | 63.322 | 8.308 | 1 |
| **Canada** | 0.135 | 2.04 | 28.032 | 28.354 | -0.322 | 3 |
| **Angola** | 0.162 | 2.24 (mean) | 23.308 | 25.652 | -2.344 | 0 |
| **Belgium** | 0.147 | 1.98 (2020) | 6.870 | 6.988 | -0.118 | 3 |
| **Portugal** | 0.137 | 2.24 (mean) | 2.318 | 2.250 | 0.068 | 0 |
| **India** | 0.106 | 1.99 | 17.970 | 18.850 | -0.88 | 0 |
| **Greece** | 0.118 | 2.57 | 0.726 | 0.565 | 0.161 | 0 |
| **Sweden** | 0.186 | 2.03 | 9.137 | 8.947 | 0.19 | 0 |
| **Saudi Arabia** | 0.212 | 2.24 (mean) | 54.257 | 45.899 | 8.358 | 0 |
| **The Netherlands** | 0.146 | 2.24 | 11.196 | 12.314 | -1.118 | 2 |
| **Switzerland** | 0.179 | 2.24 (mean) | 27.398 | 38.796 | -11.398 | 2 |
| **Mexico** | 0.138 | 2.68 | 14.349 | 14.054 | 0.295 | 0 |
| **South Africa** | 0.138 | 2.65 | 25.931 | 27.240 | -1.309 | 0 |
| **Vietnam** | 0.057 | 1.73 (2017) | 64.078 | 64.087 | -0.009 | 0 |
| **Kenya** | 0.153 | 2.82 | 0.181 | 0.174 | 0.007 | 0 |
| **Guatemala** | 0.192 | 2.24 (mean) | 0.193 | 0.087 | 0.106 | 0 |
| **Uruguay** | 0.143 | 2.24 (mean) | 2.968 | 2.559 | 0.409 | 1 |
| **Chile** | 0.156 | 2.71 (2017) | 26.291 | 27.000 | -0.709 | 1 |
| **Kuwait** | 0.163 | 2.24 (mean) | 13.417 | 15.359 | -1.942 | 0 |
| **Lebanon** | 0.175 | 2.89 | 0.026 | 0.049 | -0.023 | 0 |

**S14 Table. Correlation Analyses.** Export-to-China trade change as a proxy variable validating the measurement of Sino Favoritism.

| N=326 | 1 | 2 | 3 | 4 | 5 | 6 | 7 |  | M | SD |
| --- | --- | --- | --- | --- | --- | --- | --- | --- | --- | --- |
| 1. Chinese Population (million) | - |  |  |  |  |  |  |  | 1.30 | 1.84 |
| 1. Host Newspaper Coverage | 0.35*** | - |  |  |  |  |  |  | 16.34 | 9.29 |
| 1. Ethnic Newspaper Coverage | 0.91*** | 0.29*** | - |  |  |  |  |  | 49.81 | 53.37 |
| 1. Sino Favoritism | -0.50*** | -0.33*** | -0.38*** | - |  |  |  |  | 2.24 | 0.26 |
| 1. Trade Change (billion $) | -0.90*** | -0.26*** | -0.88*** | 0.46*** | - |  |  |  | -5.58 | 13.55 |
| 1. Sino Distance | 0.13* | 0.04 | 0.18** | -0.15** | -0.13* | - |  |  | 0.14 | 0.03 |
| 1. Anger | 0.02 | 0.12* | 0.01 | 0.06 | 0.01 | -0.02 | - |  | 4.22 | 0.70 |
| 1. National Identity | -0.01 | 0.12* | 0.01 | 0.19** | 0.04 | -0.09 | 0.29*** | - | 3.95 | 0.50 |

***p<=0.001, **p<=0.01, *p<=0.5; Host/Ethnic Newspaper Coverage refers to the number of news reports on anti-Chinese discrimination in each country (Jan.23 - Apr.23, 2020) host and ethnic media. Chinese population was measured per the request by one of the reviewers, but not included in the formal analyses.
